# Supplementary material for: Excess mortality in England and Wales during the first wave of the COVID-19 pandemic
Source: J Epidemiol Community Health. 2020 Oct 15;75(3):213–23. doi: 10.1136/jech-2020-214764 (PMC7892396; doi:10.1136/jech-2020-214764)
Supplement: Supplementary file 2 [file jech-2020-214764supp002.pdf]

Appendix 2 to excess mortality in England and Wales during the 2020 COVID19 pandemic

Evangelos Kontopantelis
Mamas A Mamas
John Deanfield
Miqdad Asaria
Tim Doran

October 7, 2020

Contents

1 England-Wales aggregates **10**
1.1 Time trend . . . 10
1.2 Time trend and Negative Binomial Regression model . . . 12
1.3 Excess number of deaths: all causes . . . 14
1.4 Excess number of deaths: all causes minus COVID19 deaths . . . 16
1.5 Excess number of deaths: all causes minus respiratory deaths . . . 18

2 Regional **20**
2.1 Time trends . . . 20
2.2 North East . . . 22
2.3 North West . . . 27
2.4 Yorkshire and the Humber . . . 32
2.5 East Midlands . . . 37
2.6 West Midlands . . . 42
2.7 East . . . 47
2.8 London . . . 52
2.9 South East . . . 57
2.10 South West . . . 62
2.11 Wales . . . 67

3 Age groups, people **72**
3.1 Time trends . . . 72
3.2 All aged under 1 . . . 74
3.3 All aged 01-14 . . . 79
3.4 All aged 15-44 . . . 84
3.5 All aged 45-64 . . . 89

3.6 All aged 65-74 94
3.7 All aged 75-84 99
3.8 All aged 85+ 104

4 Age groups, males 109
4.1 Time trends 109
4.2 Males aged under 1 111
4.3 Males aged 01-14 116
4.4 Males aged 15-44 121
4.5 Males aged 45-64 126
4.6 Males aged 65-74 131
4.7 Males aged 75-84 136
4.8 Males aged 85+ 141

5 Age groups, females 146
5.1 Time trends 146
5.2 Females aged under 1 148
5.3 Females aged 01-14 153
5.4 Females aged 15-44 158
5.5 Females aged 45-64 163
5.6 Females aged 65-74 168
5.7 Females aged 75-84 173
5.8 Females aged 85+ 178

List of Figures

1 Mortality time trend, from 2010 week 1 10
2 Mortality time trend, from 2019 week 1 11
3 Mortality time trend, from 2020 week 1 11
4 Mortality time trend and model, from 2010 week 1 12
5 Mortality time trend and model, from 2019 week 1 13
6 Mortality time trend and model, from 2020 week 1 13
7 All-cause excess deaths, from 2010 week 1 14
8 All-cause excess deaths, from 2019 week 1 15
9 All-cause excess deaths, from 2020 week 1 15
10 All-cause excess deaths minus COVID19, from 2010 week 1 . . . 16
11 All-cause excess deaths minus COVID19, from 2019 week 1 . . . 17
12 All-cause excess deaths minus COVID19, from 2020 week 1 . . . 17
13 All-cause excess deaths minus COVID19, from 2010 week 1 . . . 18
14 All-cause excess deaths minus COVID19, from 2019 week 1 . . . 19
15 All-cause excess deaths minus COVID19, from 2020 week 1 . . . 19
16 Regional mortality time trends, from 2010 week 1 20
17 Regional mortality time trends, from 2019 week 1 21
18 Regional mortality time trends, from 2020 week 1 21
19 NE Mortality time trend and model, from 2010 week 1 22

20 NE Mortality time trend and model, from 2019 week 1 23
21 NE Mortality time trend and model, from 2020 week 1 23
22 NE all-cause excess deaths, from 2010 week 1 24
23 NE all-cause excess deaths, from 2019 week 1 24
24 NE all-cause excess deaths, from 2020 week 1 25
25 NE all-cause excess deaths minus COVID19, from 2010 week 1 . 25
26 NE all-cause excess deaths minus COVID19, from 2019 week 1 . 26
27 NE all-cause excess deaths minus COVID19, from 2020 week 1 . 26
28 NW Mortality time trend and model, from 2010 week 1 27
29 NW Mortality time trend and model, from 2019 week 1 28
30 NW Mortality time trend and model, from 2020 week 1 28
31 NW all-cause excess deaths, from 2010 week 1 29
32 NW all-cause excess deaths, from 2019 week 1 29
33 NW all-cause excess deaths, from 2020 week 1 30
34 NW all-cause excess deaths minus COVID19, from 2010 week 1 . 30
35 NW all-cause excess deaths minus COVID19, from 2019 week 1 . 31
36 NW all-cause excess deaths minus COVID19, from 2020 week 1 . 31
37 YatH Mortality time trend and model, from 2010 week 1 32
38 YatH Mortality time trend and model, from 2019 week 1 33
39 YatH Mortality time trend and model, from 2020 week 1 33
40 YatH all-cause excess deaths, from 2010 week 1 34
41 YatH all-cause excess deaths, from 2019 week 1 34
42 YatH all-cause excess deaths, from 2020 week 1 35
43 YatH all-cause excess deaths minus COVID19, from 2010 week 1 35
44 YatH all-cause excess deaths minus COVID19, from 2019 week 1 36
45 YatH all-cause excess deaths minus COVID19, from 2020 week 1 36
46 EMD Mortality time trend and model, from 2010 week 1 37
47 EMD Mortality time trend and model, from 2019 week 1 38
48 EMD Mortality time trend and model, from 2020 week 1 38
49 EMD all-cause excess deaths, from 2010 week 1 39
50 EMD all-cause excess deaths, from 2019 week 1 39
51 EMD all-cause excess deaths, from 2020 week 1 40
52 EMD all-cause excess deaths minus COVID19, from 2010 week 1 40
53 EMD all-cause excess deaths minus COVID19, from 2019 week 1 41
54 EMD all-cause excess deaths minus COVID19, from 2020 week 1 41
55 WMD Mortality time trend and model, from 2010 week 1 42
56 WMD Mortality time trend and model, from 2019 week 1 43
57 WMD Mortality time trend and model, from 2020 week 1 43
58 WMD all-cause excess deaths, from 2010 week 1 44
59 WMD all-cause excess deaths, from 2019 week 1 44
60 WMD all-cause excess deaths, from 2020 week 1 45
61 WMD all-cause excess deaths minus COVID19, from 2010 week 1 45
62 WMD all-cause excess deaths minus COVID19, from 2019 week 1 46
63 WMD all-cause excess deaths minus COVID19, from 2020 week 1 46
64 East Mortality time trend and model, from 2010 week 1 47
65 East Mortality time trend and model, from 2019 week 1 48

66 East Mortality time trend and model, from 2020 week 1 48
67 East all-cause excess deaths, from 2010 week 1 49
68 East all-cause excess deaths, from 2019 week 1 49
69 East all-cause excess deaths, from 2020 week 1 50
70 East all-cause excess deaths minus COVID19, from 2010 week 1 . 50
71 East all-cause excess deaths minus COVID19, from 2019 week 1 . 51
72 East all-cause excess deaths minus COVID19, from 2020 week 1 . 51
73 London Mortality time trend and model, from 2010 week 1 . . . 52
74 London Mortality time trend and model, from 2019 week 1 . . . 53
75 London Mortality time trend and model, from 2020 week 1 . . . 53
76 London all-cause excess deaths, from 2010 week 1 54
77 London all-cause excess deaths, from 2019 week 1 54
78 London all-cause excess deaths, from 2020 week 1 55
79 London all-cause excess deaths minus COVID19, from 2010 week 1 55
80 London all-cause excess deaths minus COVID19, from 2019 week 1 56
81 London all-cause excess deaths minus COVID19, from 2020 week 1 56
82 SE Mortality time trend and model, from 2010 week 1 57
83 SE Mortality time trend and model, from 2019 week 1 58
84 SE Mortality time trend and model, from 2020 week 1 58
85 SE all-cause excess deaths, from 2010 week 1 59
86 SE all-cause excess deaths, from 2019 week 1 59
87 SE all-cause excess deaths, from 2020 week 1 60
88 SE all-cause excess deaths minus COVID19, from 2010 week 1 . . 60
89 SE all-cause excess deaths minus COVID19, from 2019 week 1 . . 61
90 SE all-cause excess deaths minus COVID19, from 2020 week 1 . . 61
91 SW Mortality time trend and model, from 2010 week 1 62
92 SE Mortality time trend and model, from 2019 week 1 63
93 SW Mortality time trend and model, from 2020 week 1 63
94 SW all-cause excess deaths, from 2010 week 1 64
95 SW all-cause excess deaths, from 2019 week 1 64
96 SW all-cause excess deaths, from 2020 week 1 65
97 SW all-cause excess deaths minus COVID19, from 2010 week 1 . 65
98 SW all-cause excess deaths minus COVID19, from 2019 week 1 . 66
99 SW all-cause excess deaths minus COVID19, from 2020 week 1 . 66
100 Wales Mortality time trend and model, from 2010 week 1 67
101 SE Mortality time trend and model, from 2019 week 1 68
102 Wales Mortality time trend and model, from 2020 week 1 68
103 Wales all-cause excess deaths, from 2010 week 1 69
104 Wales all-cause excess deaths, from 2019 week 1 69
105 Wales all-cause excess deaths, from 2020 week 1 70
106 Wales all-cause excess deaths minus COVID19, from 2010 week 1 70
107 Wales all-cause excess deaths minus COVID19, from 2019 week 1 71
108 Wales all-cause excess deaths minus COVID19, from 2020 week 1 71
109 All age group mortality time trends, from 2010 week 1 72
110 All age group mortality time trends, from 2019 week 1 73
111 All age group mortality time trends, from 2020 week 1 73

112 All < 1 mortality time trend and model, from 2010 week 1 74
113 All < 1 mortality time trend and model, from 2019 week 1 75
114 All < 1 mortality time trend and model, from 2020 week 1 75
115 All < 1 all-cause excess deaths, from 2010 week 1 76
116 All < 1 all-cause excess deaths, from 2019 week 1 76
117 All < 1 all-cause excess deaths, from 2020 week 1 77
118 All < 1 all-cause excess deaths minus COVID19, from 2010 week 1 77
119 All < 1 all-cause excess deaths minus COVID19, from 2019 week 1 78
120 All < 1 all-cause excess deaths minus COVID19, from 2020 week 1 78
121 All 01-14 mortality time trend and model, from 2010 week 1 . . . 79
122 All 01-14 mortality time trend and model, from 2019 week 1 . . . 80
123 All 01-14 mortality time trend and model, from 2020 week 1 . . . 80
124 All 01-14 all-cause excess deaths, from 2010 week 1 81
125 All 01-14 all-cause excess deaths, from 2019 week 1 81
126 All 01-14 all-cause excess deaths, from 2020 week 1 82
127 All 01-14 all-cause excess deaths (−COVID19), from 2010 week 1 82
128 All 01-14 all-cause excess deaths (−COVID19), from 2019 week 1 83
129 All 01-14 all-cause excess deaths (−COVID19), from 2020 week 1 83
130 All 15-44 mortality time trend and model, from 2010 week 1 . . . 84
131 All 15-44 mortality time trend and model, from 2019 week 1 . . . 85
132 All 15-44 mortality time trend and model, from 2020 week 1 . . . 85
133 All 15-44 all-cause excess deaths, from 2010 week 1 86
134 All 15-44 all-cause excess deaths, from 2019 week 1 86
135 All 15-44 all-cause excess deaths, from 2020 week 1 87
136 All 15-44 all-cause excess deaths (−COVID19), from 2010 week 1 87
137 All 15-44 all-cause excess deaths (−COVID19), from 2019 week 1 88
138 All 15-44 all-cause excess deaths (−COVID19), from 2020 week 1 88
139 All 45-64 mortality time trend and model, from 2010 week 1 . . . 89
140 All 45-64 mortality time trend and model, from 2019 week 1 . . . 90
141 All 45-64 mortality time trend and model, from 2020 week 1 . . . 90
142 All 45-64 all-cause excess deaths, from 2010 week 1 91
143 All 45-64 all-cause excess deaths, from 2019 week 1 91
144 All 45-64 all-cause excess deaths, from 2020 week 1 92
145 All 45-64 all-cause excess deaths (−COVID19), from 2010 week 1 92
146 All 45-64 all-cause excess deaths (−COVID19), from 2019 week 1 93
147 All 45-64 all-cause excess deaths (−COVID19), from 2020 week 1 93
148 All 65-74 mortality time trend and model, from 2010 week 1 . . . 94
149 All 65-74 mortality time trend and model, from 2019 week 1 . . . 95
150 All 65-74 mortality time trend and model, from 2020 week 1 . . . 95
151 All 65-74 all-cause excess deaths, from 2010 week 1 96
152 All 65-74 all-cause excess deaths, from 2019 week 1 96
153 All 65-74 all-cause excess deaths, from 2020 week 1 97
154 All 65-74 all-cause excess deaths (−COVID19), from 2010 week 1 97
155 All 65-74 all-cause excess deaths (−COVID19), from 2019 week 1 98
156 All 65-74 all-cause excess deaths (−COVID19), from 2020 week 1 98
157 All 75-84 mortality time trend and model, from 2010 week 1 . . . 99

158 All 75-84 mortality time trend and model, from 2019 week 1 . . . 100
159 All 75-84 mortality time trend and model, from 2020 week 1 . . . 100
160 All 75-84 all-cause excess deaths, from 2010 week 1 101
161 All 75-84 all-cause excess deaths, from 2019 week 1 101
162 All 75-84 all-cause excess deaths, from 2020 week 1 102
163 All 75-84 all-cause excess deaths (−COVID19), from 2010 week 1 102
164 All 75-84 all-cause excess deaths (−COVID19), from 2019 week 1 103
165 All 75-84 all-cause excess deaths (−COVID19), from 2020 week 1 103
166 All 85+ mortality time trend and model, from 2010 week 1 . . . 104
167 All 85+ mortality time trend and model, from 2019 week 1 . . . 105
168 All 85+ mortality time trend and model, from 2020 week 1 . . . 105
169 All 85+ all-cause excess deaths, from 2010 week 1 106
170 All 85+ all-cause excess deaths, from 2019 week 1 106
171 All 85+ all-cause excess deaths, from 2020 week 1 107
172 All 85+ all-cause excess deaths (−COVID19), from 2010 week 1 107
173 All 85+ all-cause excess deaths (−COVID19), from 2019 week 1 108
174 All 85+ all-cause excess deaths (−COVID19), from 2020 week 1 108
175 Male age group mortality time trends, from 2010 wk1 109
176 Male age group mortality time trends, from 2019 wk1 110
177 Male age group mortality time trends, from 2020 wk1 110
178 Males < 1 mortality time trend and model, from 2010 wk1 111
179 Males < 1 mortality time trend and model, from 2019 wk1 112
180 Males < 1 mortality time trend and model, from 2020 wk1 112
181 Males < 1 all-cause excess deaths, from 2010 wk1 113
182 Males < 1 all-cause excess deaths, from 2019 wk1 113
183 Males < 1 all-cause excess deaths, from 2020 wk1 114
184 Males < 1 all-cause excess deaths minus COVID19, from 2010 wk1 114
185 Males < 1 all-cause excess deaths minus COVID19, from 2019 wk1 115
186 Males < 1 all-cause excess deaths minus COVID19, from 2020 wk1 115
187 Males 01-14 mortality time trend and model, from 2010 wk1 . . . 116
188 Males 01-14 mortality time trend and model, from 2019 wk1 . . . 117
189 Males 01-14 mortality time trend and model, from 2020 wk1 . . . 117
190 Males 01-14 all-cause excess deaths, from 2010 wk1 118
191 Males 01-14 all-cause excess deaths, from 2019 wk1 118
192 Males 01-14 all-cause excess deaths, from 2020 wk1 119
193 Males 01-14 all-cause excess deaths (−COVID19), from 2010 wk1 119
194 Males 01-14 all-cause excess deaths (−COVID19), from 2019 wk1 120
195 Males 01-14 all-cause excess deaths (−COVID19), from 2020 wk1 120
196 Males 15-44 mortality time trend and model, from 2010 wk1 . . . 121
197 Males 15-44 mortality time trend and model, from 2019 wk1 . . . 122
198 Males 15-44 mortality time trend and model, from 2020 wk1 . . . 122
199 Males 15-44 all-cause excess deaths, from 2010 wk1 123
200 Males 15-44 all-cause excess deaths, from 2019 wk1 123
201 Males 15-44 all-cause excess deaths, from 2020 wk1 124
202 Males 15-44 all-cause excess deaths (−COVID19), from 2010 wk1 124
203 Males 15-44 all-cause excess deaths (−COVID19), from 2019 wk1 125

204 Males 15-44 all-cause excess deaths (−COVID19), from 2020 wk1 125
205 Males 45-64 mortality time trend and model, from 2010 wk1 . . . 126
206 Males 45-64 mortality time trend and model, from 2019 wk1 . . . 127
207 Males 45-64 mortality time trend and model, from 2020 wk1 . . . 127
208 Males 45-64 all-cause excess deaths, from 2010 wk1 128
209 Males 45-64 all-cause excess deaths, from 2019 wk1 128
210 Males 45-64 all-cause excess deaths, from 2020 wk1 129
211 Males 45-64 all-cause excess deaths (−COVID19), from 2010 wk1 129
212 Males 45-64 all-cause excess deaths (−COVID19), from 2019 wk1 130
213 Males 45-64 all-cause excess deaths (−COVID19), from 2020 wk1 130
214 Males 65-74 mortality time trend and model, from 2010 wk1 . . . 131
215 Males 65-74 mortality time trend and model, from 2019 wk1 . . . 132
216 Males 65-74 mortality time trend and model, from 2020 wk1 . . . 132
217 Males 65-74 all-cause excess deaths, from 2010 wk1 133
218 Males 65-74 all-cause excess deaths, from 2019 wk1 133
219 Males 65-74 all-cause excess deaths, from 2020 wk1 134
220 Males 65-74 all-cause excess deaths (−COVID19), from 2010 wk1 134
221 Males 65-74 all-cause excess deaths (−COVID19), from 2019 wk1 135
222 Males 65-74 all-cause excess deaths (−COVID19), from 2020 wk1 135
223 Males 75-84 mortality time trend and model, from 2010 wk1 . . . 136
224 Males 75-84 mortality time trend and model, from 2019 wk1 . . . 137
225 Males 75-84 mortality time trend and model, from 2020 wk1 . . . 137
226 Males 75-84 all-cause excess deaths, from 2010 wk1 138
227 Males 75-84 all-cause excess deaths, from 2019 wk1 138
228 Males 75-84 all-cause excess deaths, from 2020 wk1 139
229 Males 75-84 all-cause excess deaths (−COVID19), from 2010 wk1 139
230 Males 75-84 all-cause excess deaths (−COVID19), from 2019 wk1 140
231 Males 75-84 all-cause excess deaths (−COVID19), from 2020 wk1 140
232 Males 85+ mortality time trend and model, from 2010 wk1 . . . 141
233 Males 85+ mortality time trend and model, from 2019 wk1 . . . 142
234 Males 85+ mortality time trend and model, from 2020 wk1 . . . 142
235 Males 85+ all-cause excess deaths, from 2010 wk1 143
236 Males 85+ all-cause excess deaths, from 2019 wk1 143
237 Males 85+ all-cause excess deaths, from 2020 wk1 144
238 Males 85+ all-cause excess deaths (−COVID19), from 2010 wk1 144
239 Males 85+ all-cause excess deaths (−COVID19), from 2019 wk1 145
240 Males 85+ all-cause excess deaths (−COVID19), from 2020 wk1 145
241 Female age group mortality time trends, fm 2010 wk1 146
242 Female age group mortality time trends, fm 2019 wk1 147
243 Female age group mortality time trends, fm 2020 wk1 147
244 Females < 1 mortality time trend and model, fm 2010 wk1 . . . 148
245 Females < 1 mortality time trend and model, fm 2019 wk1 . . . 149
246 Females < 1 mortality time trend and model, fm 2020 wk1 . . . 149
247 Females < 1 all-cause excess deaths, fm 2010 wk1 150
248 Females < 1 all-cause excess deaths, fm 2019 wk1 150
249 Females < 1 all-cause excess deaths, fm 2020 wk1 151

250 Females < 1 all-cause excess deaths minus COVID19, fm 2010 wk1 151
251 Females < 1 all-cause excess deaths minus COVID19, fm 2019 wk1 152
252 Females < 1 all-cause excess deaths minus COVID19, fm 2020 wk1 152
253 Females 01-14 mortality time trend and model, fm 2010 wk1 . . 153
254 Females 01-14 mortality time trend and model, fm 2019 wk1 . . 154
255 Females 01-14 mortality time trend and model, fm 2020 wk1 . . 154
256 Females 01-14 all-cause excess deaths, fm 2010 wk1 155
257 Females 01-14 all-cause excess deaths, fm 2019 wk1 155
258 Females 01-14 all-cause excess deaths, fm 2020 wk1 156
259 Females 01-14 all-cause excess deaths (−COVID19), fm 2010 wk1 156
260 Females 01-14 all-cause excess deaths (−COVID19), fm 2019 wk1 157
261 Females 01-14 all-cause excess deaths (−COVID19), fm 2020 wk1 157
262 Females 15-44 mortality time trend and model, fm 2010 wk1 . . 158
263 Females 15-44 mortality time trend and model, fm 2019 wk1 . . 159
264 Females 15-44 mortality time trend and model, fm 2020 wk1 . . 159
265 Females 15-44 all-cause excess deaths, fm 2010 wk1 160
266 Females 15-44 all-cause excess deaths, fm 2019 wk1 160
267 Females 15-44 all-cause excess deaths, fm 2020 wk1 161
268 Females 15-44 all-cause excess deaths (−COVID19), fm 2010 wk1 161
269 Females 15-44 all-cause excess deaths (−COVID19), fm 2019 wk1 162
270 Females 15-44 all-cause excess deaths (−COVID19), fm 2020 wk1 162
271 Females 45-64 mortality time trend and model, fm 2010 wk1 . . 163
272 Females 45-64 mortality time trend and model, fm 2019 wk1 . . 164
273 Females 45-64 mortality time trend and model, fm 2020 wk1 . . 164
274 Females 45-64 all-cause excess deaths, fm 2010 wk1 165
275 Females 45-64 all-cause excess deaths, fm 2019 wk1 165
276 Females 45-64 all-cause excess deaths, fm 2020 wk1 166
277 Females 45-64 all-cause excess deaths (−COVID19), fm 2010 wk1 166
278 Females 45-64 all-cause excess deaths (−COVID19), fm 2019 wk1 167
279 Females 45-64 all-cause excess deaths (−COVID19), fm 2020 wk1 167
280 Females 65-74 mortality time trend and model, fm 2010 wk1 . . 168
281 Females 65-74 mortality time trend and model, fm 2019 wk1 . . 169
282 Females 65-74 mortality time trend and model, fm 2020 wk1 . . 169
283 Females 65-74 all-cause excess deaths, fm 2010 wk1 170
284 Females 65-74 all-cause excess deaths, fm 2019 wk1 170
285 Females 65-74 all-cause excess deaths, fm 2020 wk1 171
286 Females 65-74 all-cause excess deaths (−COVID19), fm 2010 wk1 171
287 Females 65-74 all-cause excess deaths (−COVID19), fm 2019 wk1 172
288 Females 65-74 all-cause excess deaths (−COVID19), fm 2020 wk1 172
289 Females 75-84 mortality time trend and model, fm 2010 wk1 . . 173
290 Females 75-84 mortality time trend and model, fm 2019 wk1 . . 174
291 Females 75-84 mortality time trend and model, fm 2020 wk1 . . 174
292 Females 75-84 all-cause excess deaths, fm 2010 wk1 175
293 Females 75-84 all-cause excess deaths, fm 2019 wk1 175
294 Females 75-84 all-cause excess deaths, fm 2020 wk1 176
295 Females 75-84 all-cause excess deaths (−COVID19), fm 2010 wk1 176

296 Females 75-84 all-cause excess deaths (−COVID19), fm 2019 wk1 177
297 Females 75-84 all-cause excess deaths (−COVID19), fm 2020 wk1 177
298 Females 85+ mortality time trend and model, fm 2010 wk1 . . . 178
299 Females 85+ mortality time trend and model, fm 2019 wk1 . . . 179
300 Females 85+ mortality time trend and model, fm 2020 wk1 . . . 179
301 Females 85+ all-cause excess deaths, fm 2010 wk1 180
302 Females 85+ all-cause excess deaths, fm 2019 wk1 180
303 Females 85+ all-cause excess deaths, fm 2020 wk1 181
304 Females 85+ all-cause excess deaths (−COVID19), fm 2010 wk1 181
305 Females 85+ all-cause excess deaths (−COVID19), fm 2019 wk1 182
306 Females 85+ all-cause excess deaths (−COVID19), fm 2020 wk1 182

1 England-Wales aggregates

1.1 Time trend

Figure 1: Mortality time trend, from 2010 week 1

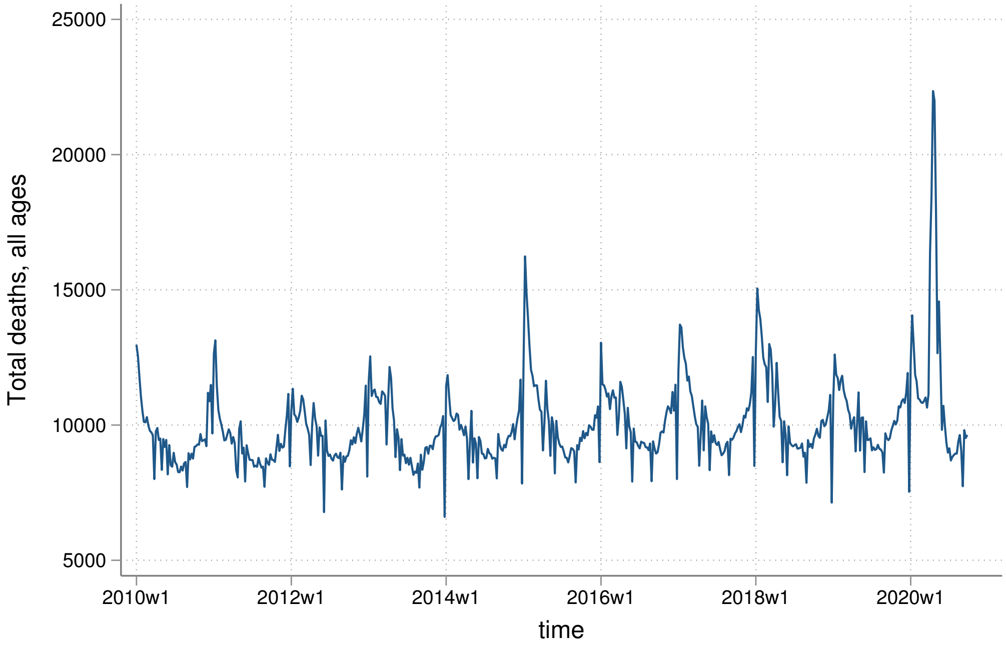

Figure 2: Mortality time trend, from 2019 week 1

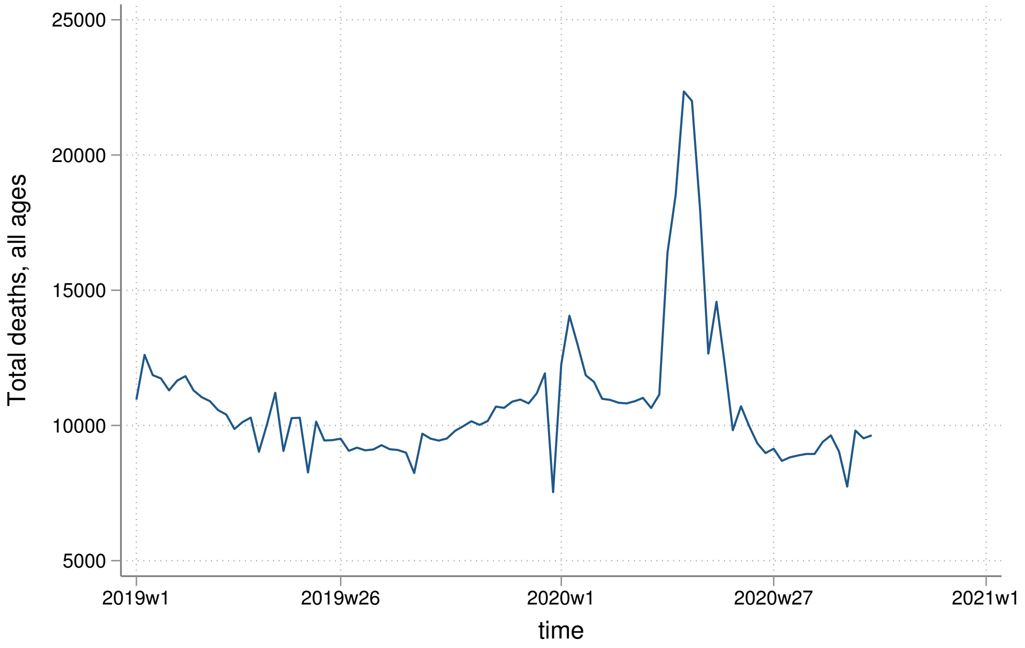

Figure 3: Mortality time trend, from 2020 week 1

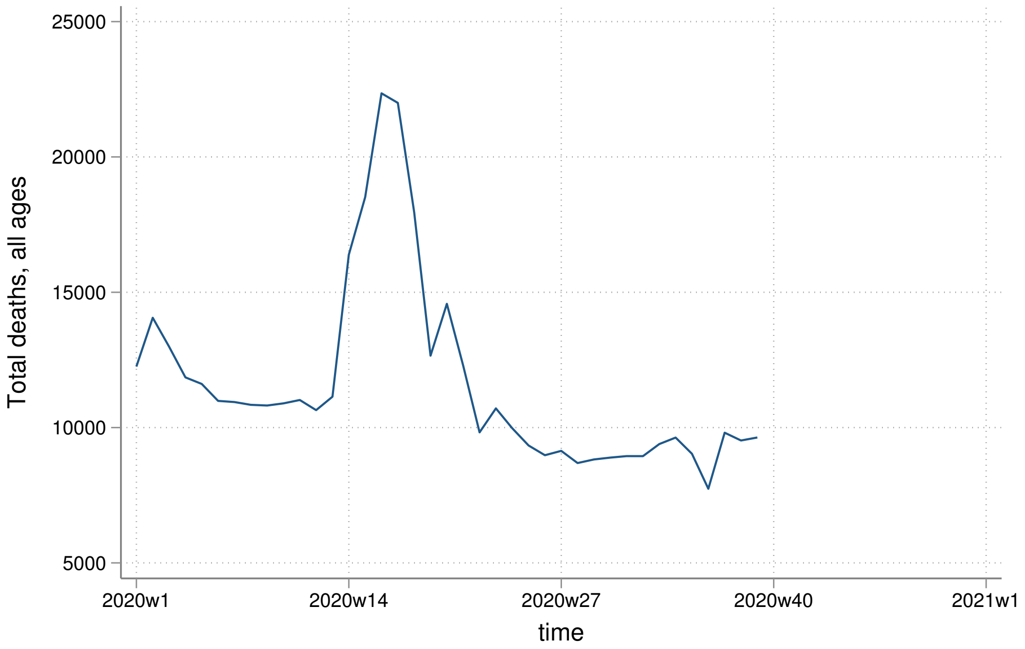

1.2 Time trend and Negative Binomial Regression model

Figure 4: Mortality time trend and model, from 2010 week 1

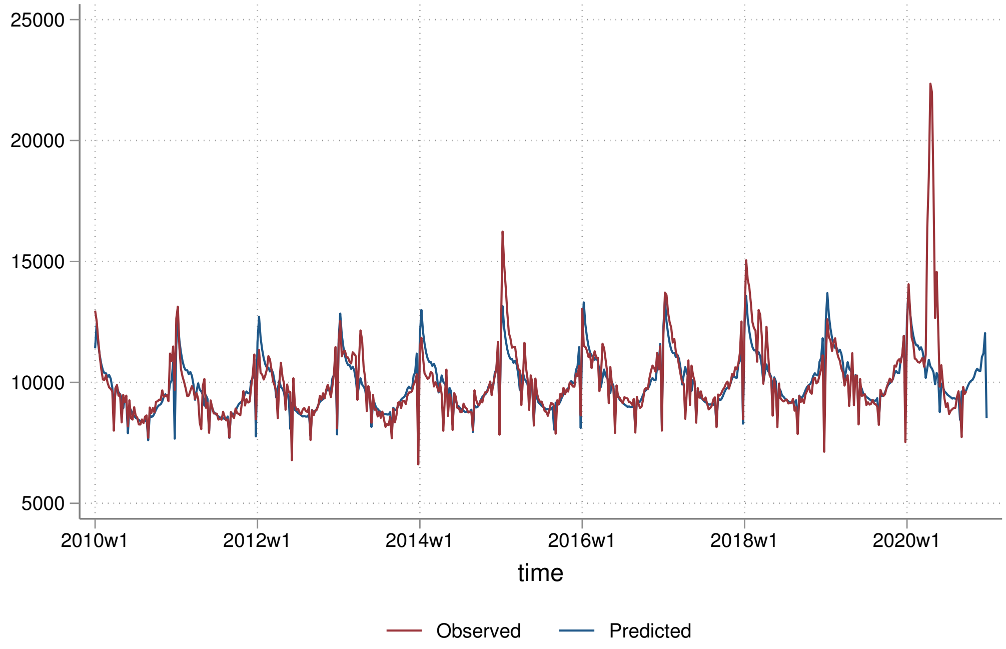

Figure 5: Mortality time trend and model, from 2019 week 1

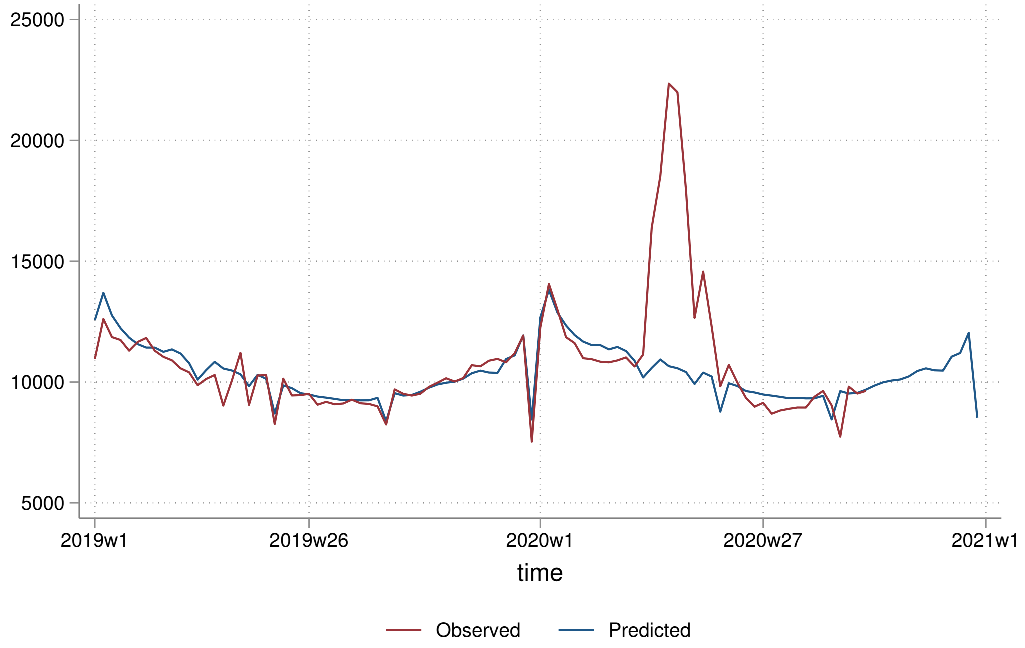

Figure 6: Mortality time trend and model, from 2020 week 1

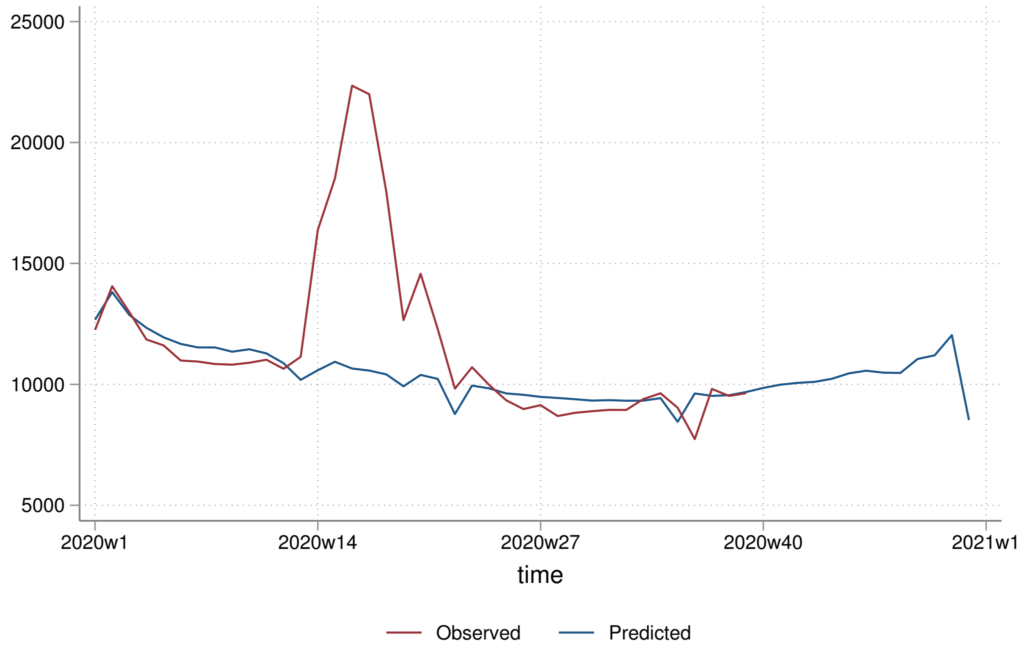

1.3 Excess number of deaths: all causes

Figure 7: All-cause excess deaths, from 2010 week 1

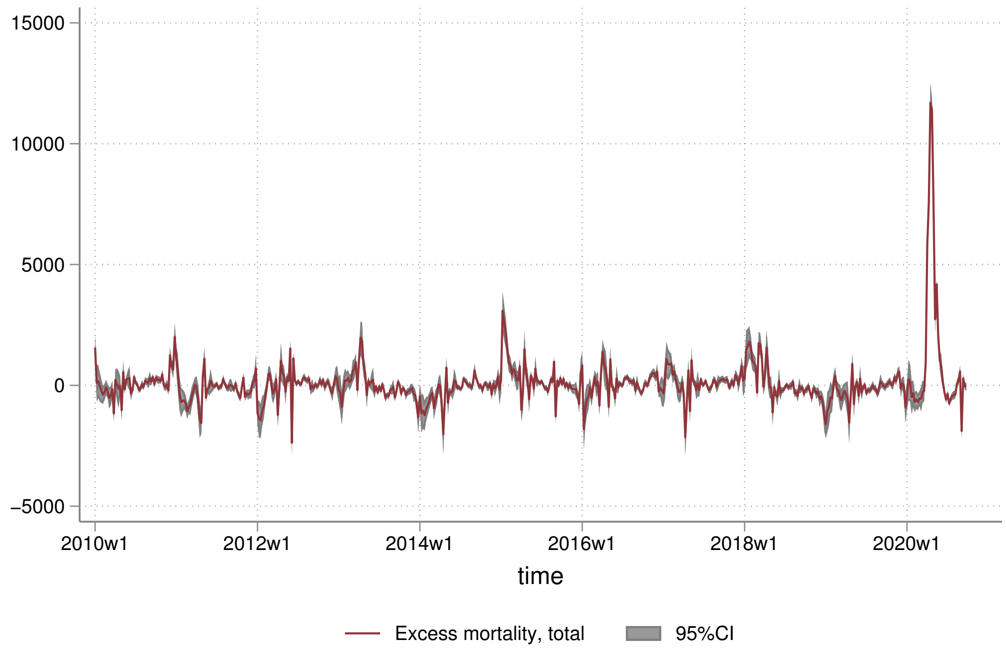

Figure 8: All-cause excess deaths, from 2019 week 1

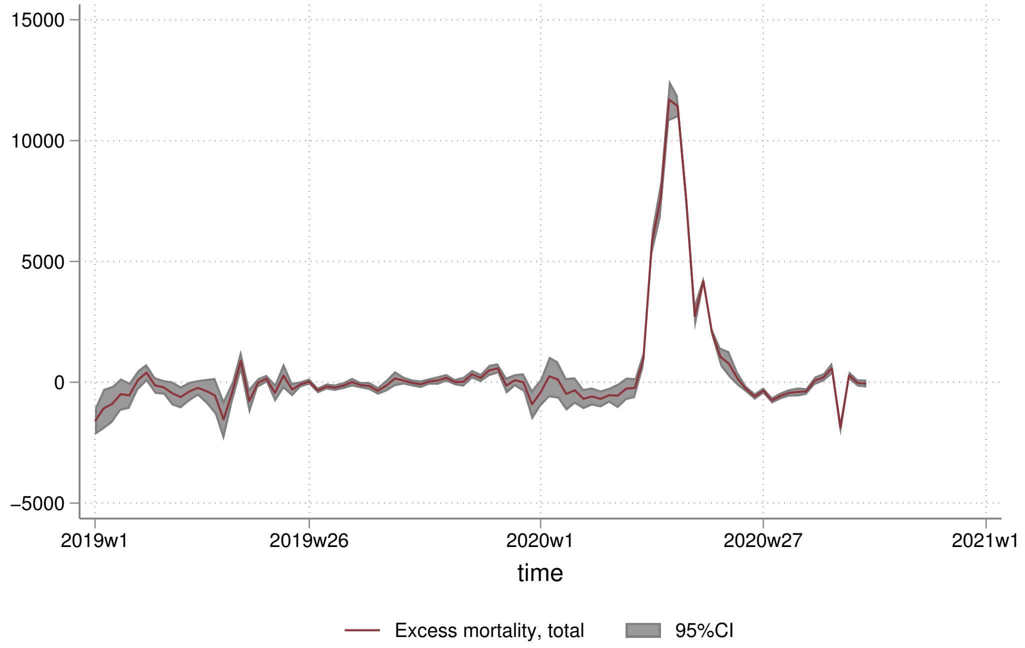

Figure 9: All-cause excess deaths, from 2020 week 1

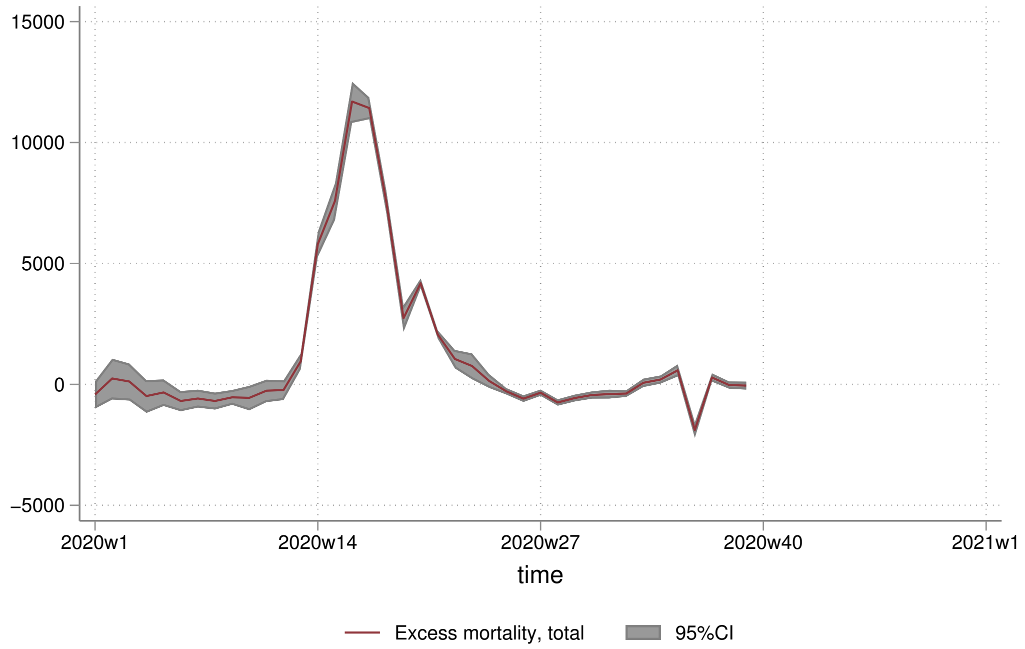

1.4 Excess number of deaths: all causes minus COVID19 deaths

Excluding deaths where COVID19 was mentioned in the death certificate (ICD10 U07.1 and U07.2). A condition mentioned in the death certificate may be the main reason or a contributory reason to the cause of death.

Figure 10: All-cause excess deaths minus COVID19, from 2010 week 1

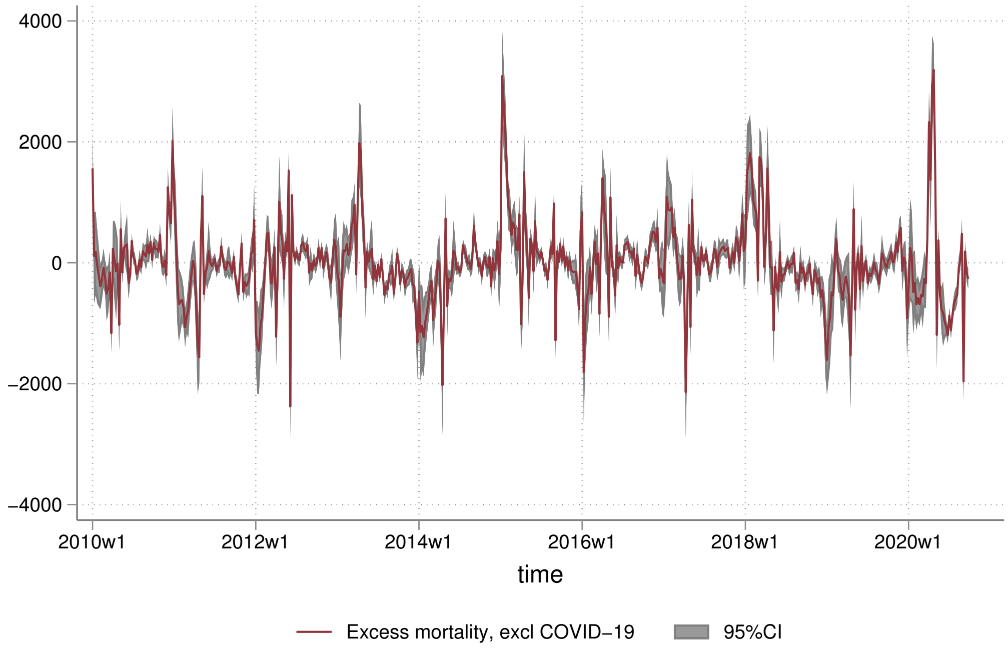

Figure 11: All-cause excess deaths minus COVID19, from 2019 week 1

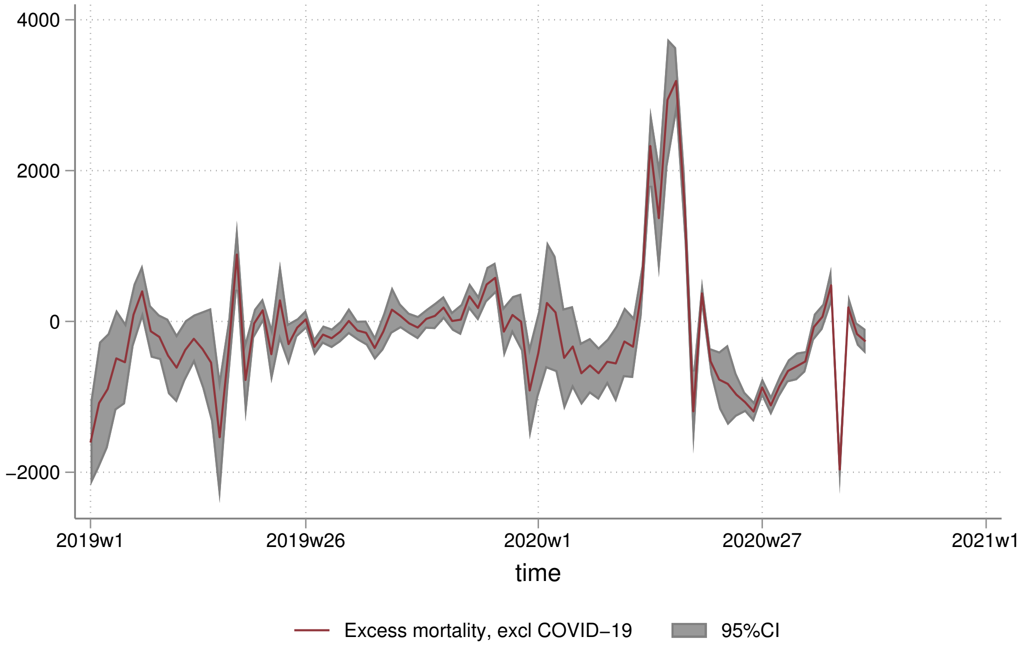

Figure 12: All-cause excess deaths minus COVID19, from 2020 week 1

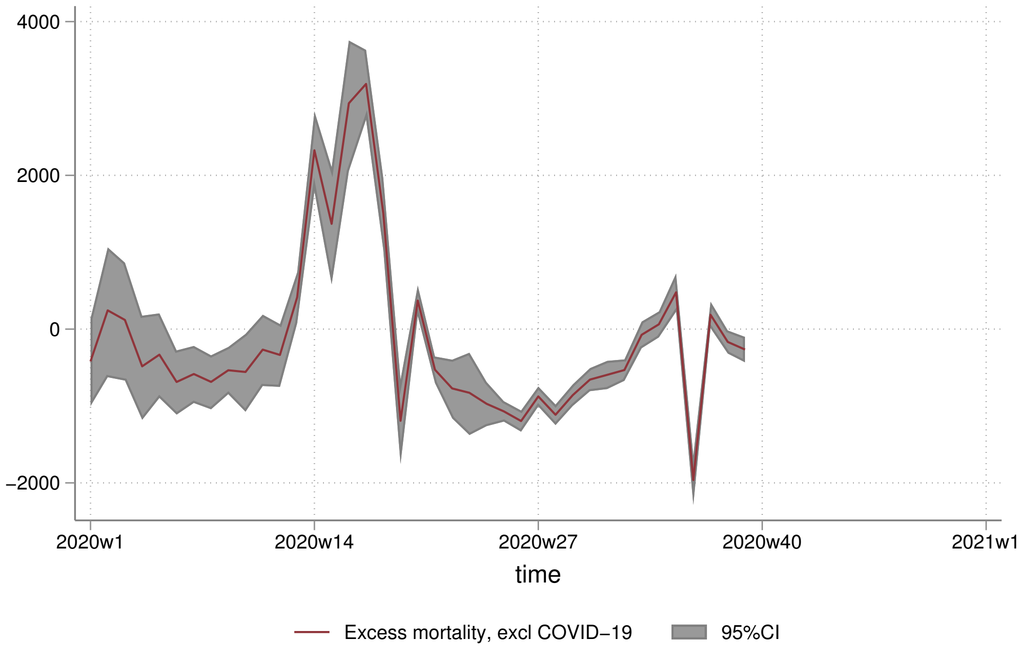

1.5 Excess number of deaths: all causes minus respiratory deaths

Excluding deaths where the underlying cause of death has been recorded as respiratory (ICD-10 codes J00-J99), and excluded deaths at age under 28 days. Underlying cause of death, as reported in England and Wales, is compatible with WHO recommendations using ICD rules (with small changes in 2011 and 2014), and is obtained from the death certificate. The underlying cause of death is defined by the WHO as the disease or injury that initiated the series of events that led directly to death, or the circumstances of the accident or violence that produced the fatal injury (ONS 2020).

Figure 13: All-cause excess deaths minus COVID19, from 2010 week 1

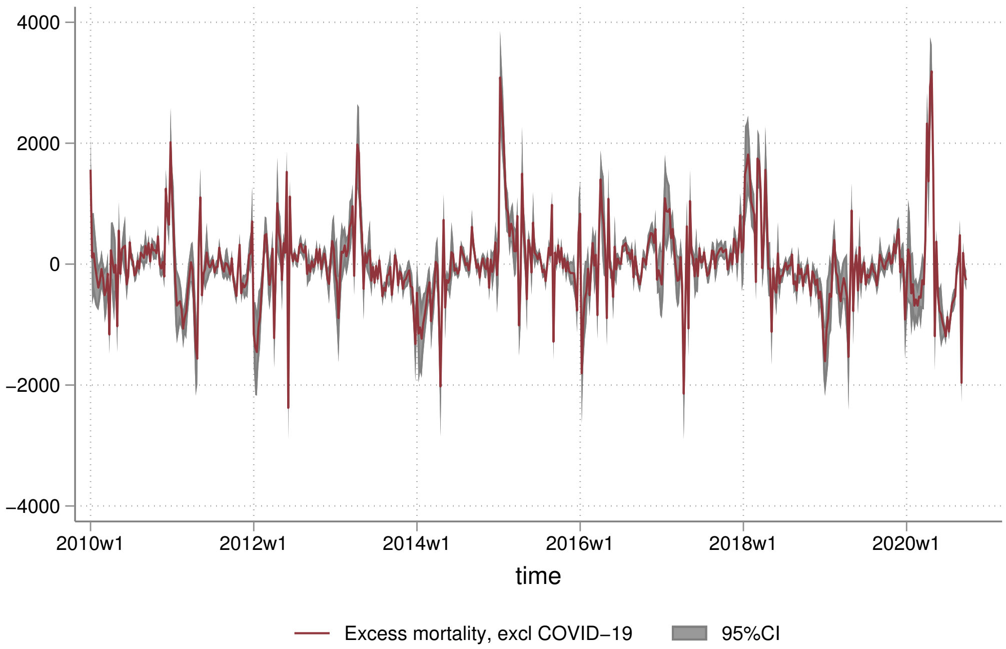

Figure 14: All-cause excess deaths minus COVID19, from 2019 week 1

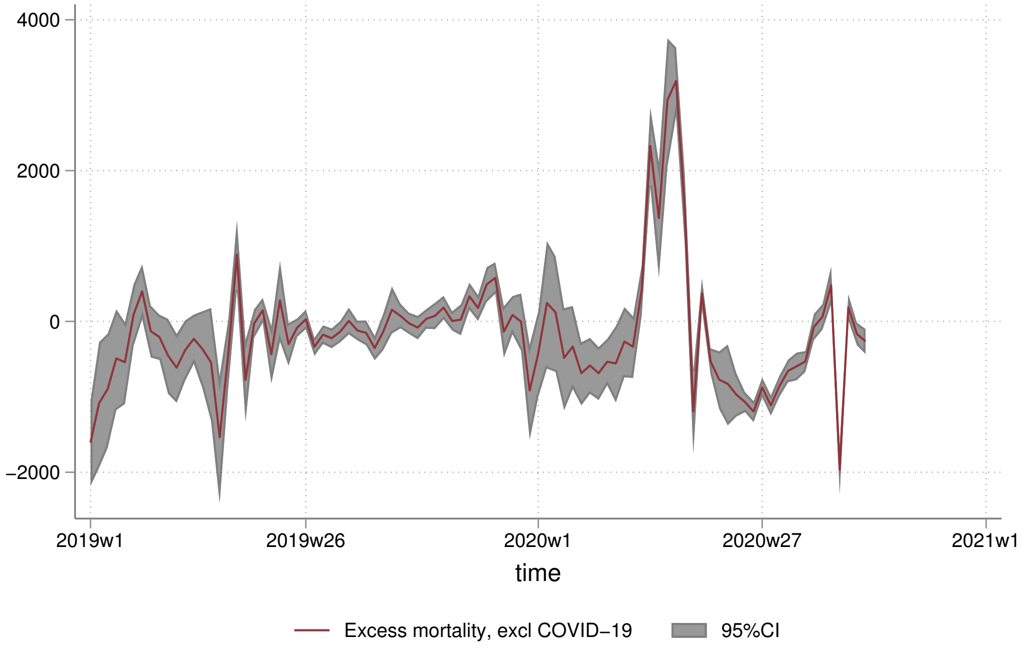

Figure 15: All-cause excess deaths minus COVID19, from 2020 week 1

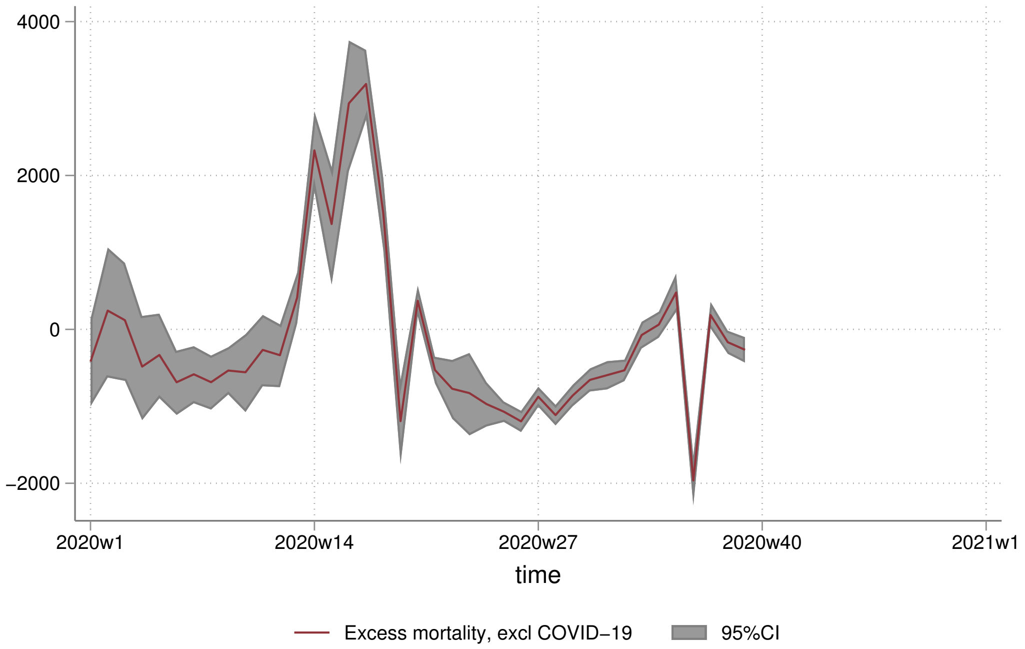

2 Regional

2.1 Time trends

Figure 16: Regional mortality time trends, from 2010 week 1

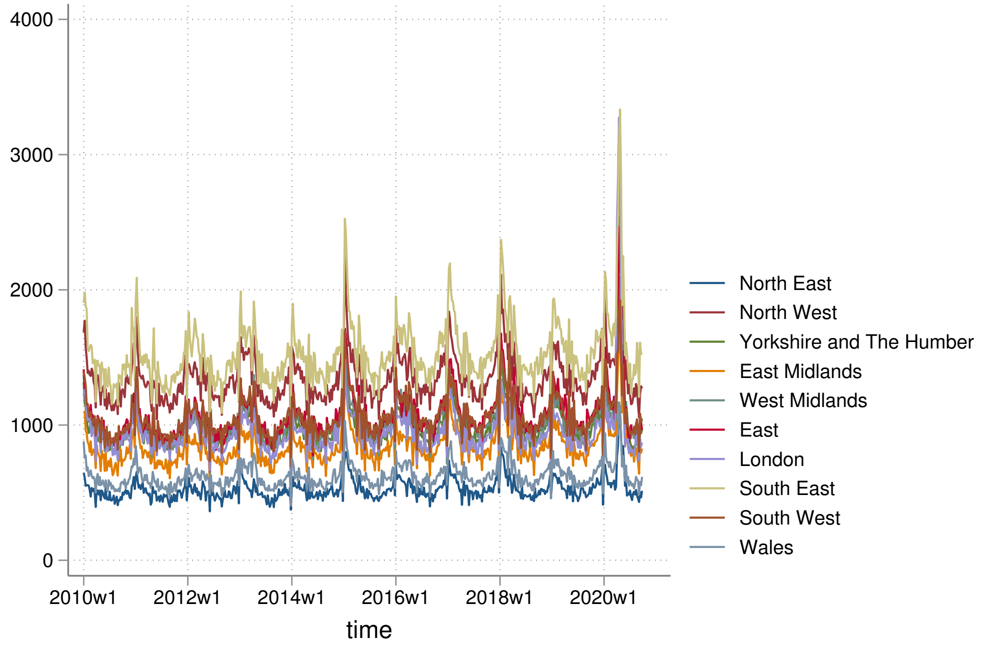

Figure 17: Regional mortality time trends, from 2019 week 1

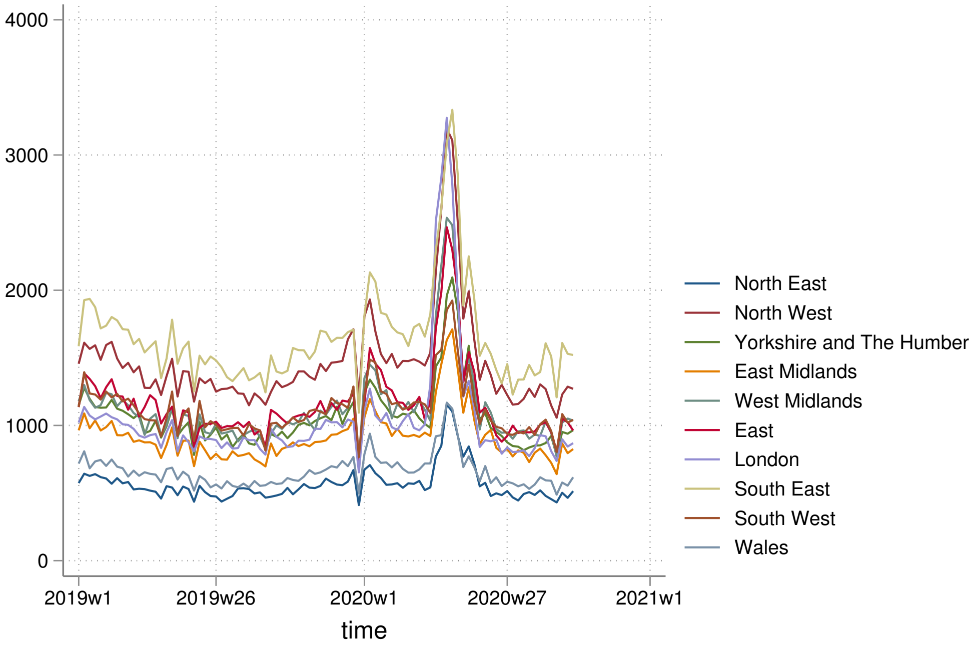

Figure 18: Regional mortality time trends, from 2020 week 1

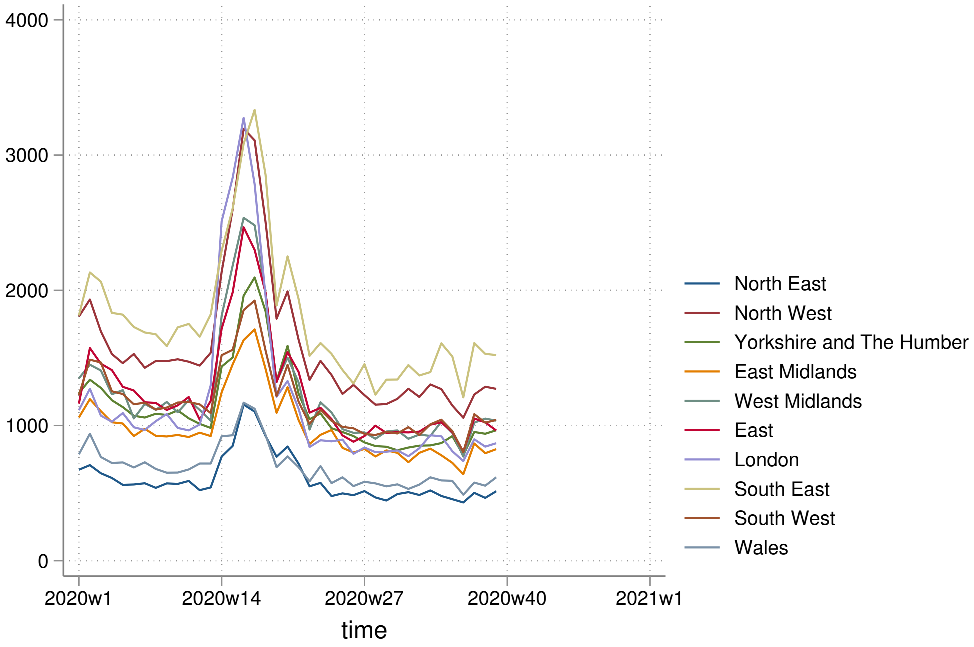

2.2 North East

Figure 19: NE Mortality time trend and model, from 2010 week 1

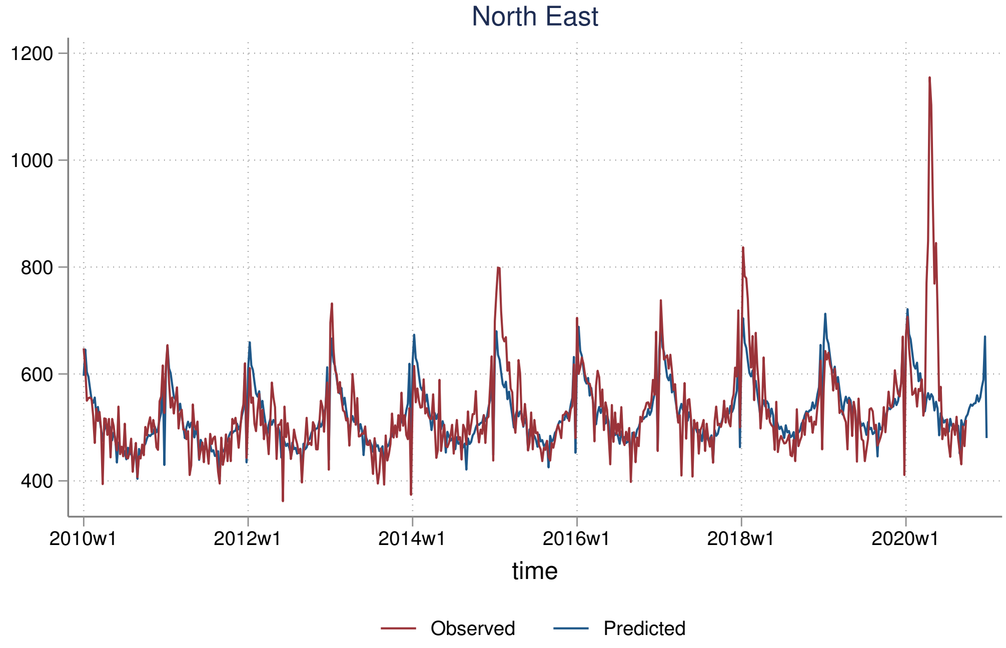

Figure 20: NE Mortality time trend and model, from 2019 week 1

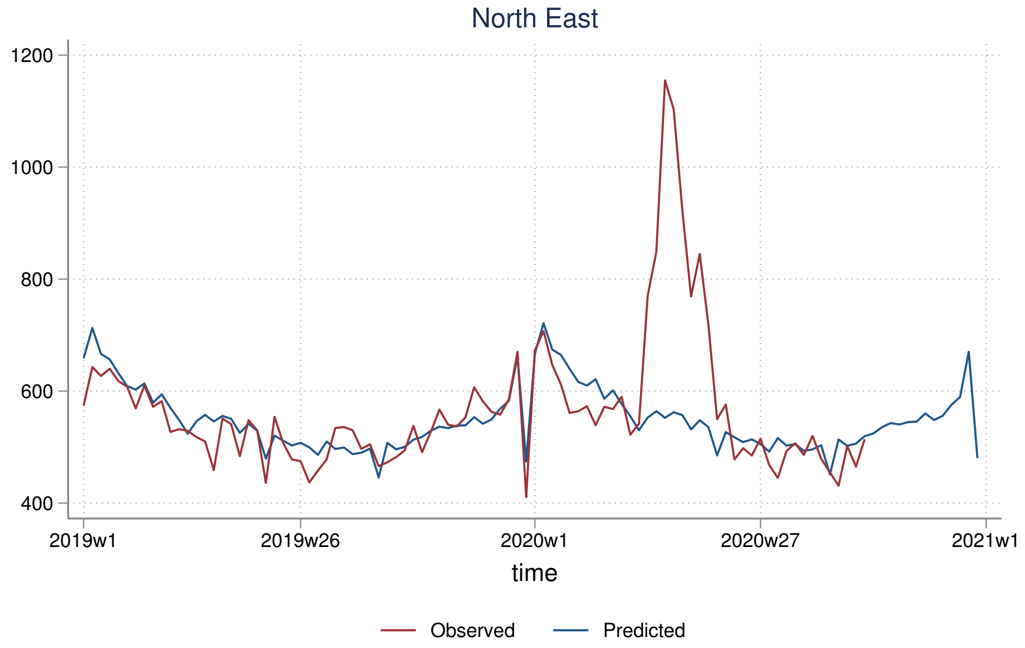

Figure 21: NE Mortality time trend and model, from 2020 week 1

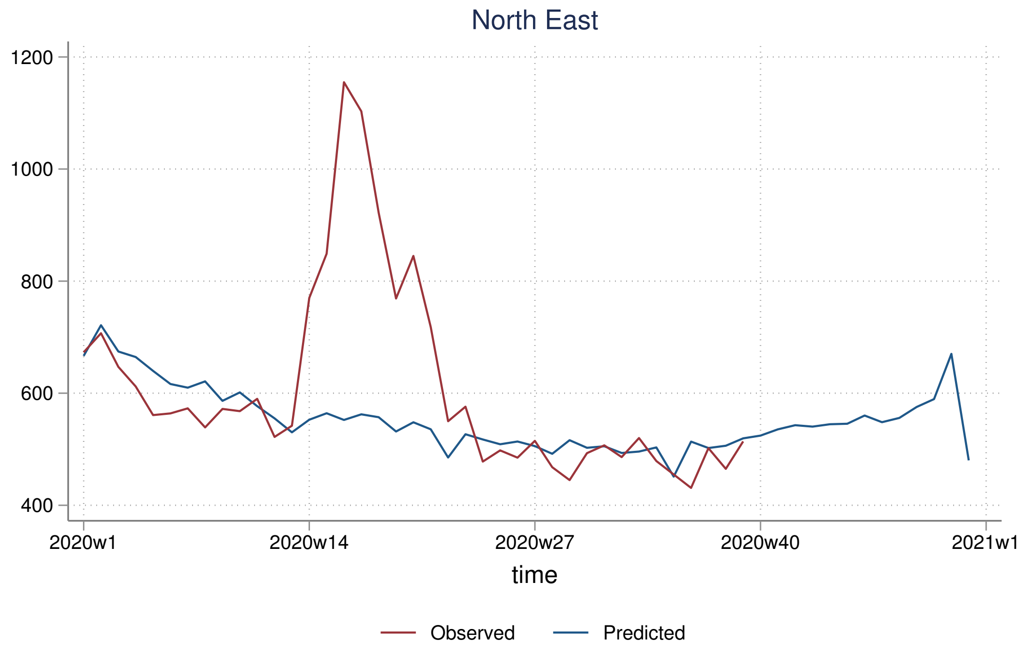

Figure 22: NE all-cause excess deaths, from 2010 week 1

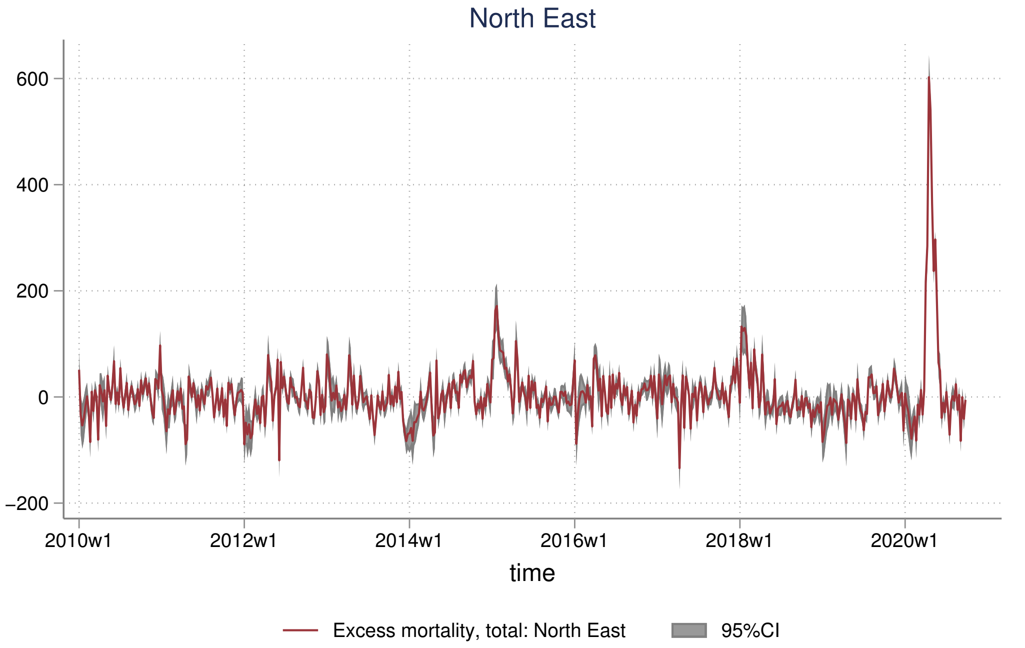

Figure 23: NE all-cause excess deaths, from 2019 week 1

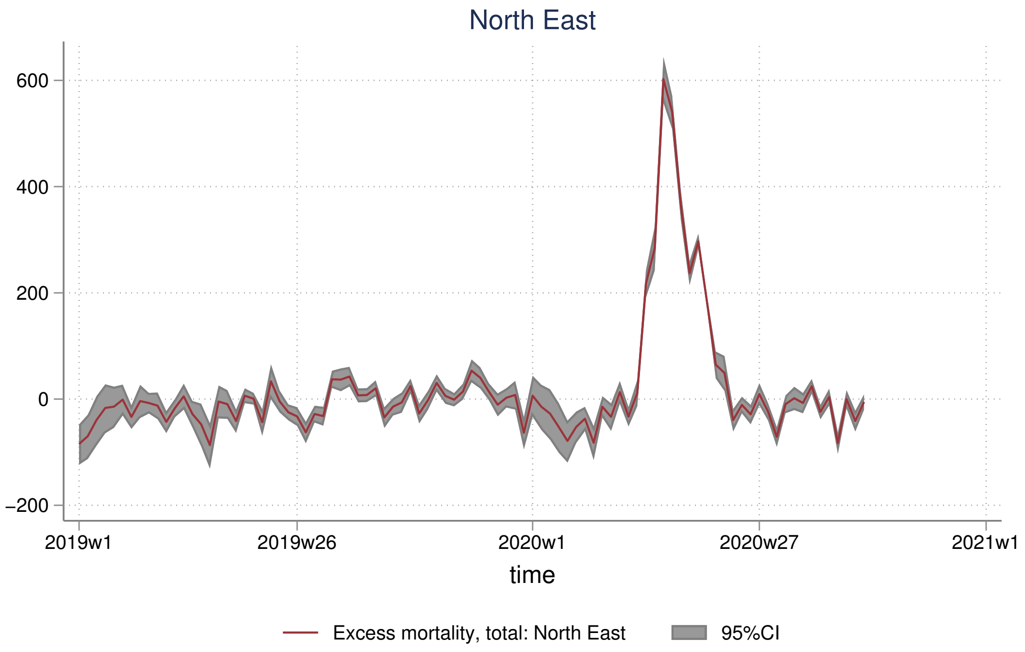

Figure 24: NE all-cause excess deaths, from 2020 week 1

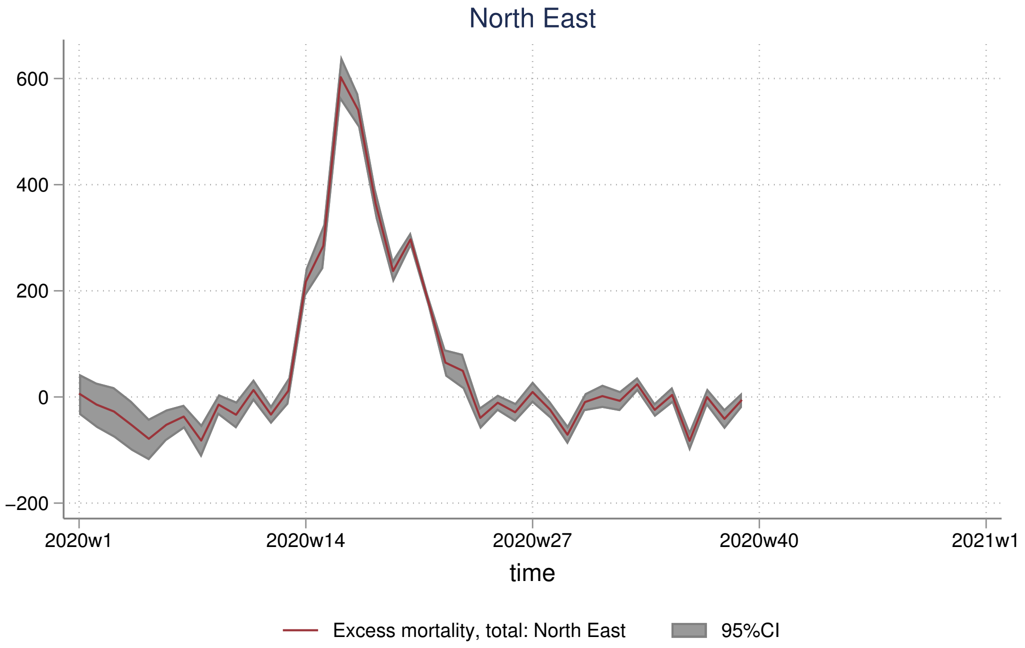

Figure 25: NE all-cause excess deaths minus COVID19, from 2010 week 1

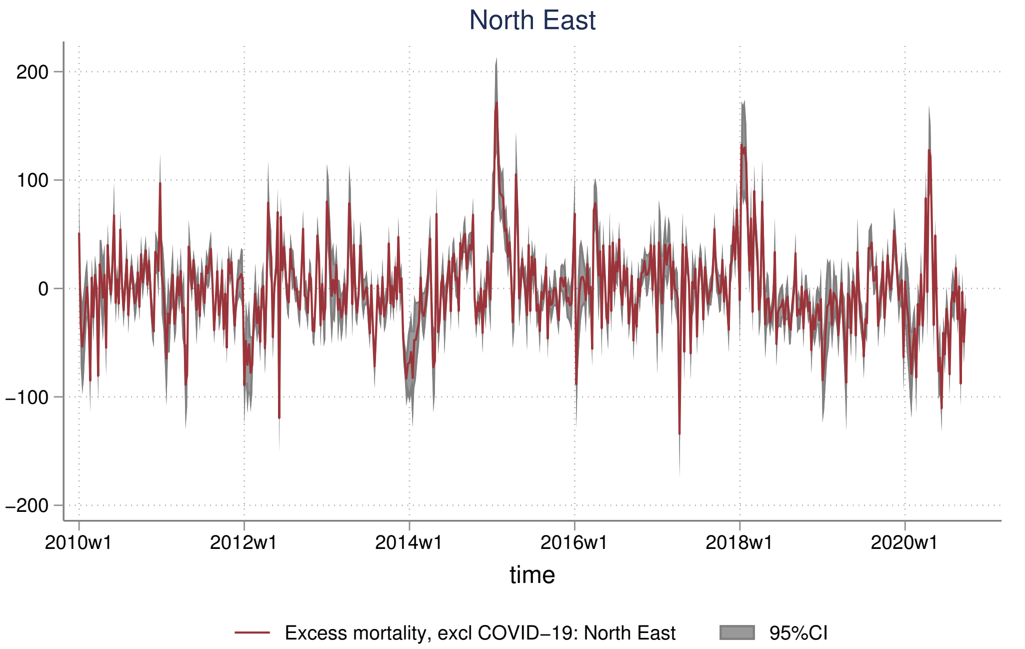

Figure 26: NE all-cause excess deaths minus COVID19, from 2019 week 1

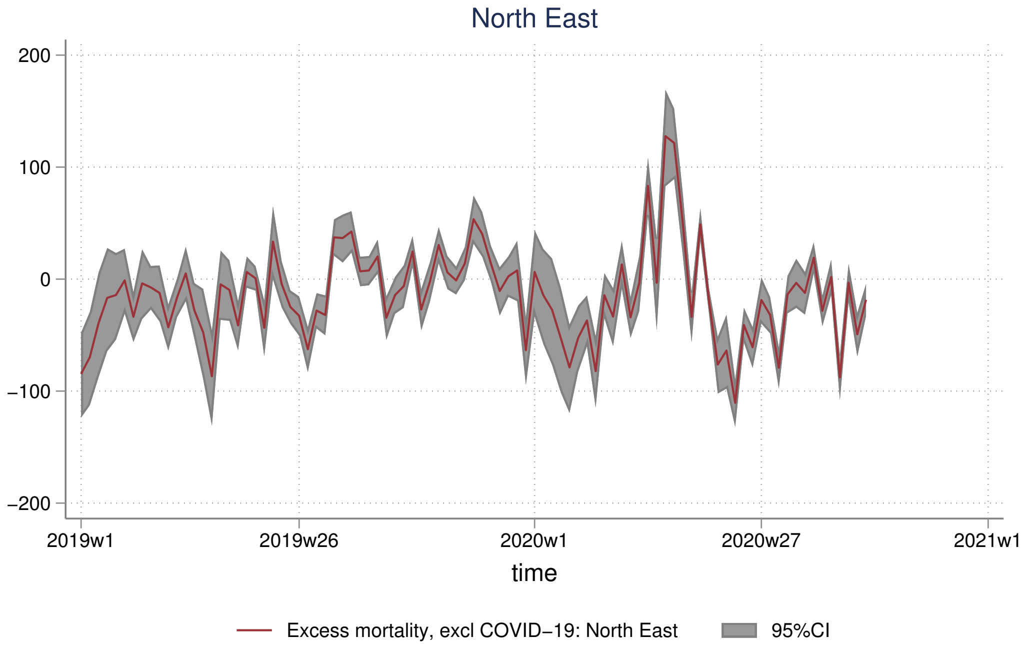

Figure 27: NE all-cause excess deaths minus COVID19, from 2020 week 1

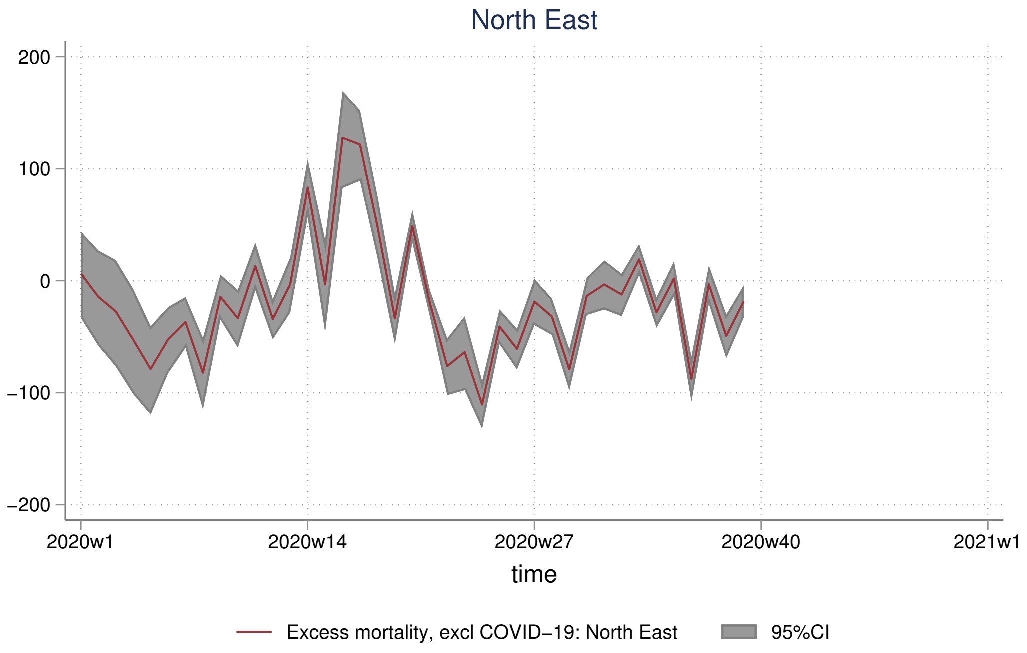

2.3 North West

Figure 28: NW Mortality time trend and model, from 2010 week 1

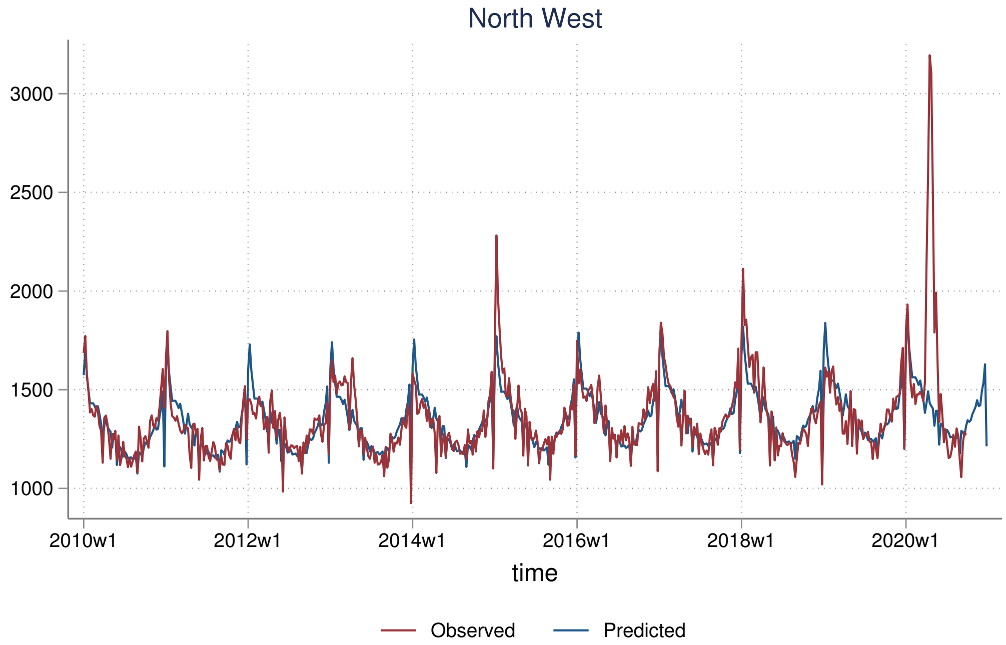

Figure 29: NW Mortality time trend and model, from 2019 week 1

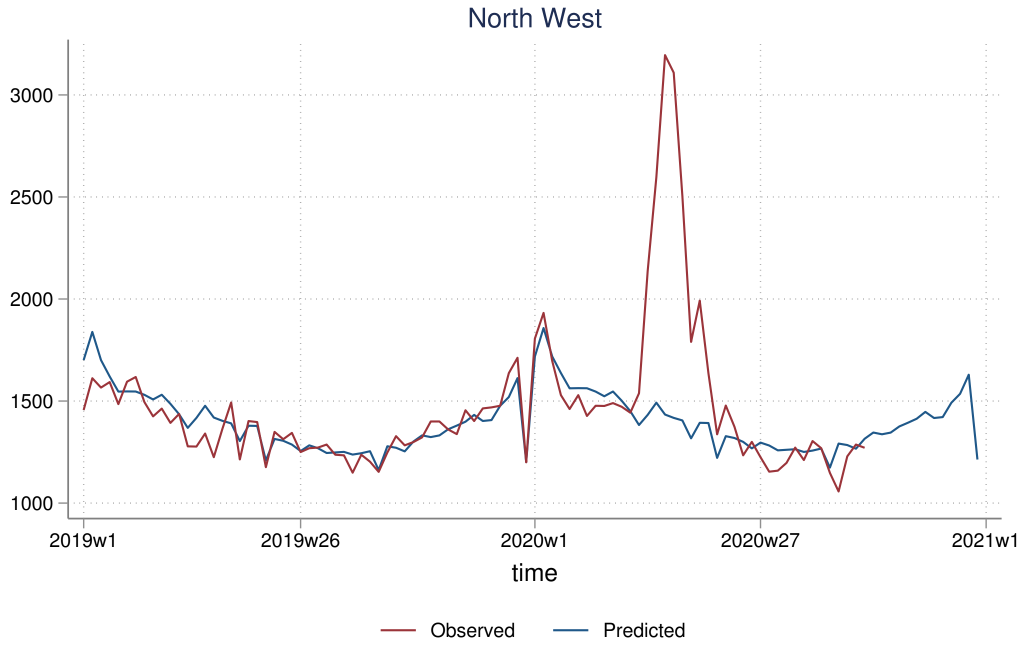

Figure 30: NW Mortality time trend and model, from 2020 week 1

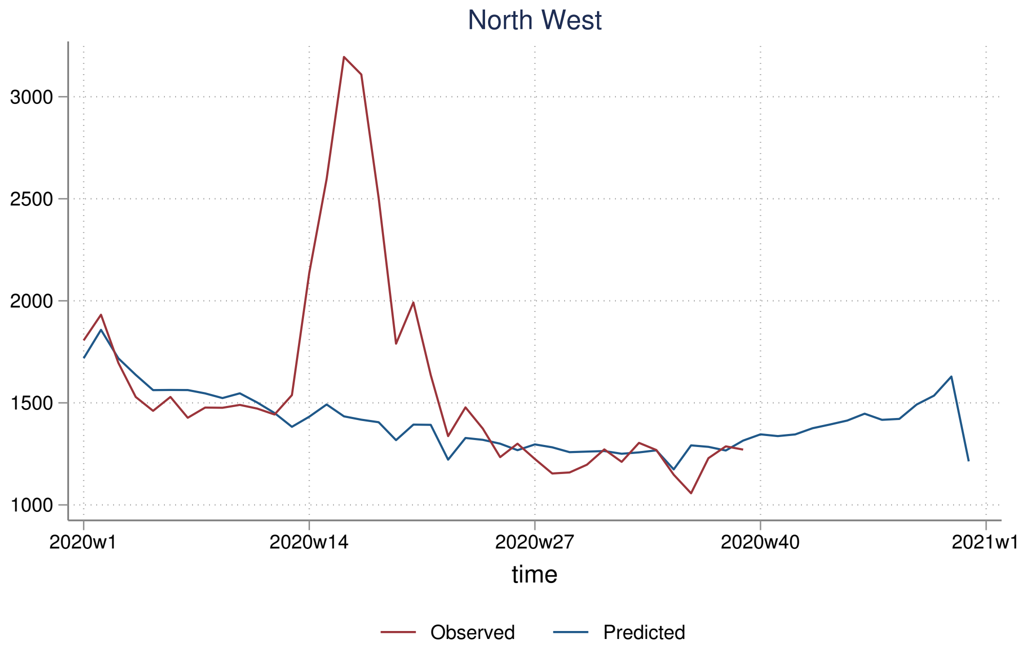

Figure 31: NW all-cause excess deaths, from 2010 week 1

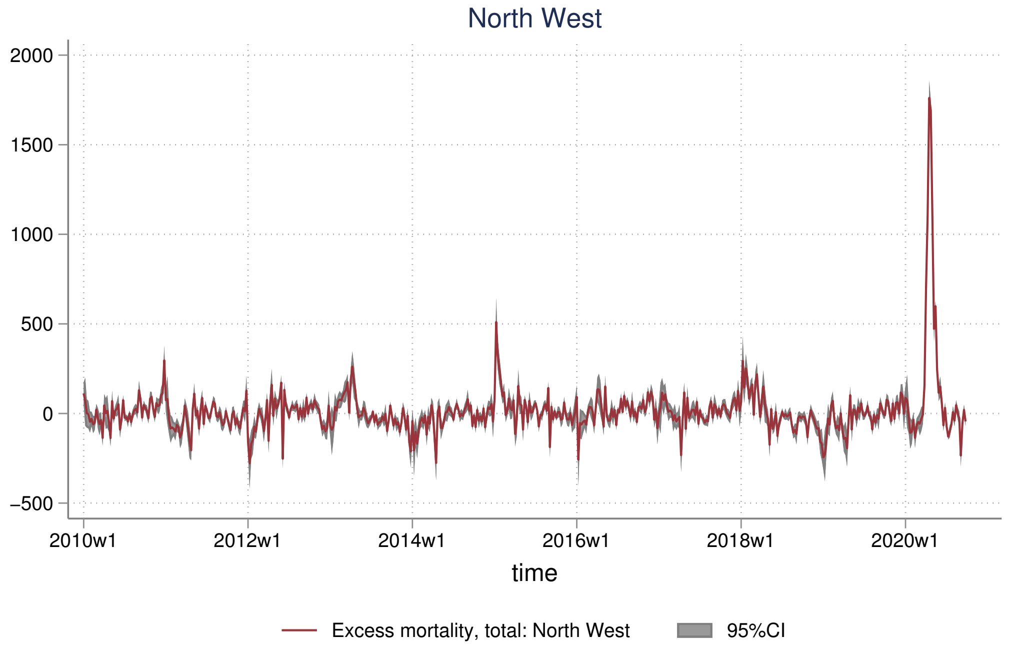

Figure 32: NW all-cause excess deaths, from 2019 week 1

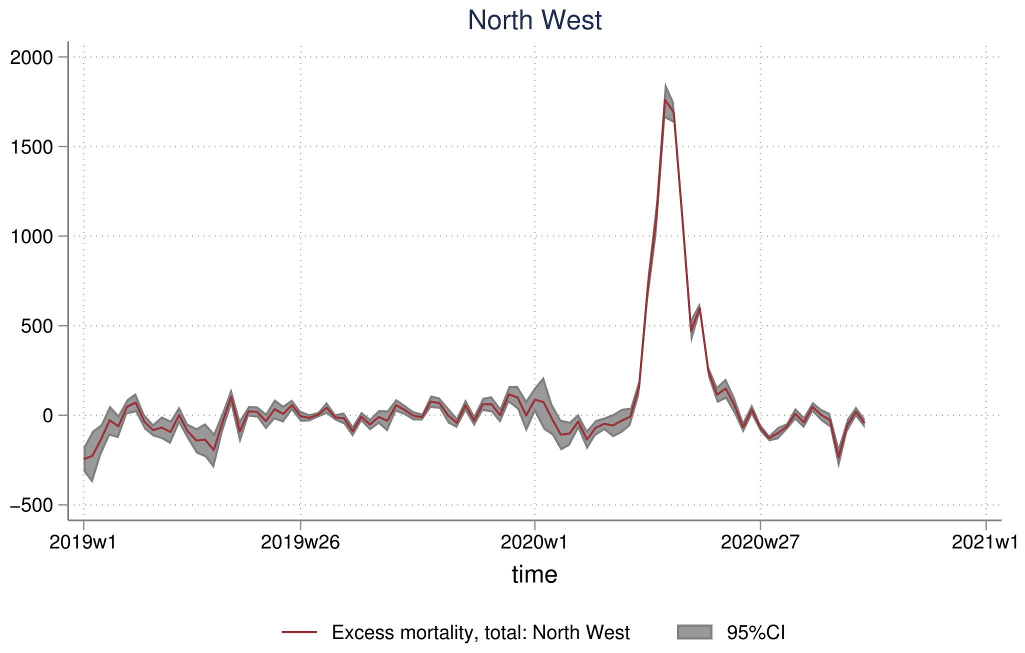

Figure 33: NW all-cause excess deaths, from 2020 week 1

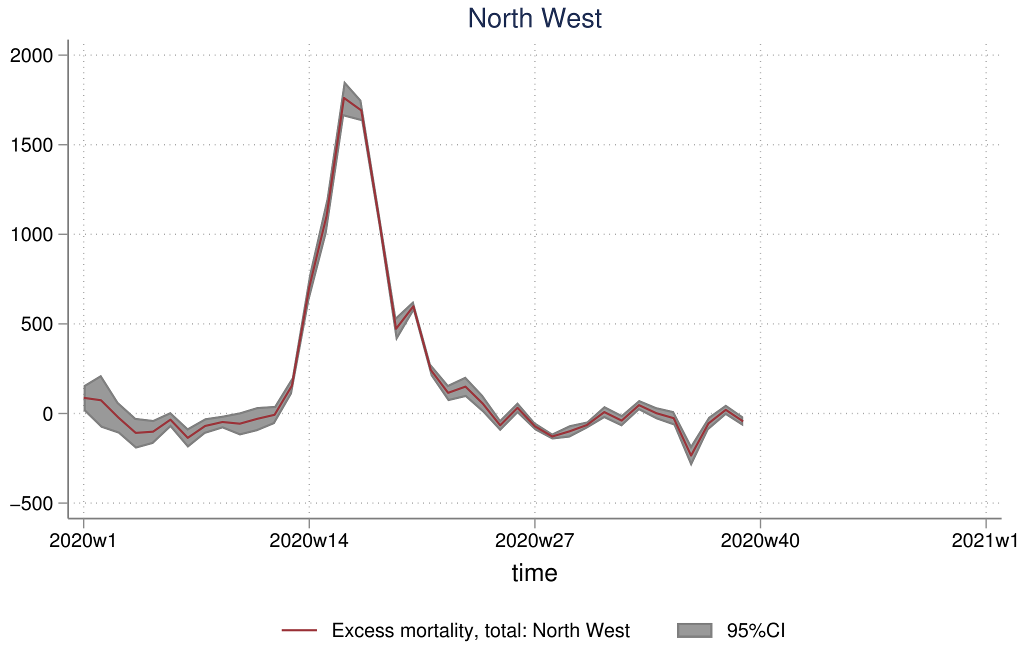

Figure 34: NW all-cause excess deaths minus COVID19, from 2010 week 1

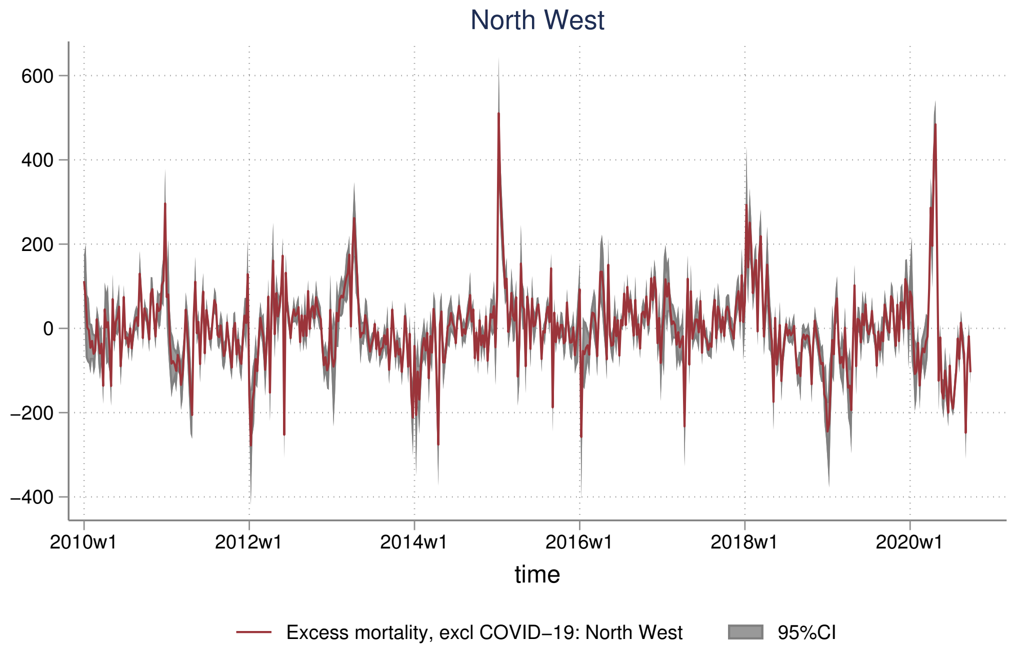

Figure 35: NW all-cause excess deaths minus COVID19, from 2019 week 1

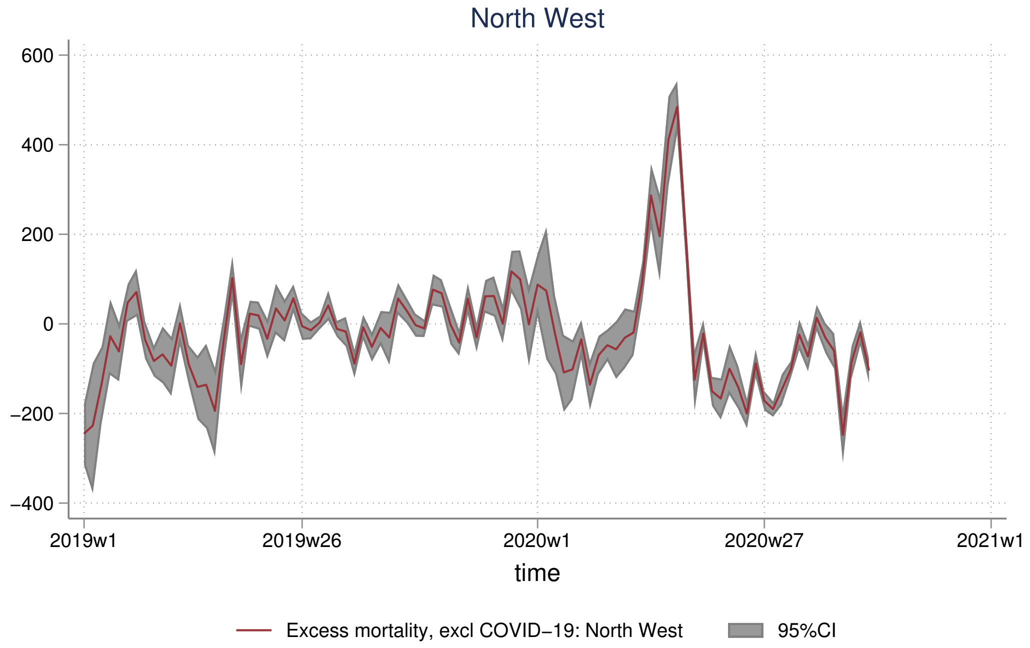

Figure 36: NW all-cause excess deaths minus COVID19, from 2020 week 1

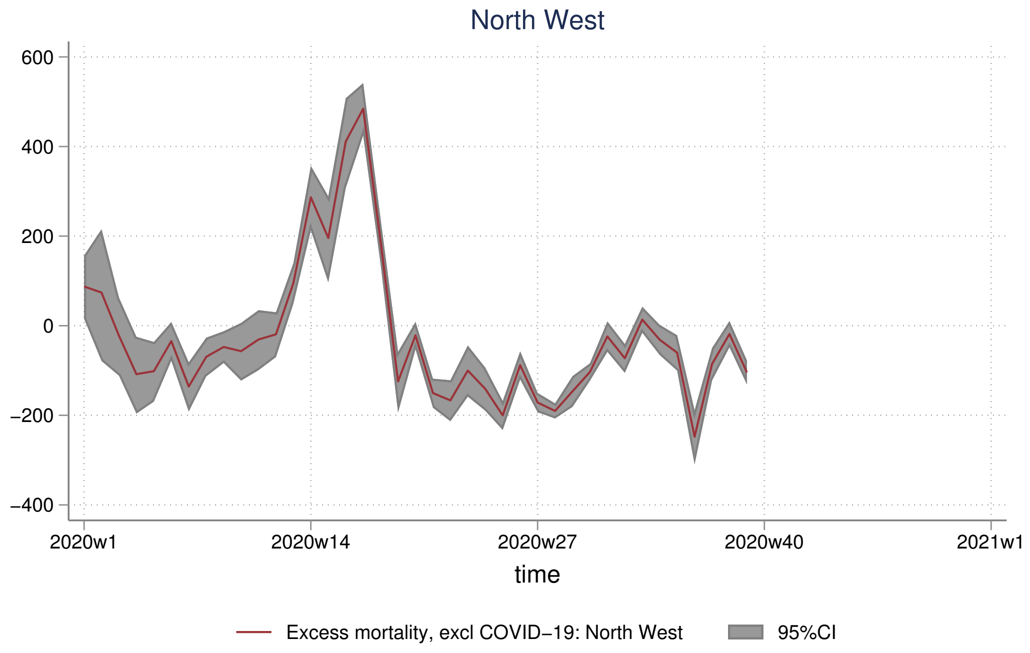

2.4 Yorkshire and the Humber

Figure 37: YatH Mortality time trend and model, from 2010 week 1

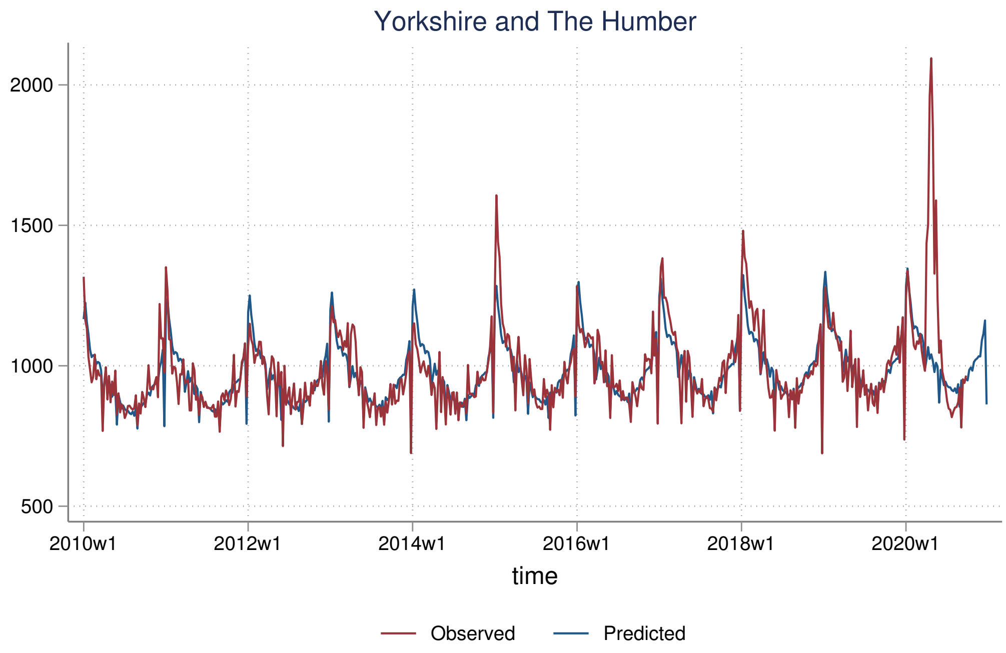

Figure 38: YatH Mortality time trend and model, from 2019 week 1

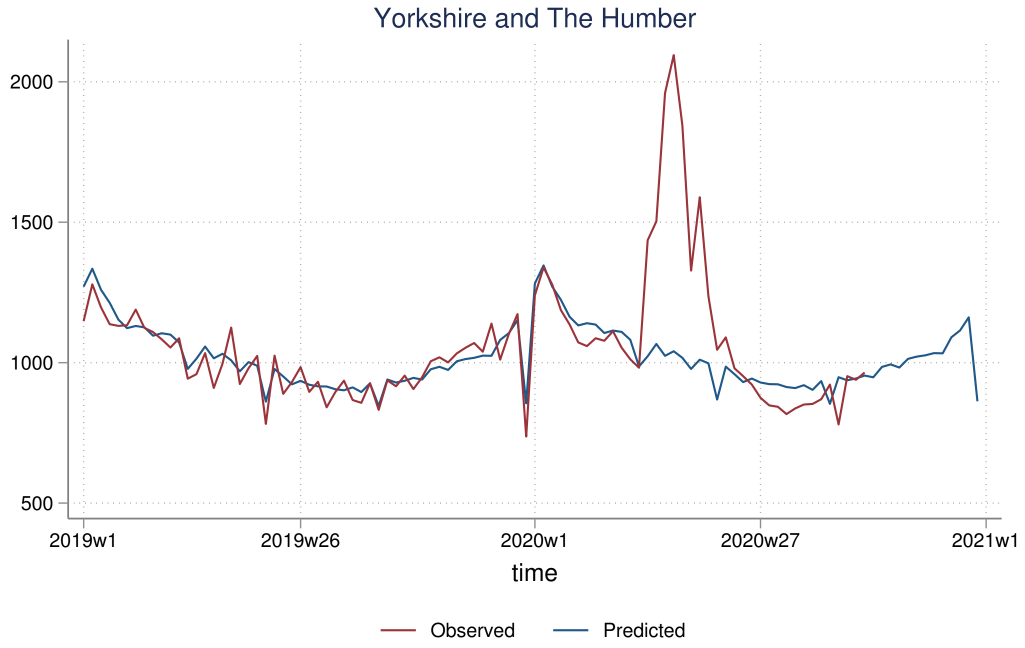

Figure 39: YatH Mortality time trend and model, from 2020 week 1

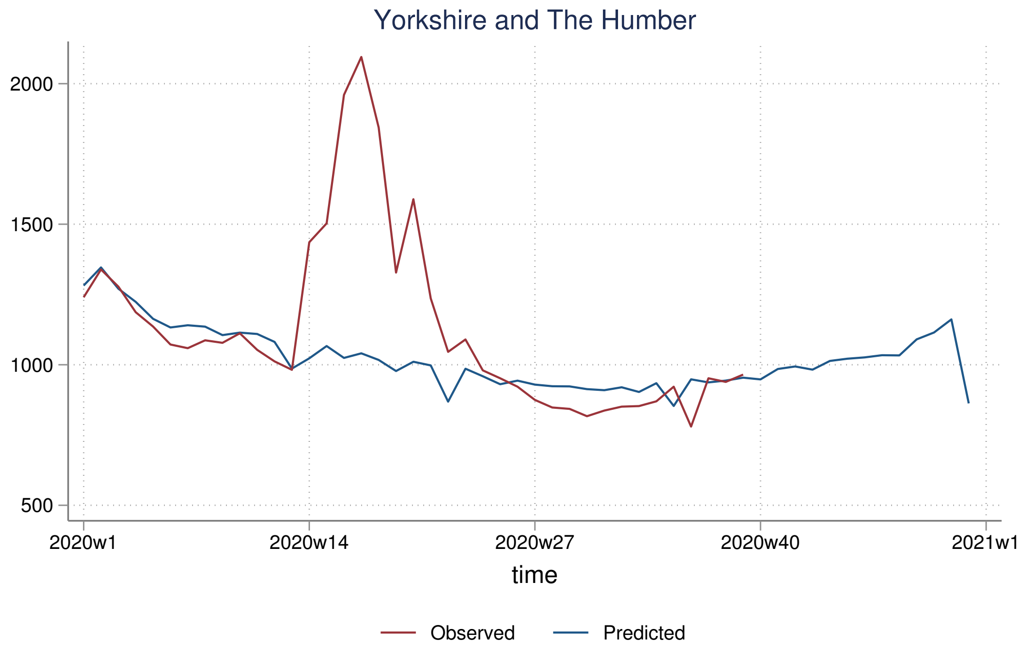

Figure 40: YatH all-cause excess deaths, from 2010 week 1

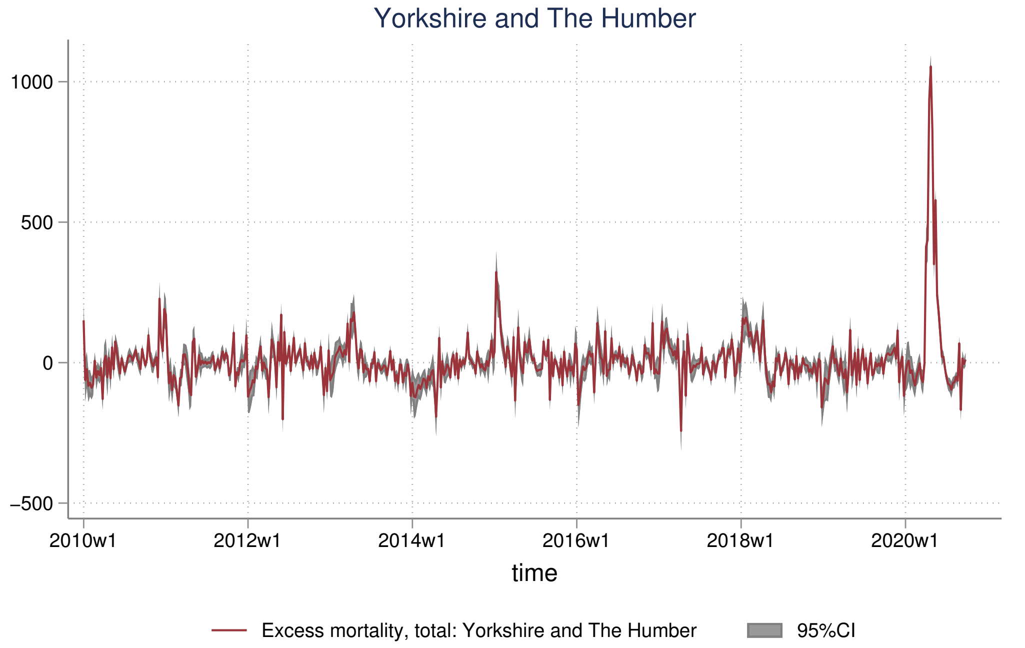

Figure 41: YatH all-cause excess deaths, from 2019 week 1

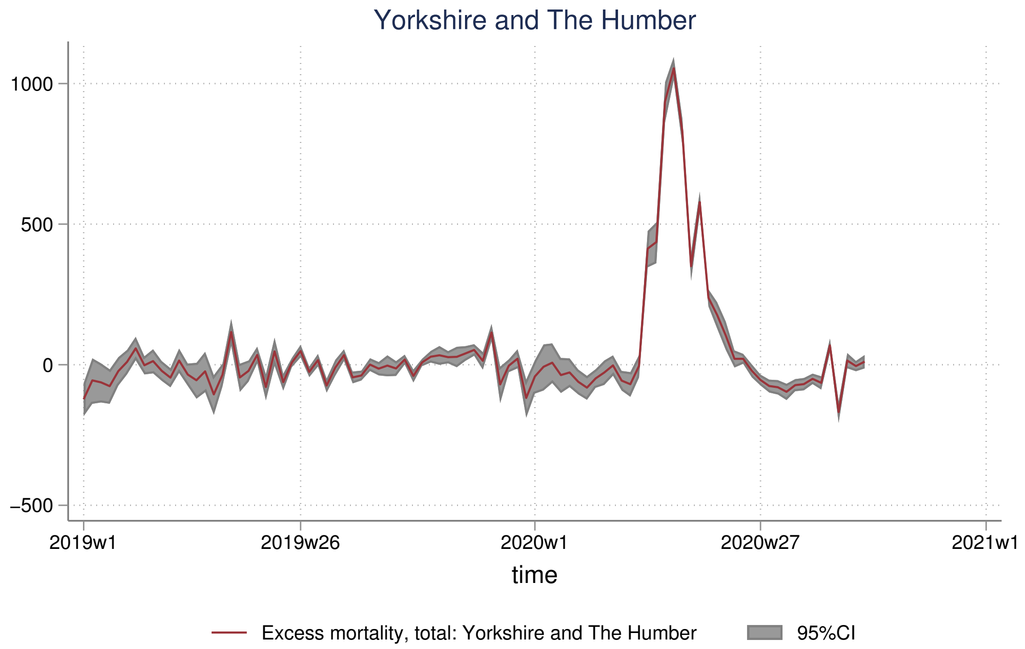

Figure 42: YatH all-cause excess deaths, from 2020 week 1

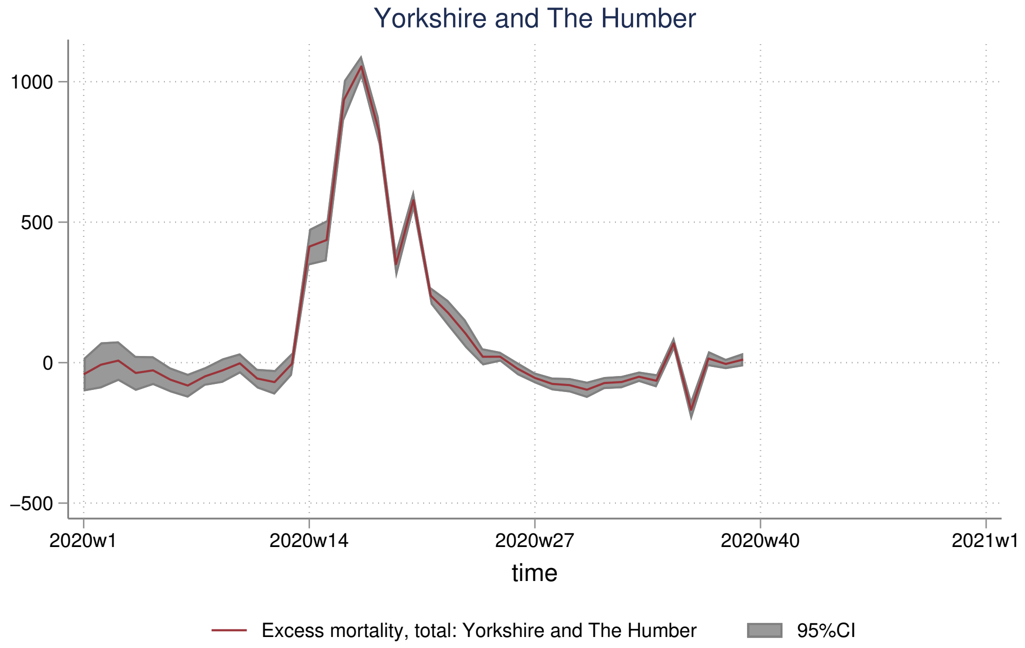

Figure 43: YatH all-cause excess deaths minus COVID19, from 2010 week 1

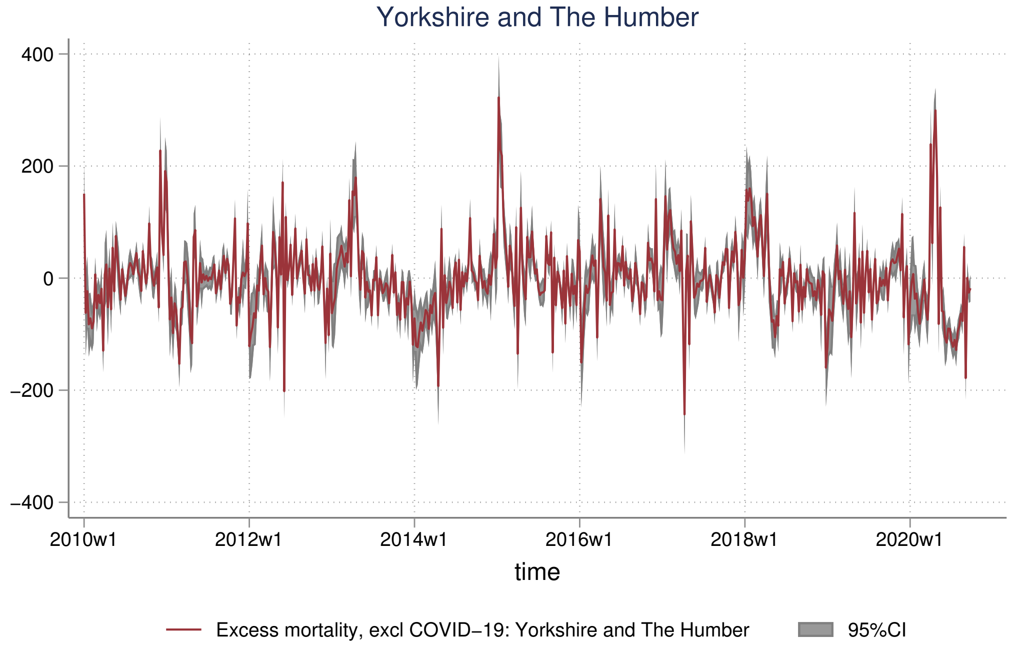

Figure 44: YatH all-cause excess deaths minus COVID19, from 2019 week 1

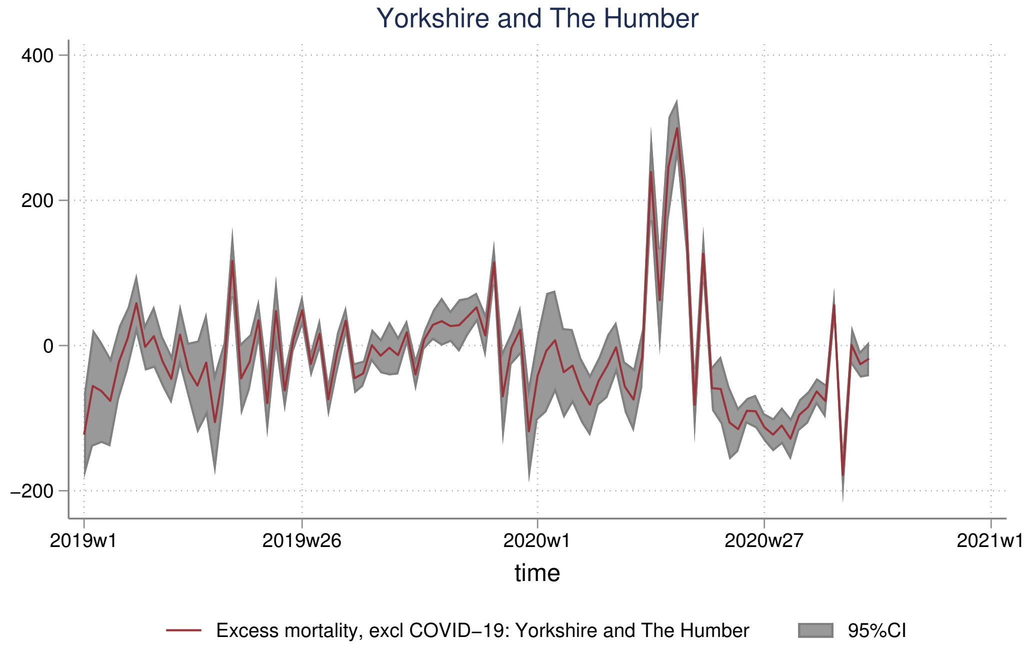

Figure 45: YatH all-cause excess deaths minus COVID19, from 2020 week 1

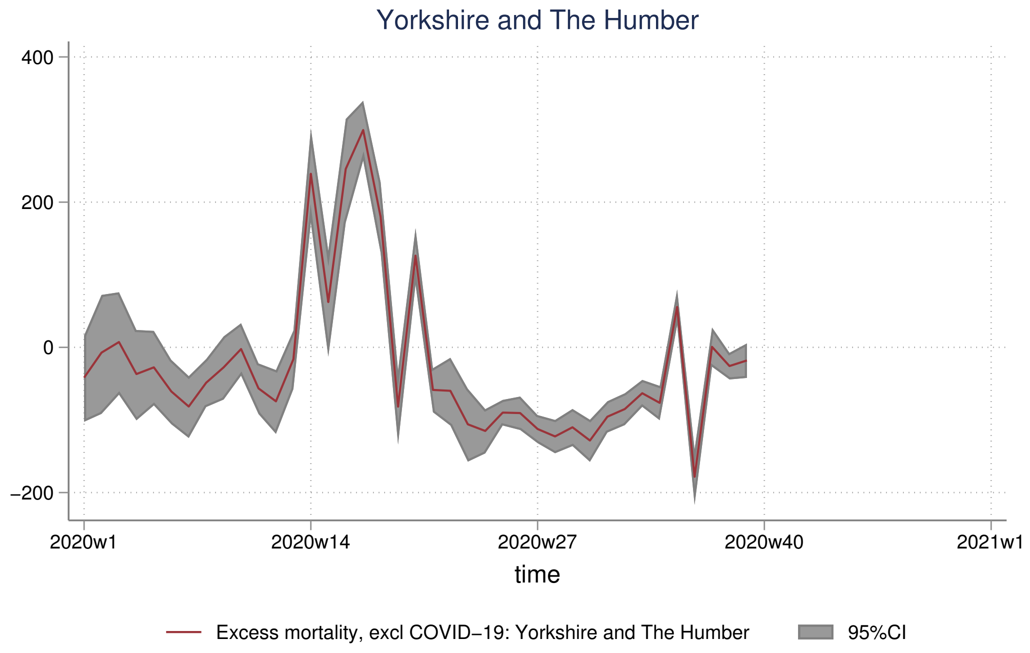

2.5 East Midlands

Figure 46: EMD Mortality time trend and model, from 2010 week 1

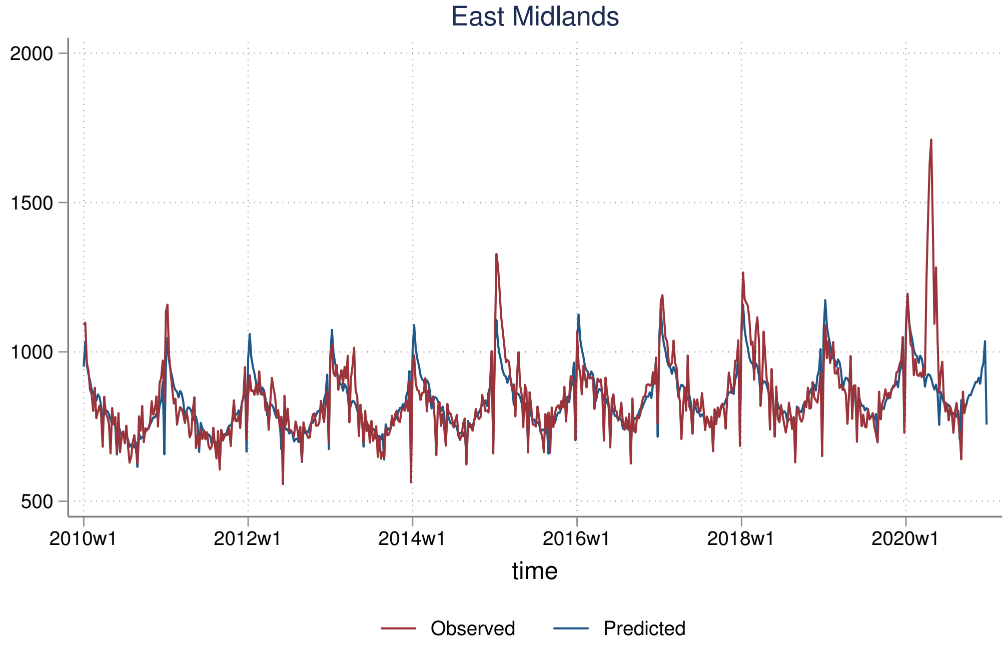

Figure 47: EMD Mortality time trend and model, from 2019 week 1

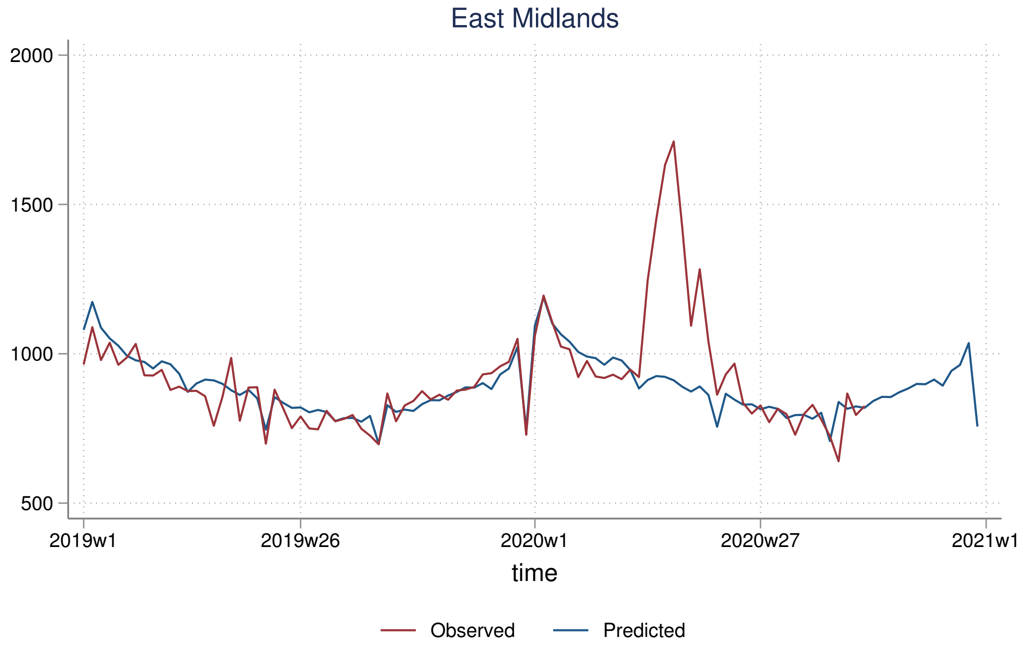

Figure 48: EMD Mortality time trend and model, from 2020 week 1

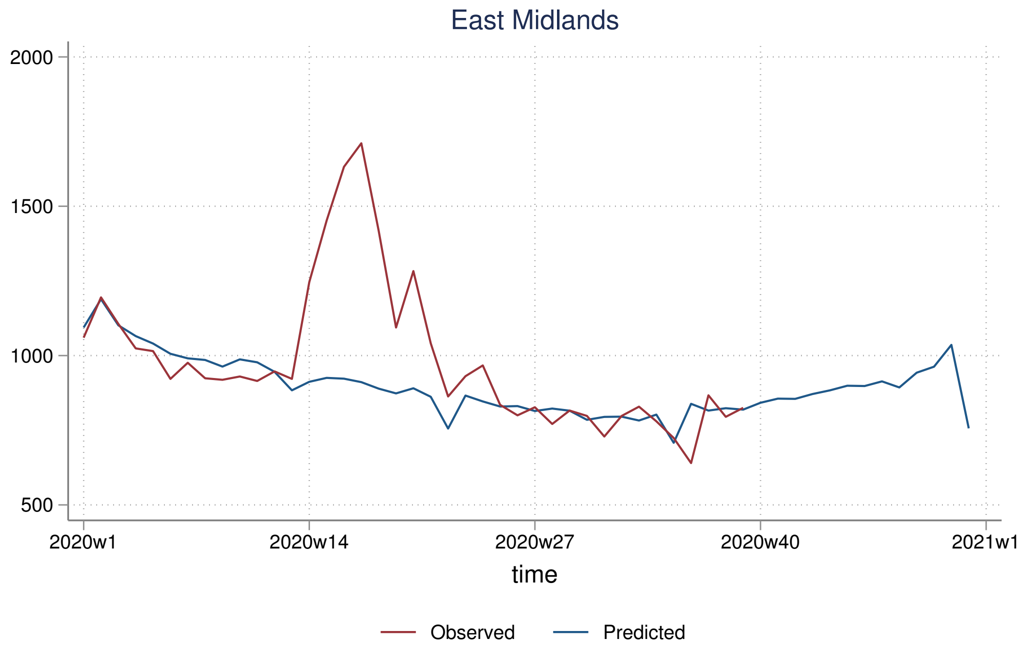

Figure 49: EMD all-cause excess deaths, from 2010 week 1

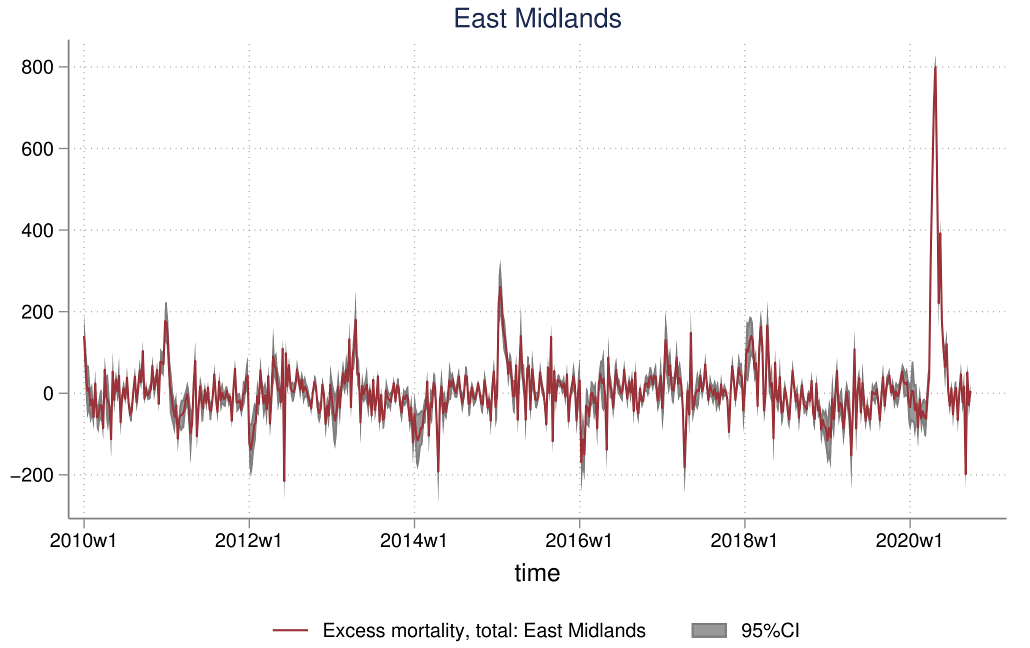

Figure 50: EMD all-cause excess deaths, from 2019 week 1

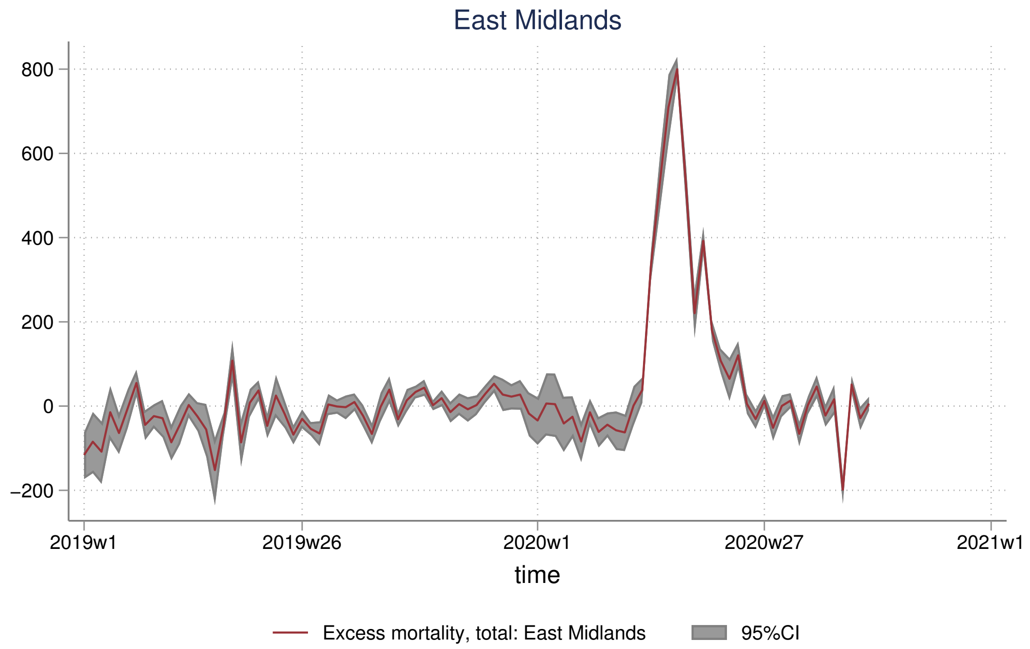

Figure 51: EMD all-cause excess deaths, from 2020 week 1

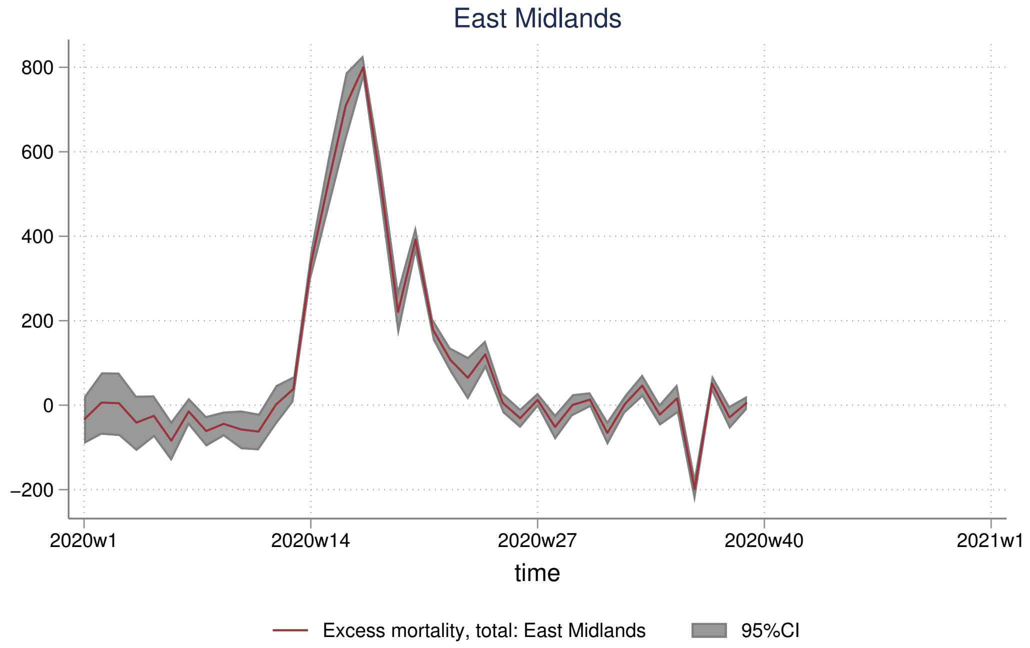

Figure 52: EMD all-cause excess deaths minus COVID19, from 2010 week 1

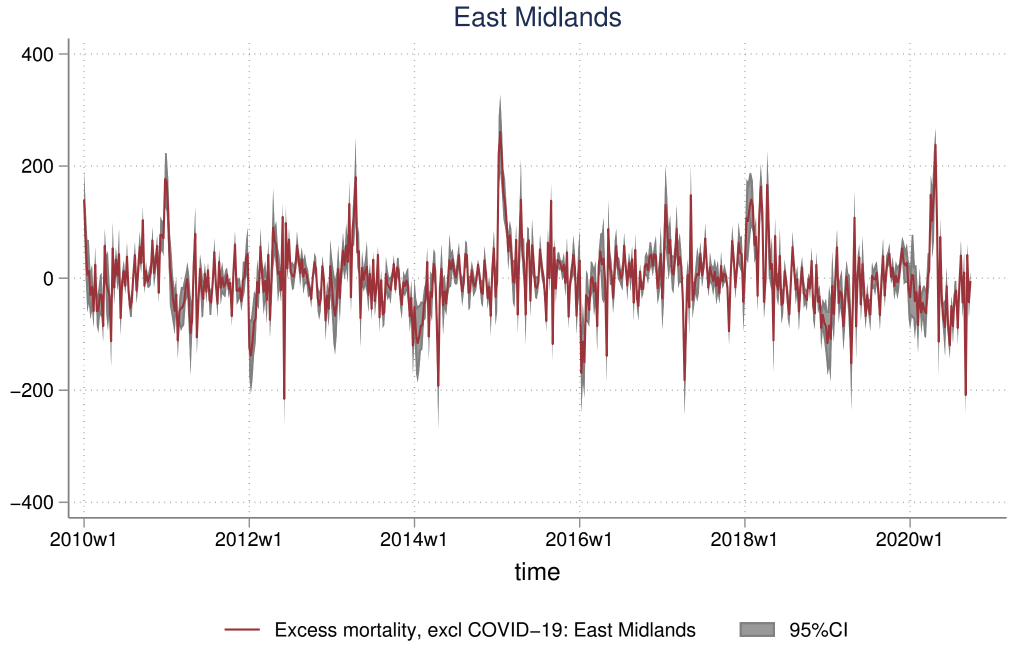

Figure 53: EMD all-cause excess deaths minus COVID19, from 2019 week 1

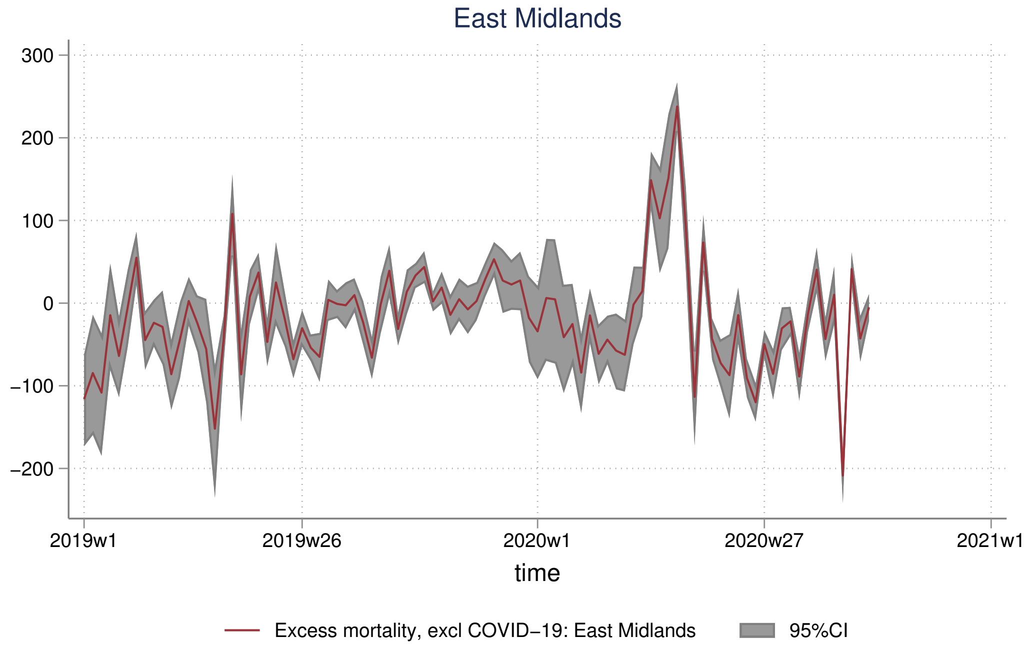

Figure 54: EMD all-cause excess deaths minus COVID19, from 2020 week 1

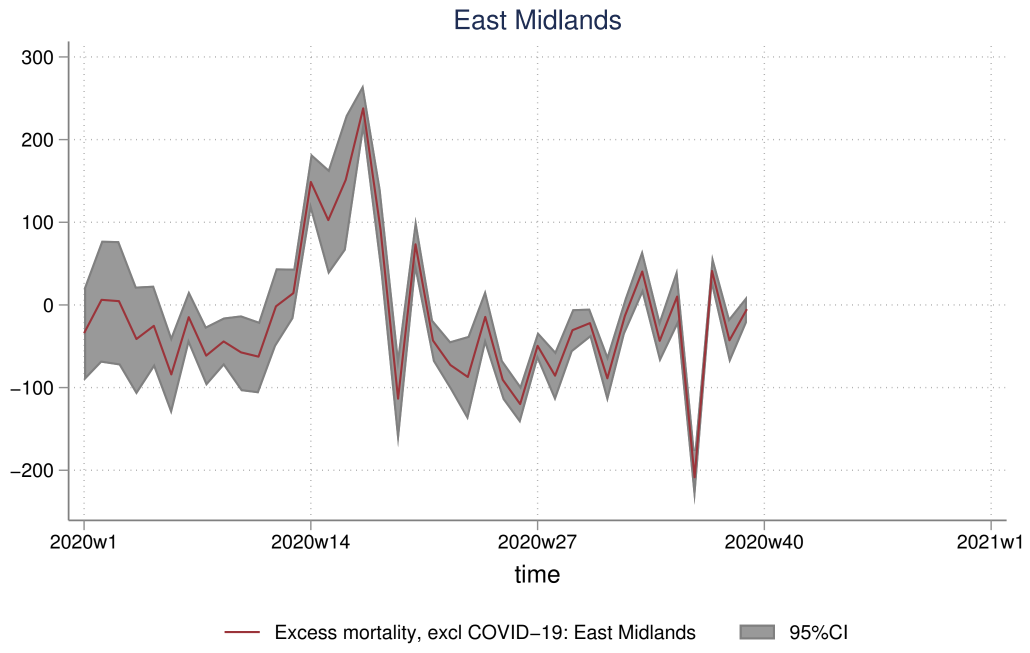

2.6 West Midlands

Figure 55: WMD Mortality time trend and model, from 2010 week 1

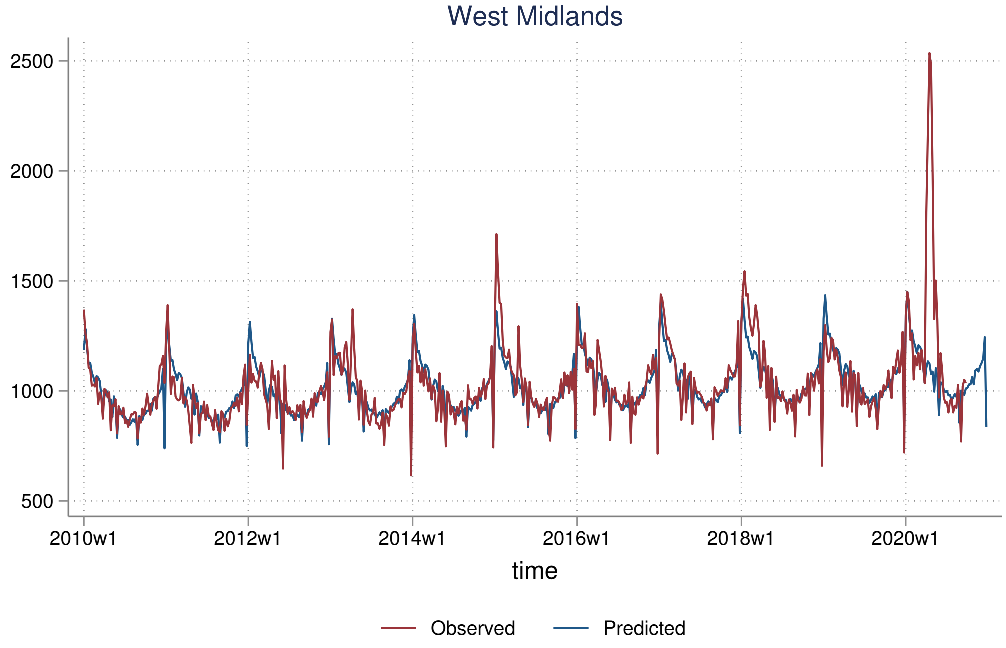

Figure 56: WMD Mortality time trend and model, from 2019 week 1

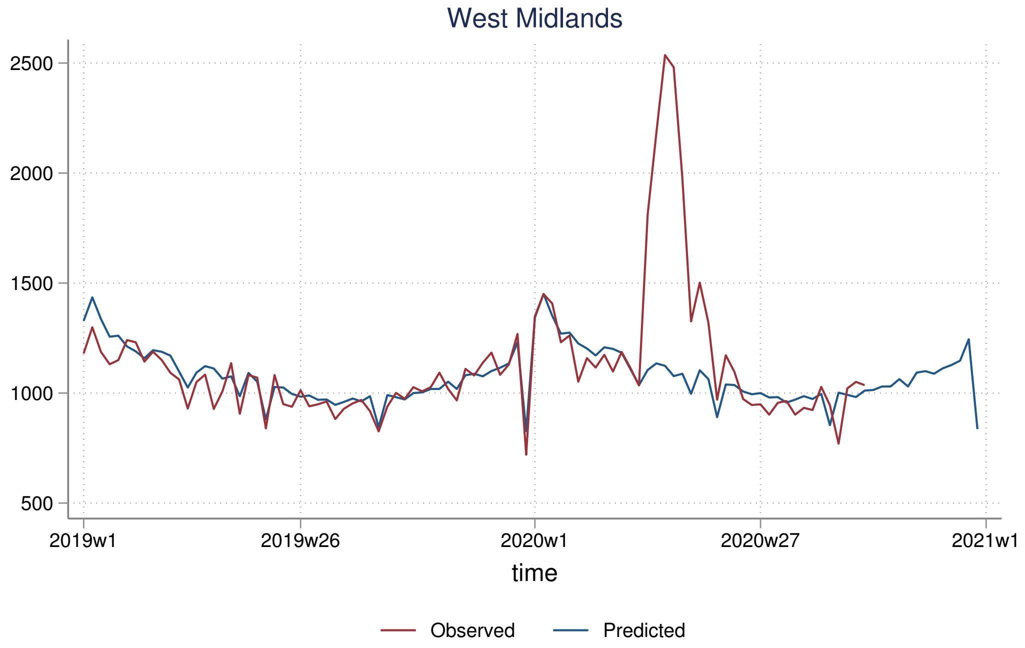

Figure 57: WMD Mortality time trend and model, from 2020 week 1

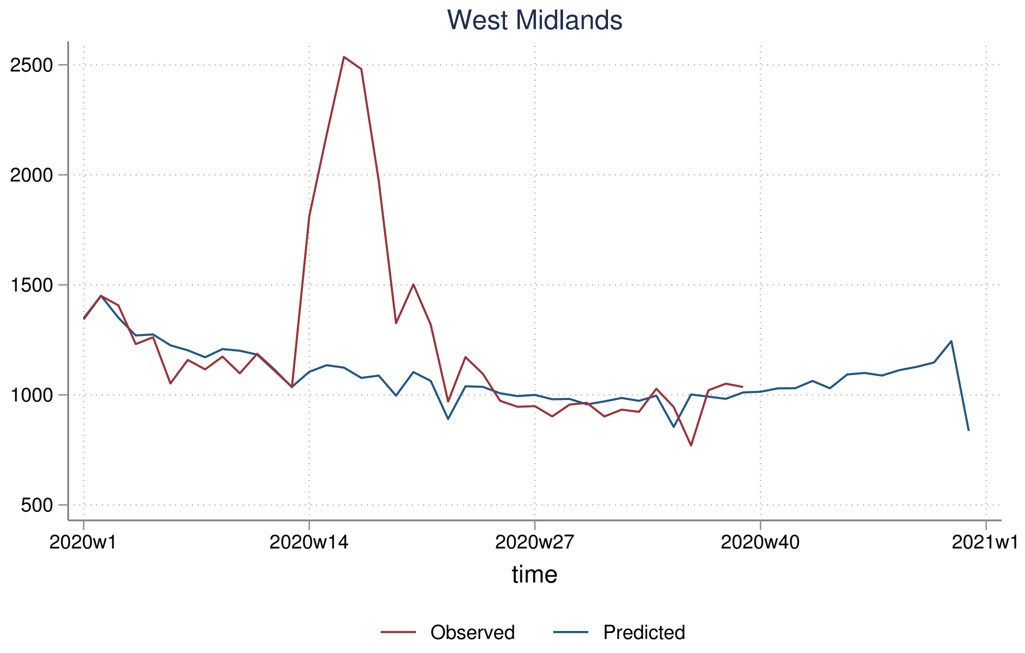

Figure 58: WMD all-cause excess deaths, from 2010 week 1

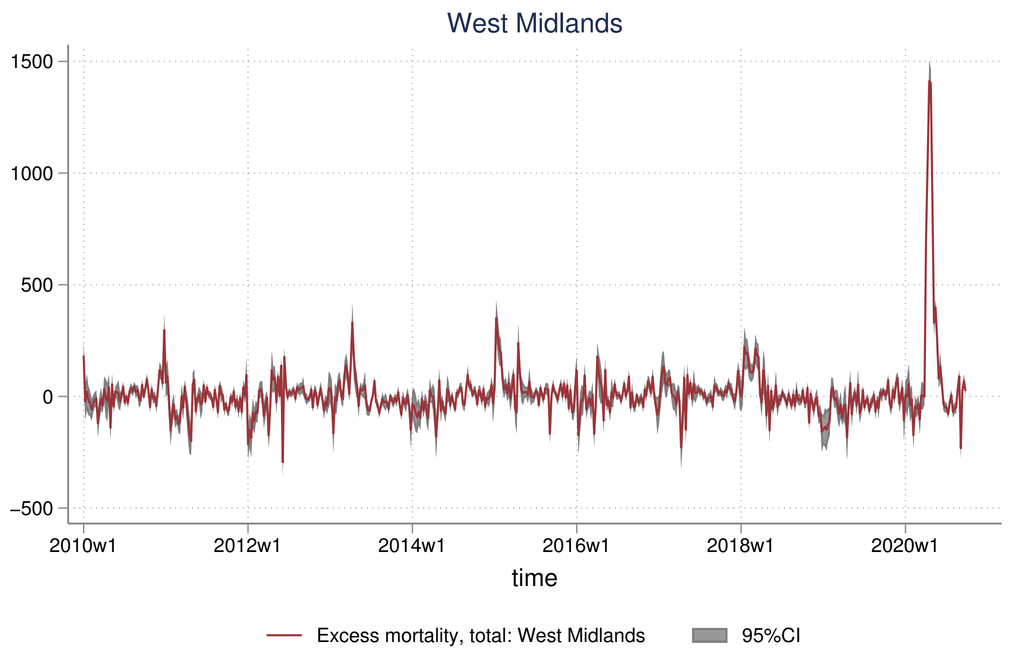

Figure 59: WMD all-cause excess deaths, from 2019 week 1

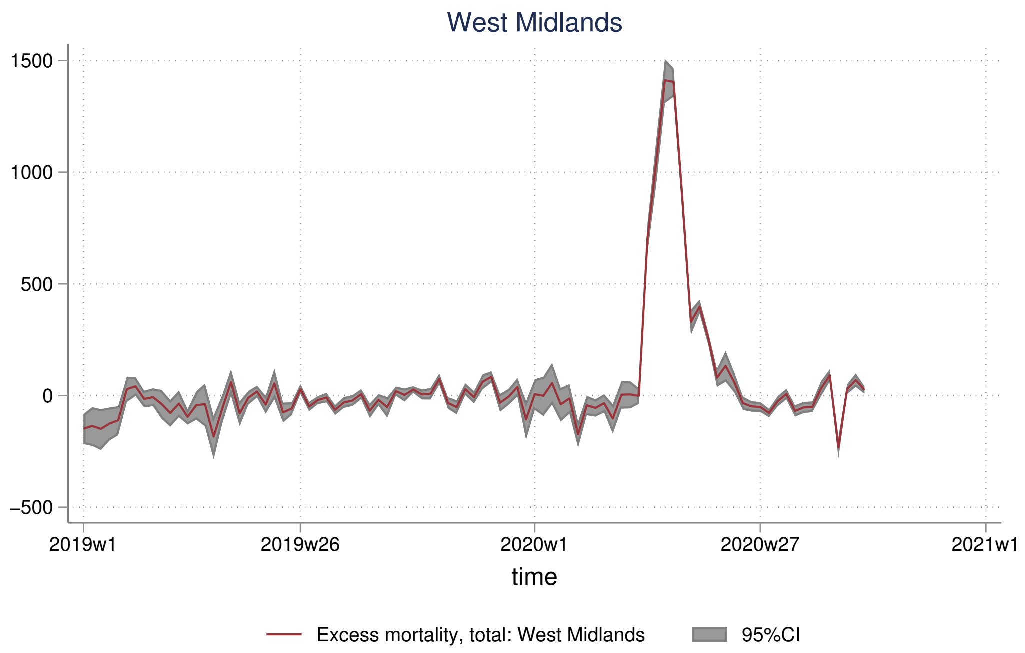

Figure 60: WMD all-cause excess deaths, from 2020 week 1

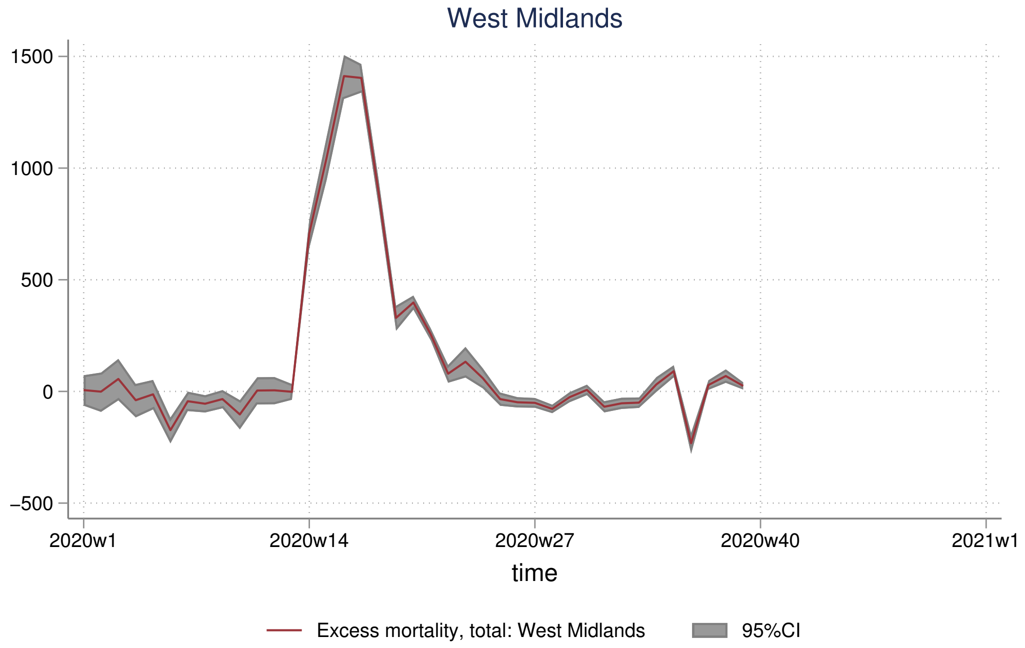

Figure 61: WMD all-cause excess deaths minus COVID19, from 2010 week 1

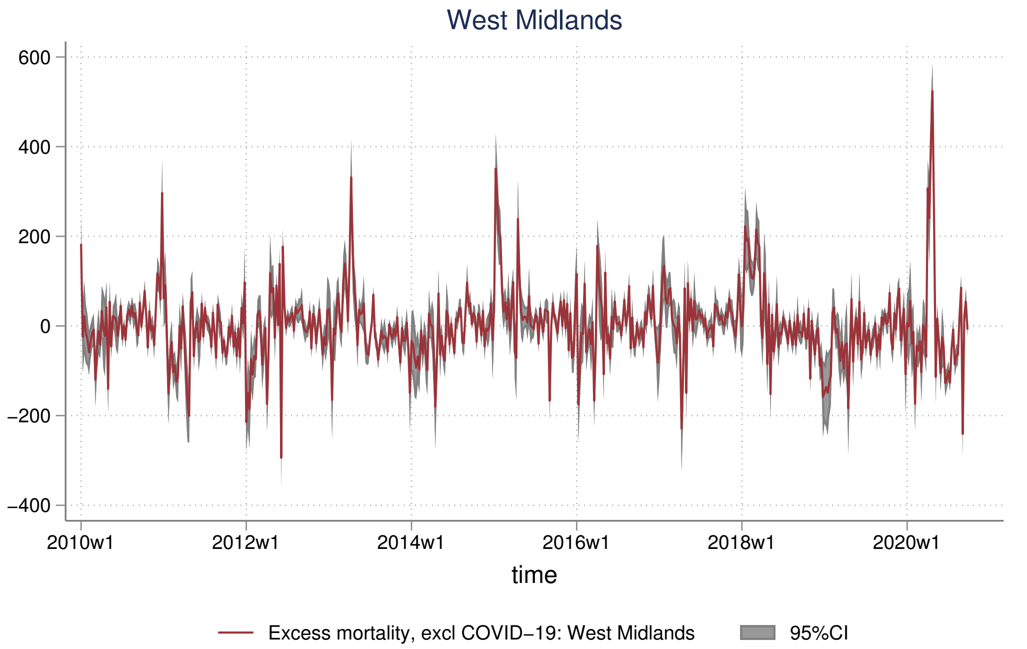

Figure 62: WMD all-cause excess deaths minus COVID19, from 2019 week 1

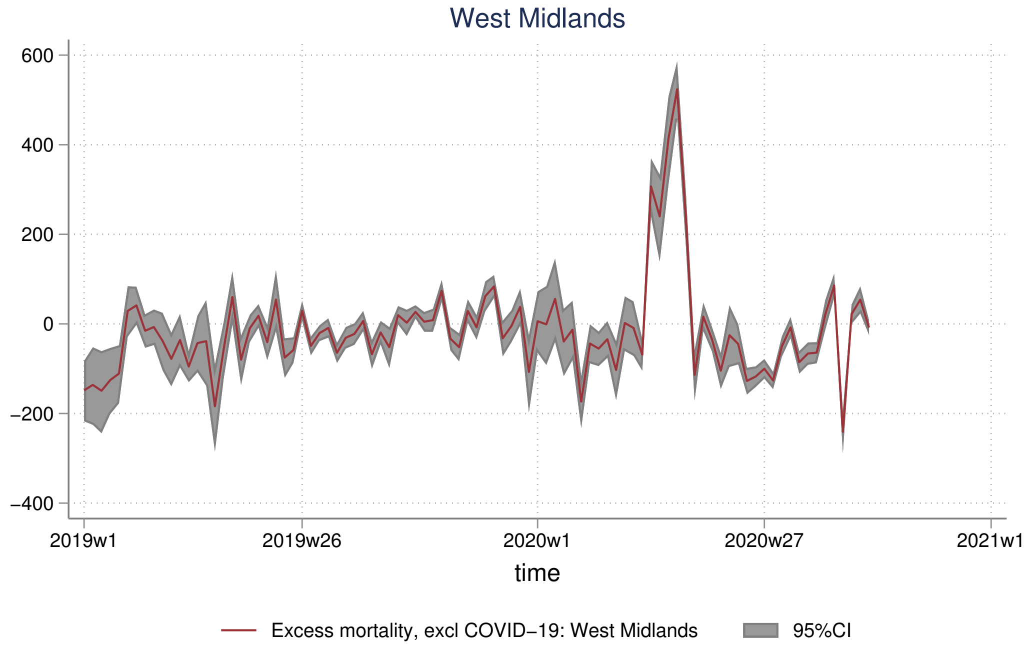

Figure 63: WMD all-cause excess deaths minus COVID19, from 2020 week 1

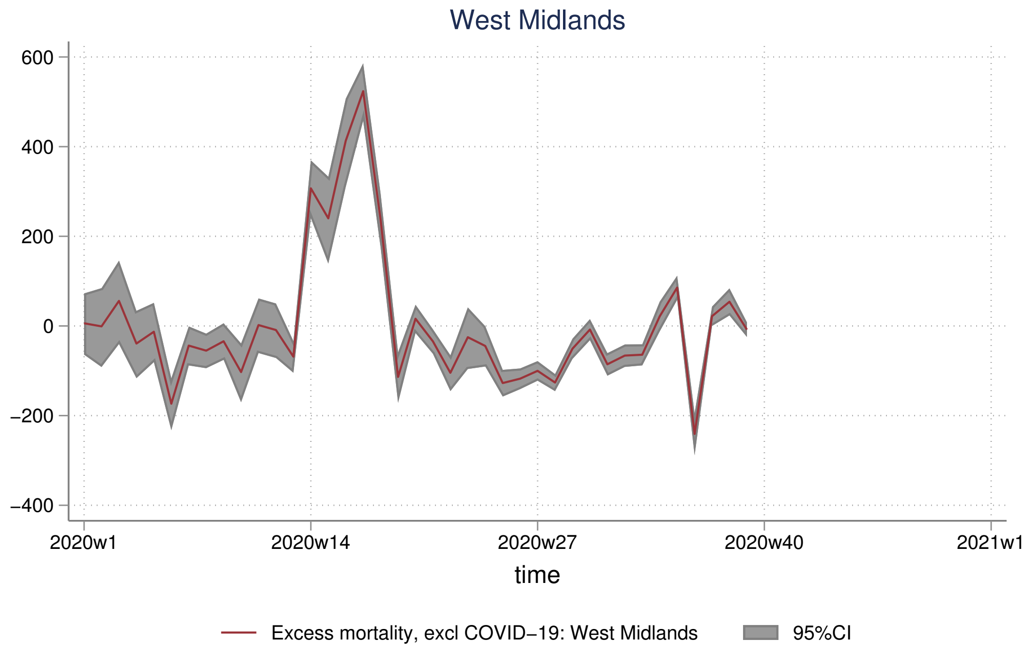

2.7 East

Figure 64: East Mortality time trend and model, from 2010 week 1

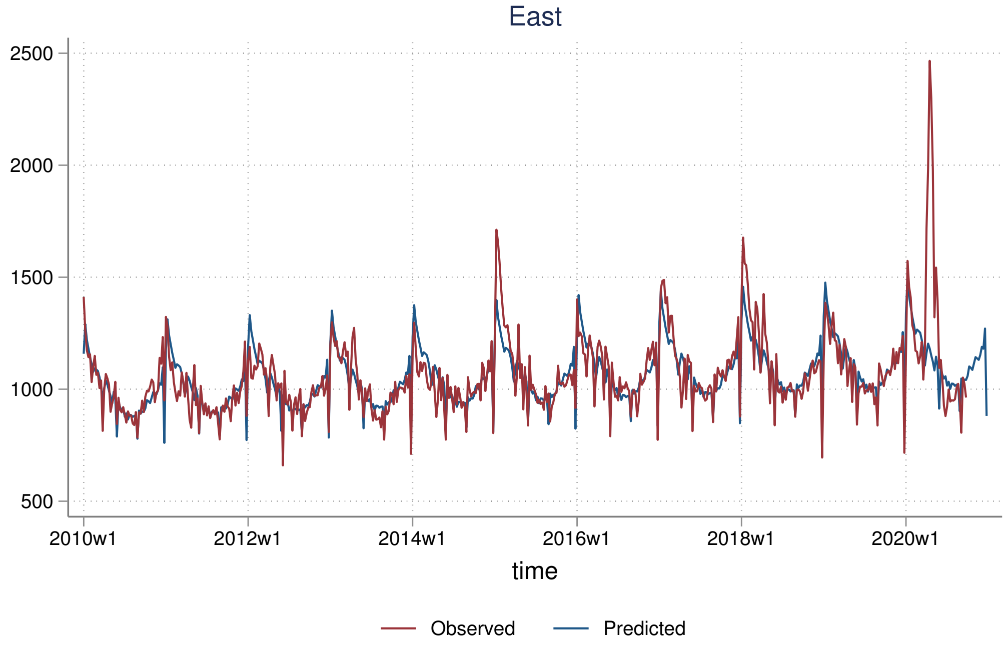

Figure 65: East Mortality time trend and model, from 2019 week 1

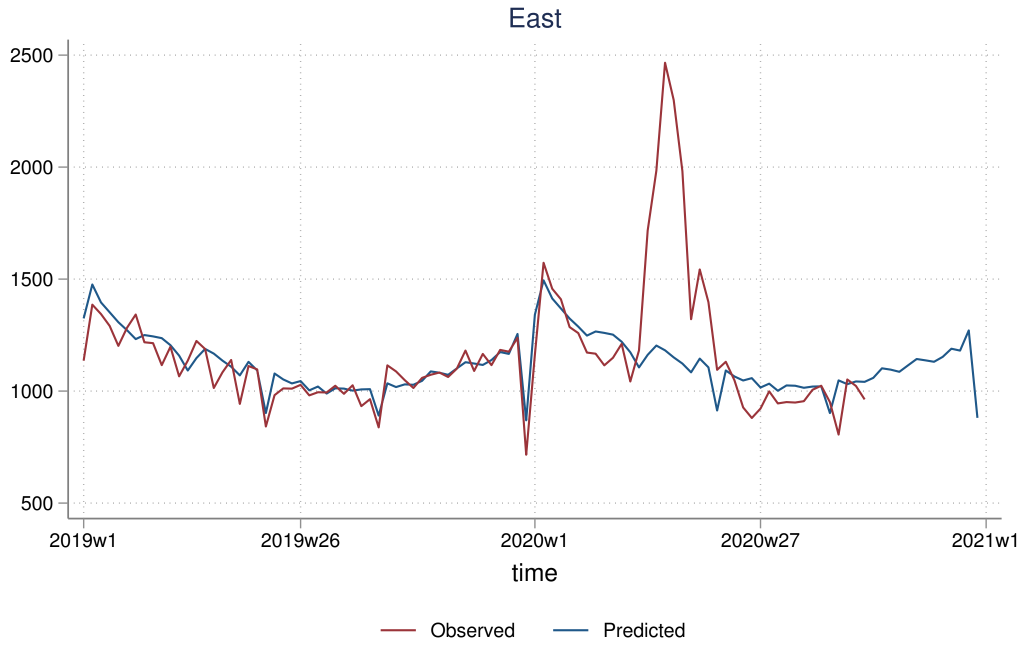

Figure 66: East Mortality time trend and model, from 2020 week 1

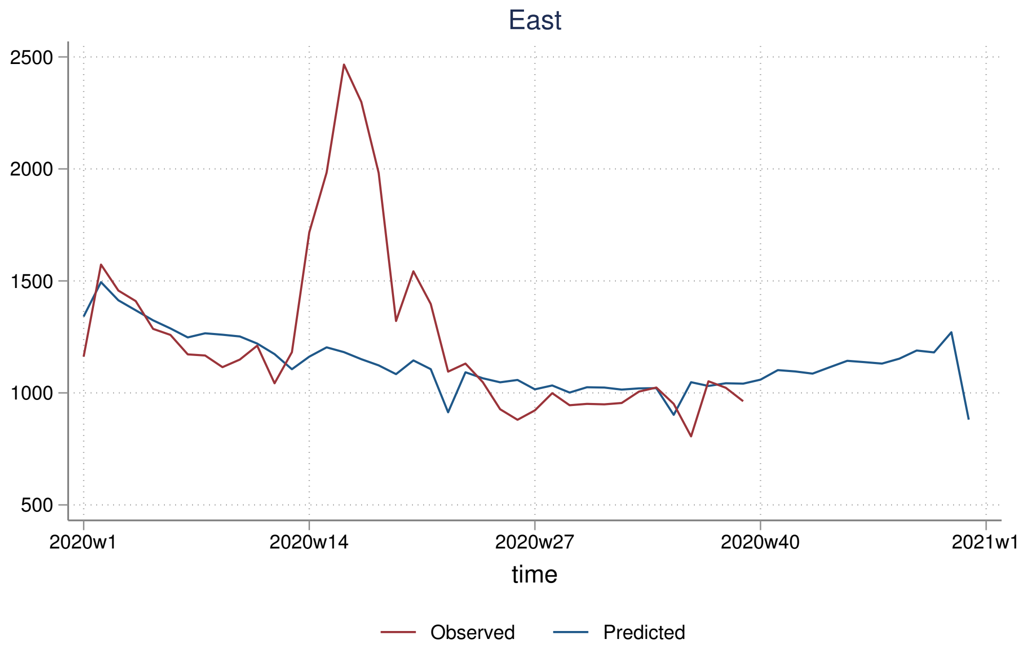

Figure 67: East all-cause excess deaths, from 2010 week 1

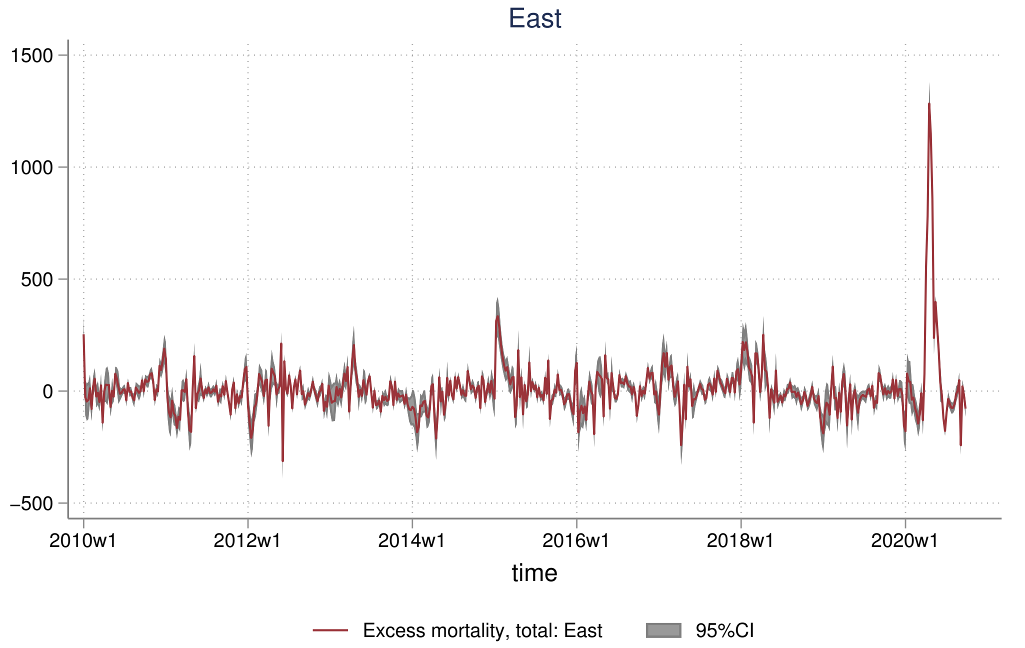

Figure 68: East all-cause excess deaths, from 2019 week 1

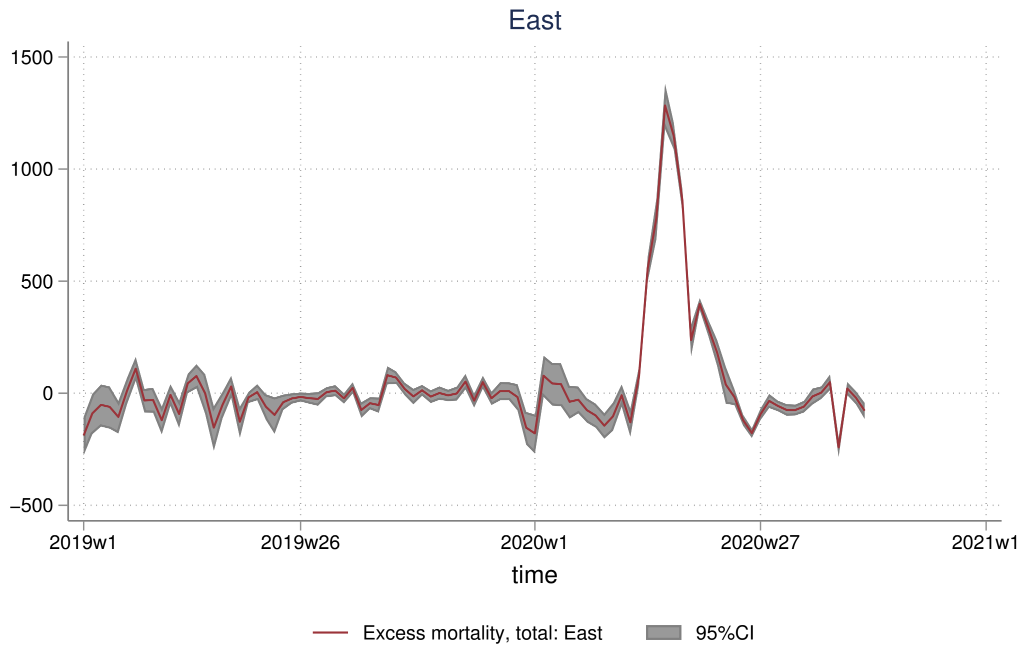

Figure 69: East all-cause excess deaths, from 2020 week 1

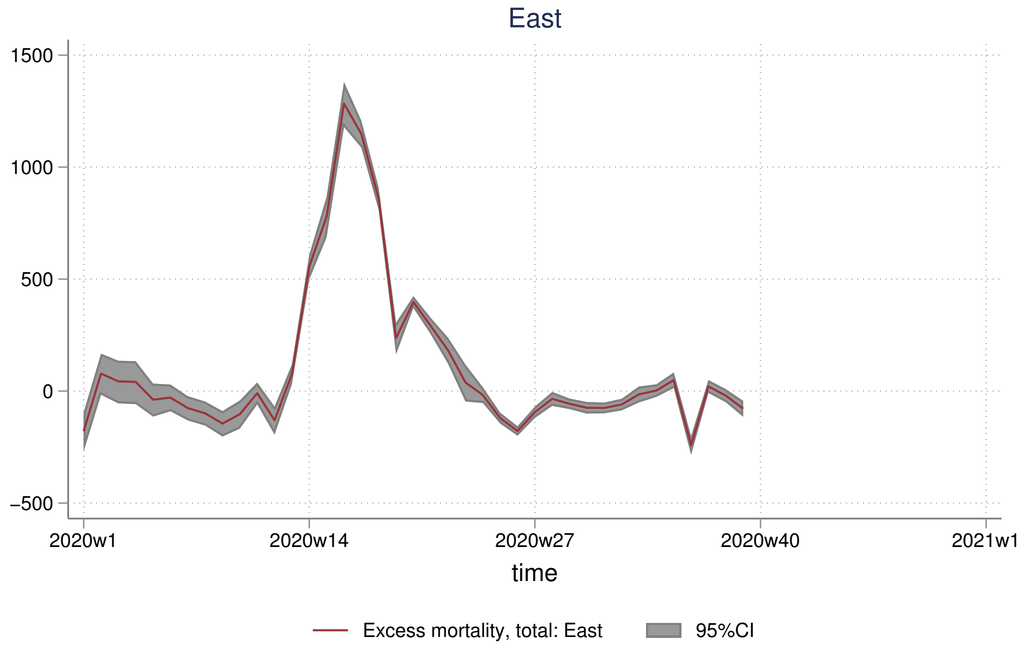

Figure 70: East all-cause excess deaths minus COVID19, from 2010 week 1

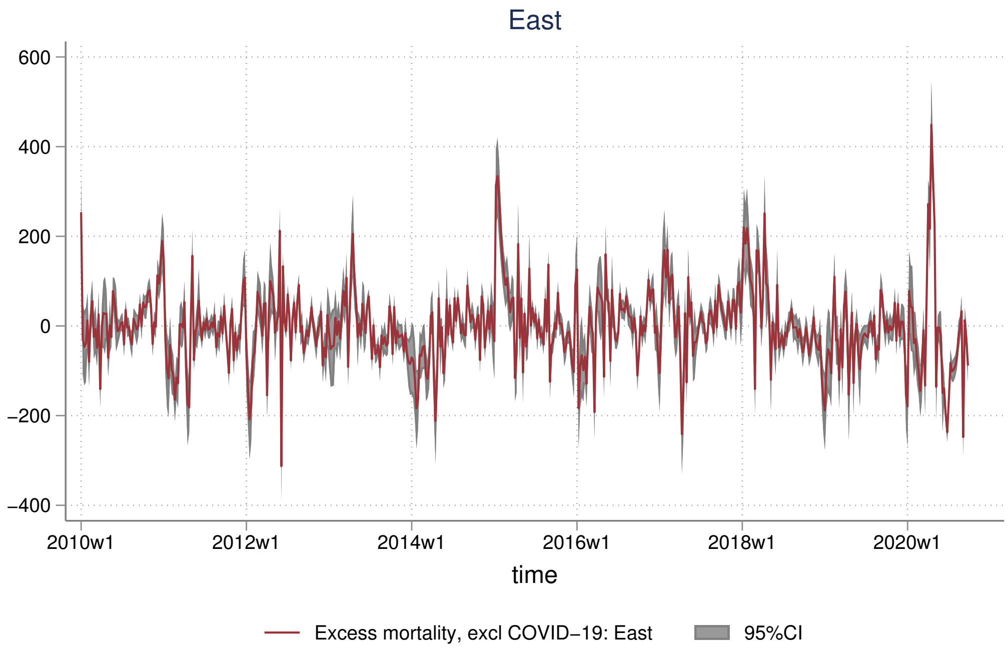

Figure 71: East all-cause excess deaths minus COVID19, from 2019 week 1

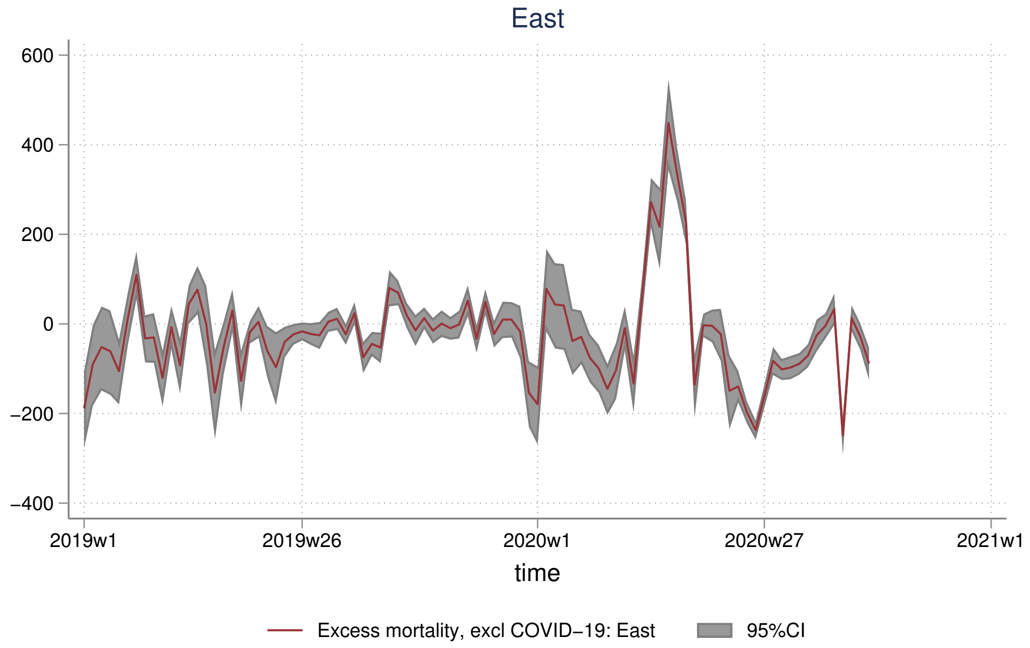

Figure 72: East all-cause excess deaths minus COVID19, from 2020 week 1

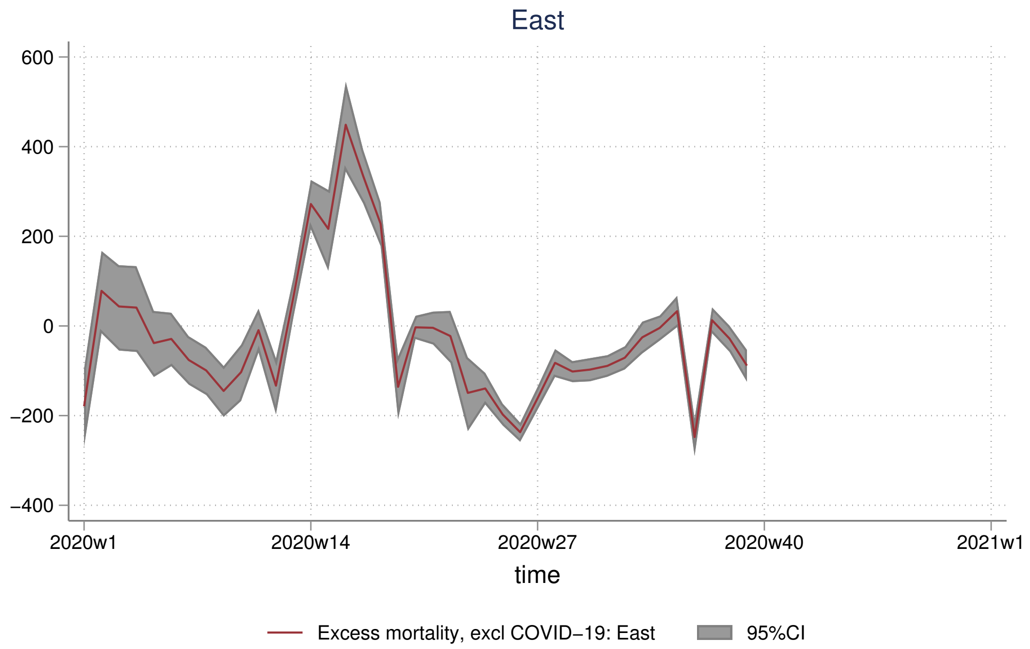

2.8 London

Figure 73: London Mortality time trend and model, from 2010 week 1

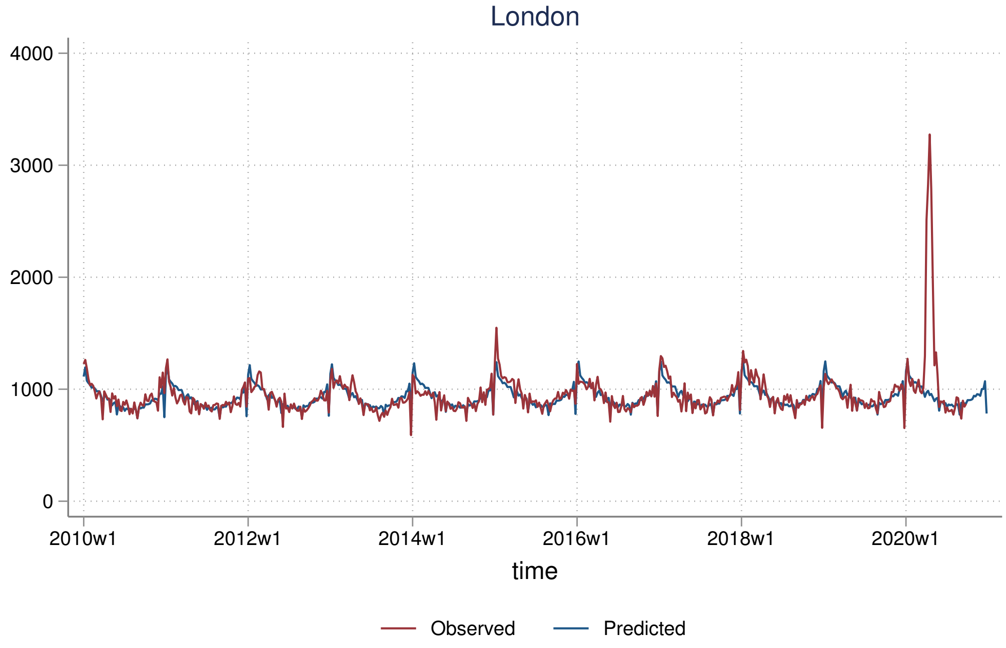

Figure 74: London Mortality time trend and model, from 2019 week 1

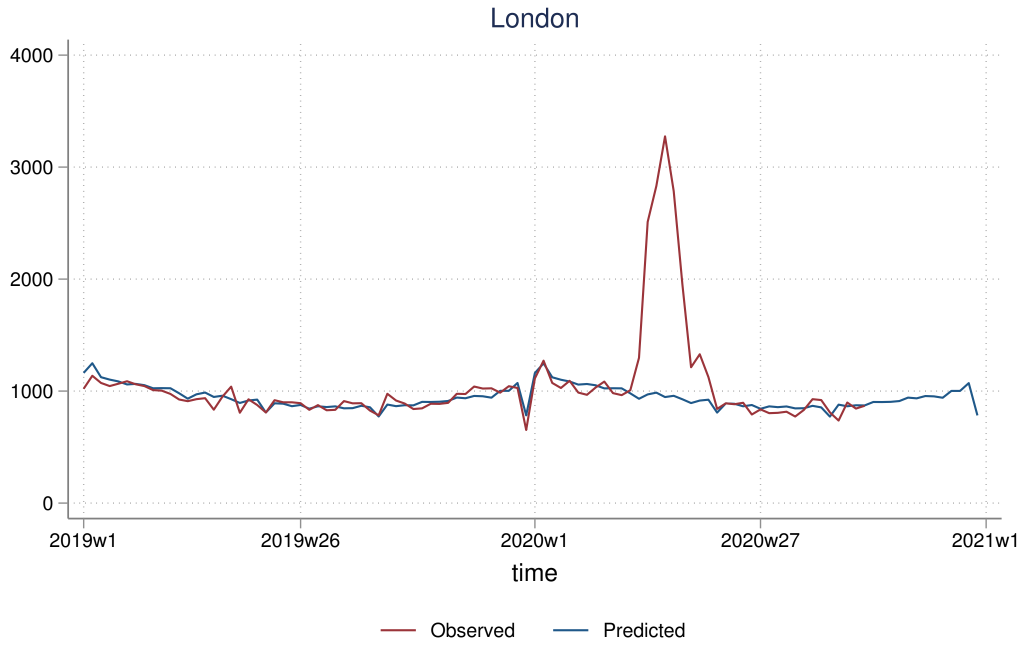

Figure 75: London Mortality time trend and model, from 2020 week 1

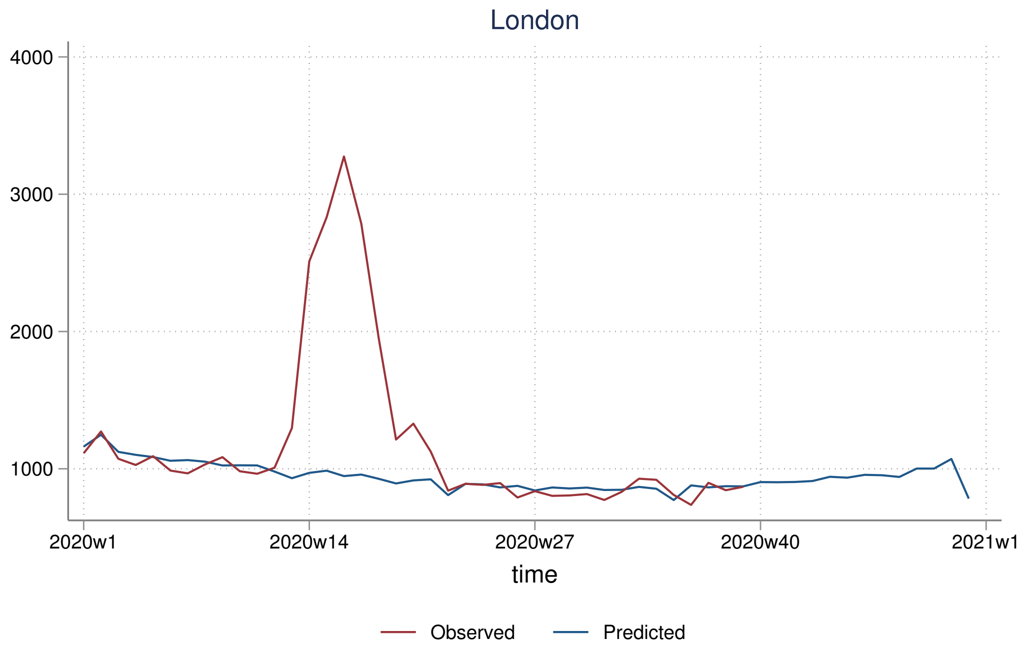

Figure 76: London all-cause excess deaths, from 2010 week 1

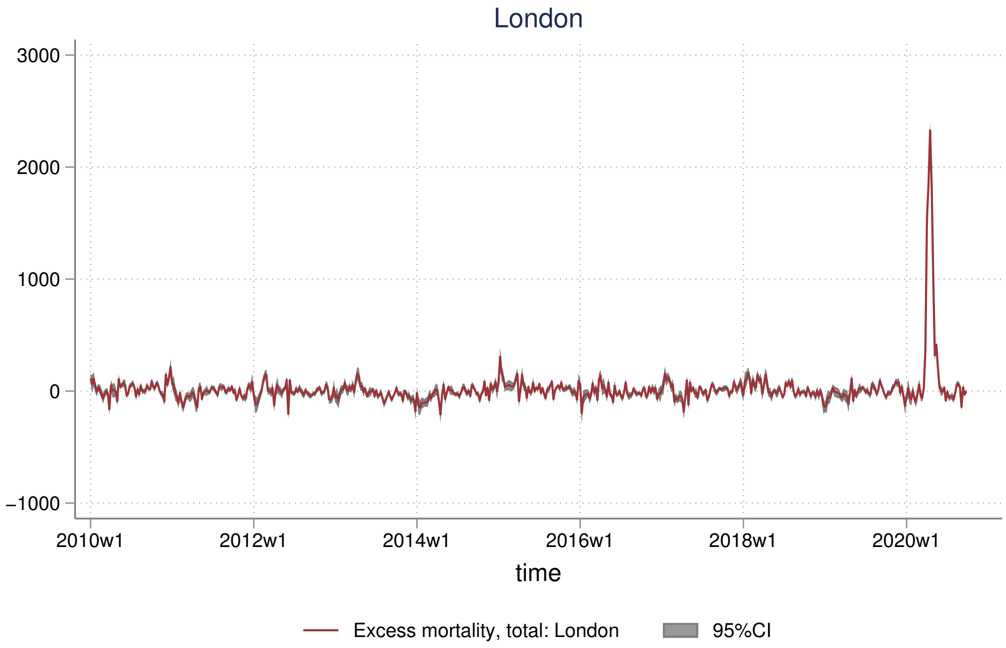

Figure 77: London all-cause excess deaths, from 2019 week 1

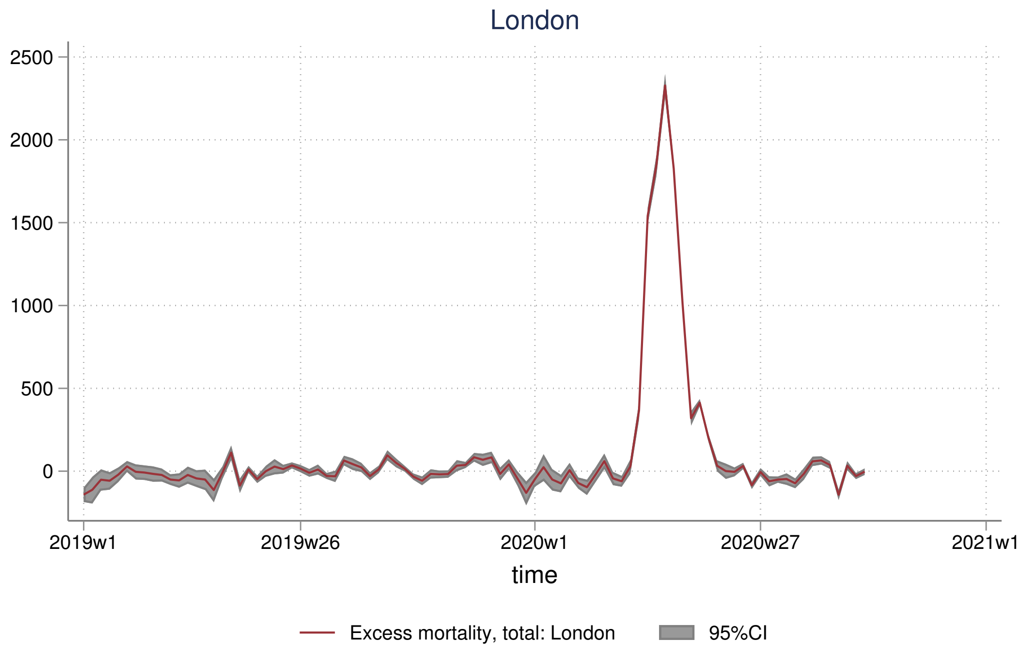

Figure 78: London all-cause excess deaths, from 2020 week 1

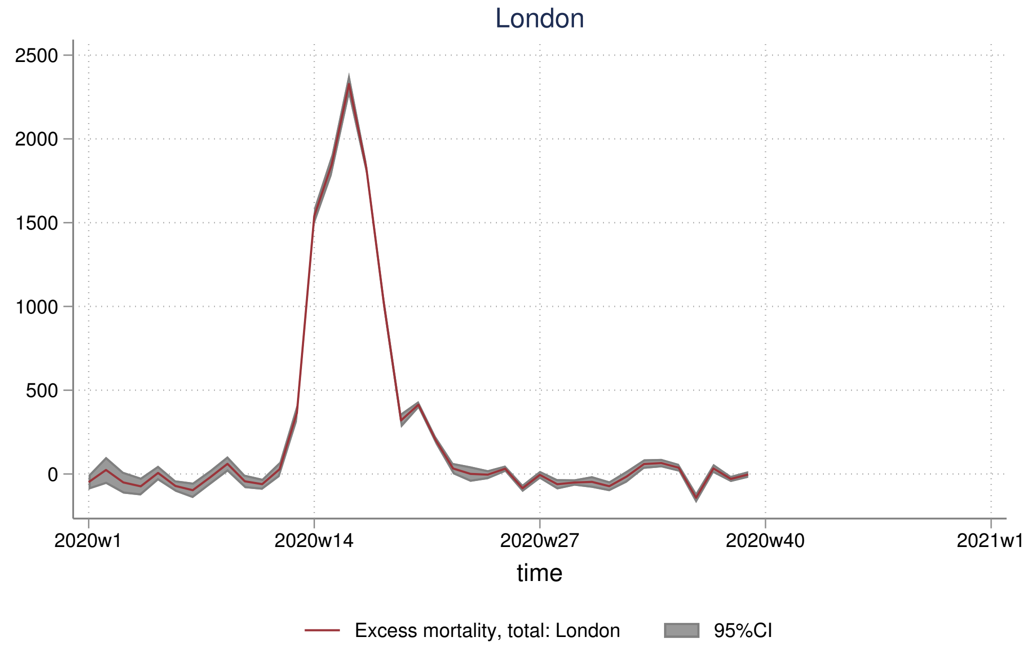

Figure 79: London all-cause excess deaths minus COVID19, from 2010 week 1

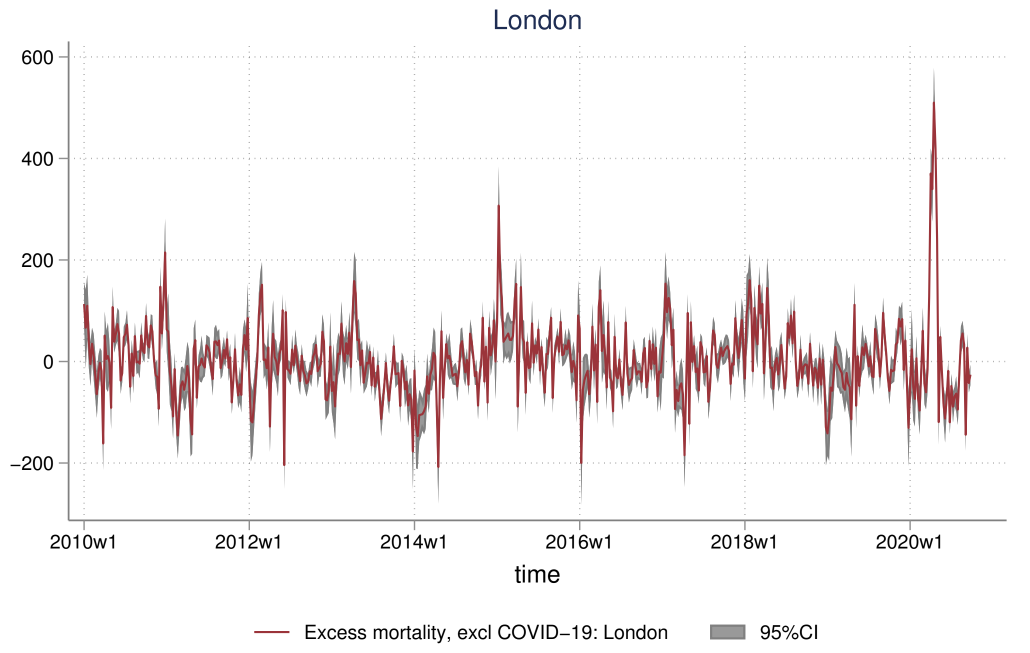

Figure 80: London all-cause excess deaths minus COVID19, from 2019 week 1

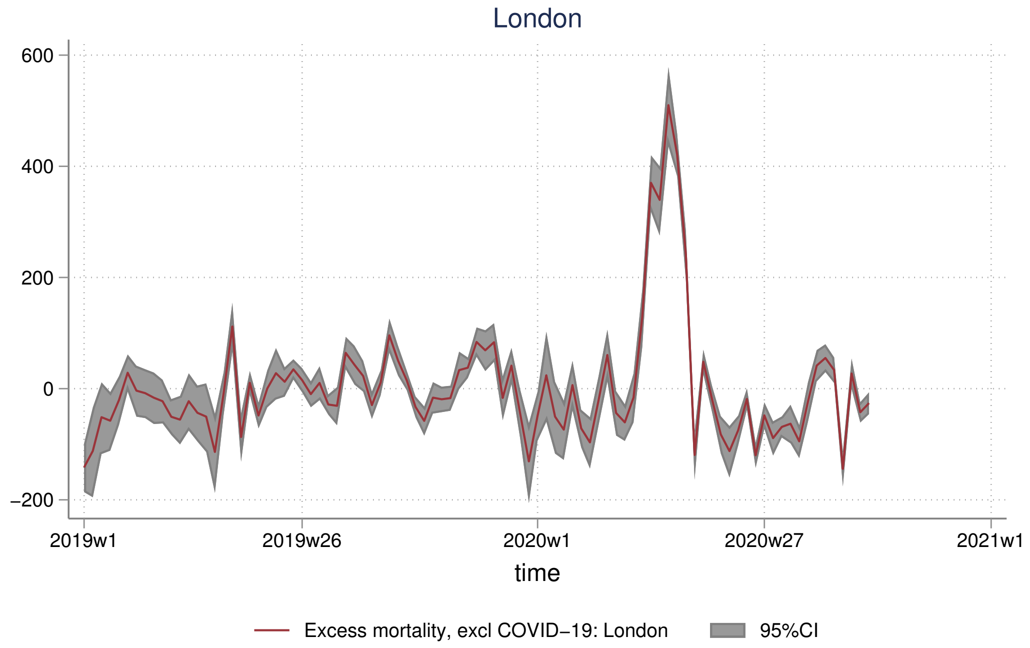

Figure 81: London all-cause excess deaths minus COVID19, from 2020 week 1

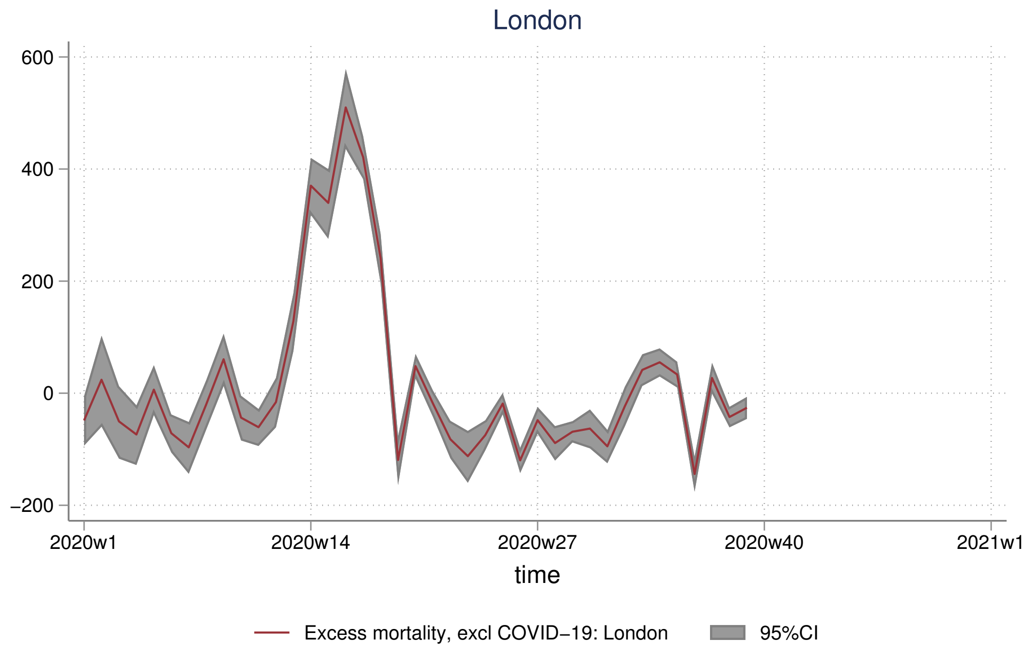

2.9 South East

Figure 82: SE Mortality time trend and model, from 2010 week 1

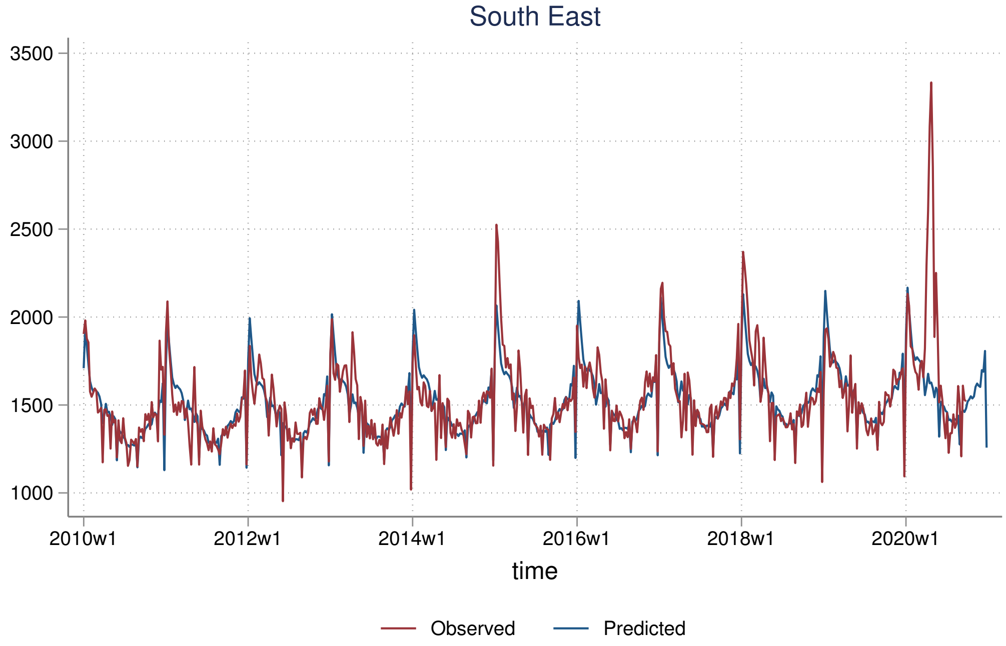

Figure 83: SE Mortality time trend and model, from 2019 week 1

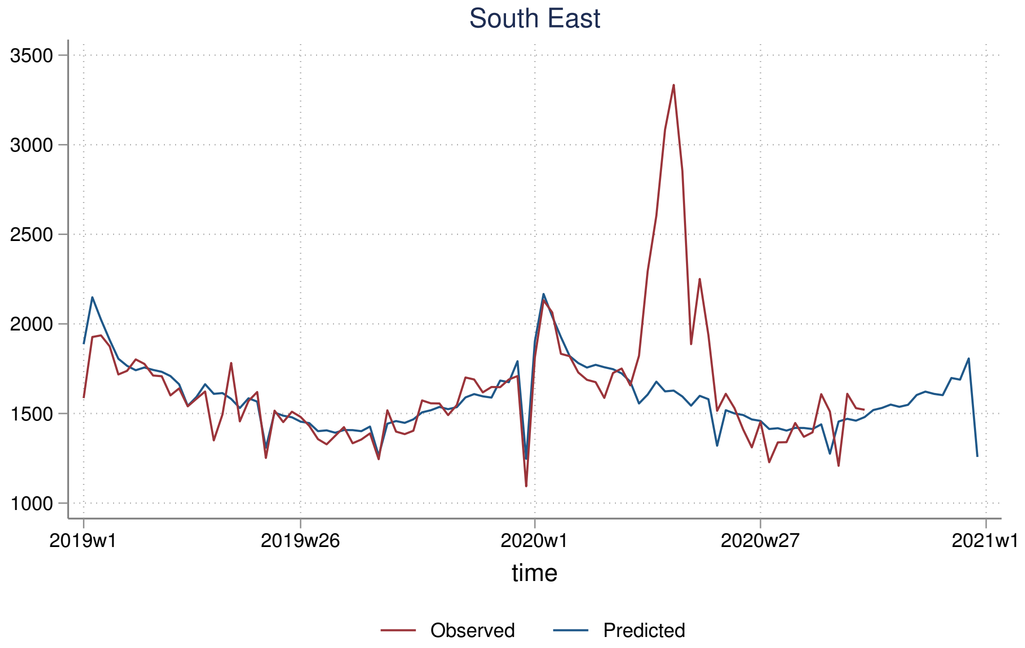

Figure 84: SE Mortality time trend and model, from 2020 week 1

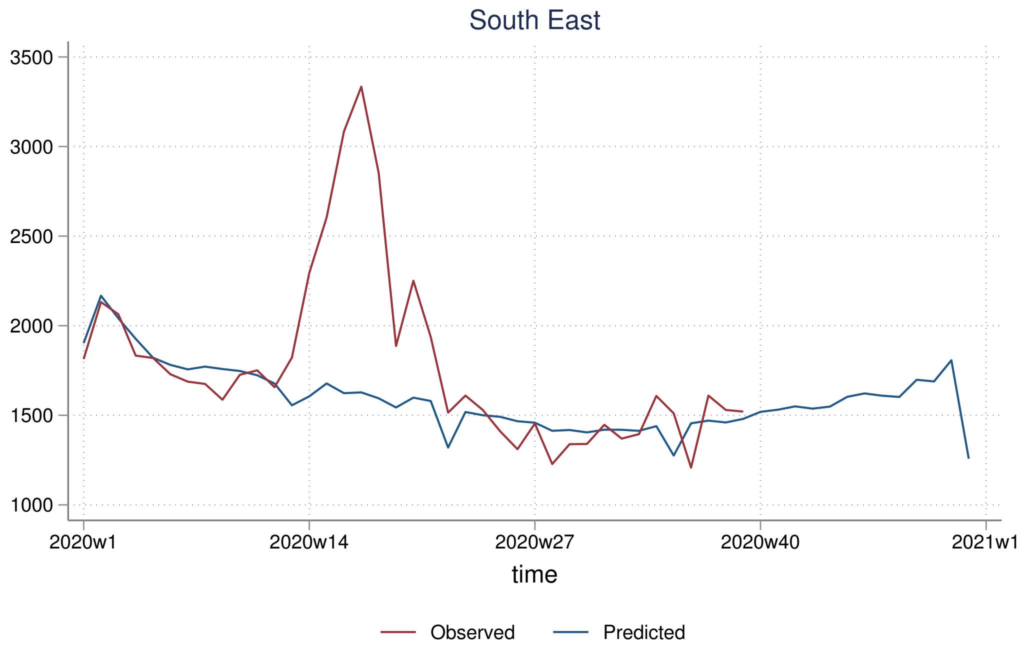

Figure 85: SE all-cause excess deaths, from 2010 week 1

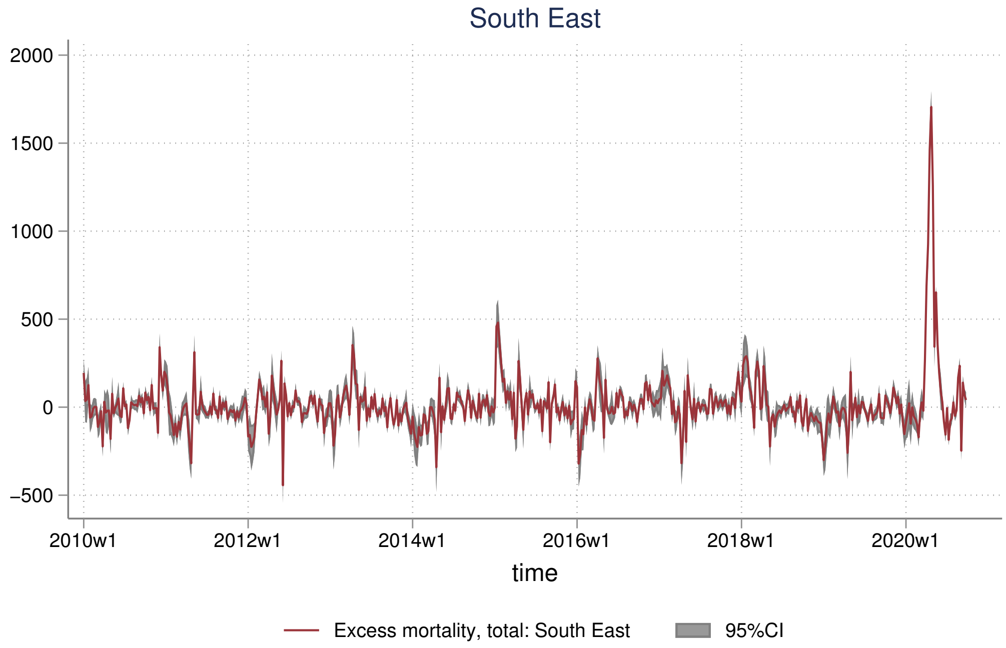

Figure 86: SE all-cause excess deaths, from 2019 week 1

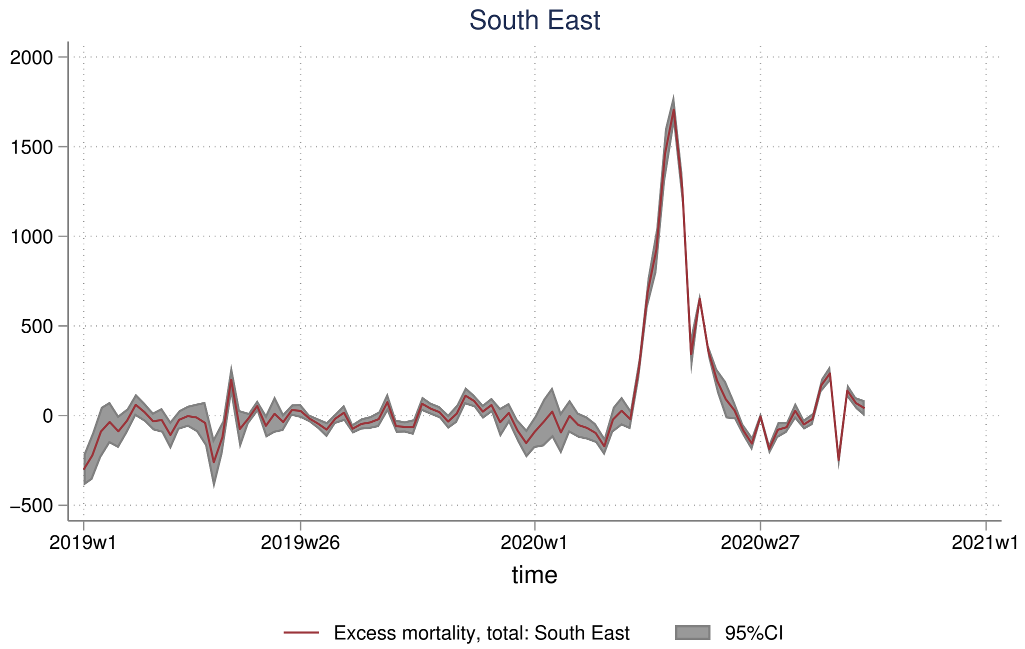

Figure 87: SE all-cause excess deaths, from 2020 week 1

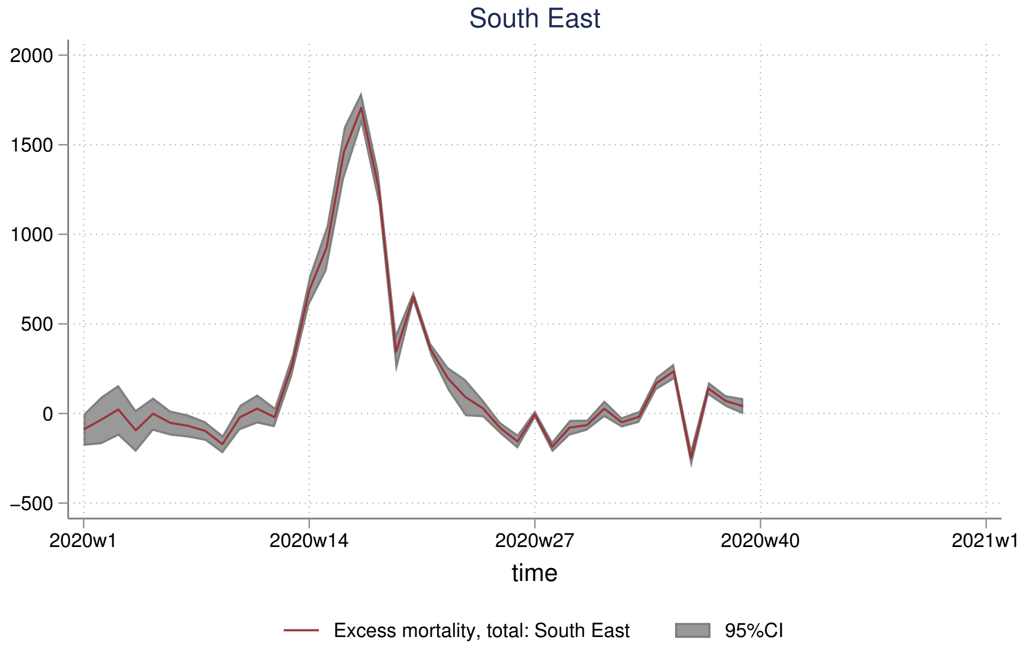

Figure 88: SE all-cause excess deaths minus COVID19, from 2010 week 1

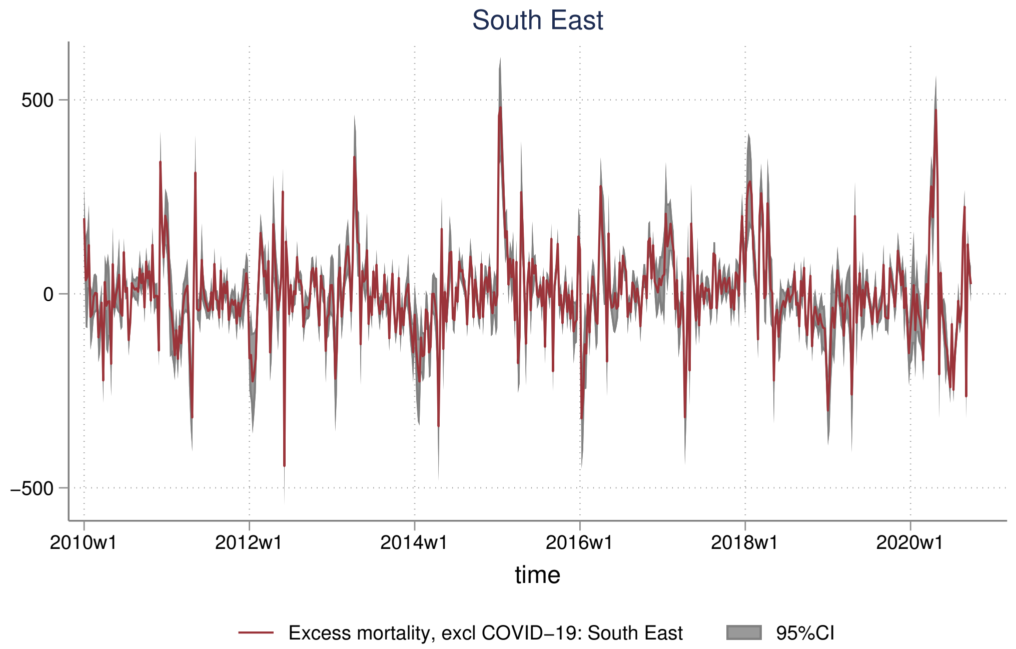

Figure 89: SE all-cause excess deaths minus COVID19, from 2019 week 1

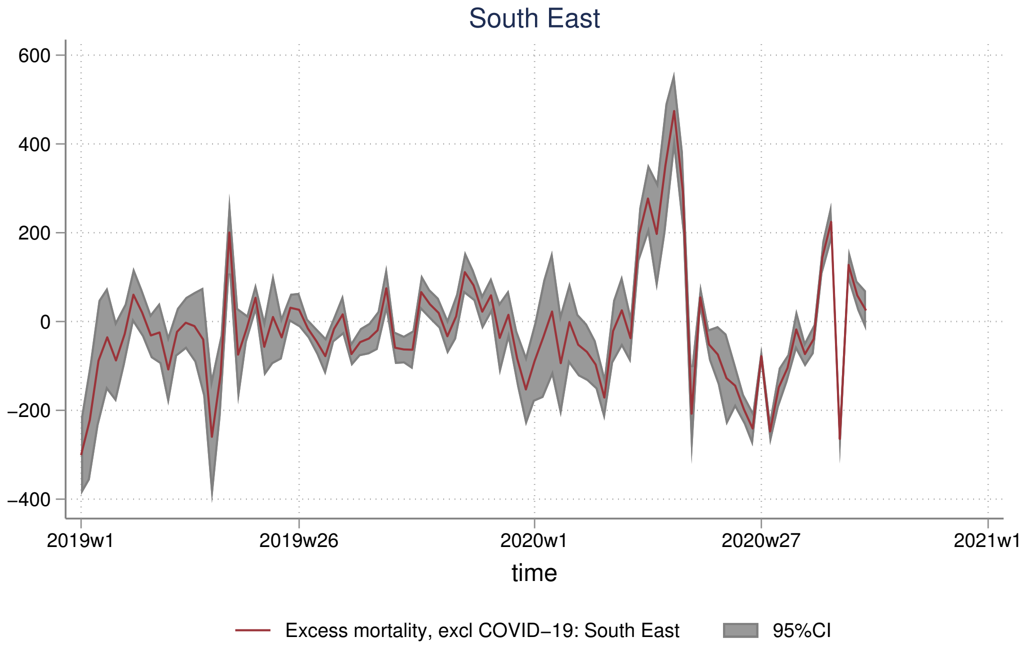

Figure 90: SE all-cause excess deaths minus COVID19, from 2020 week 1

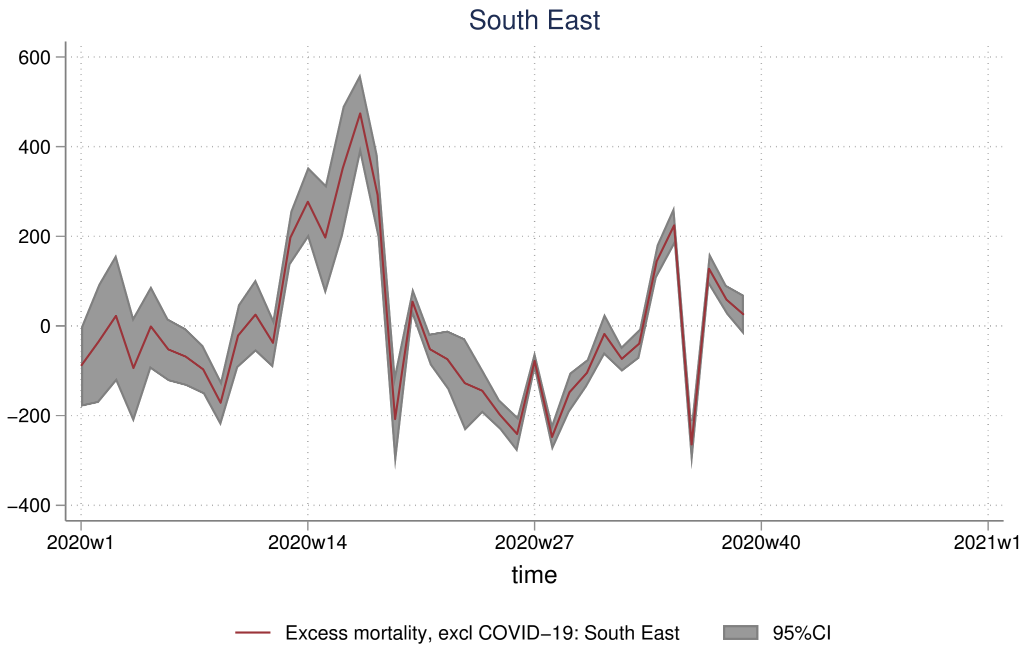

2.10 South West

Figure 91: SW Mortality time trend and model, from 2010 week 1

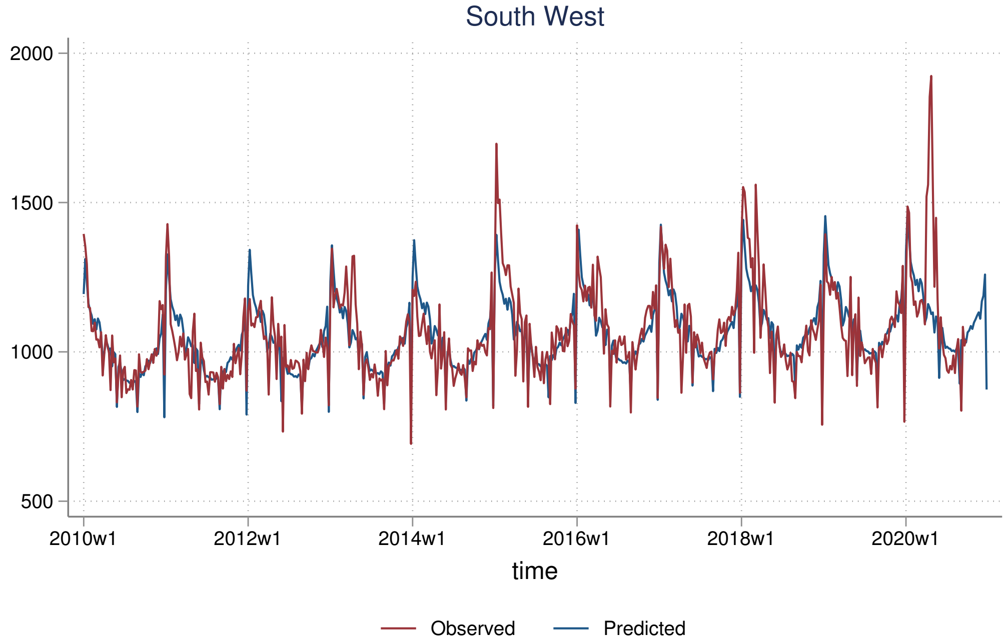

Figure 92: SE Mortality time trend and model, from 2019 week 1

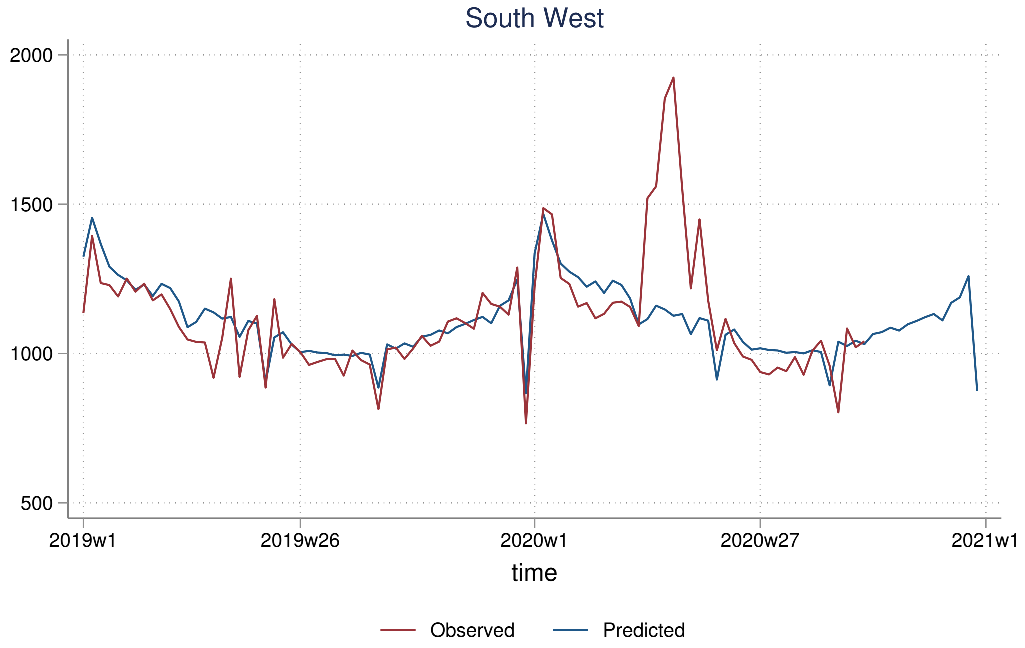

Figure 93: SW Mortality time trend and model, from 2020 week 1

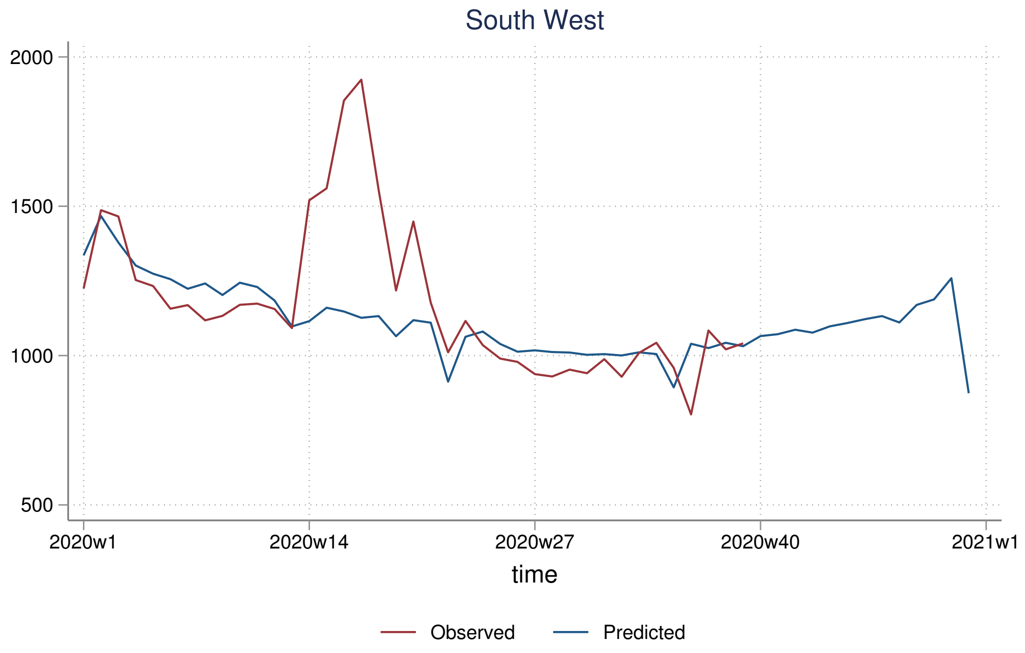

Figure 94: SW all-cause excess deaths, from 2010 week 1

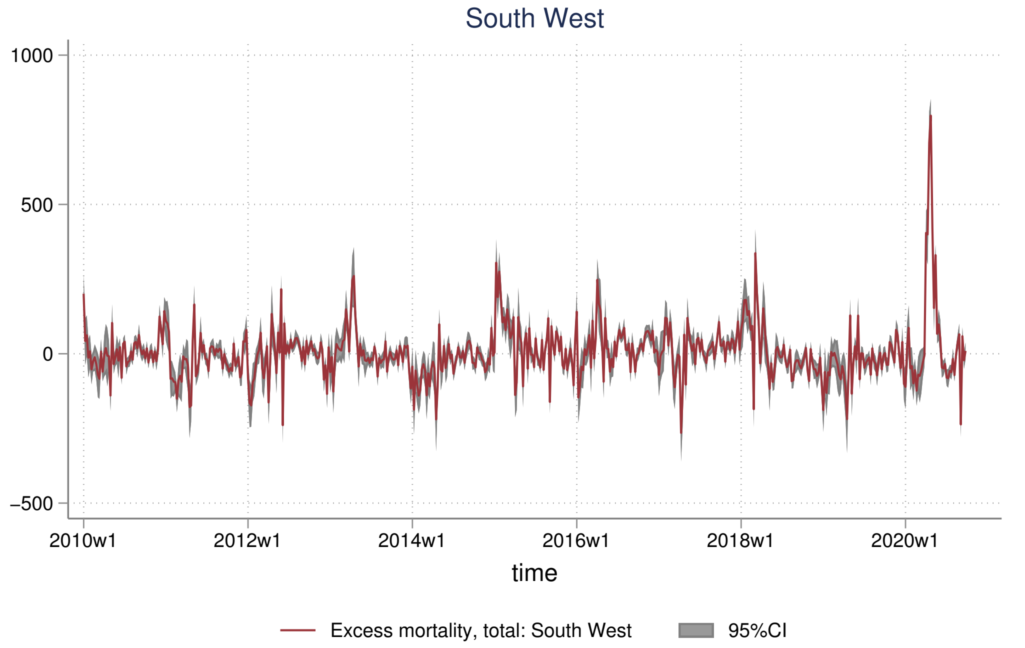

Figure 95: SW all-cause excess deaths, from 2019 week 1

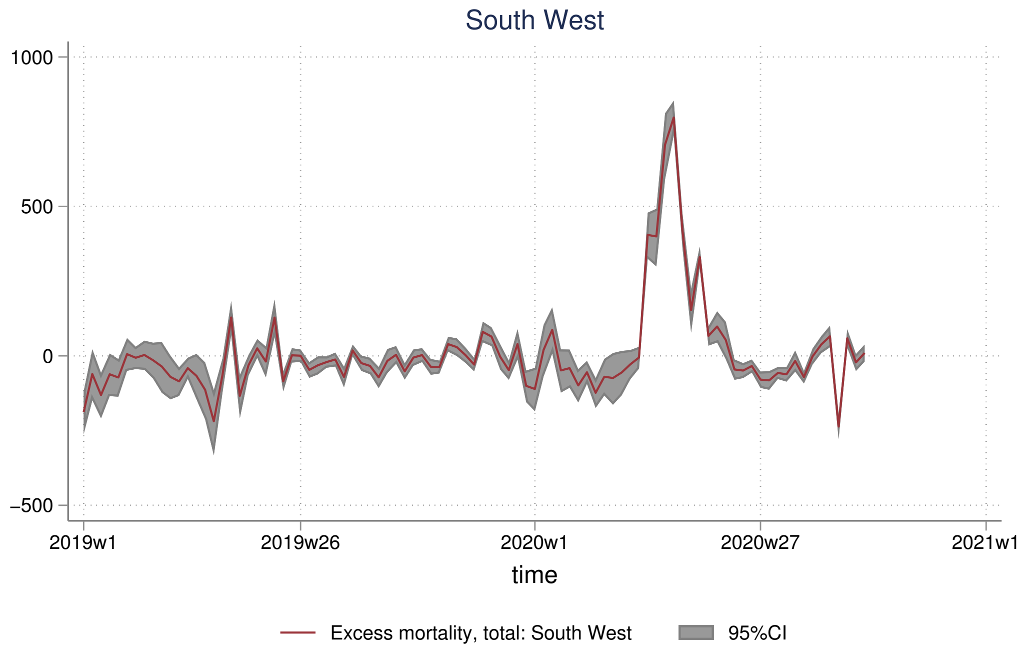

Figure 96: SW all-cause excess deaths, from 2020 week 1

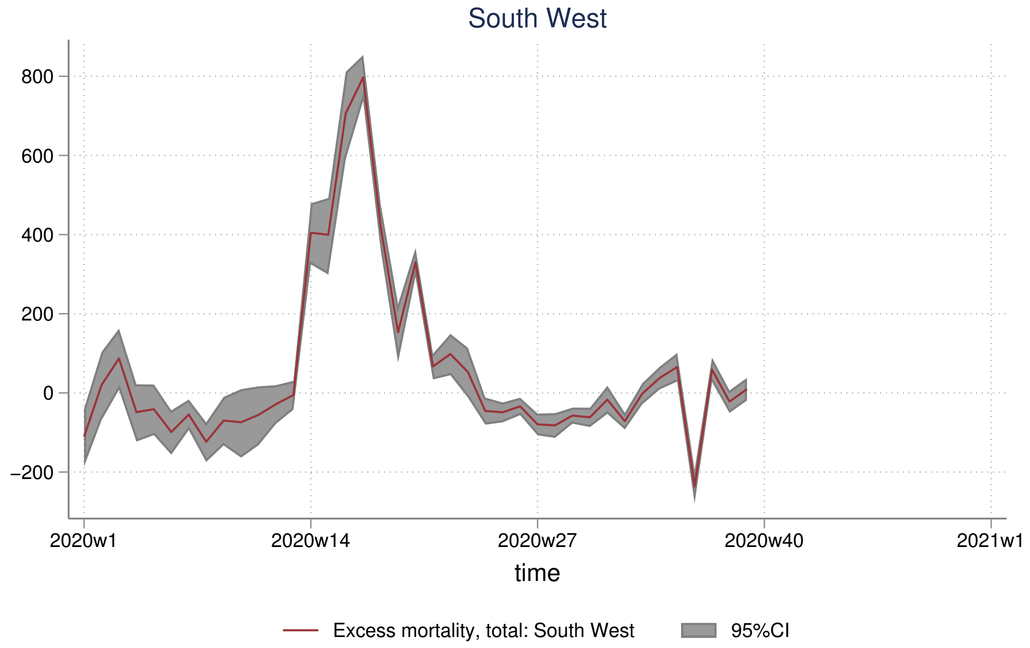

Figure 97: SW all-cause excess deaths minus COVID19, from 2010 week 1

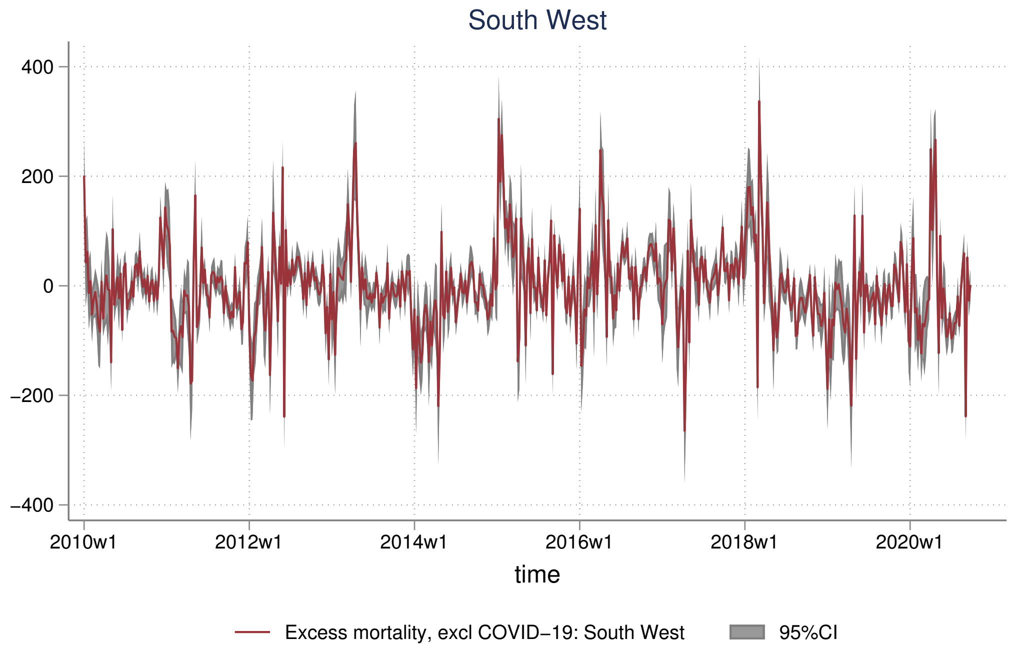

Figure 98: SW all-cause excess deaths minus COVID19, from 2019 week 1

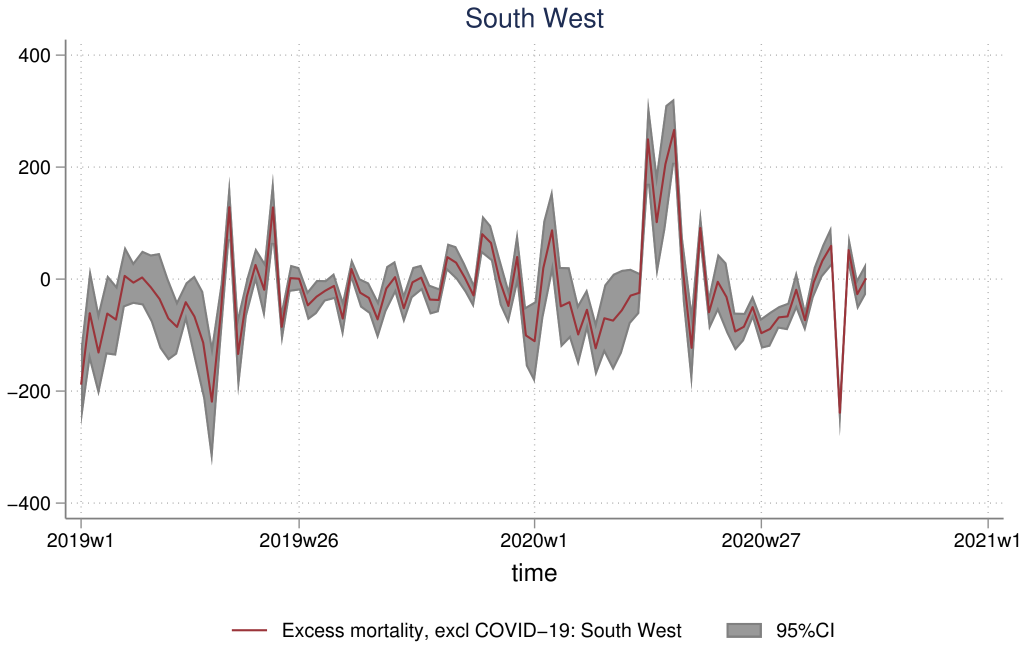

Figure 99: SW all-cause excess deaths minus COVID19, from 2020 week 1

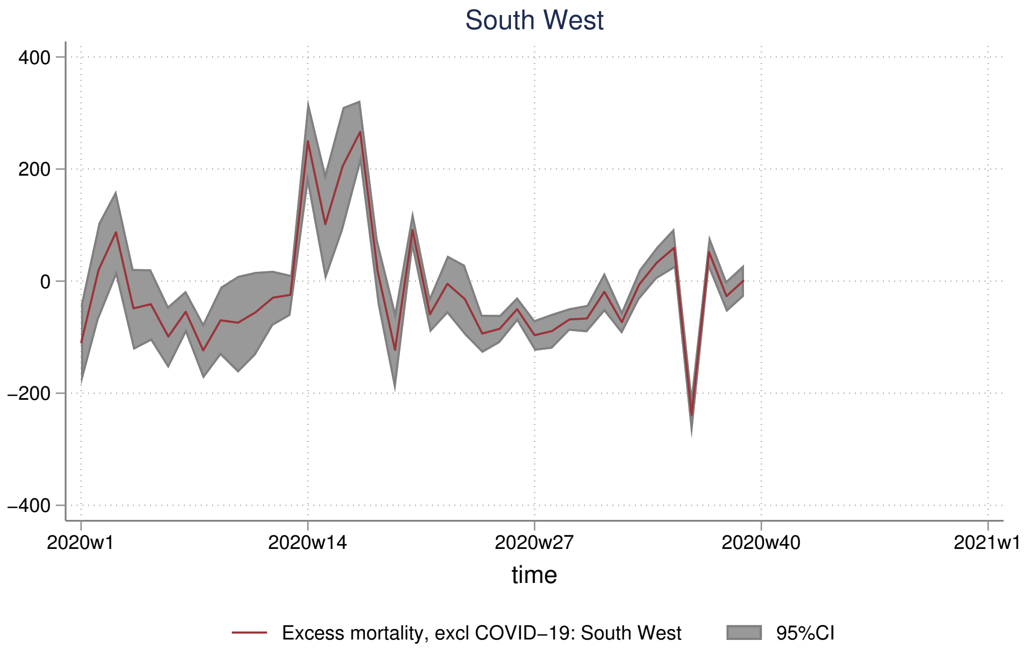

2.11 Wales

Figure 100: Wales Mortality time trend and model, from 2010 week 1

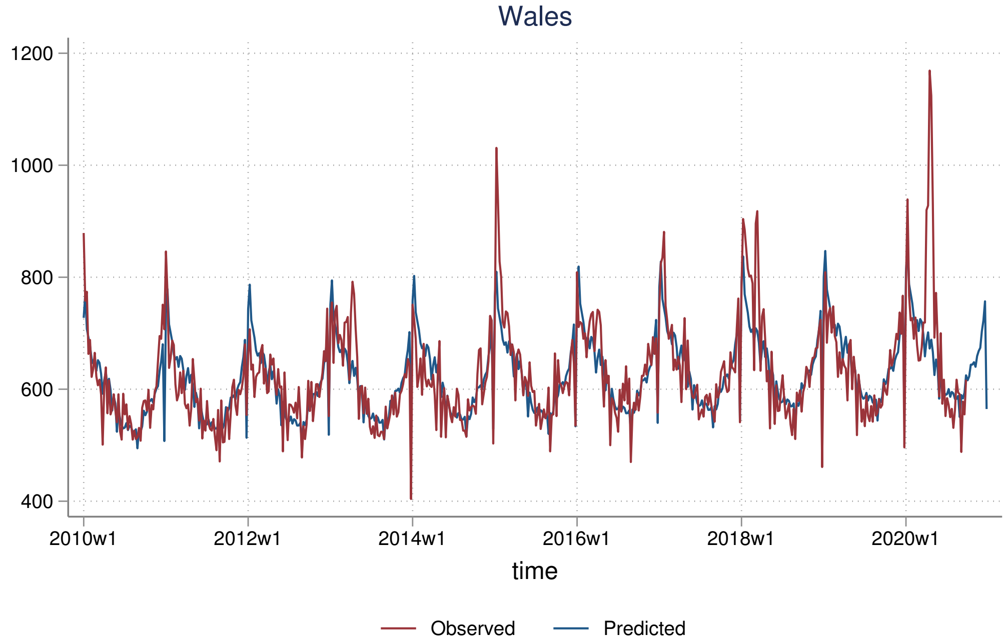

Figure 101: SE Mortality time trend and model, from 2019 week 1

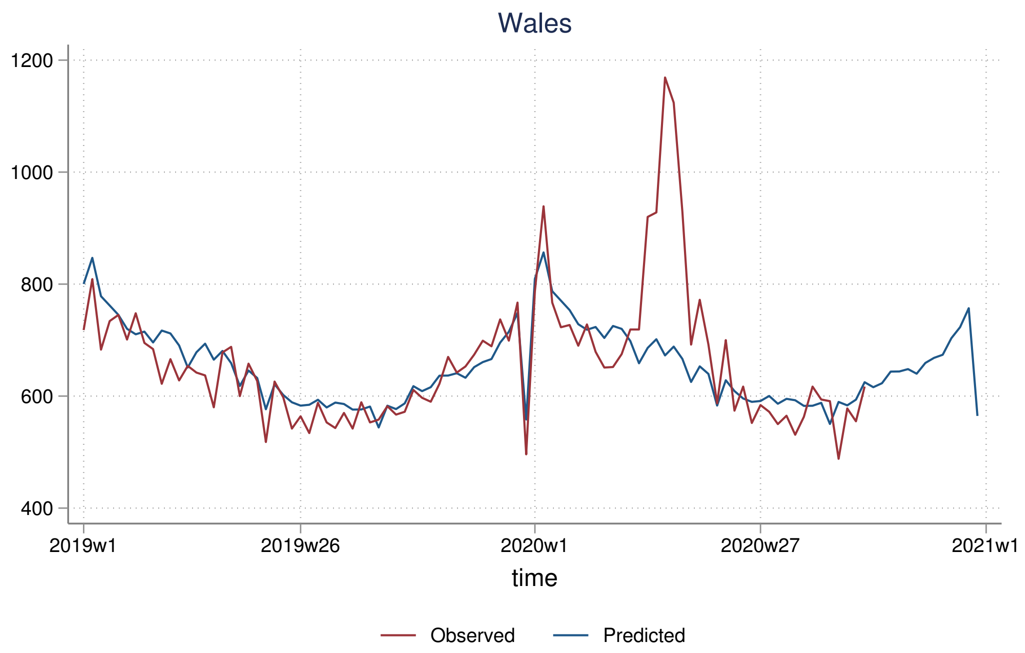

Figure 102: Wales Mortality time trend and model, from 2020 week 1

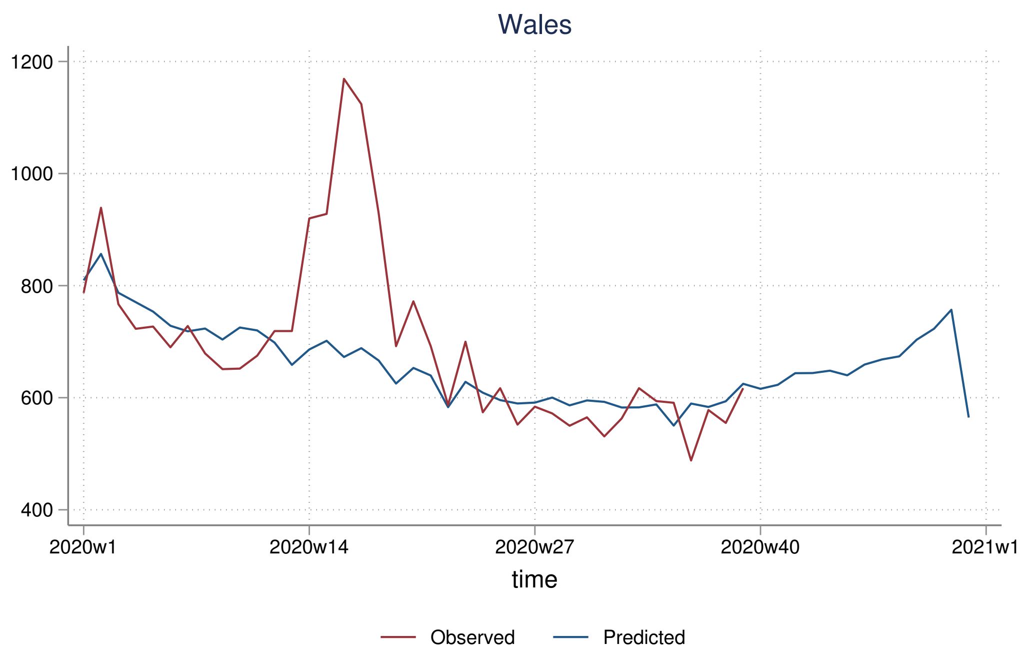

Figure 103: Wales all-cause excess deaths, from 2010 week 1

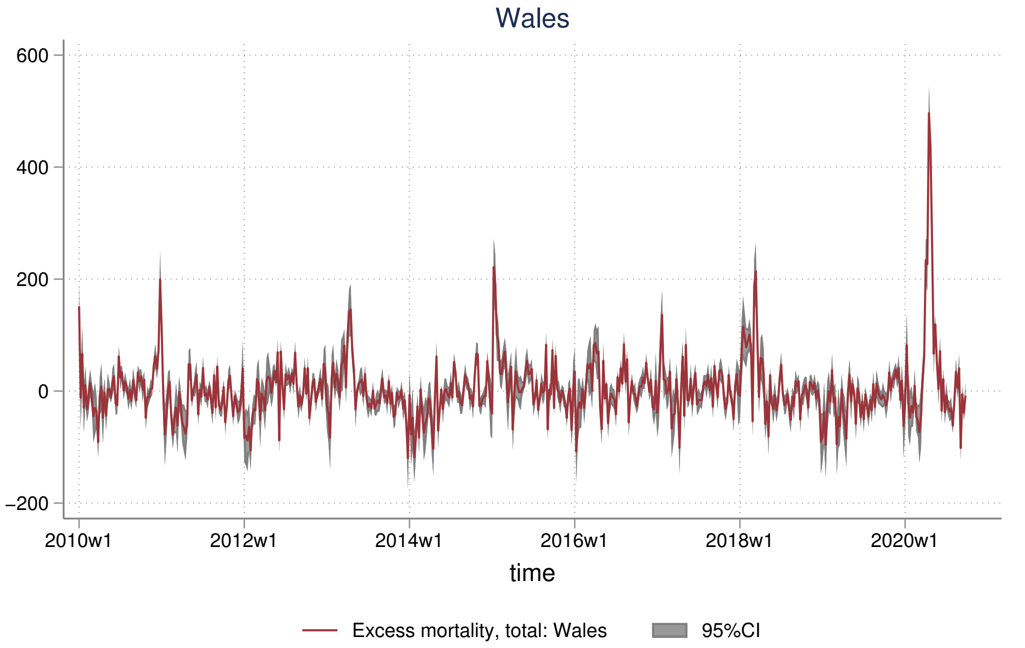

Figure 104: Wales all-cause excess deaths, from 2019 week 1

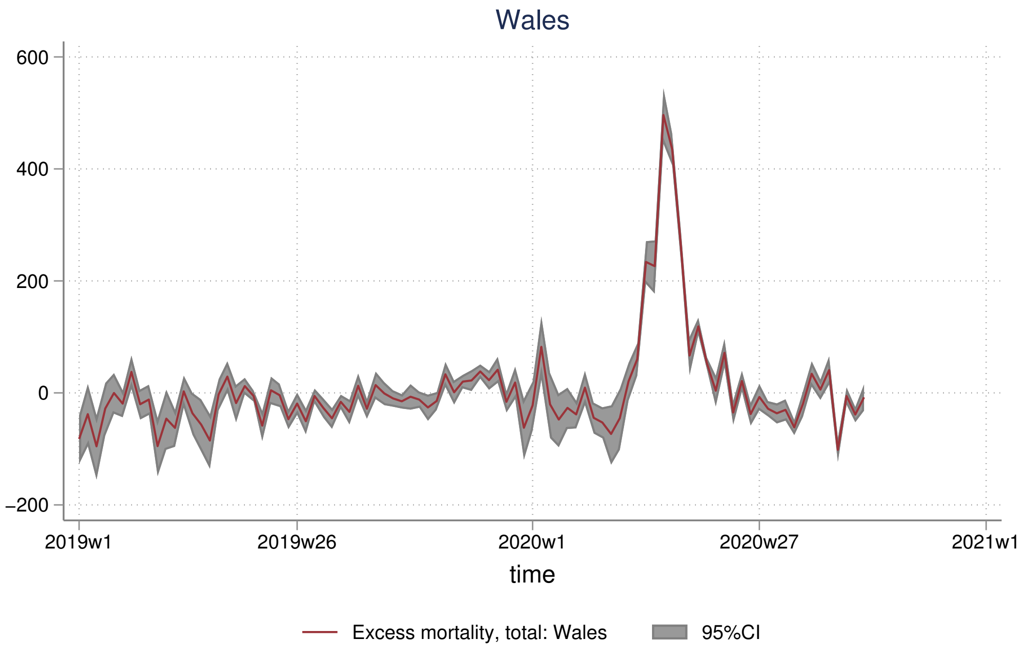

Figure 105: Wales all-cause excess deaths, from 2020 week 1

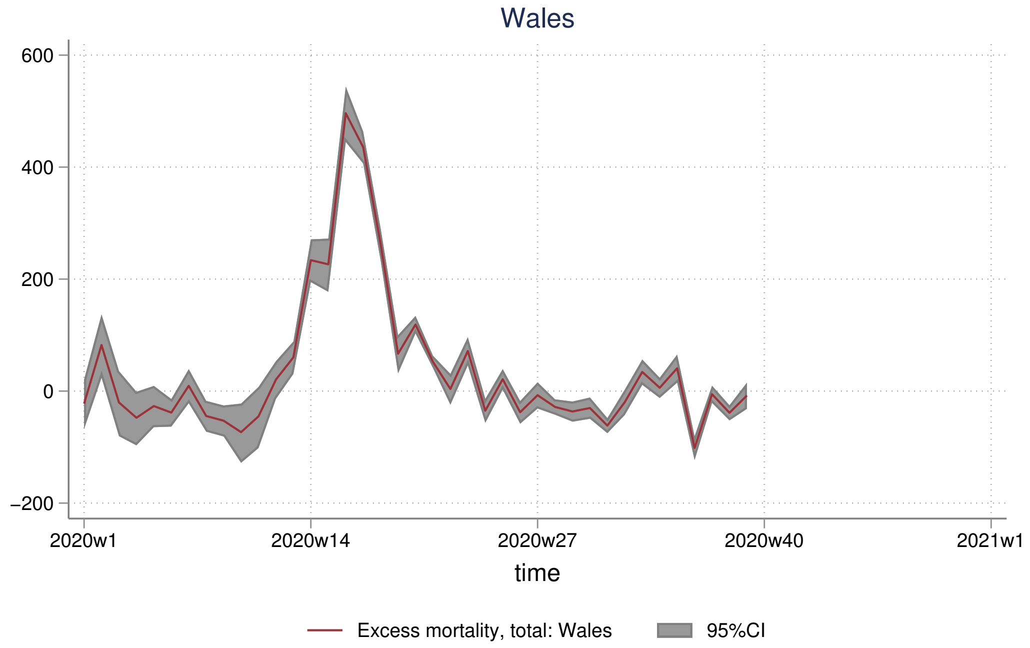

Figure 106: Wales all-cause excess deaths minus COVID19, from 2010 week 1

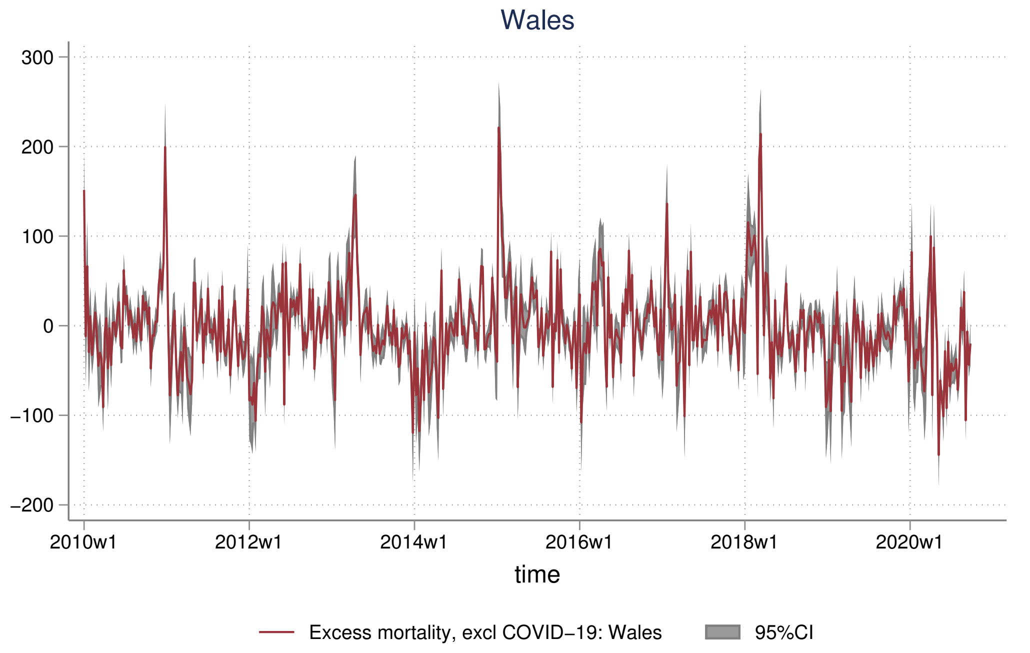

Figure 107: Wales all-cause excess deaths minus COVID19, from 2019 week 1

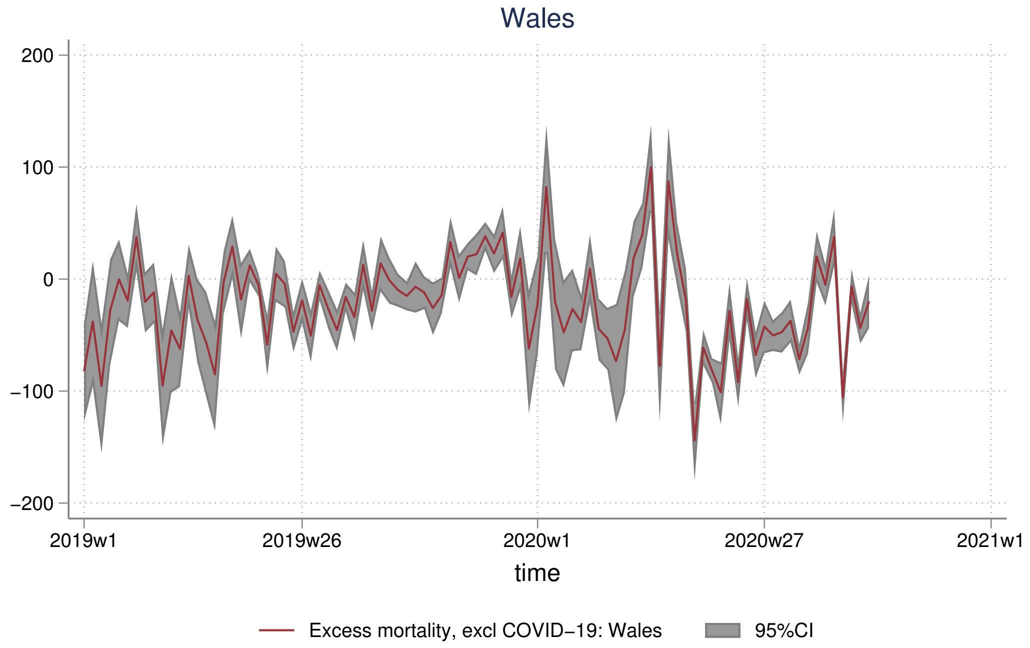

Figure 108: Wales all-cause excess deaths minus COVID19, from 2020 week 1

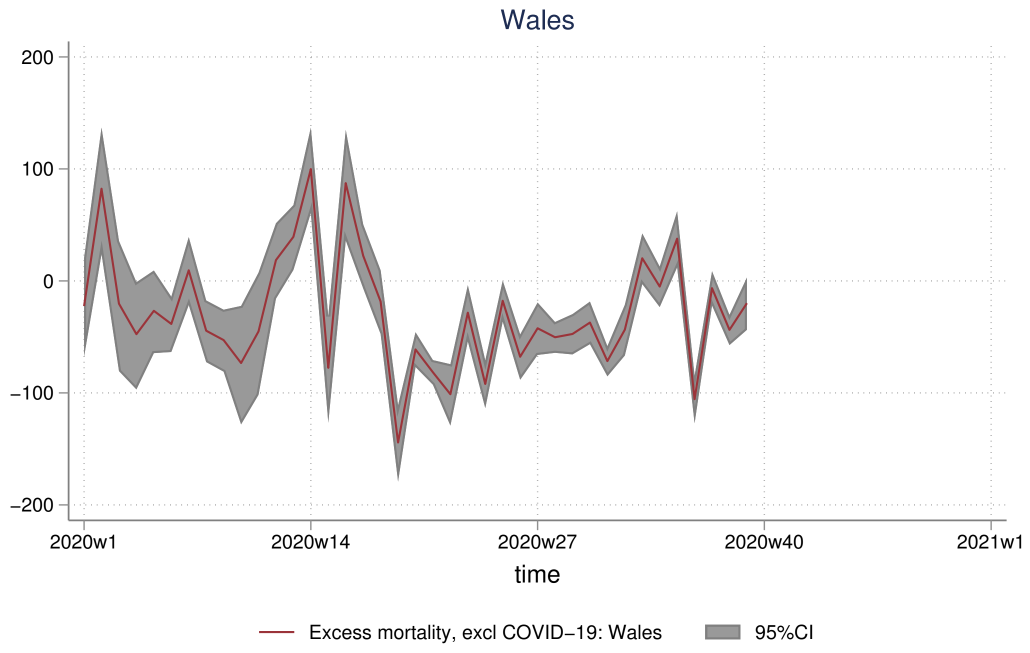

3 Age groups, people

3.1 Time trends

Figure 109: All age group mortality time trends, from 2010 week 1

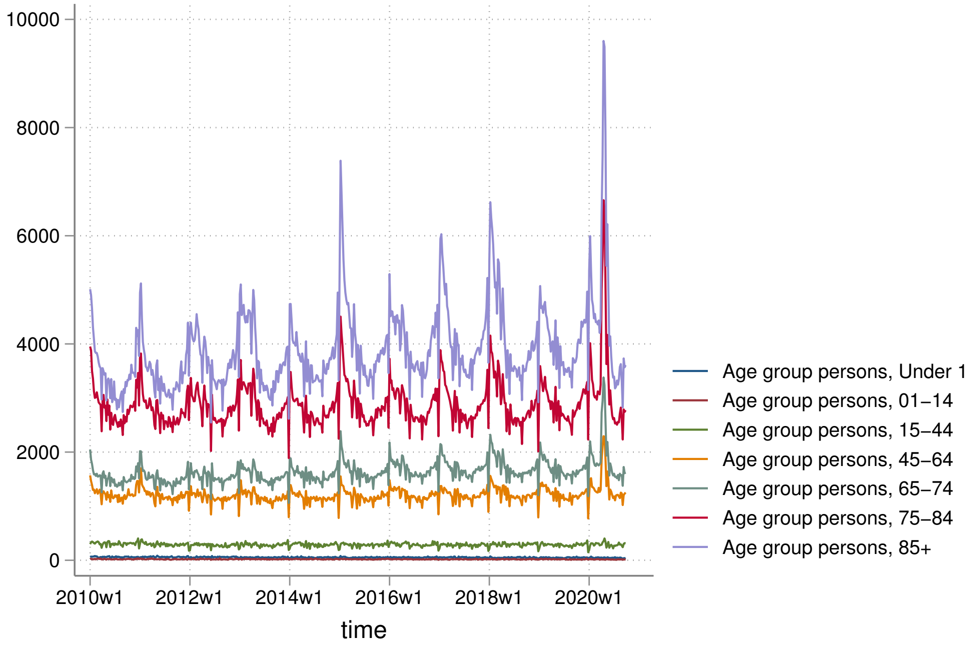

Figure 110: All age group mortality time trends, from 2019 week 1

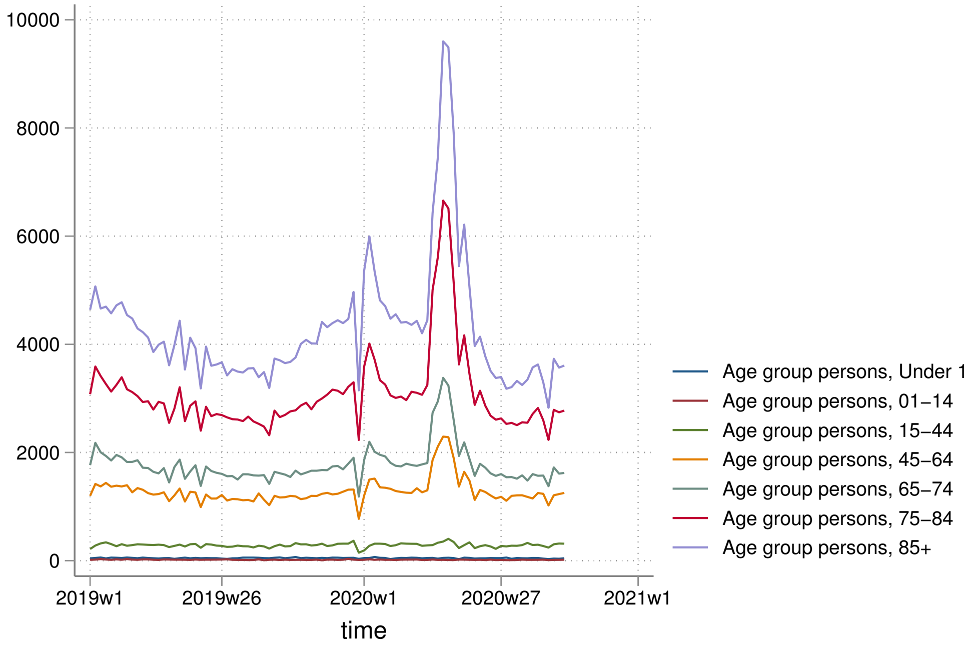

Figure 111: All age group mortality time trends, from 2020 week 1

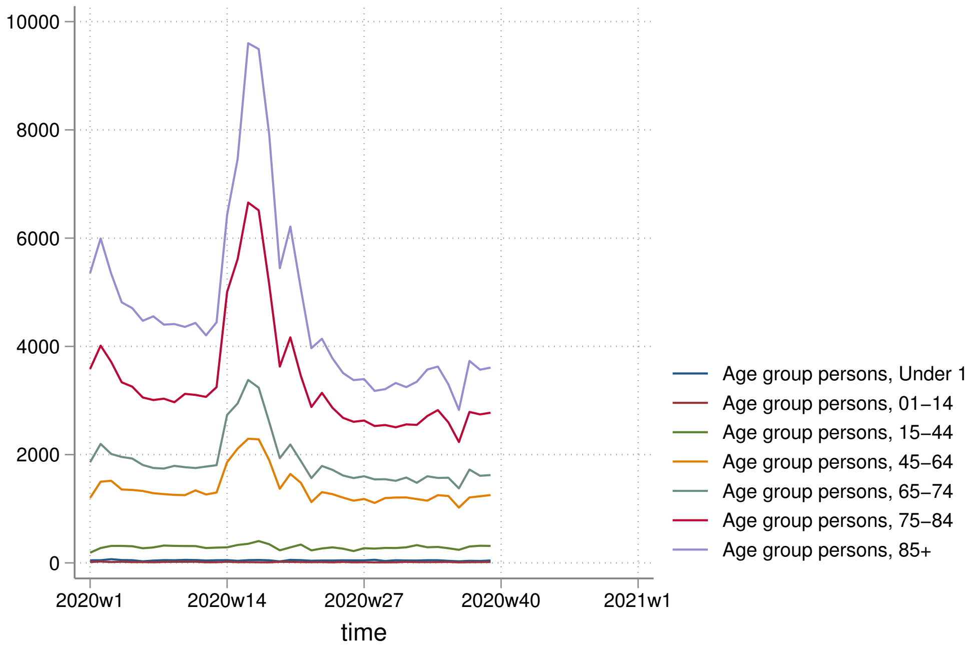

3.2 All aged under 1

Figure 112: All < 1 mortality time trend and model, from 2010 week 1

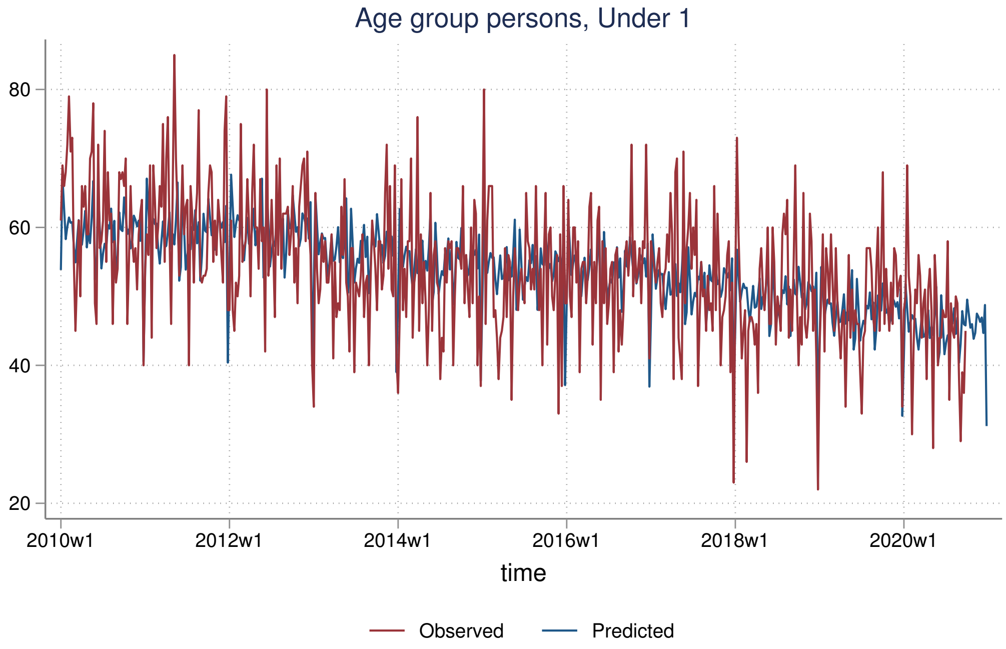

Figure 113: All < 1 mortality time trend and model, from 2019 week 1

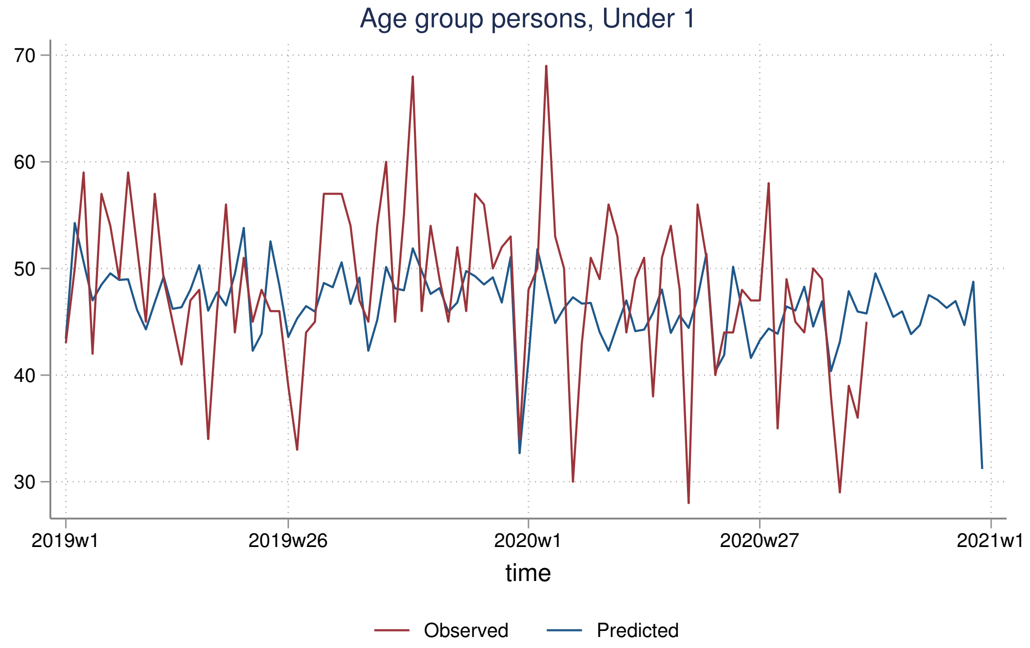

Figure 114: All < 1 mortality time trend and model, from 2020 week 1

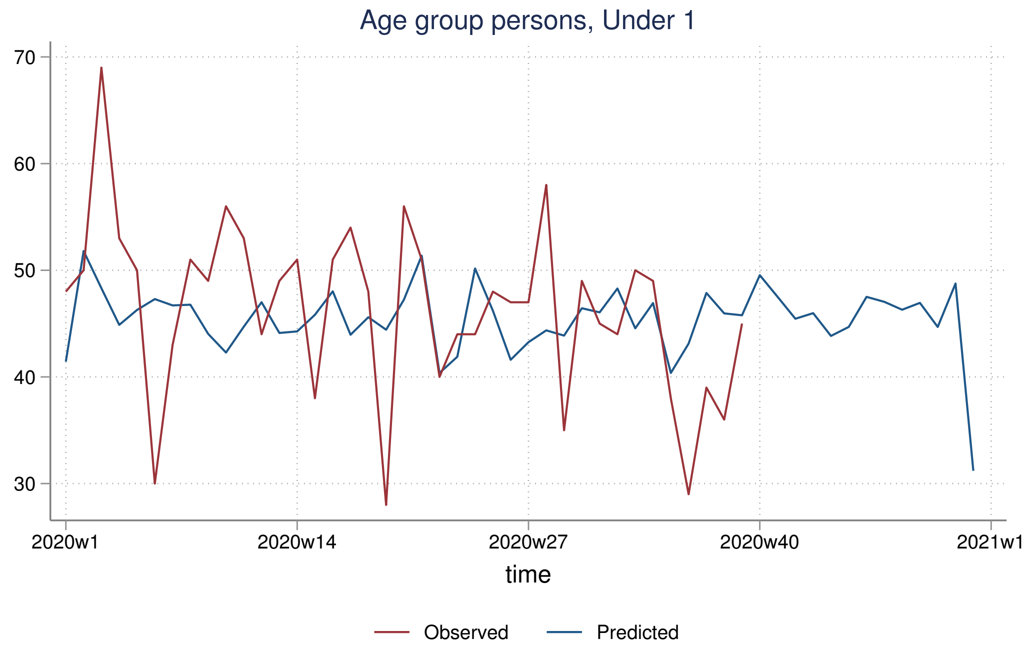

Figure 115: All < 1 all-cause excess deaths, from 2010 week 1

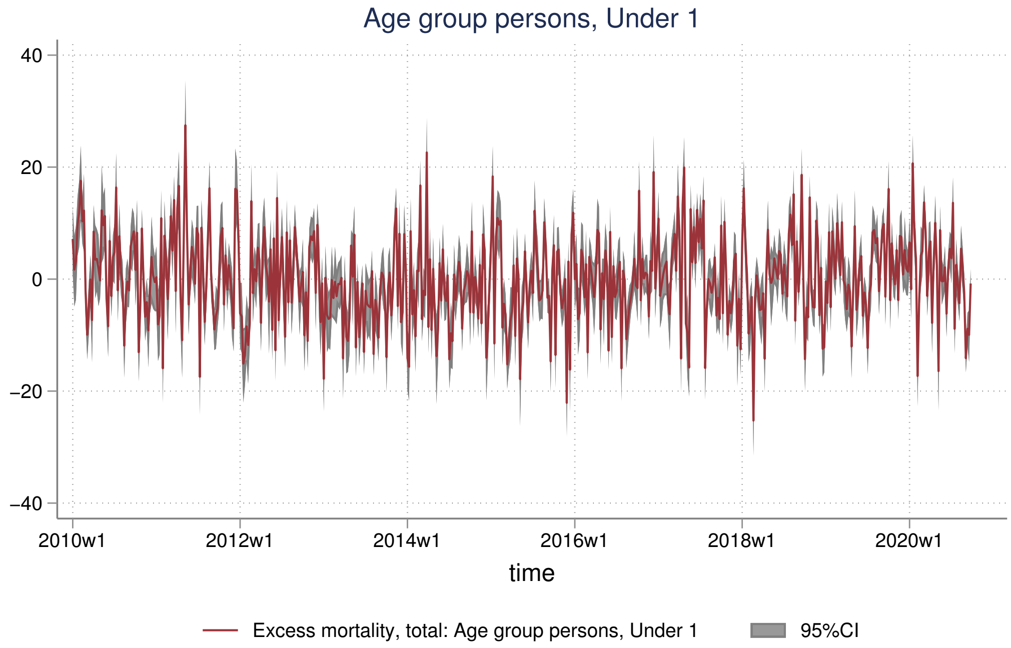

Figure 116: All < 1 all-cause excess deaths, from 2019 week 1

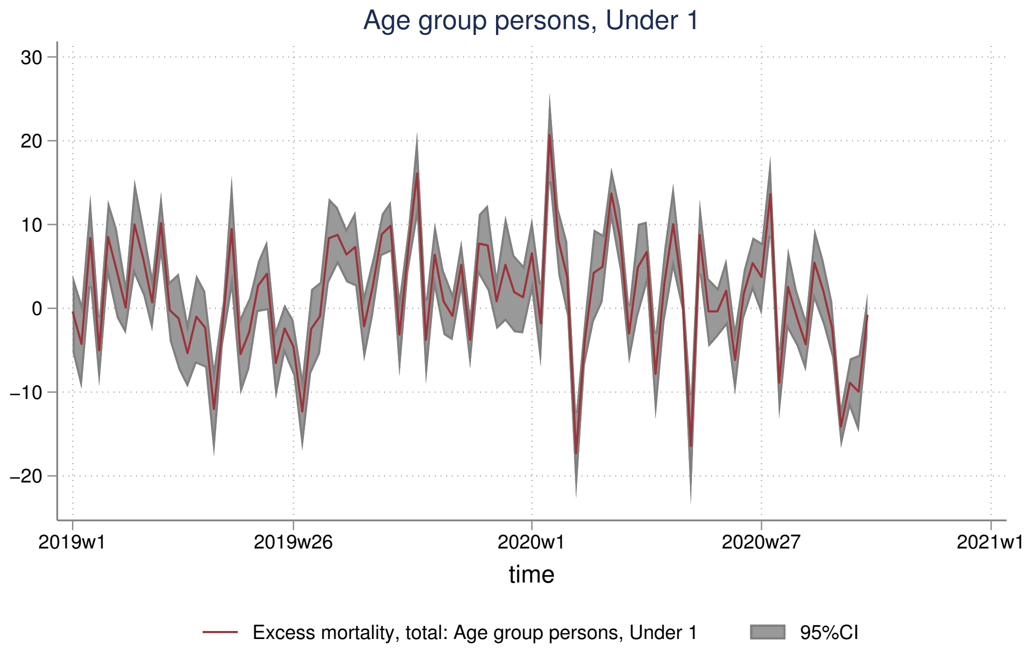

Figure 117: All < 1 all-cause excess deaths, from 2020 week 1

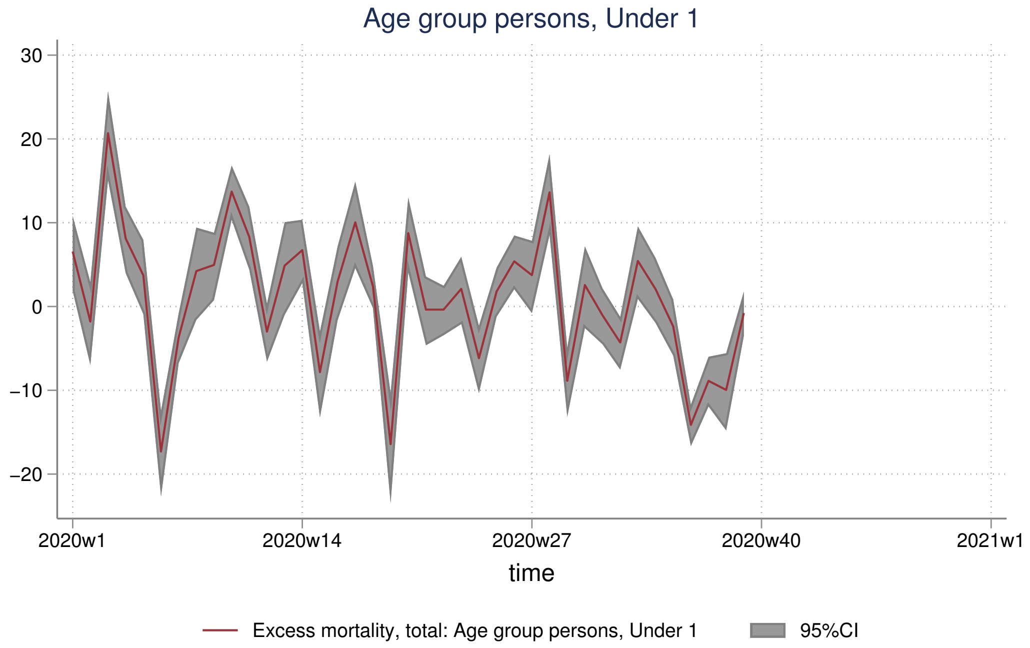

Figure 118: All < 1 all-cause excess deaths minus COVID19, from 2010 week 1

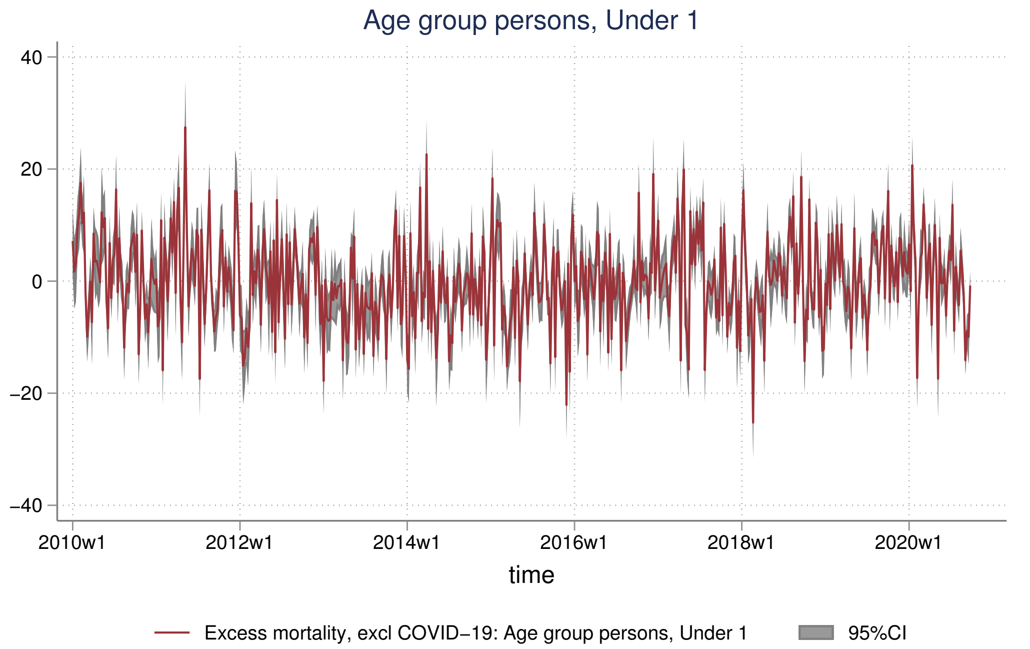

Figure 119: All < 1 all-cause excess deaths minus COVID19, from 2019 week 1

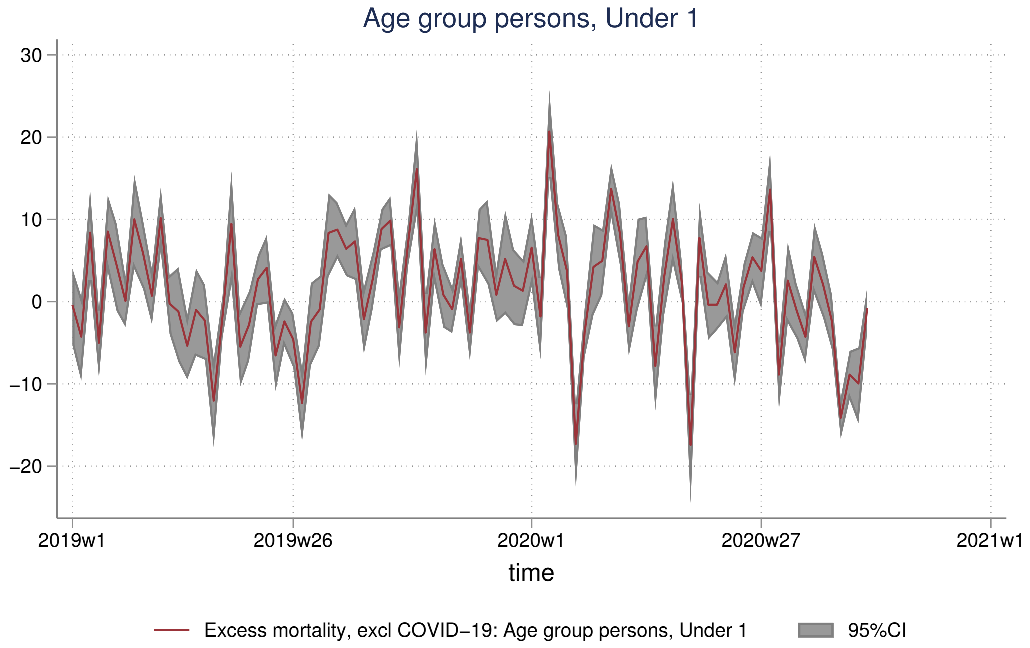

Figure 120: All < 1 all-cause excess deaths minus COVID19, from 2020 week 1

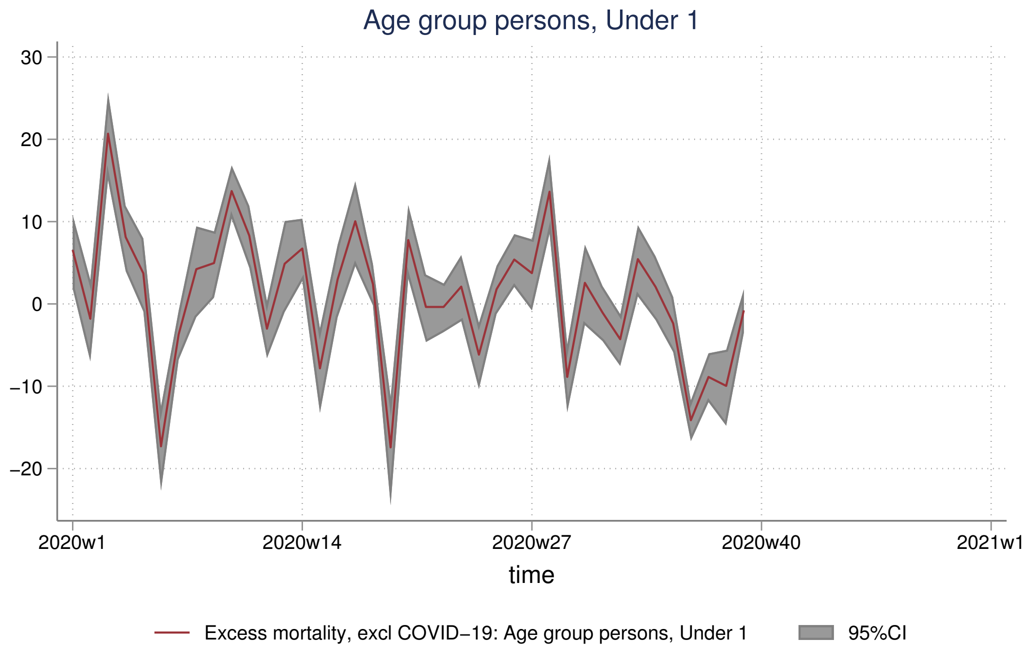

3.3 All aged 01-14

Figure 121: All 01-14 mortality time trend and model, from 2010 week 1

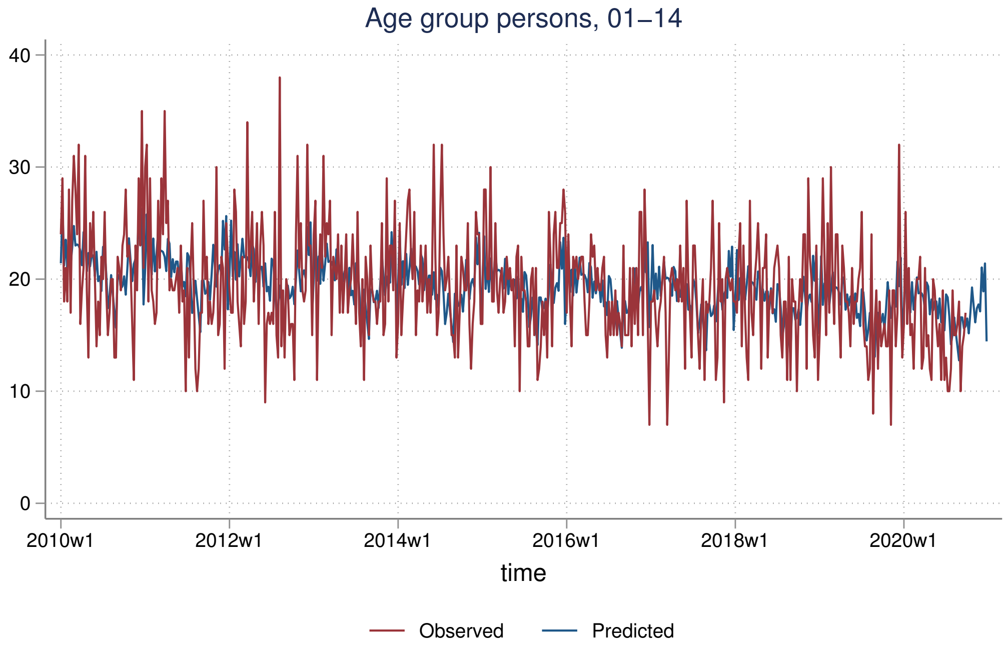

Figure 122: All 01-14 mortality time trend and model, from 2019 week 1

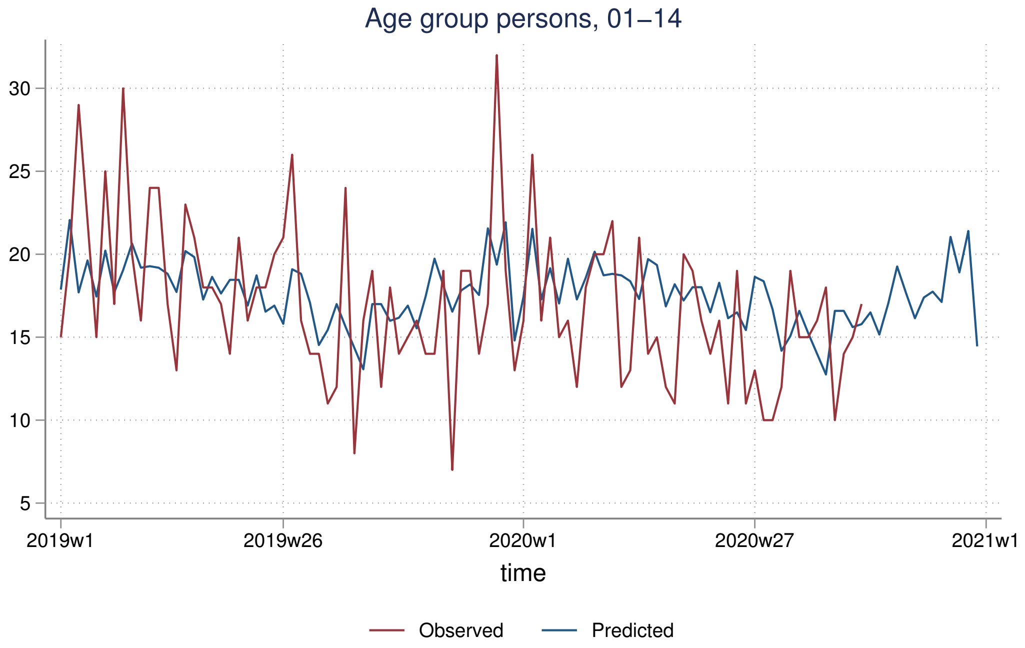

Figure 123: All 01-14 mortality time trend and model, from 2020 week 1

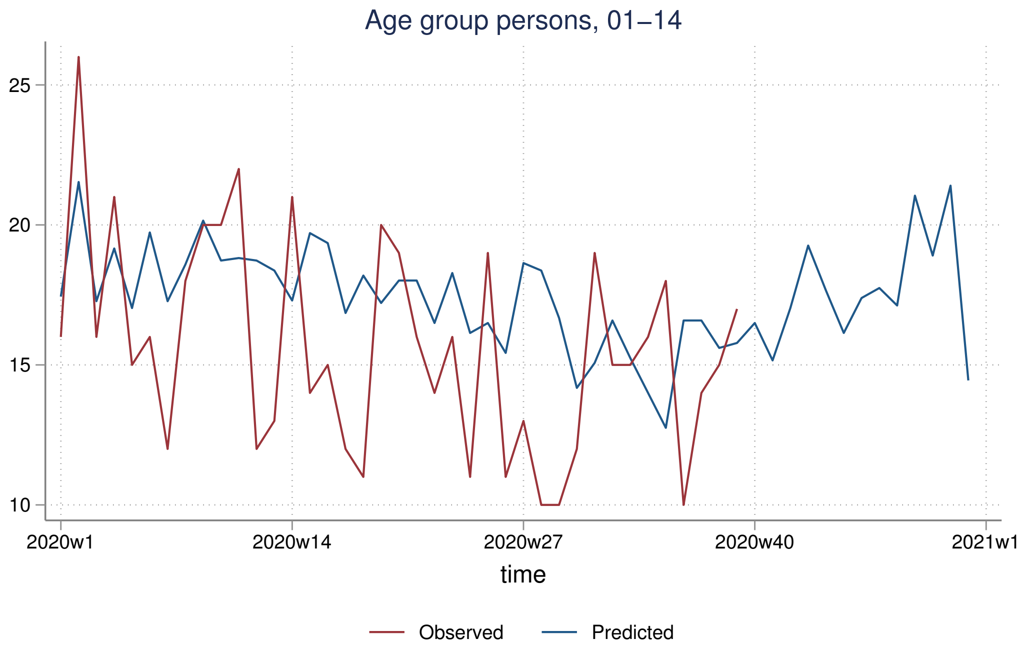

Figure 124: All 01-14 all-cause excess deaths, from 2010 week 1

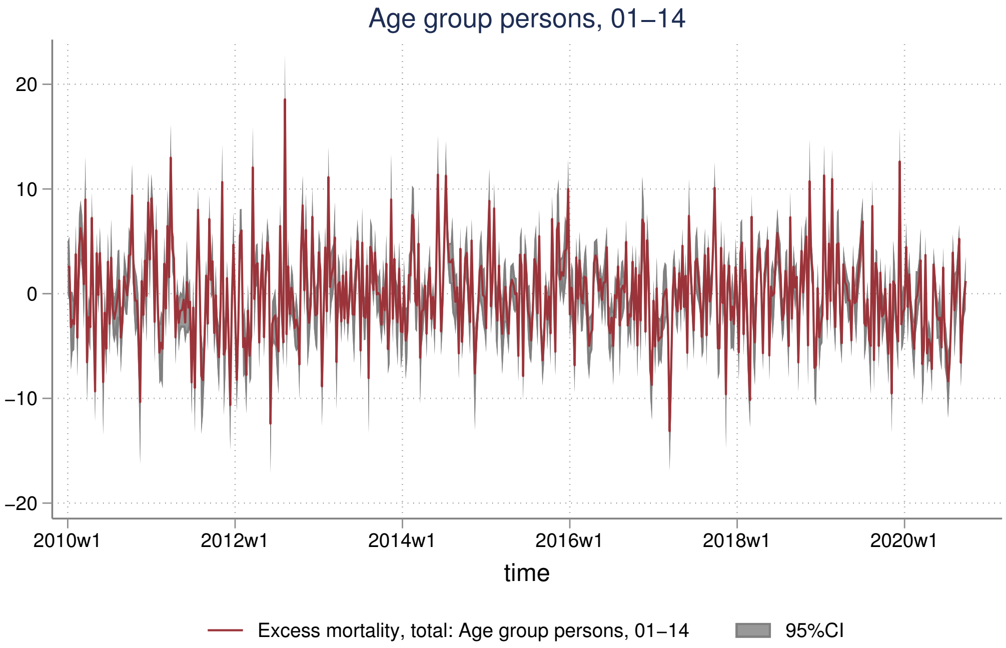

Figure 125: All 01-14 all-cause excess deaths, from 2019 week 1

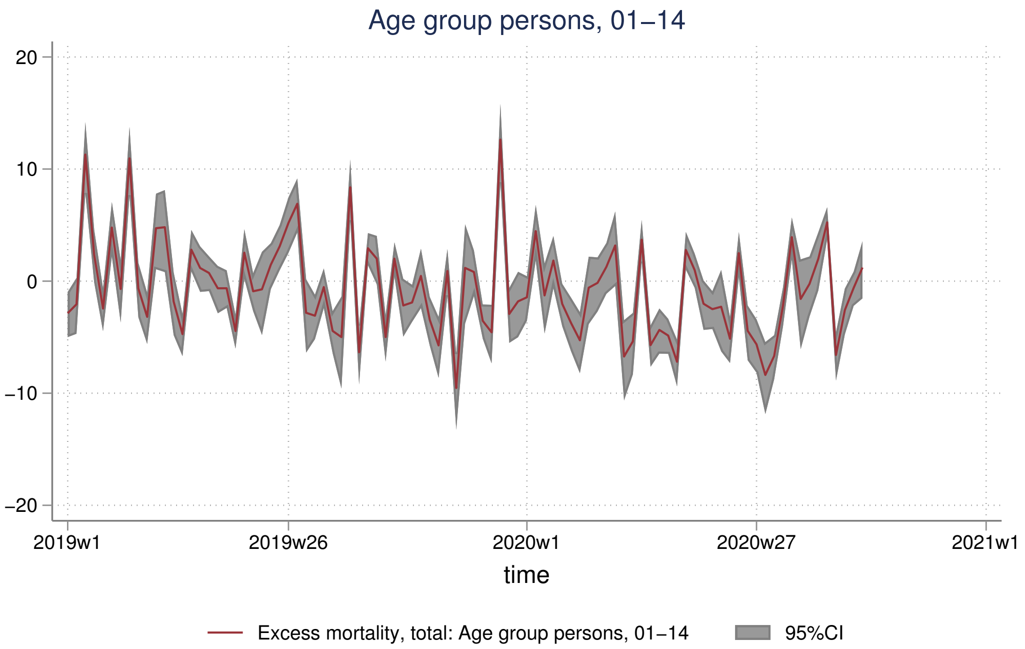

Figure 126: All 01-14 all-cause excess deaths, from 2020 week 1

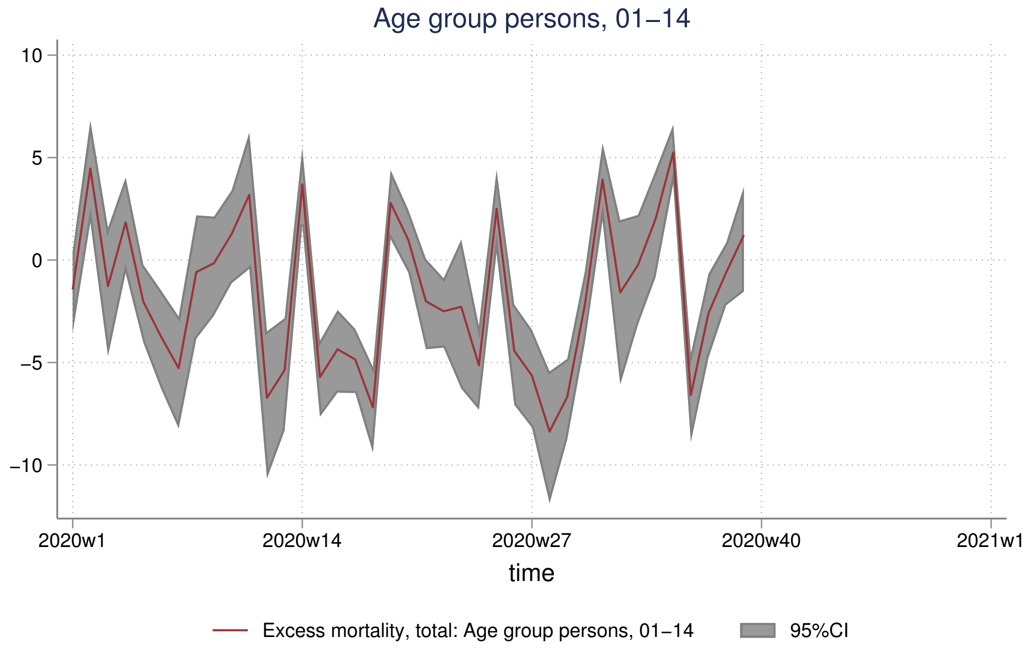

Figure 127: All 01-14 all-cause excess deaths (−COVID19), from 2010 week 1

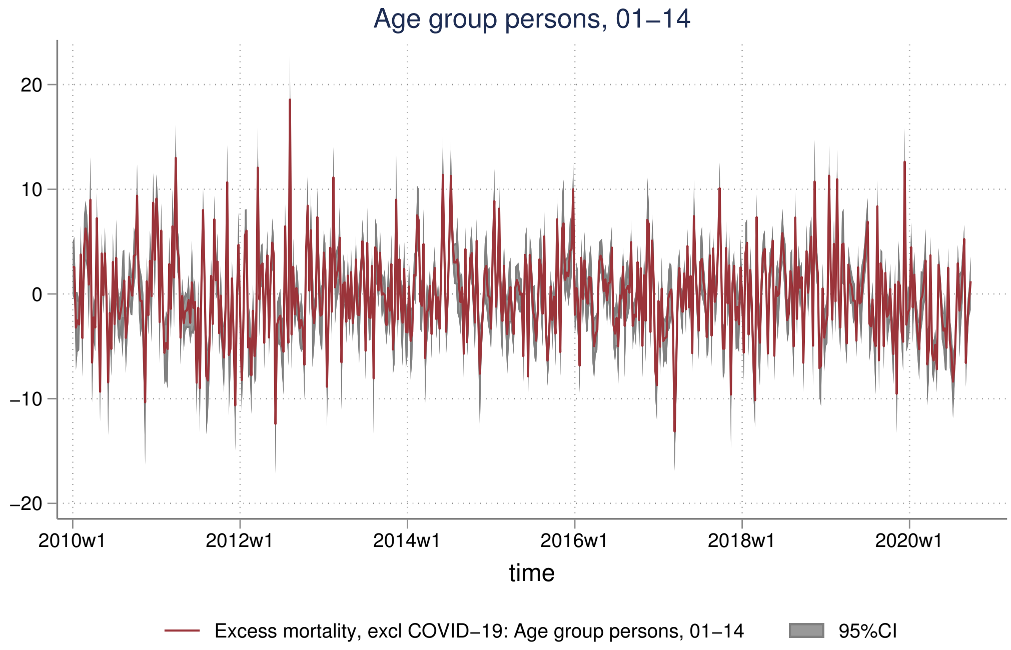

Figure 128: All 01-14 all-cause excess deaths (−COVID19), from 2019 week 1

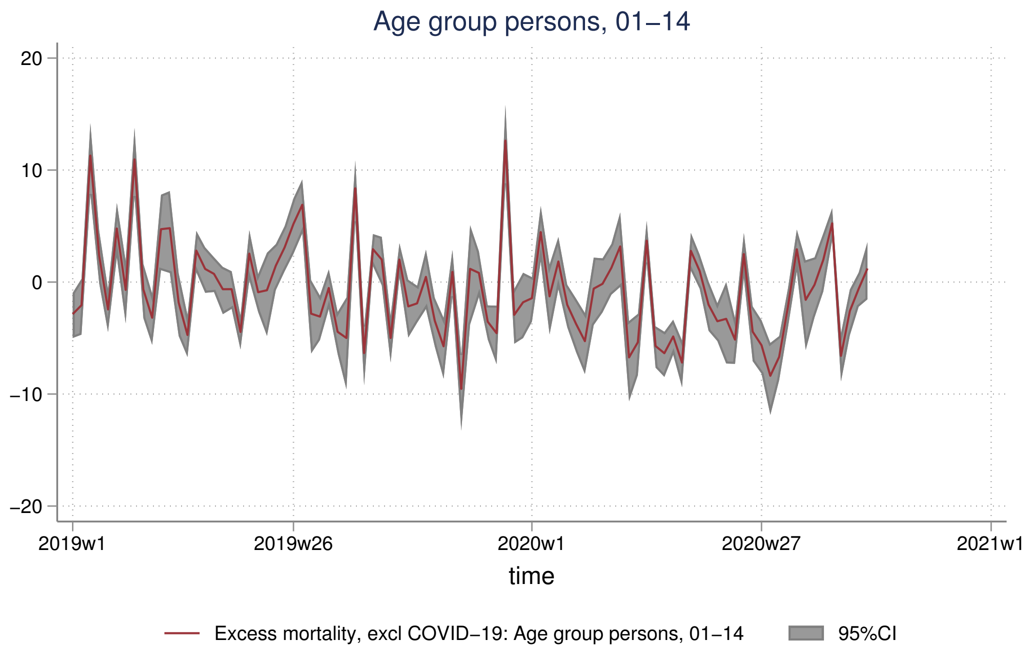

Figure 129: All 01-14 all-cause excess deaths (−COVID19), from 2020 week 1

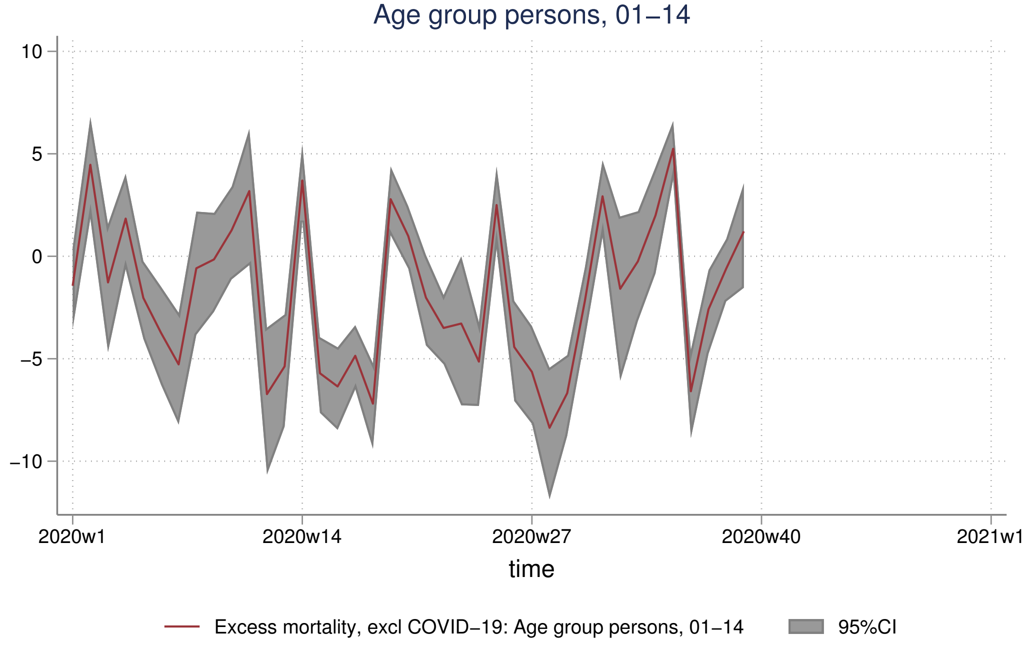

3.4 All aged 15-44

Figure 130: All 15-44 mortality time trend and model, from 2010 week 1

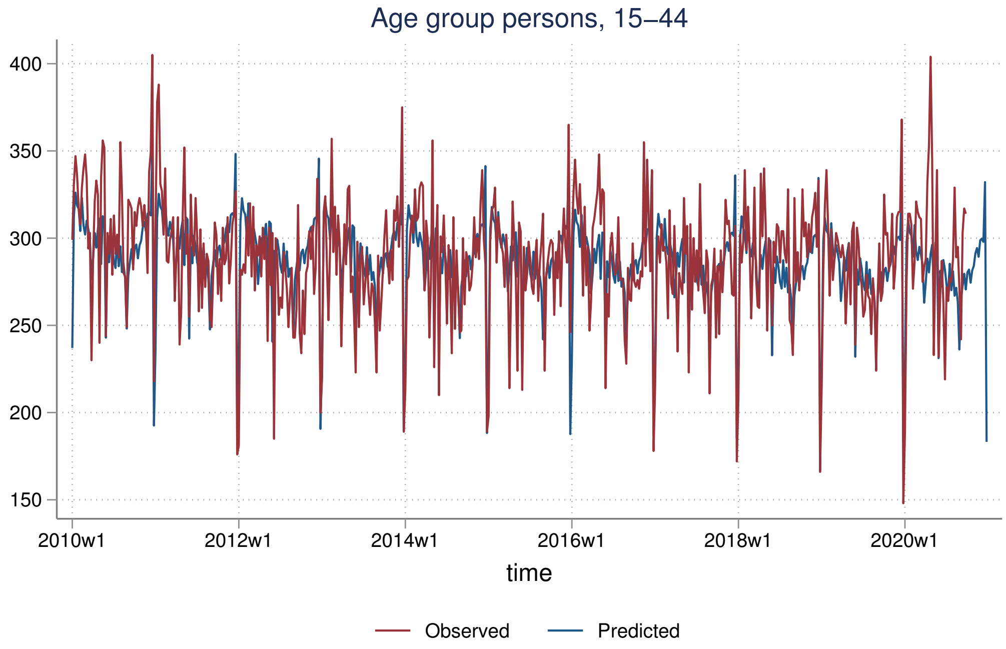

Figure 131: All 15-44 mortality time trend and model, from 2019 week 1

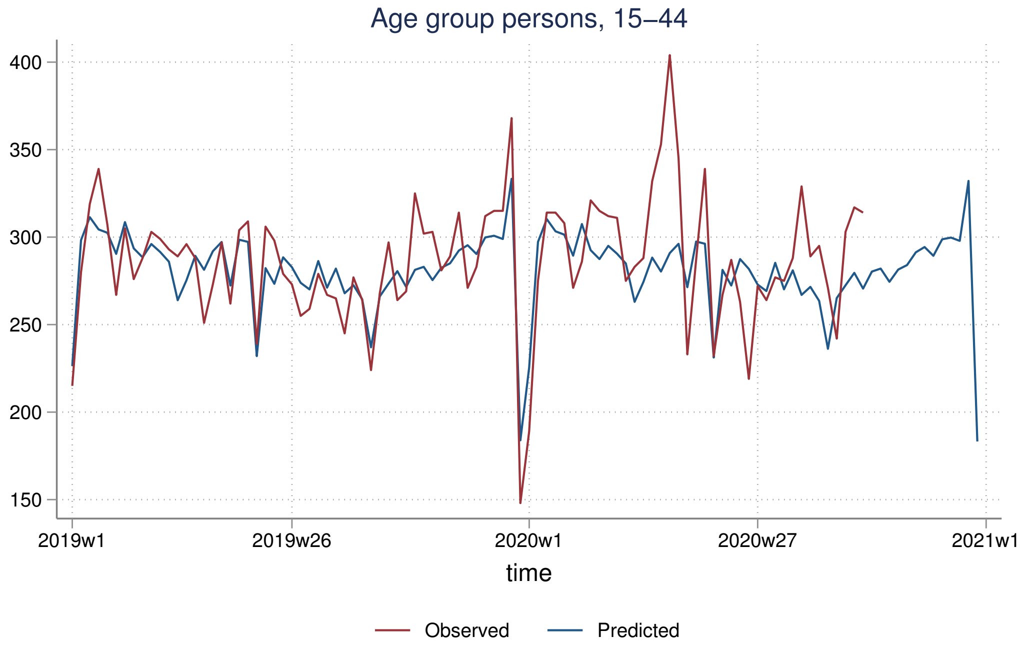

Figure 132: All 15-44 mortality time trend and model, from 2020 week 1

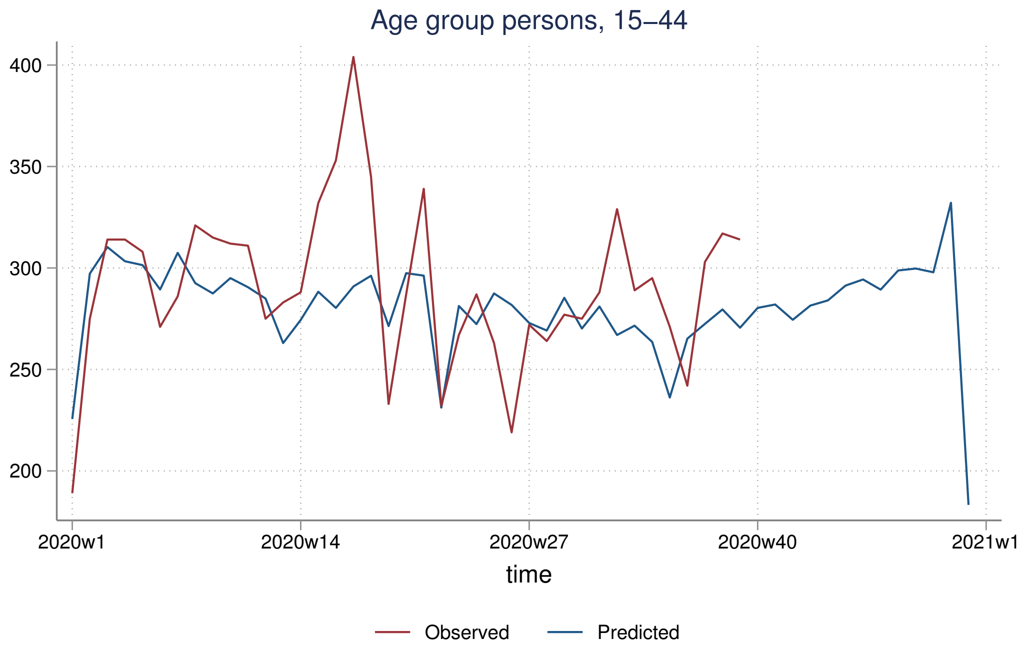

Figure 133: All 15-44 all-cause excess deaths, from 2010 week 1

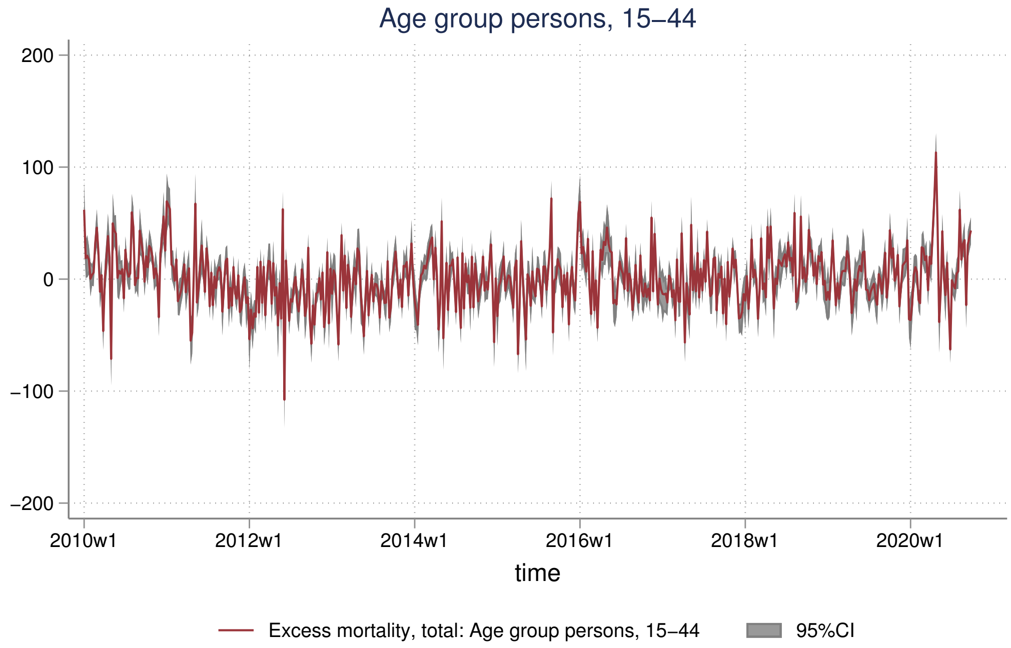

Figure 134: All 15-44 all-cause excess deaths, from 2019 week 1

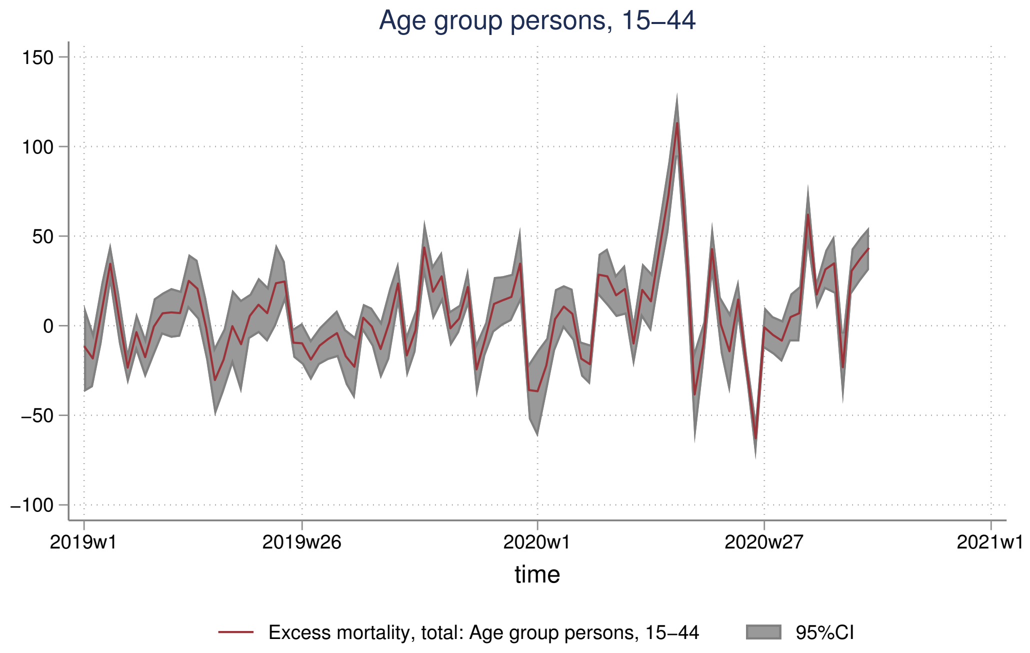

Figure 135: All 15-44 all-cause excess deaths, from 2020 week 1

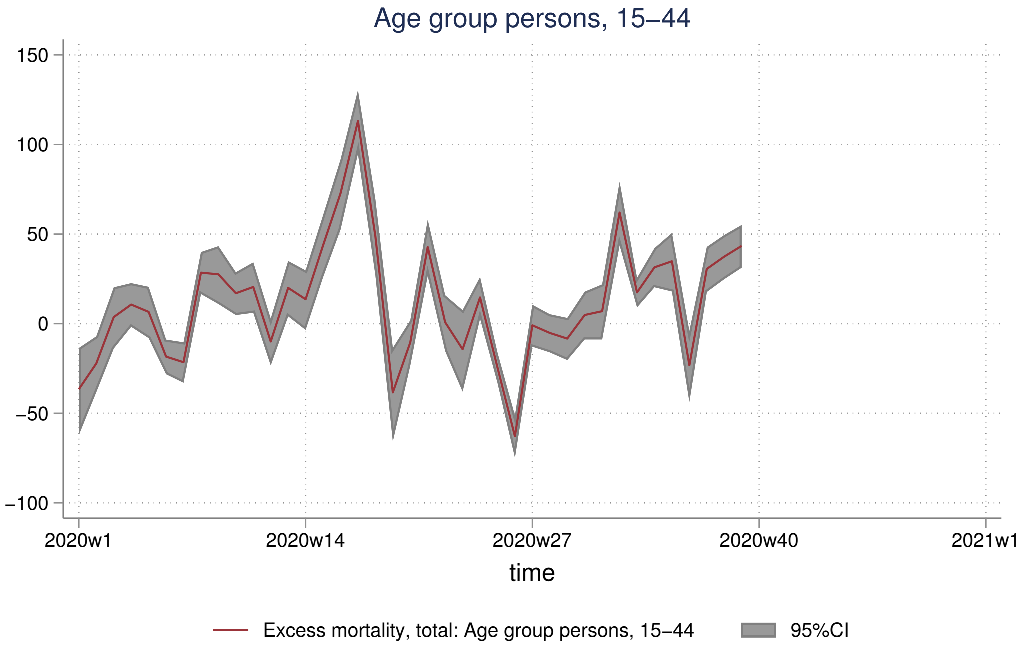

Figure 136: All 15-44 all-cause excess deaths (−COVID19), from 2010 week 1

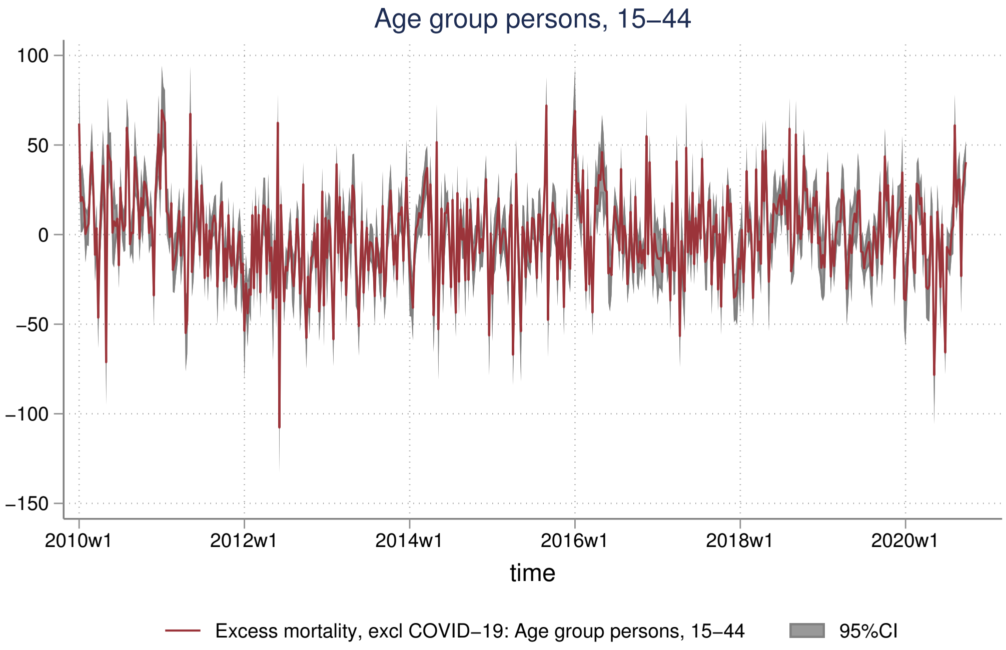

Figure 137: All 15-44 all-cause excess deaths (−COVID19), from 2019 week 1

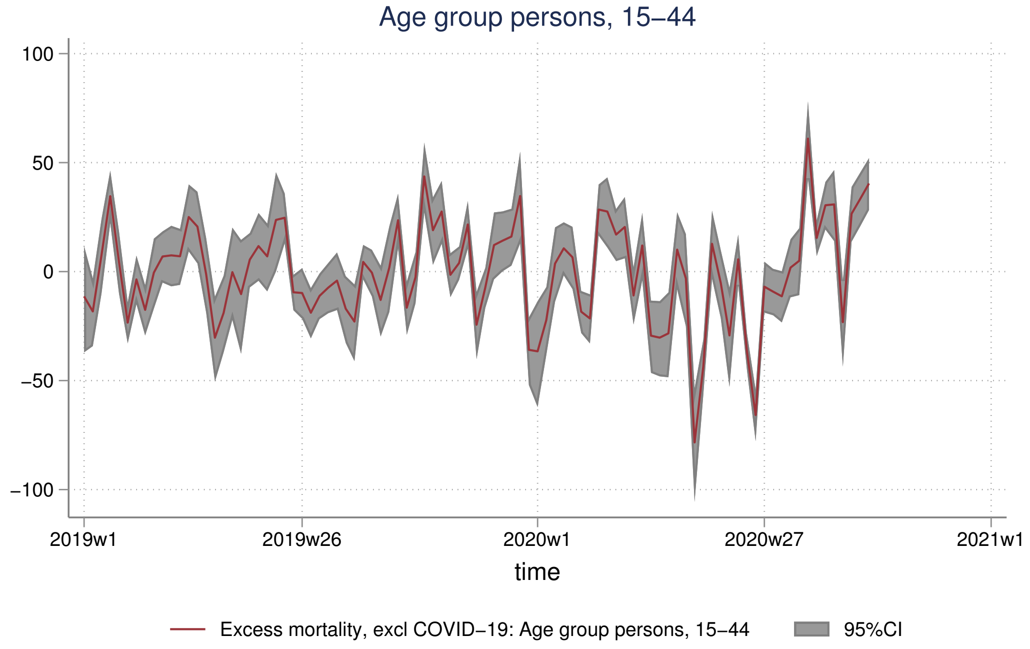

Figure 138: All 15-44 all-cause excess deaths (−COVID19), from 2020 week 1

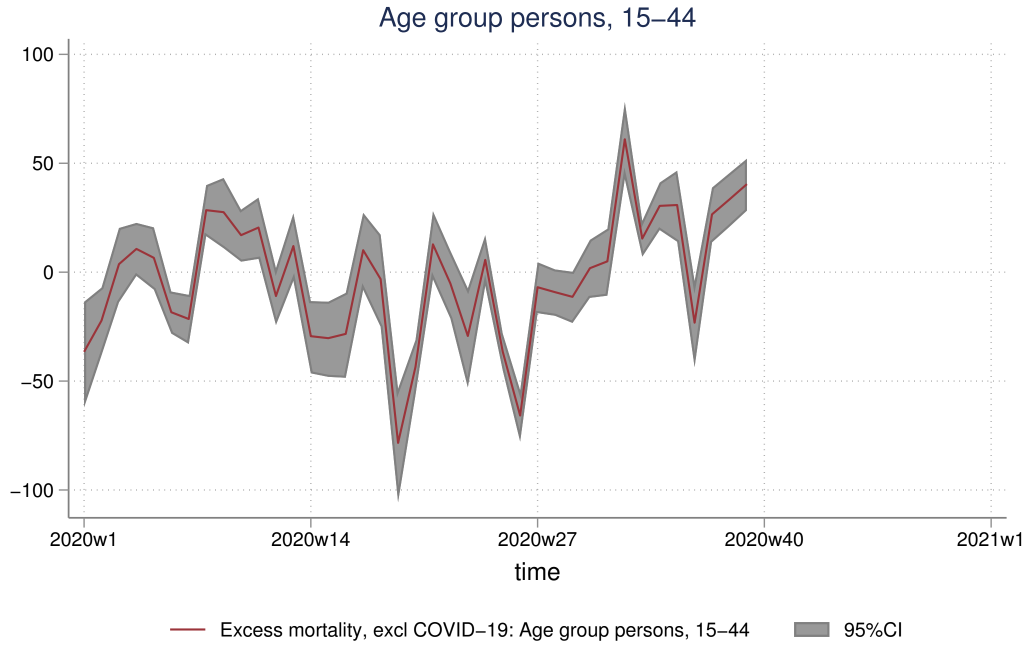

3.5 All aged 45-64

Figure 139: All 45-64 mortality time trend and model, from 2010 week 1

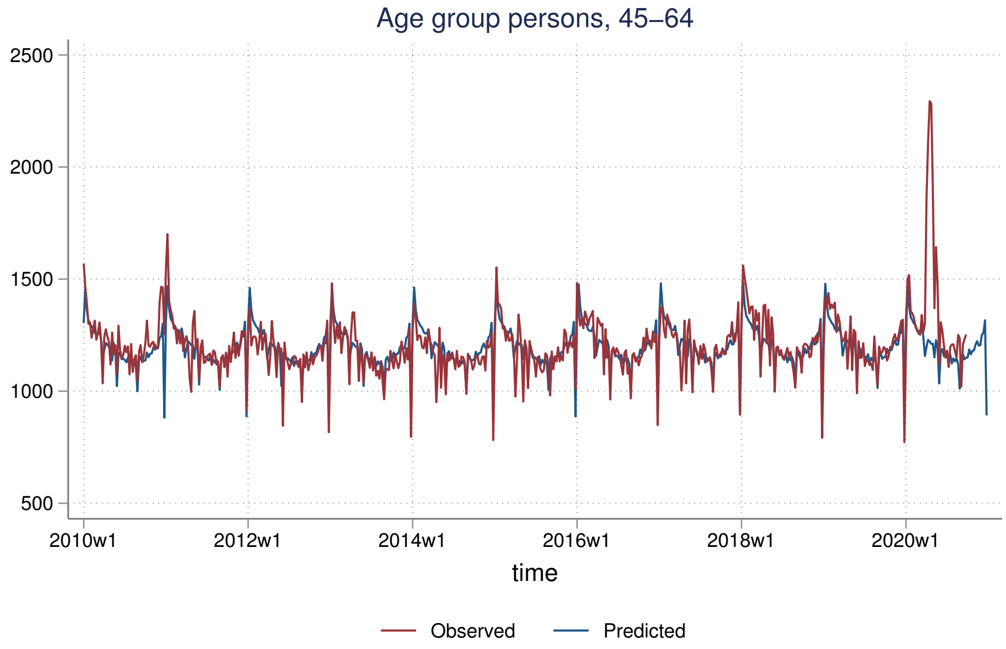

Figure 140: All 45-64 mortality time trend and model, from 2019 week 1

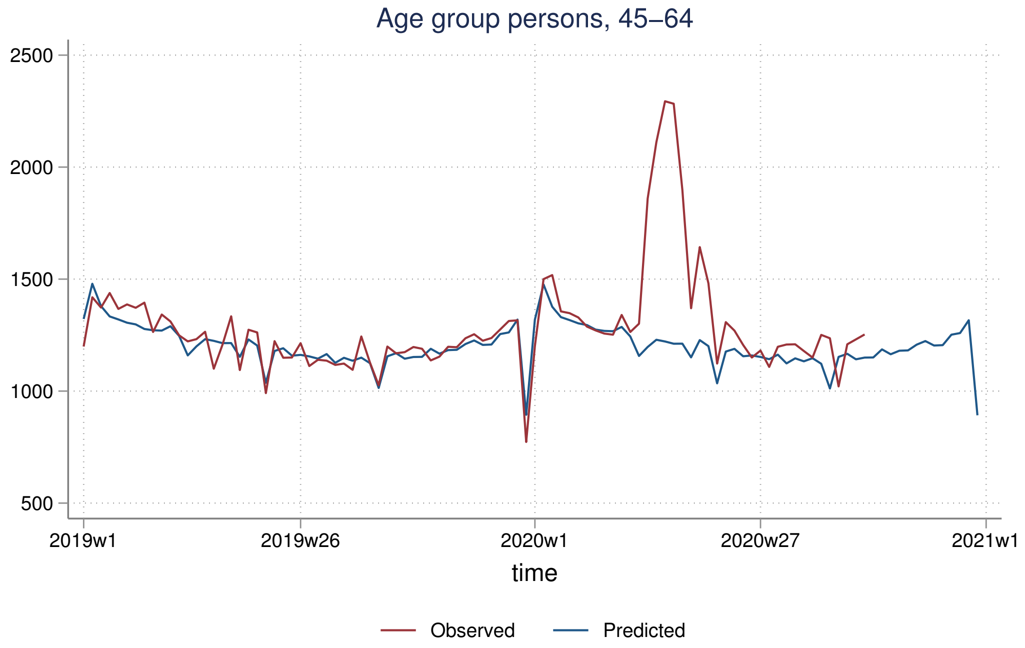

Figure 141: All 45-64 mortality time trend and model, from 2020 week 1

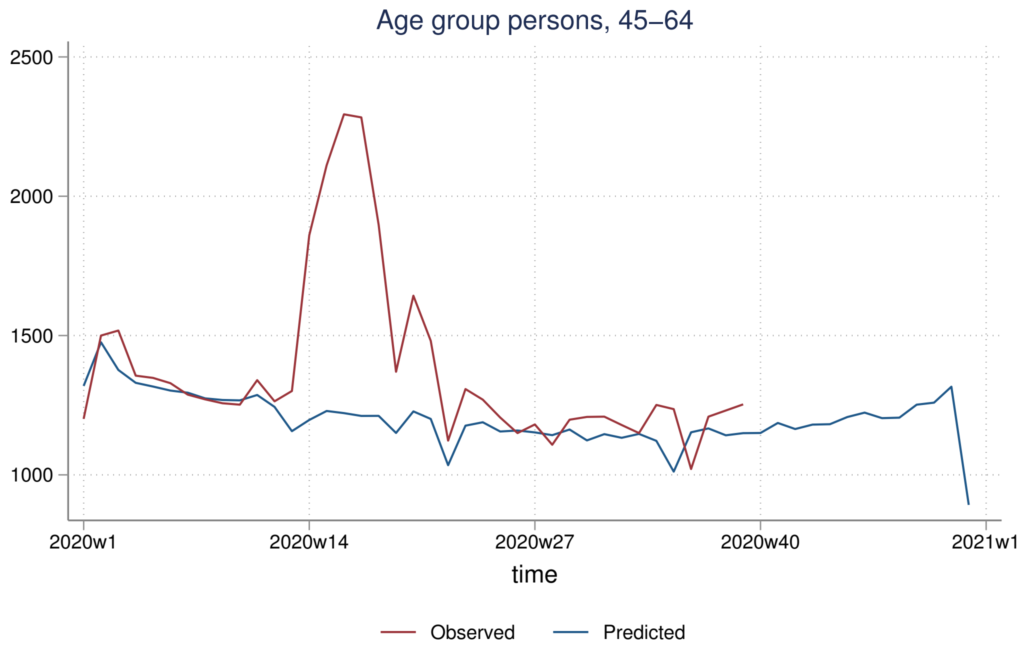

Figure 142: All 45-64 all-cause excess deaths, from 2010 week 1

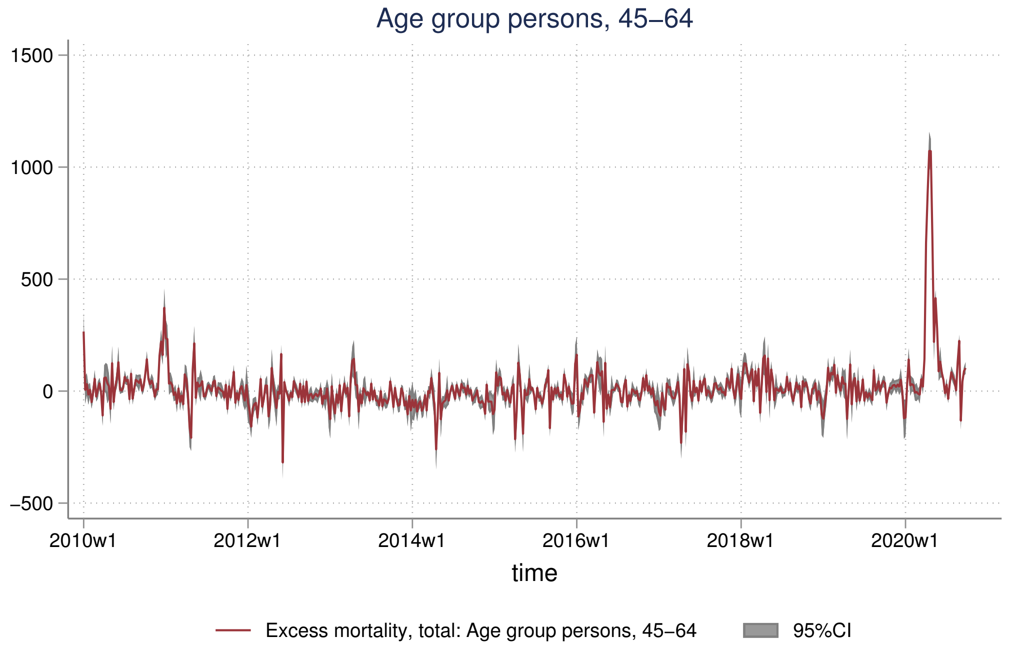

Figure 143: All 45-64 all-cause excess deaths, from 2019 week 1

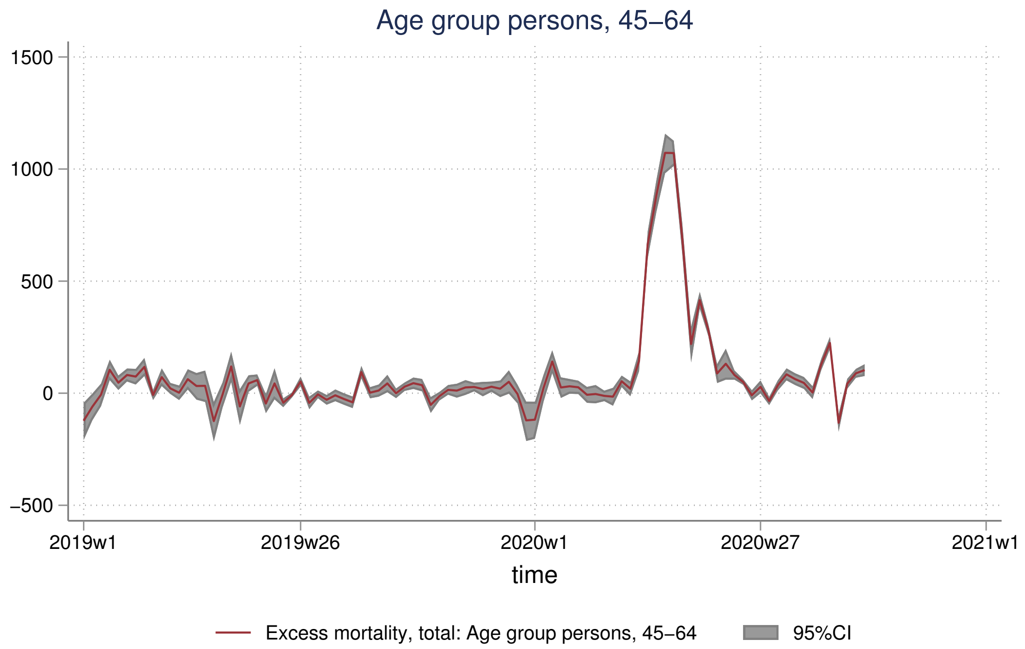

Figure 144: All 45-64 all-cause excess deaths, from 2020 week 1

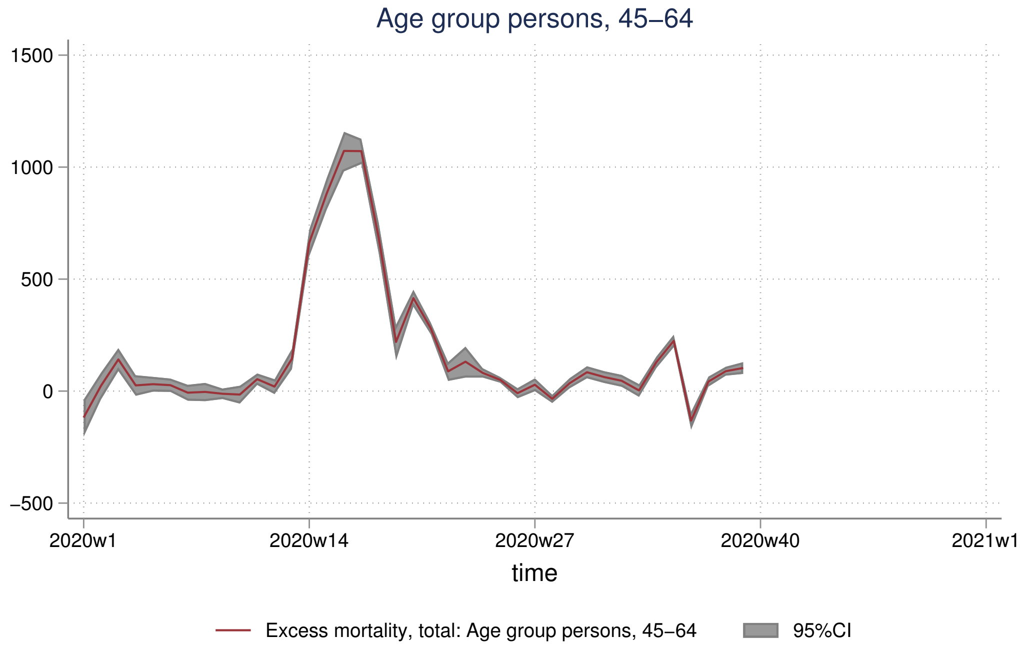

Figure 145: All 45-64 all-cause excess deaths (−COVID19), from 2010 week 1

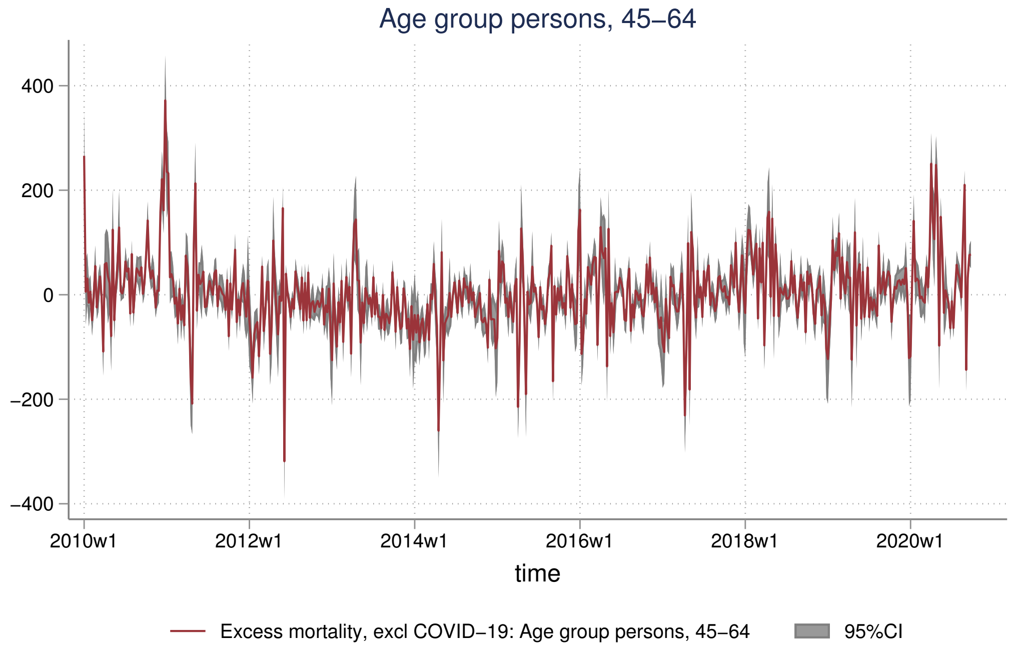

Figure 146: All 45-64 all-cause excess deaths (−COVID19), from 2019 week 1

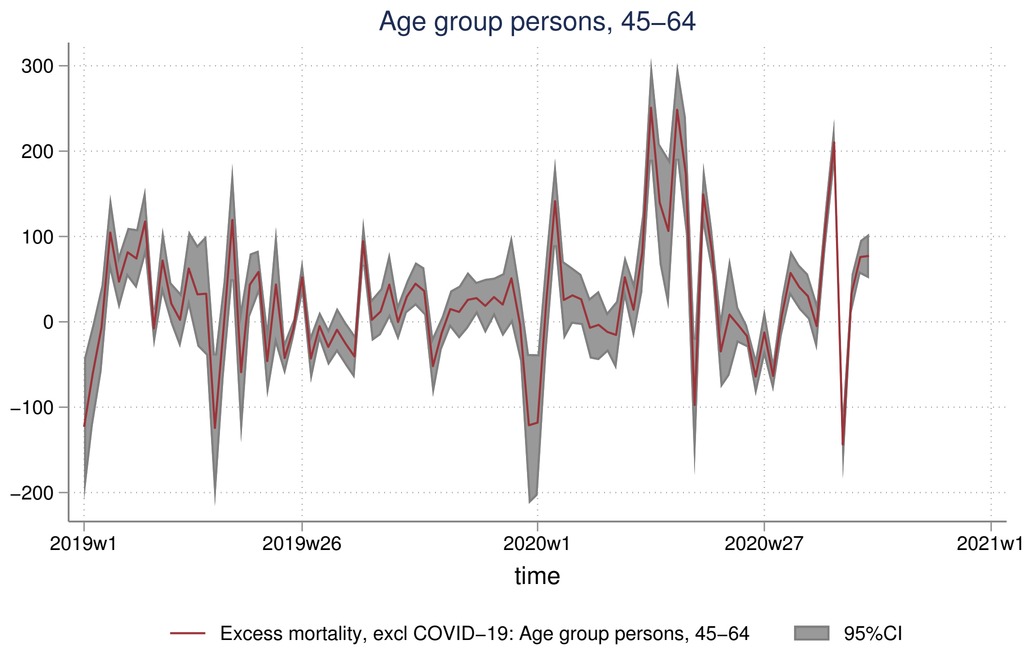

Figure 147: All 45-64 all-cause excess deaths (−COVID19), from 2020 week 1

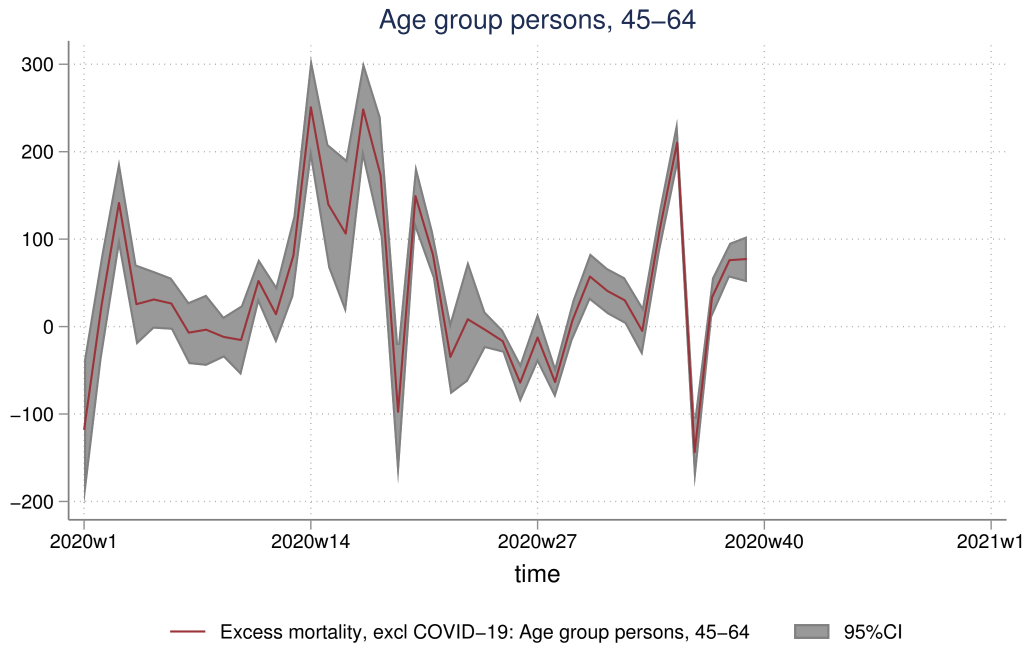

3.6 All aged 65-74

Figure 148: All 65-74 mortality time trend and model, from 2010 week 1

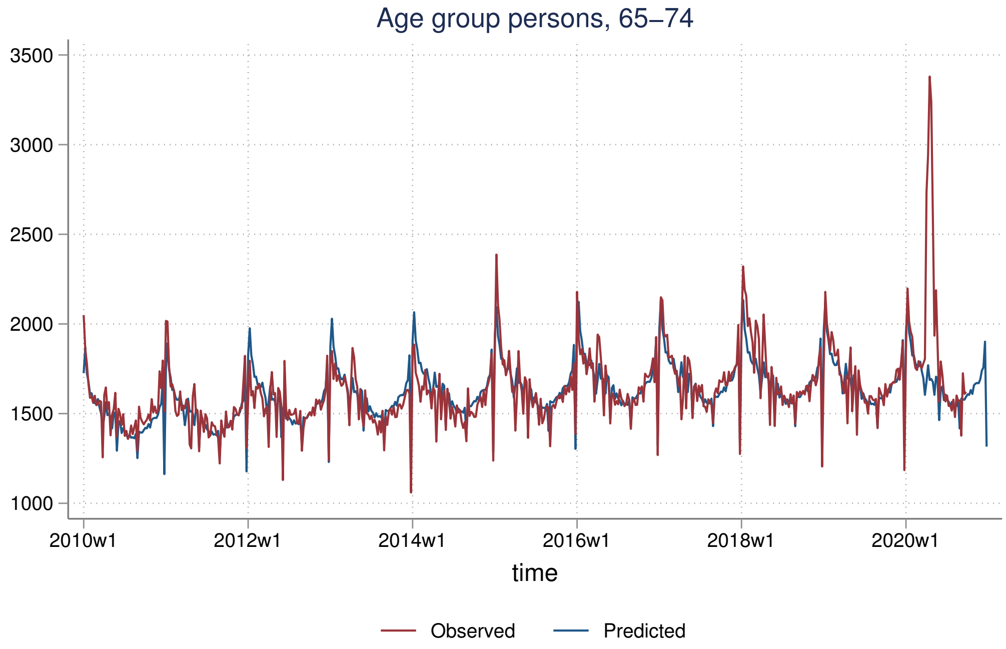

Figure 149: All 65-74 mortality time trend and model, from 2019 week 1

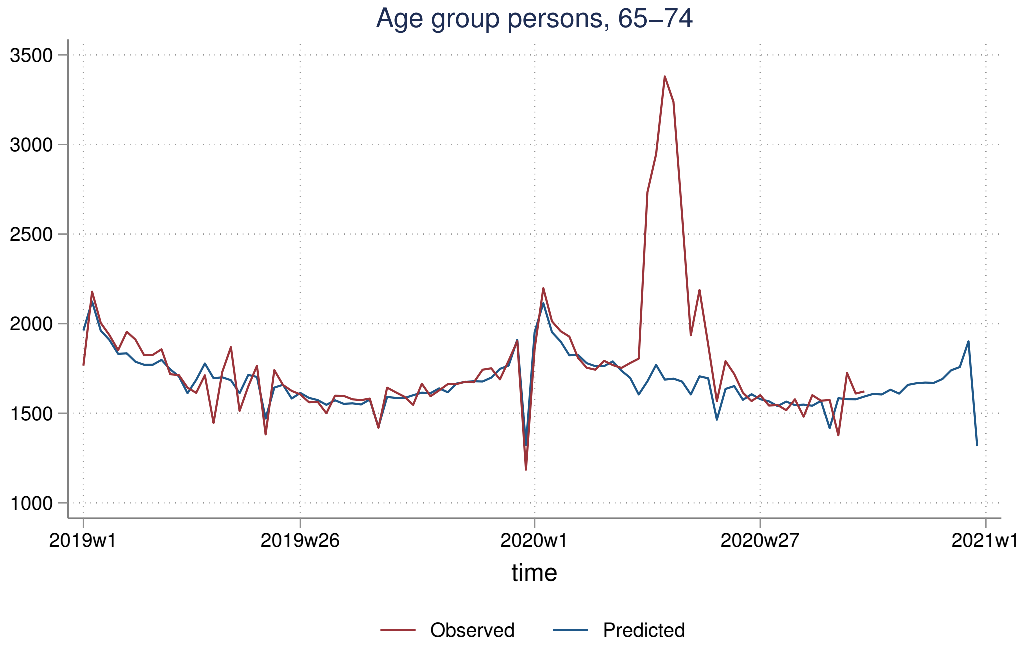

Figure 150: All 65-74 mortality time trend and model, from 2020 week 1

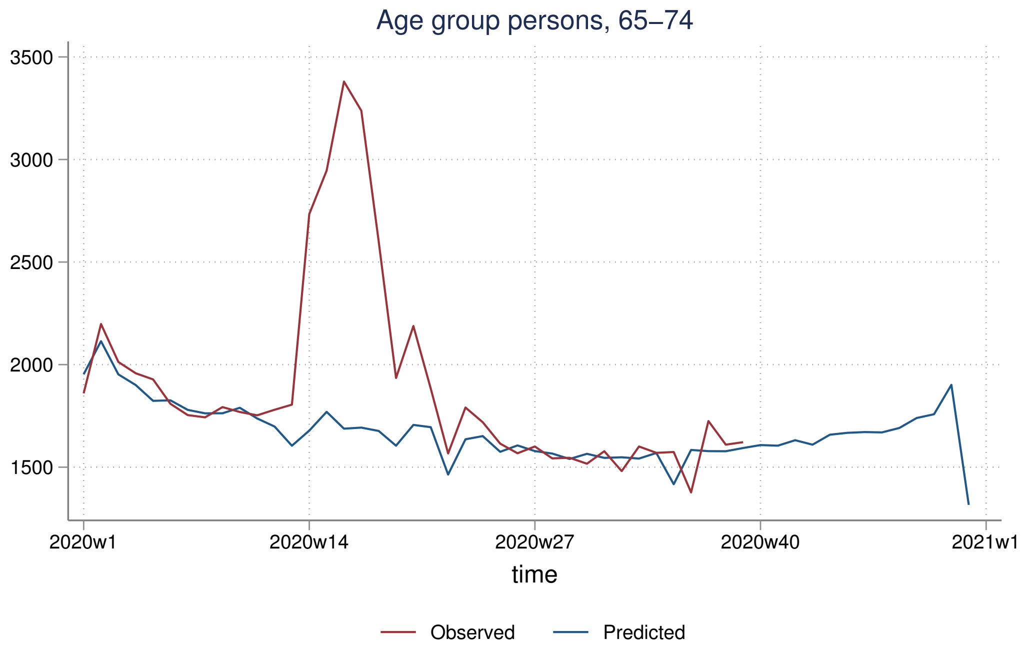

Figure 151: All 65-74 all-cause excess deaths, from 2010 week 1

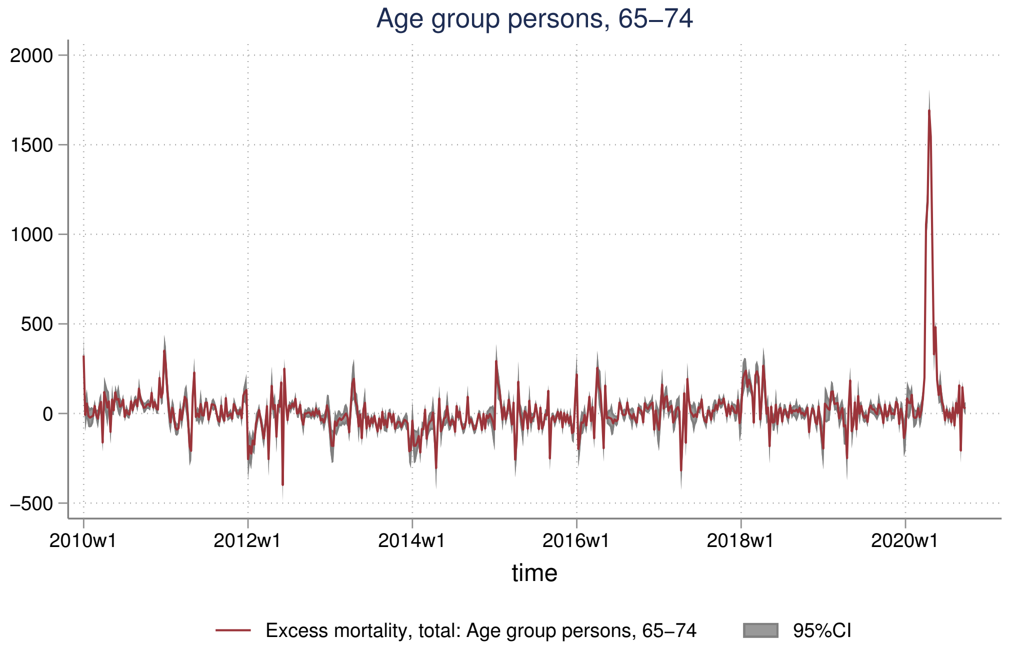

Figure 152: All 65-74 all-cause excess deaths, from 2019 week 1

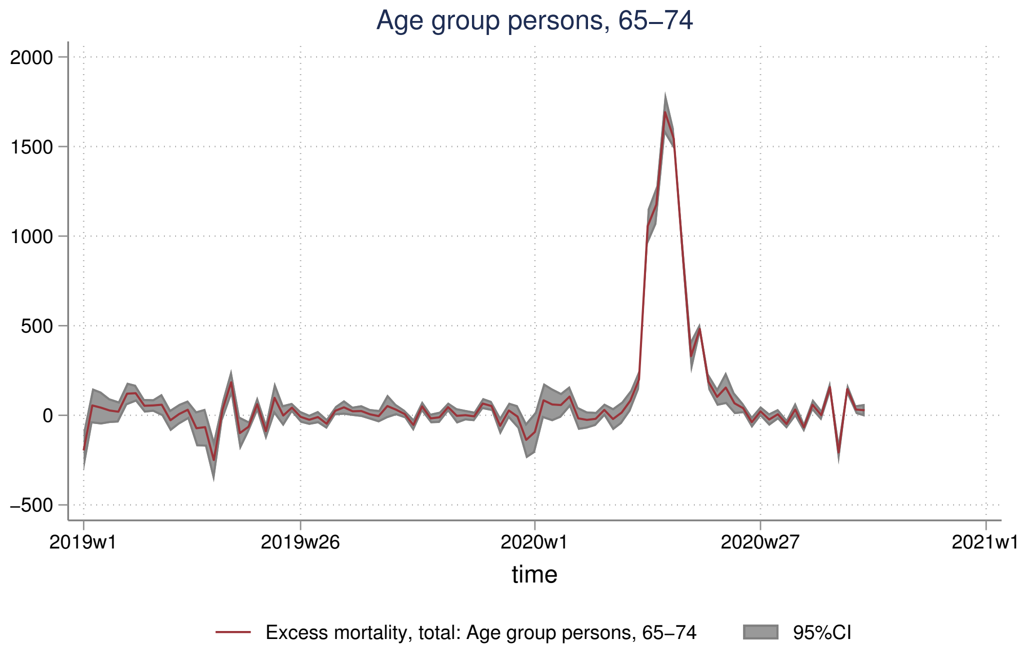

Figure 153: All 65-74 all-cause excess deaths, from 2020 week 1

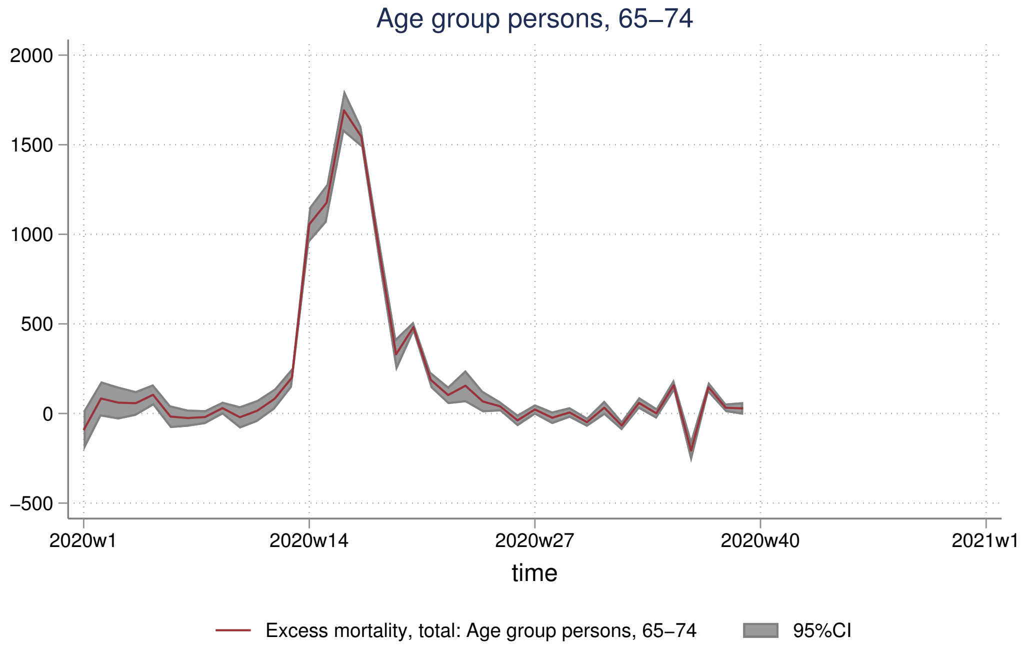

Figure 154: All 65-74 all-cause excess deaths (−COVID19), from 2010 week 1

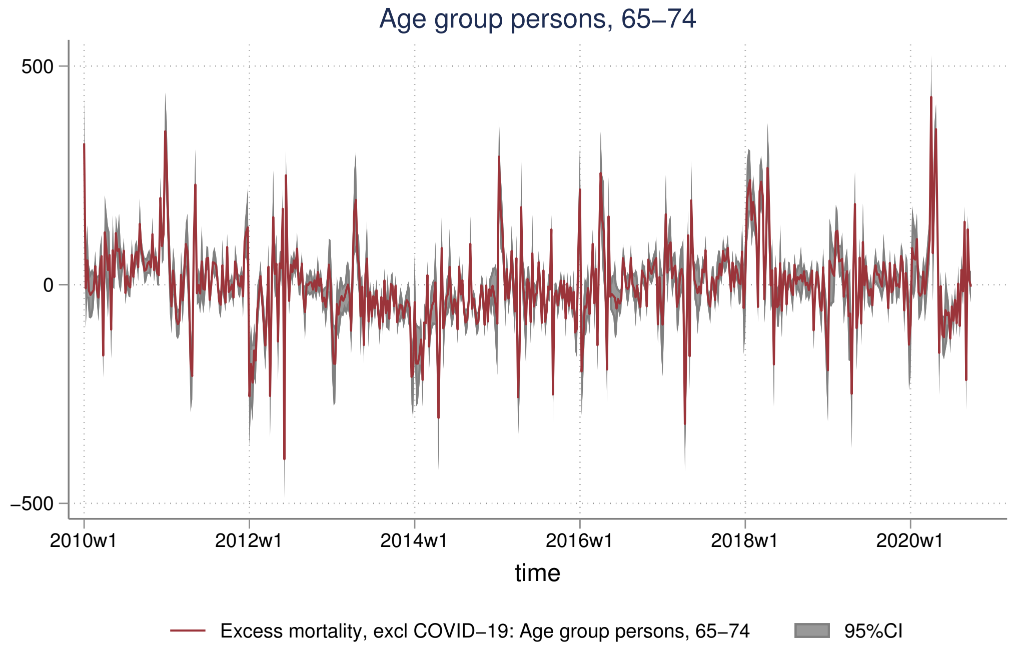

Figure 155: All 65-74 all-cause excess deaths (−COVID19), from 2019 week 1

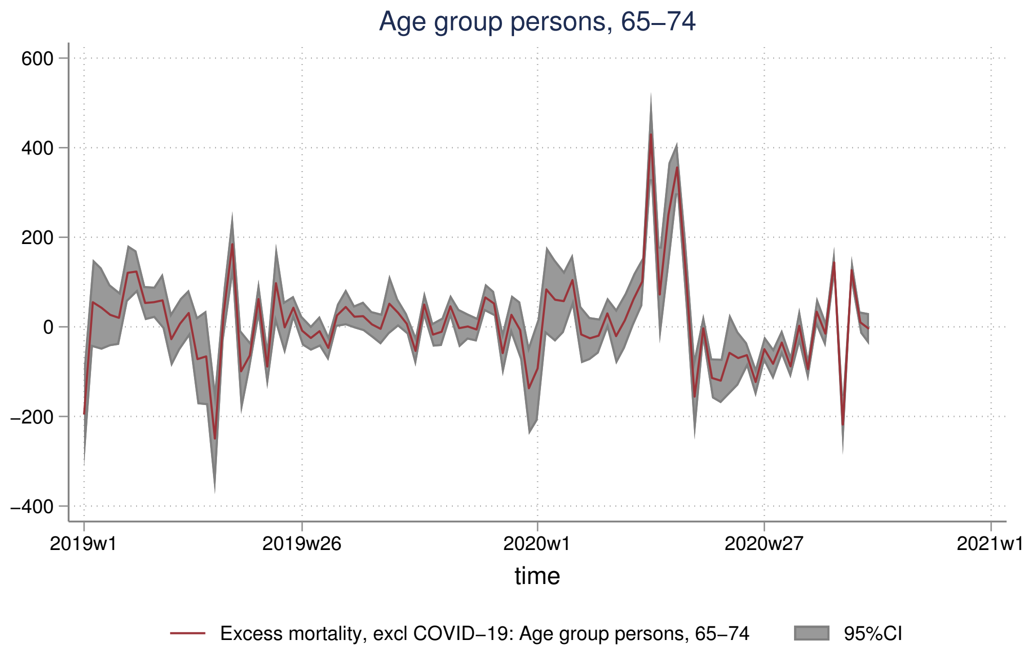

Figure 156: All 65-74 all-cause excess deaths (−COVID19), from 2020 week 1

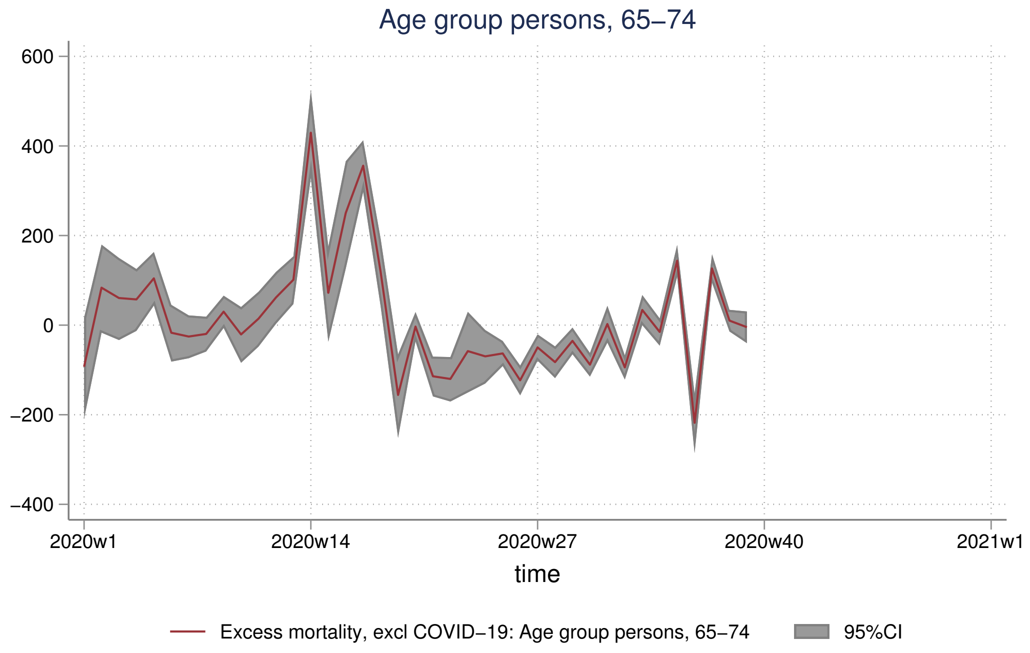

3.7 All aged 75-84

Figure 157: All 75-84 mortality time trend and model, from 2010 week 1

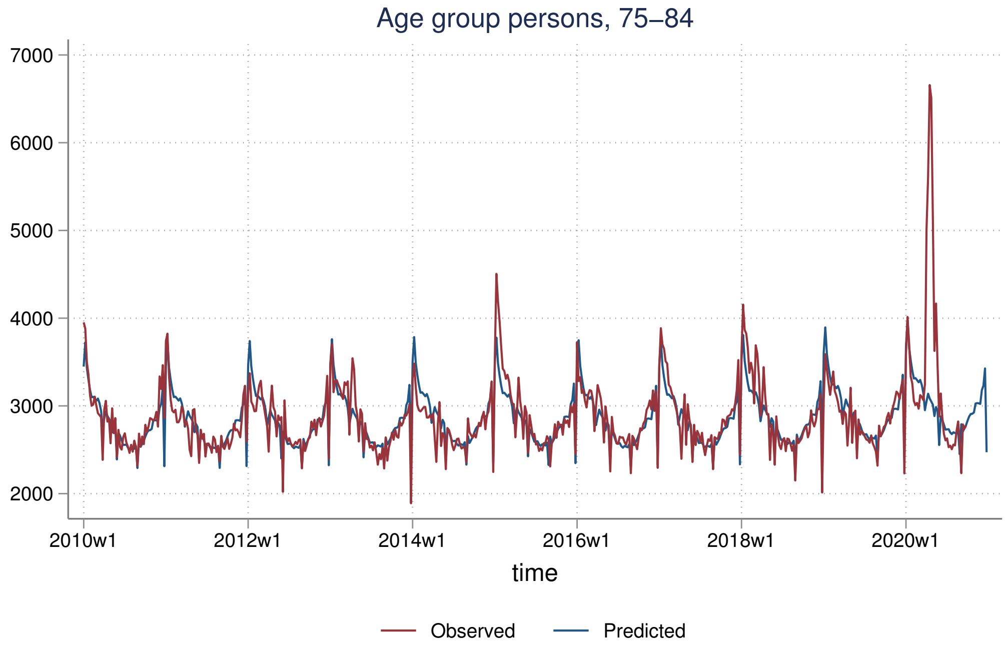

Figure 158: All 75-84 mortality time trend and model, from 2019 week 1

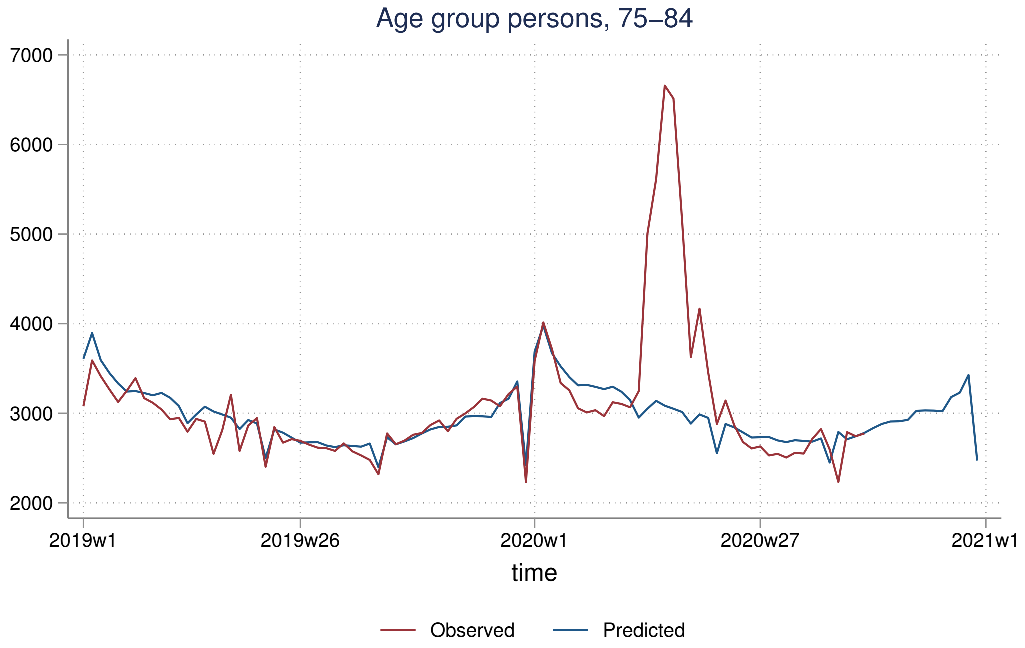

Figure 159: All 75-84 mortality time trend and model, from 2020 week 1

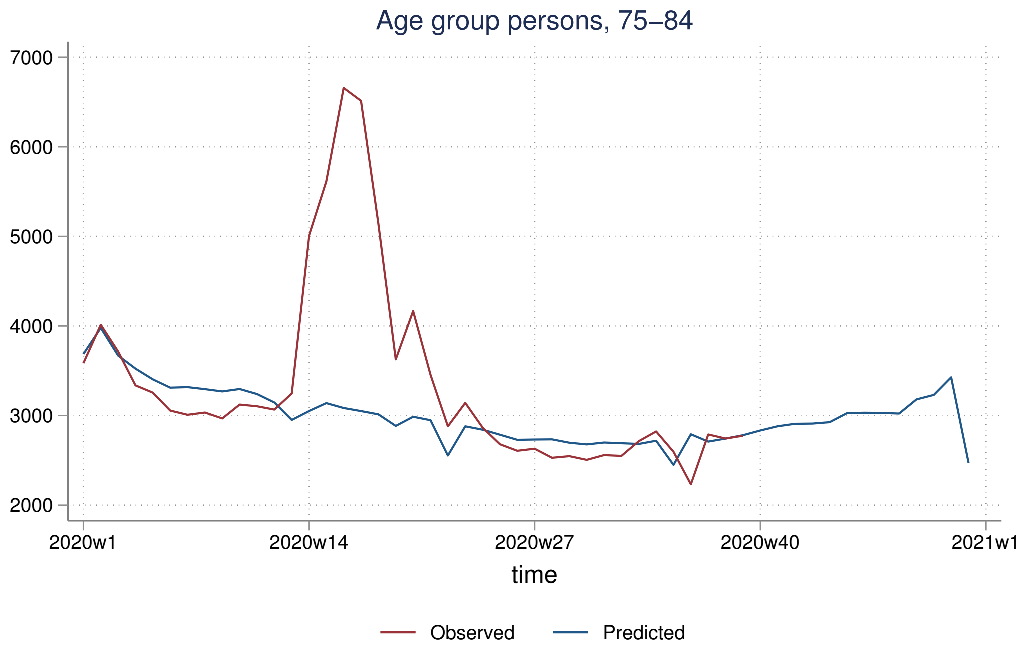

Figure 160: All 75-84 all-cause excess deaths, from 2010 week 1

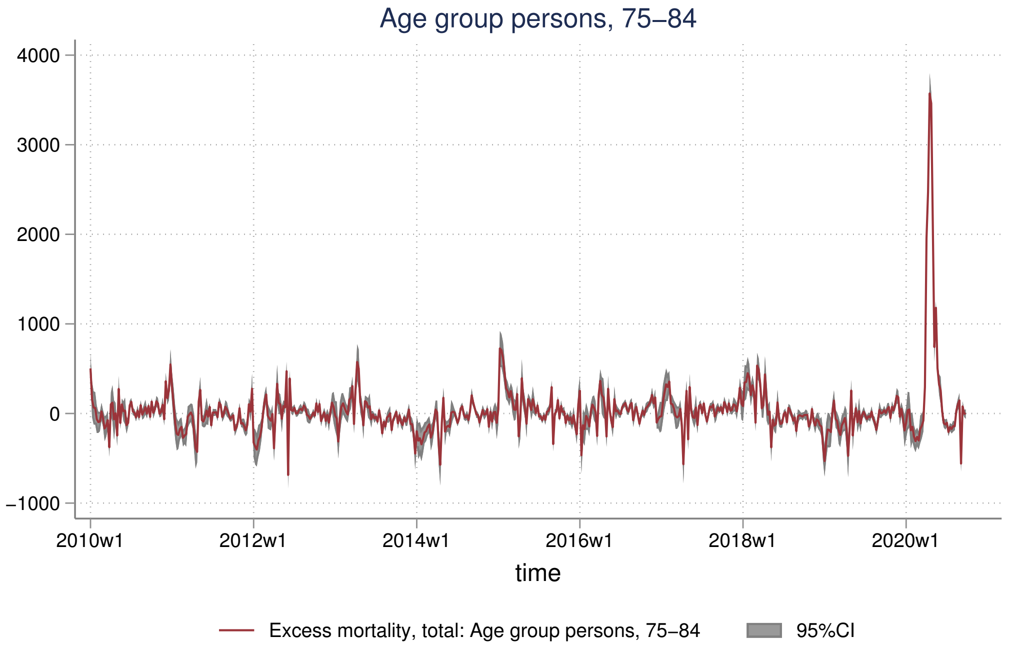

Figure 161: All 75-84 all-cause excess deaths, from 2019 week 1

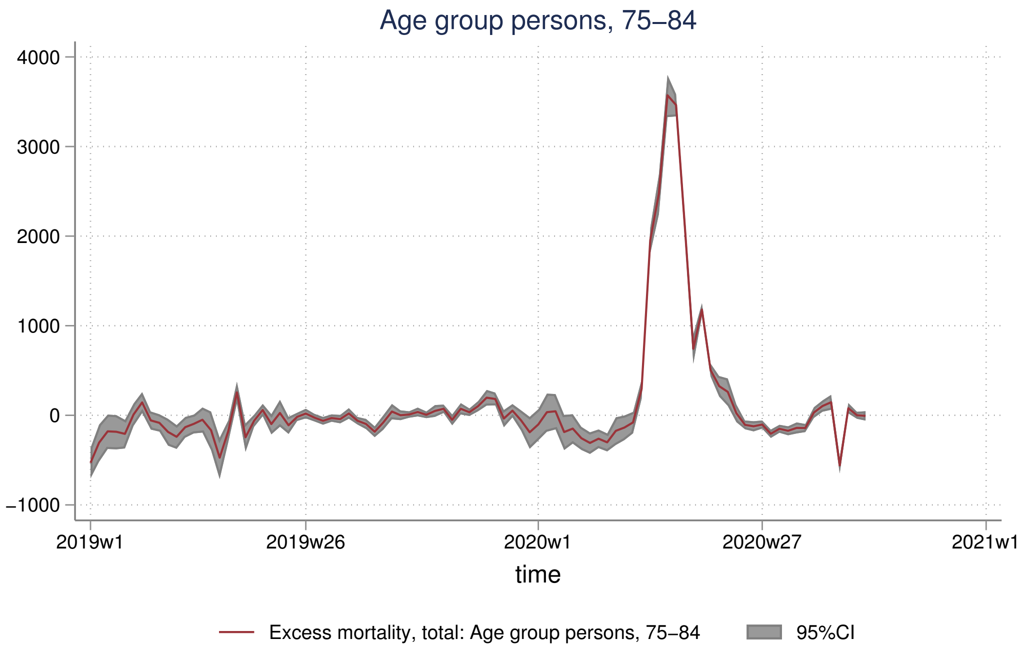

Figure 162: All 75-84 all-cause excess deaths, from 2020 week 1

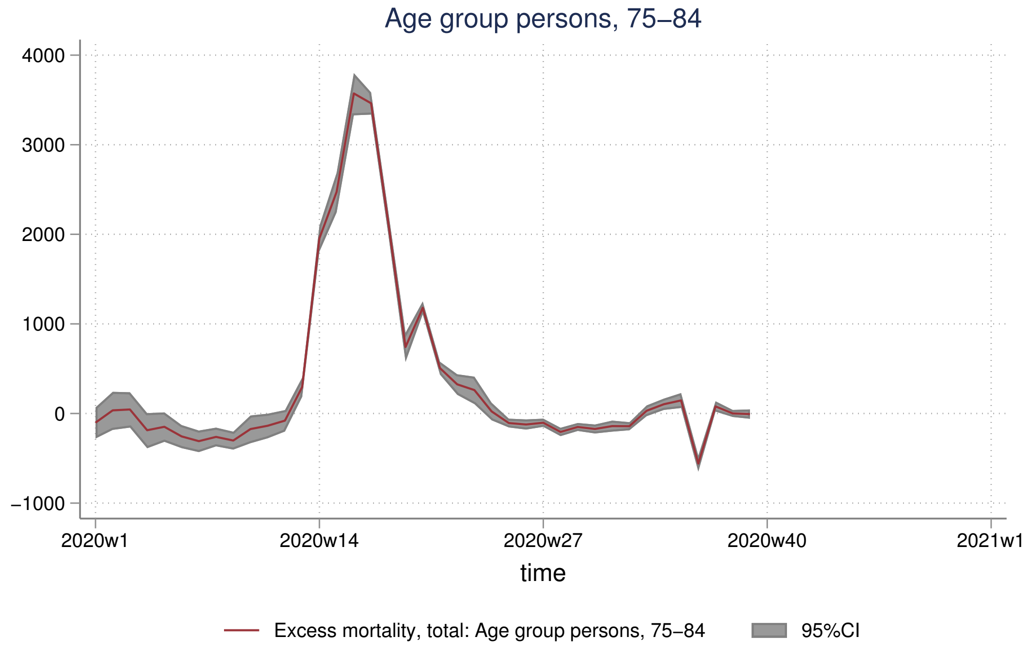

Figure 163: All 75-84 all-cause excess deaths (−COVID19), from 2010 week 1

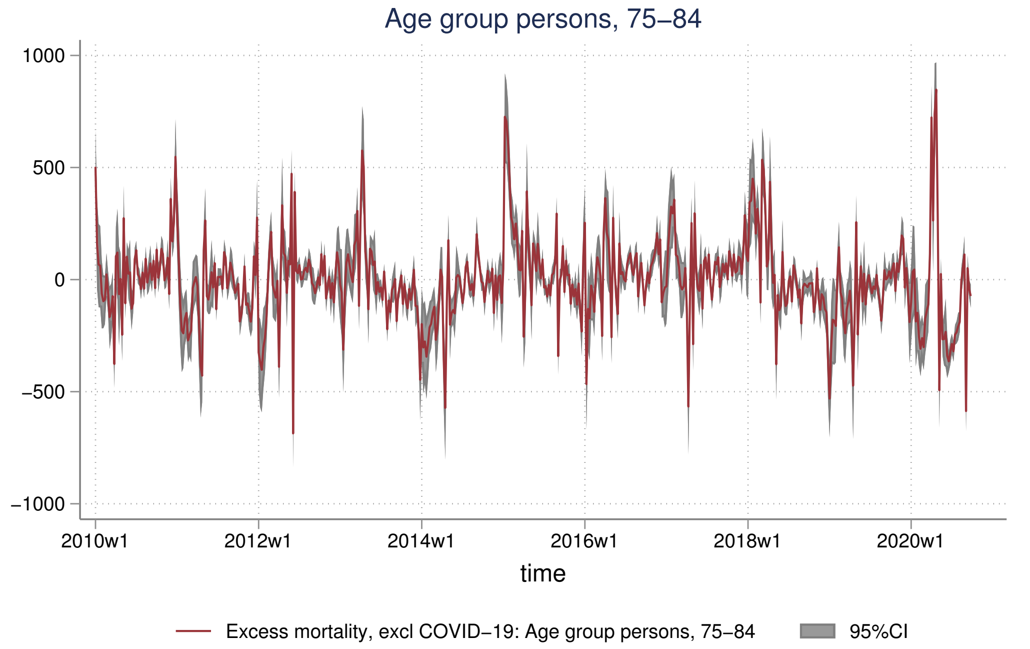

Figure 164: All 75-84 all-cause excess deaths (−COVID19), from 2019 week 1

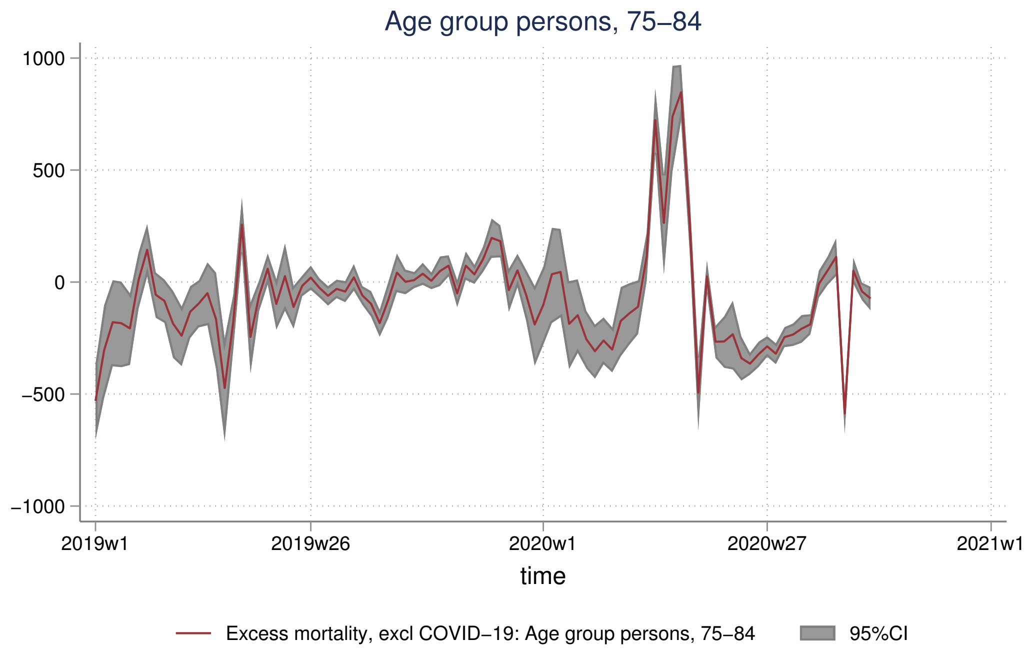

Figure 165: All 75-84 all-cause excess deaths (−COVID19), from 2020 week 1

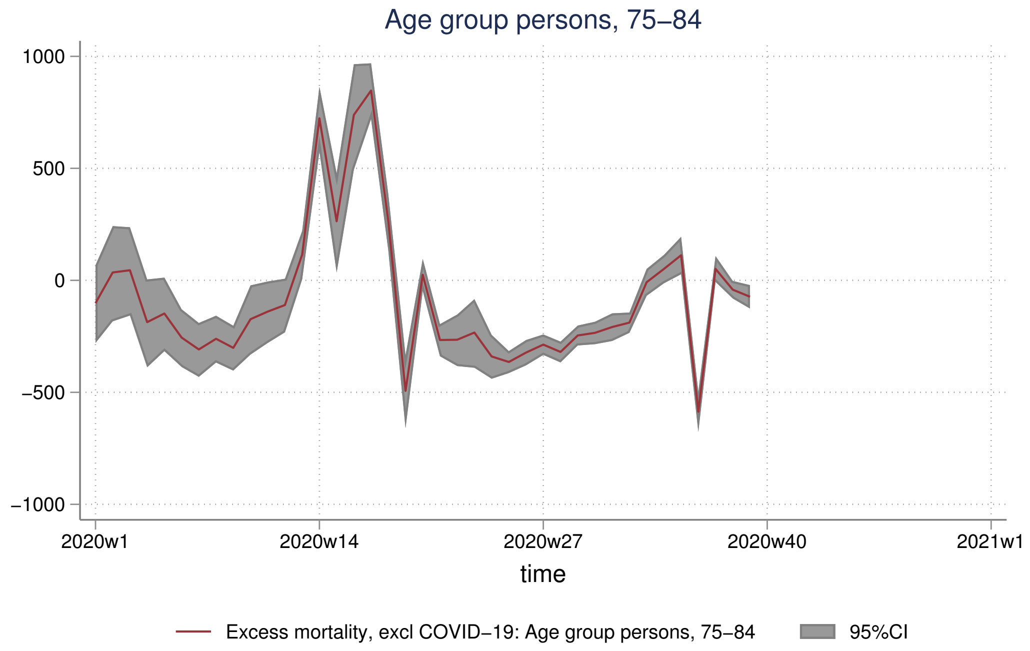

3.8 All aged 85+

Figure 166: All 85+ mortality time trend and model, from 2010 week 1

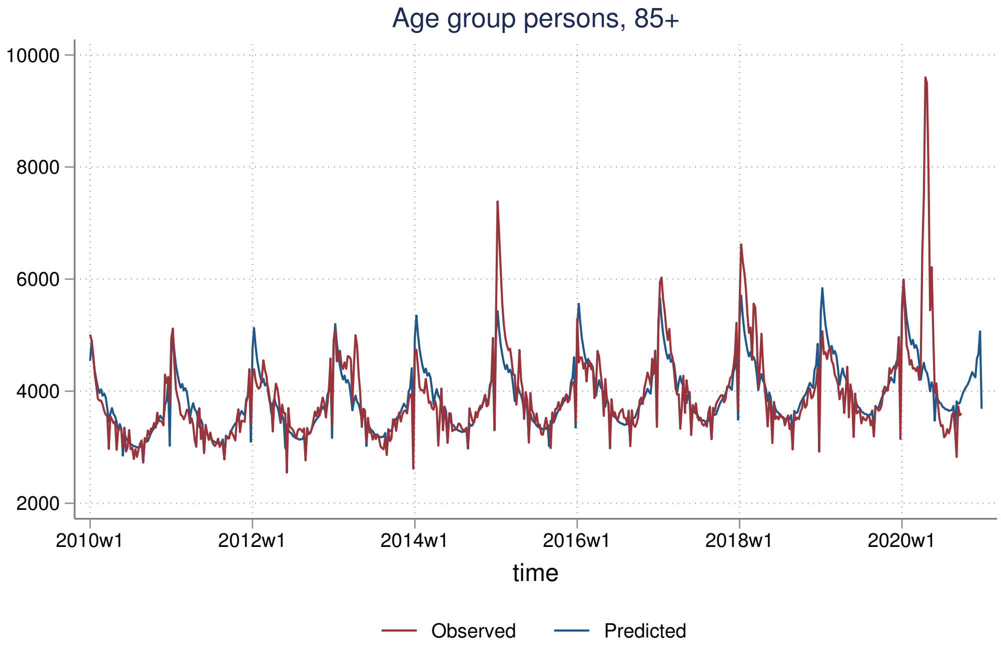

Figure 167: All 85+ mortality time trend and model, from 2019 week 1

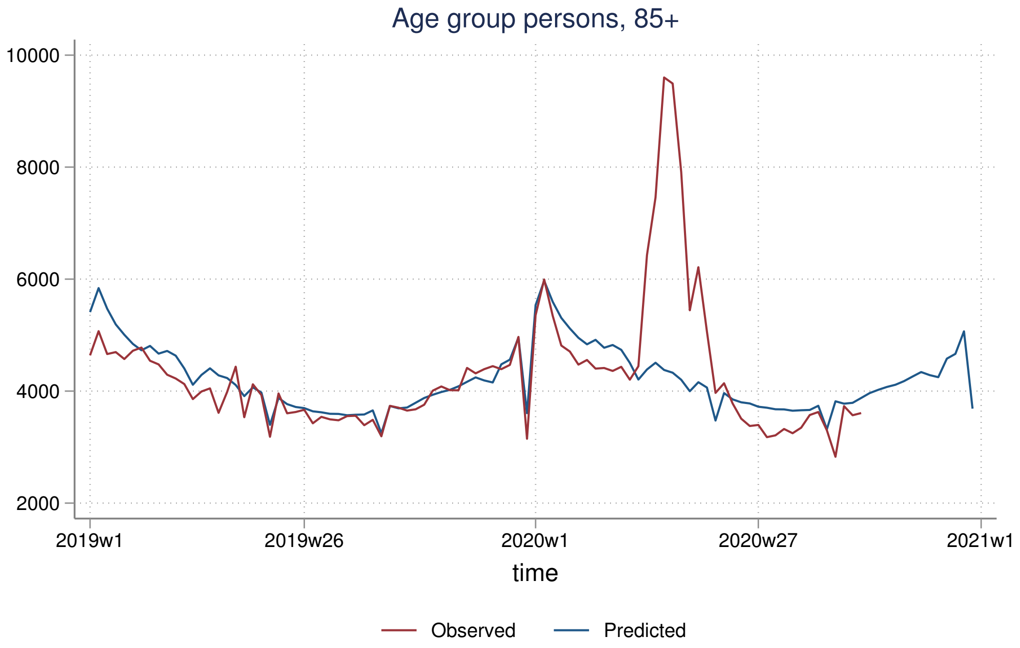

Figure 168: All 85+ mortality time trend and model, from 2020 week 1

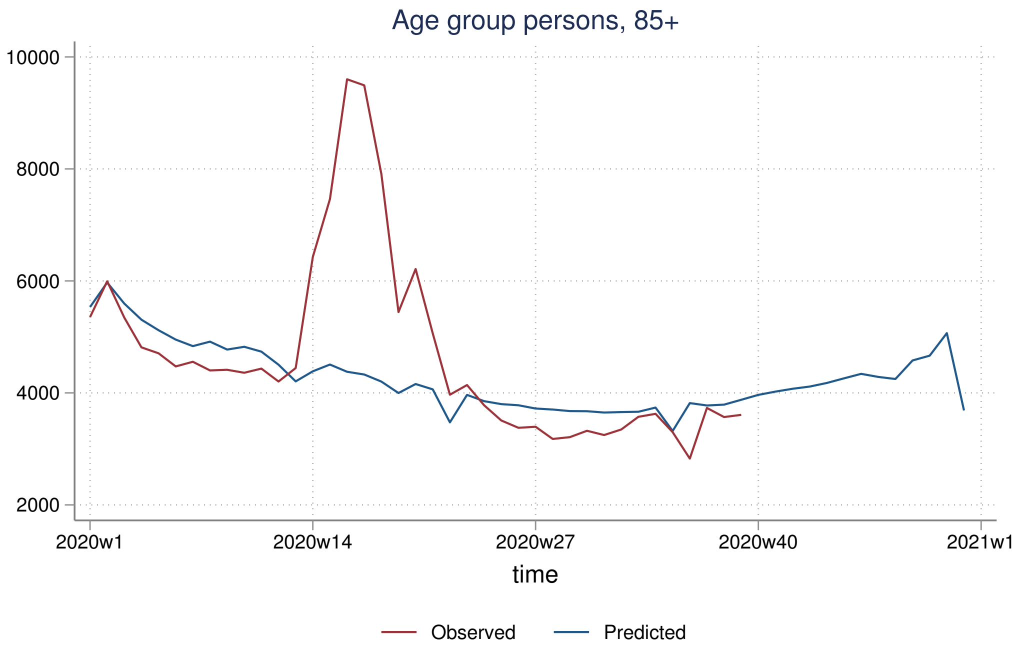

Figure 169: All 85+ all-cause excess deaths, from 2010 week 1

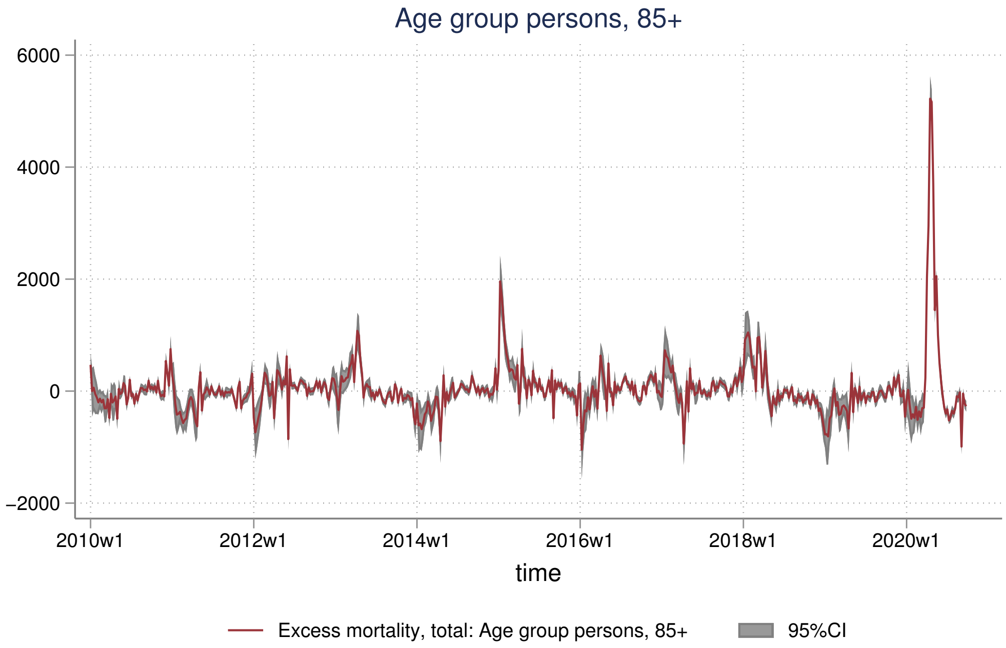

Figure 170: All 85+ all-cause excess deaths, from 2019 week 1

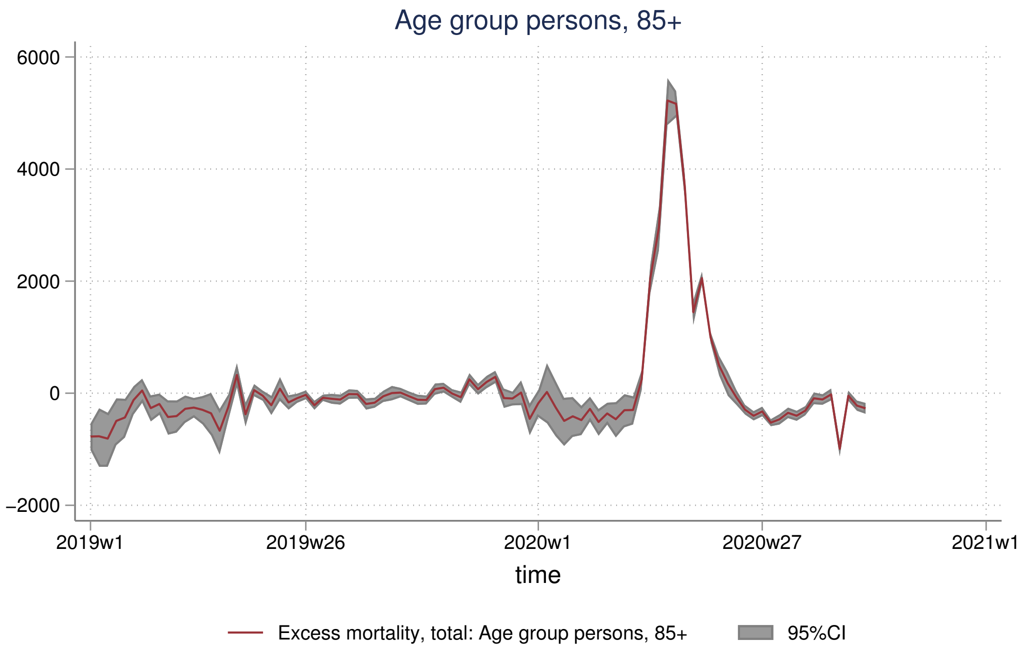

Figure 171: All 85+ all-cause excess deaths, from 2020 week 1

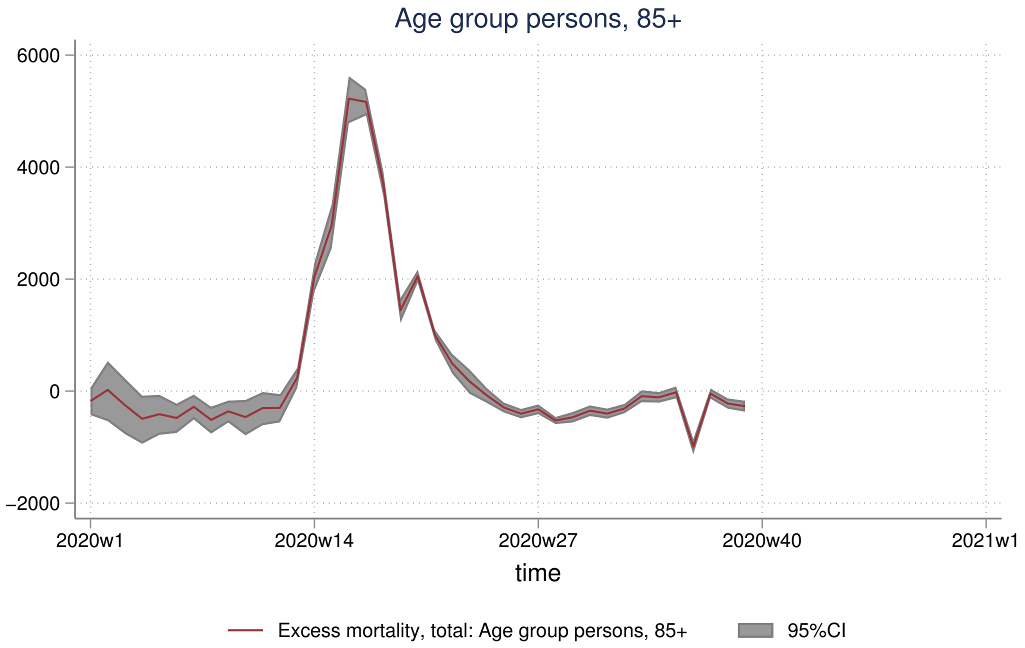

Figure 172: All 85+ all-cause excess deaths (−COVID19), from 2010 week 1

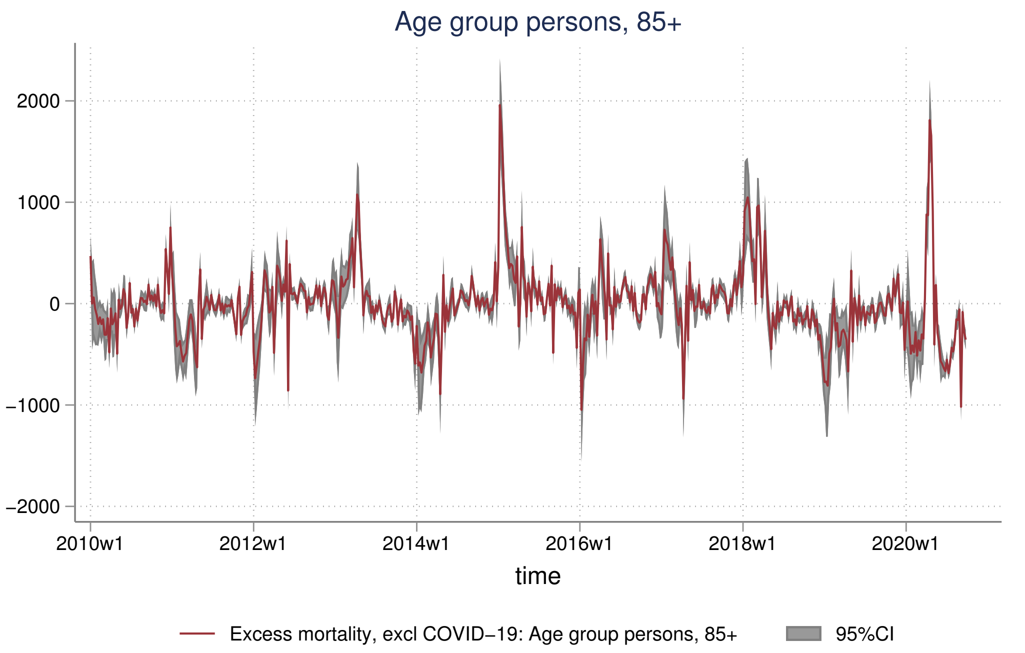

Figure 173: All 85+ all-cause excess deaths (−COVID19), from 2019 week 1

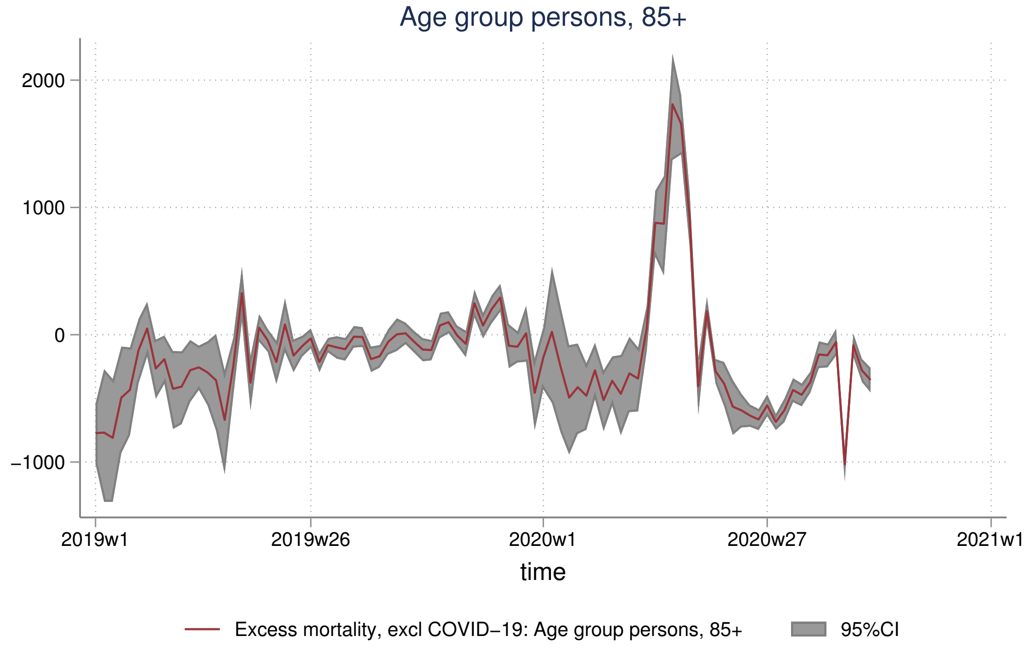

Figure 174: All 85+ all-cause excess deaths (−COVID19), from 2020 week 1

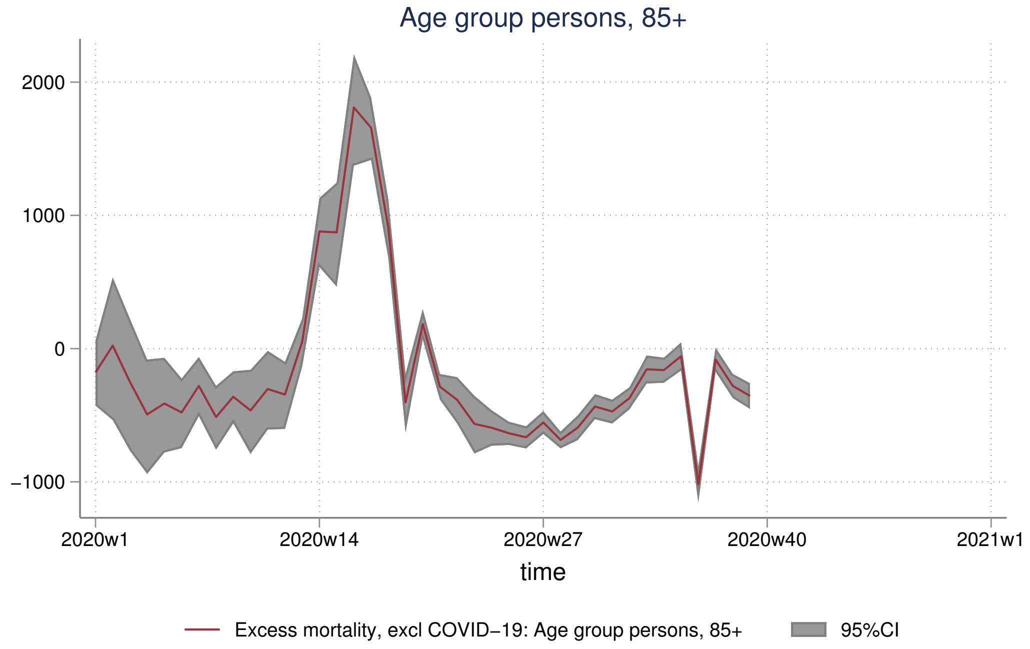

4 Age groups, males

4.1 Time trends

Figure 175: Male age group mortality time trends, from 2010 wk1

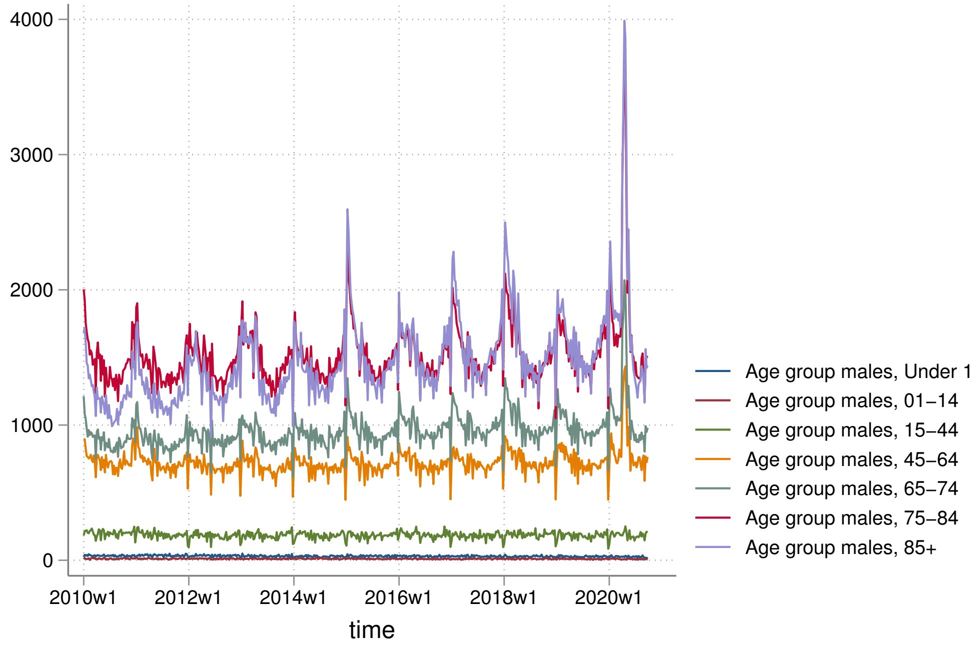

Figure 176: Male age group mortality time trends, from 2019 wk1

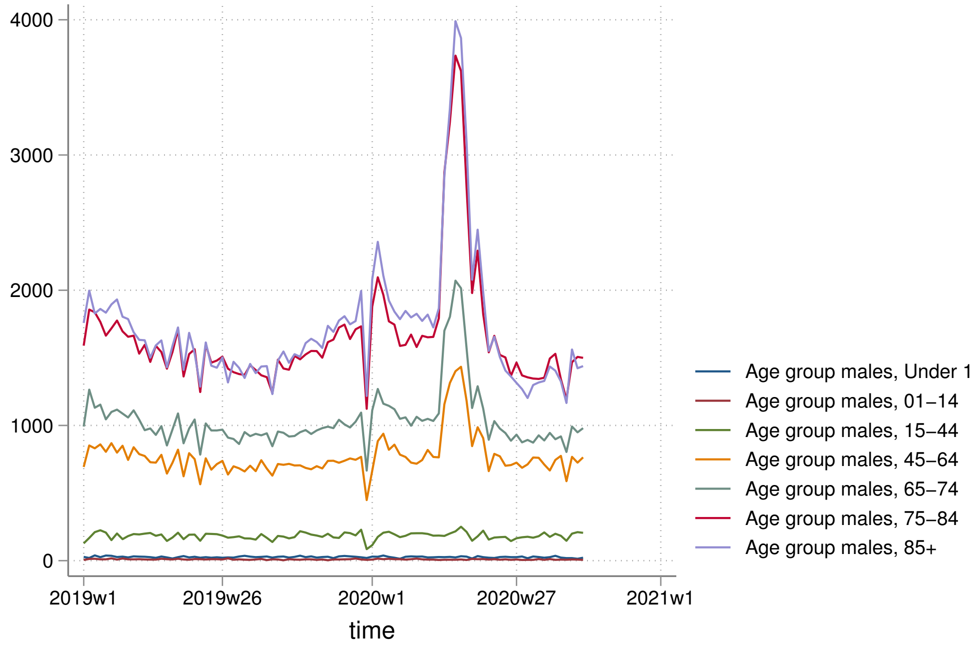

Figure 177: Male age group mortality time trends, from 2020 wk1

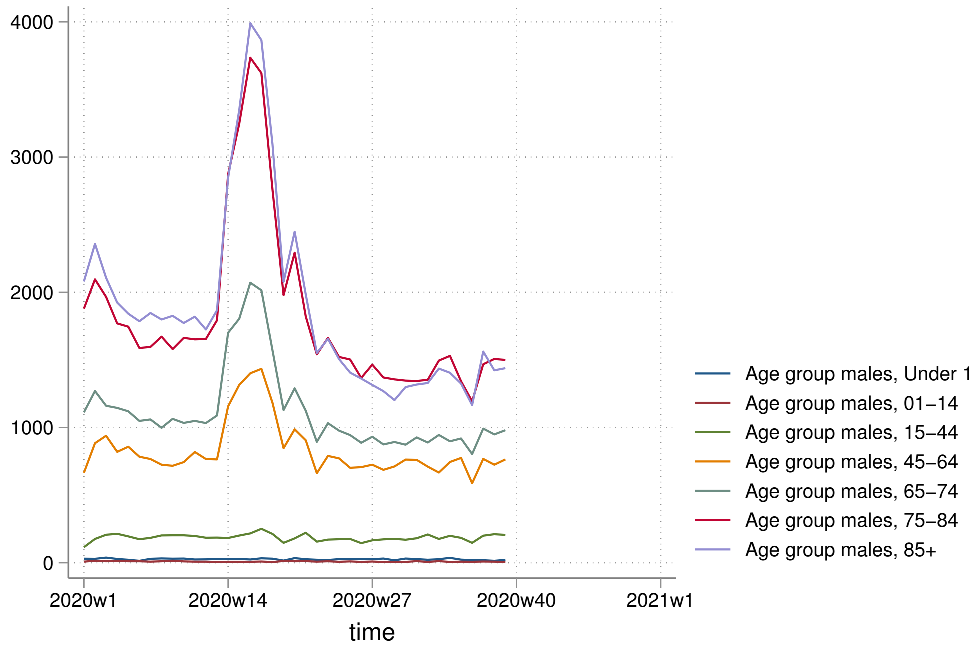

4.2 Males aged under 1

Figure 178: Males < 1 mortality time trend and model, from 2010 wk1

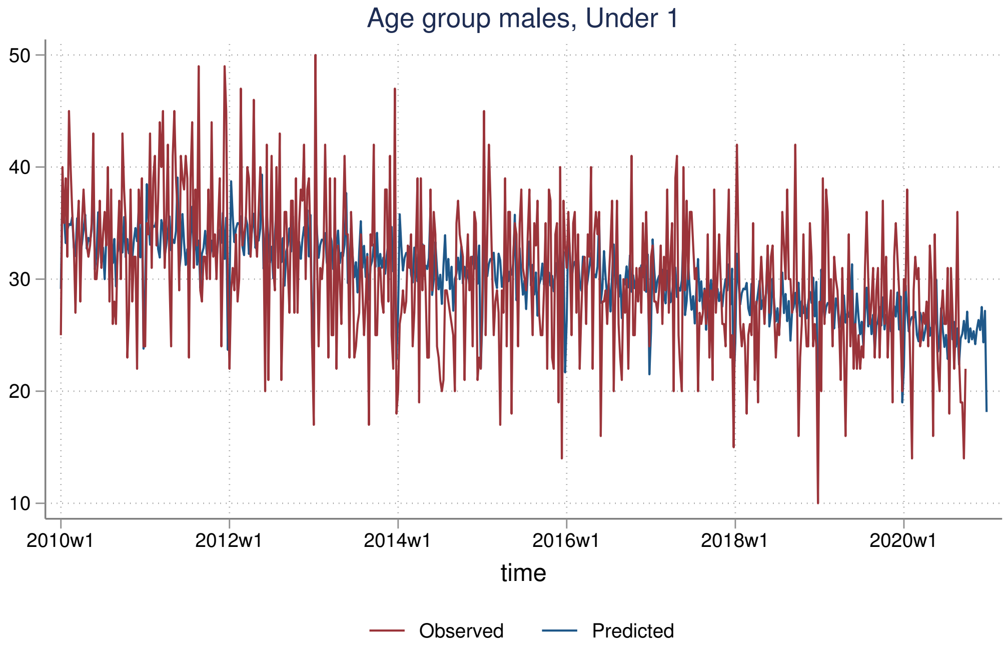

Figure 179: Males < 1 mortality time trend and model, from 2019 wk1

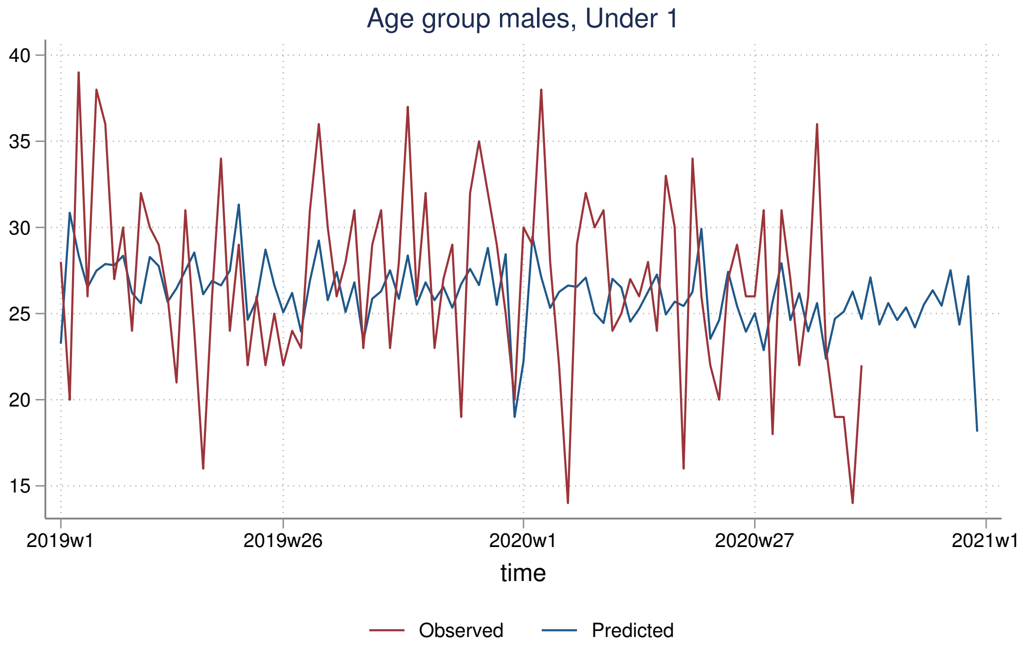

Figure 180: Males < 1 mortality time trend and model, from 2020 wk1

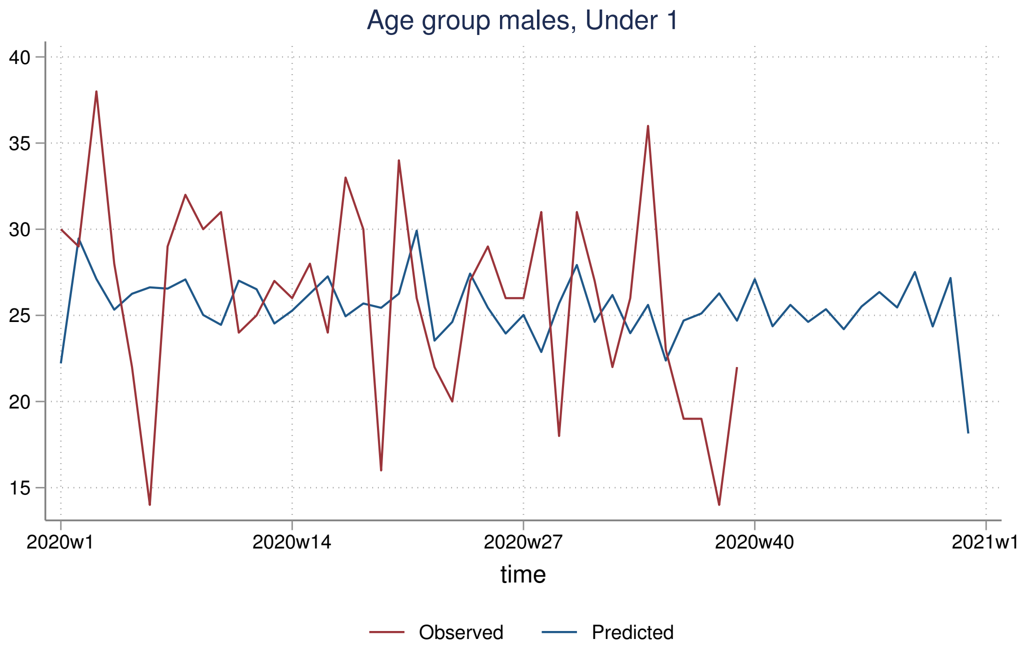

Figure 181: Males < 1 all-cause excess deaths, from 2010 wk1

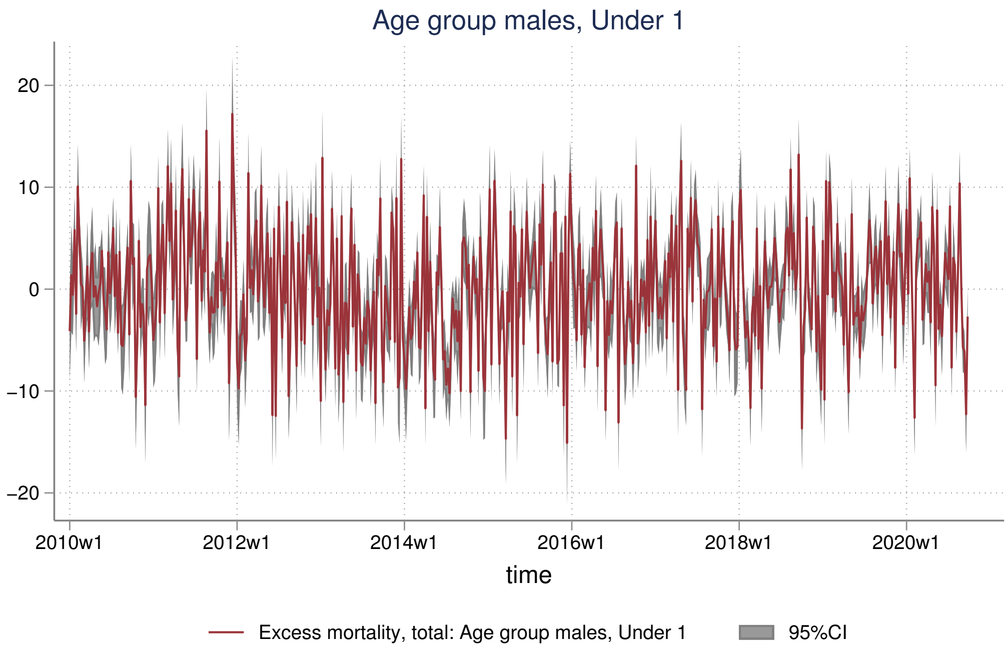

Figure 182: Males < 1 all-cause excess deaths, from 2019 wk1

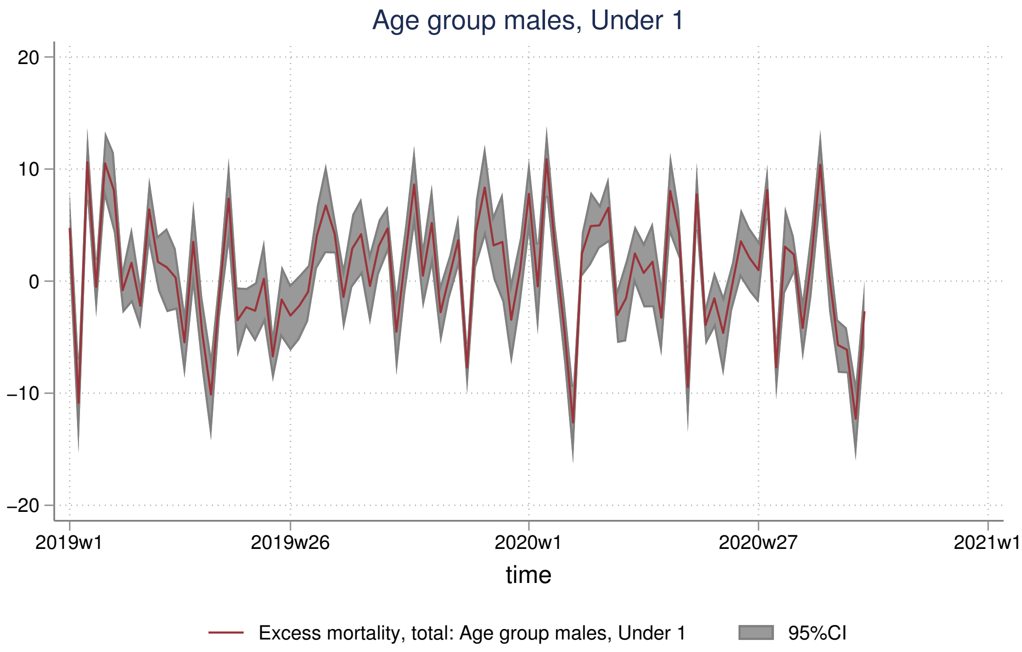

Figure 183: Males < 1 all-cause excess deaths, from 2020 wk1

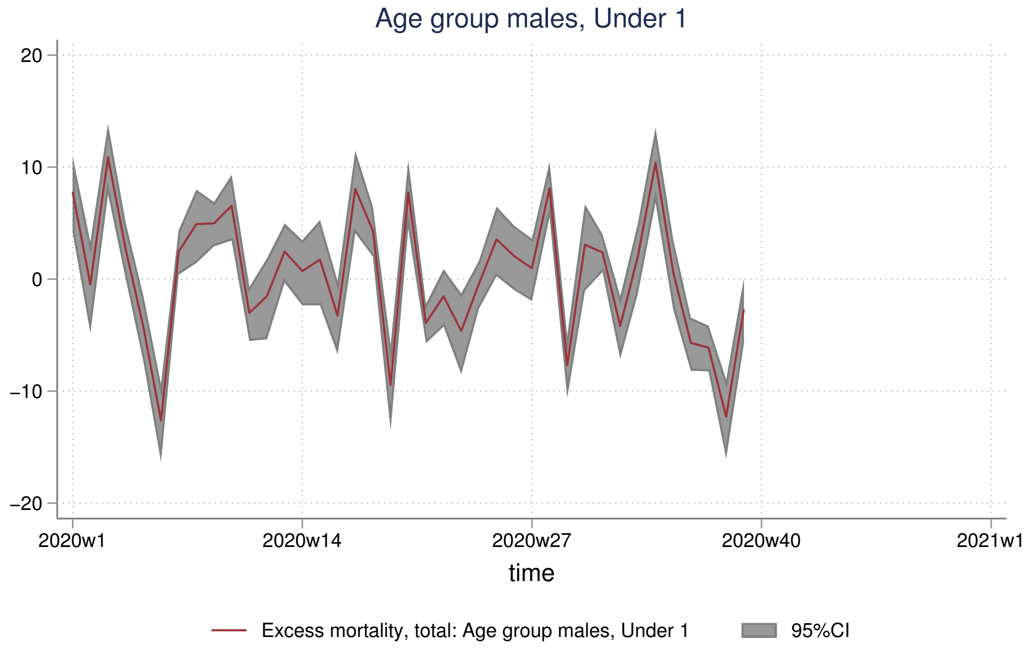

Figure 184: Males < 1 all-cause excess deaths minus COVID19, from 2010 wk1

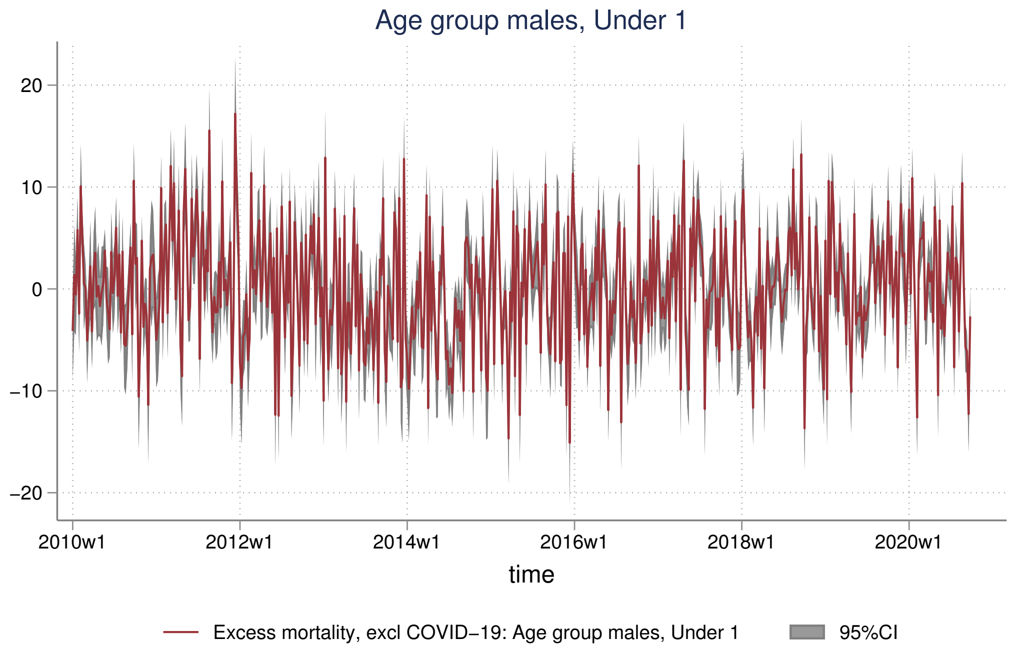

Figure 185: Males < 1 all-cause excess deaths minus COVID19, from 2019 wk1

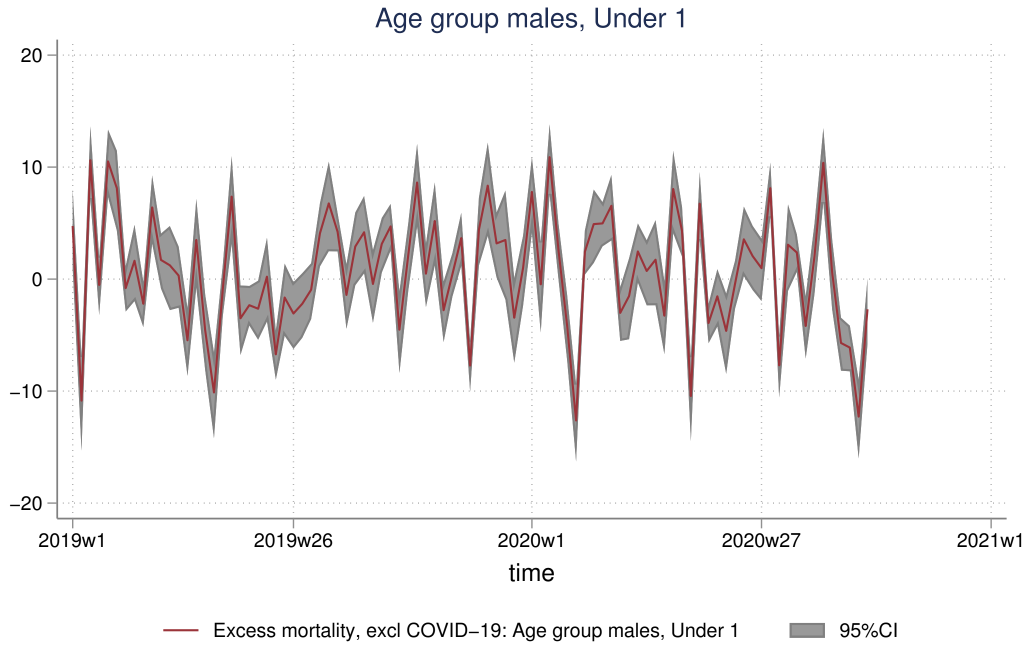

Figure 186: Males < 1 all-cause excess deaths minus COVID19, from 2020 wk1

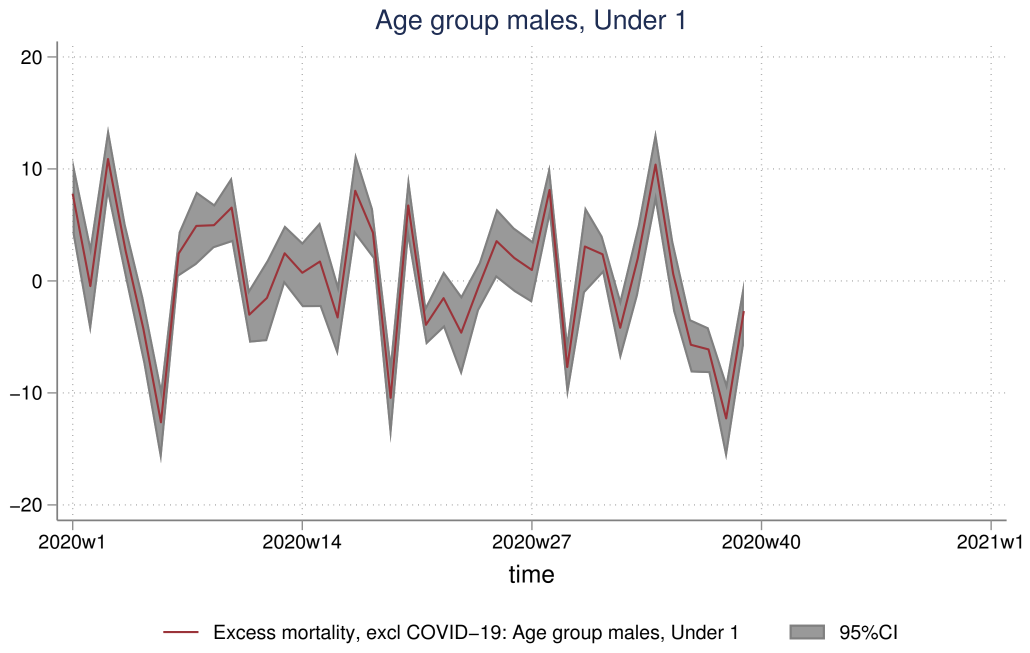

4.3 Males aged 01-14

Figure 187: Males 01-14 mortality time trend and model, from 2010 wk1

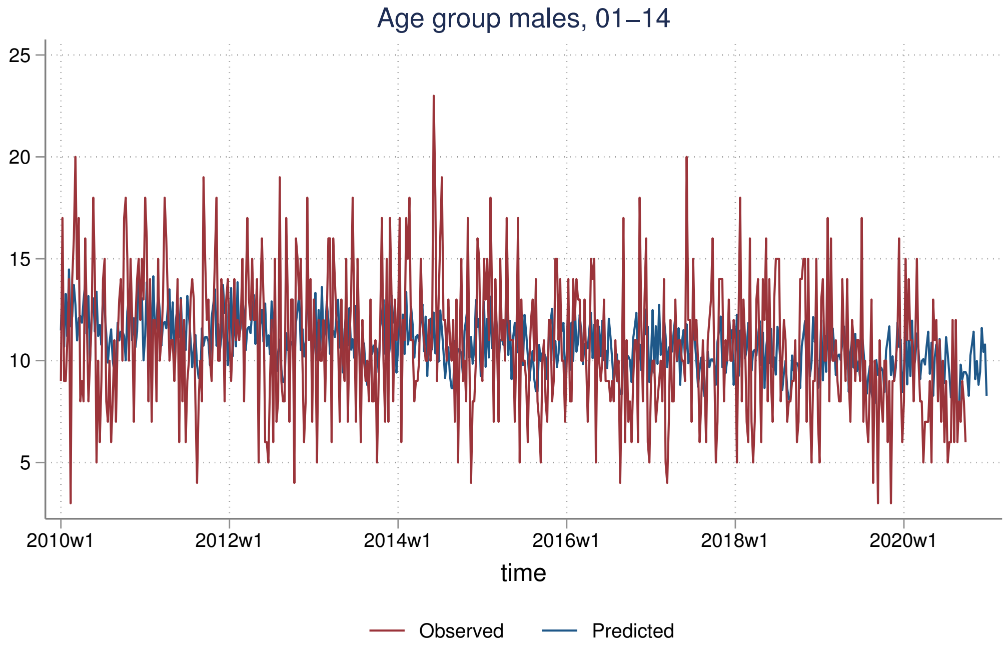

Figure 188: Males 01-14 mortality time trend and model, from 2019 wk1

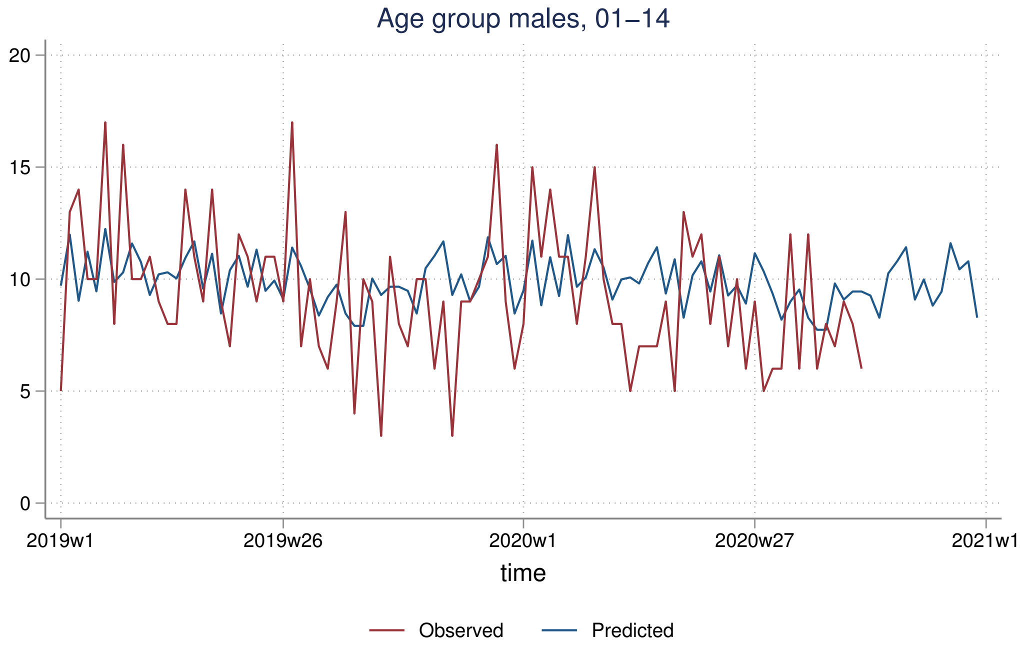

Figure 189: Males 01-14 mortality time trend and model, from 2020 wk1

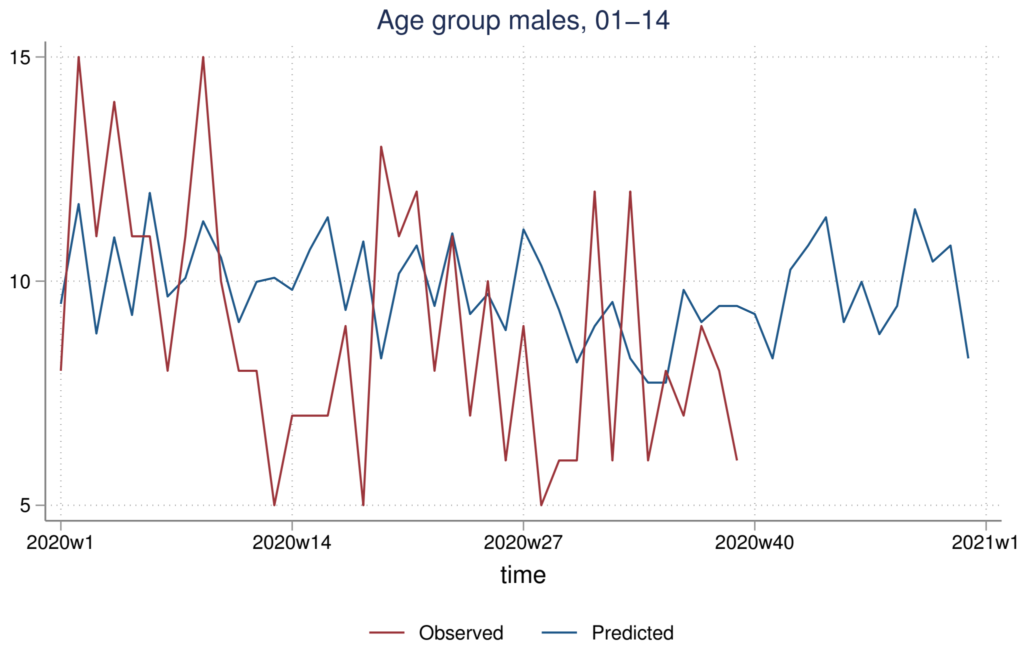

Figure 190: Males 01-14 all-cause excess deaths, from 2010 wk1

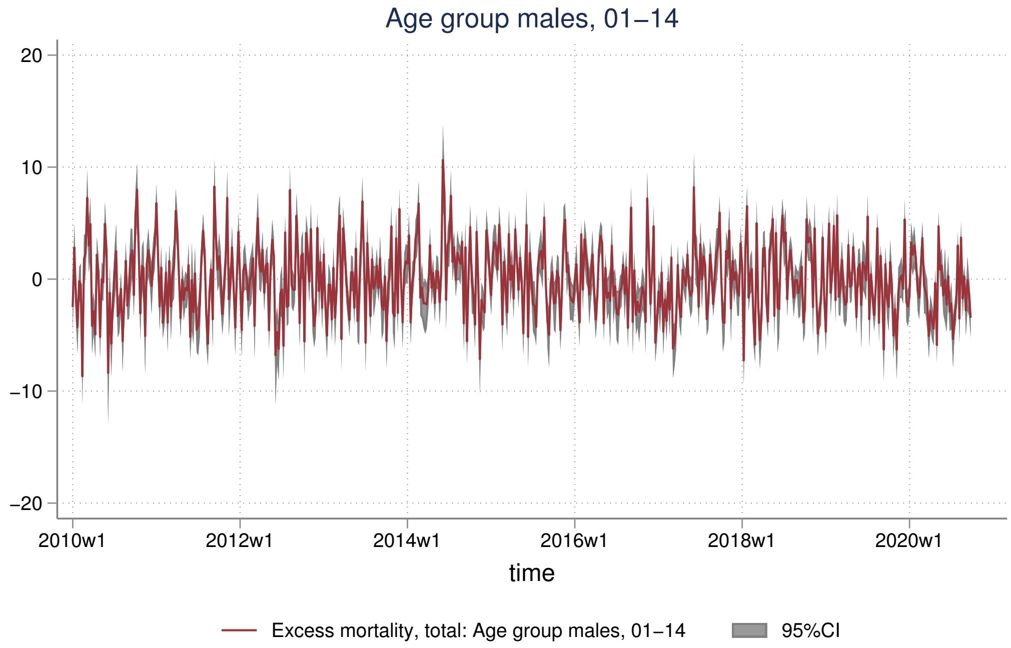

Figure 191: Males 01-14 all-cause excess deaths, from 2019 wk1

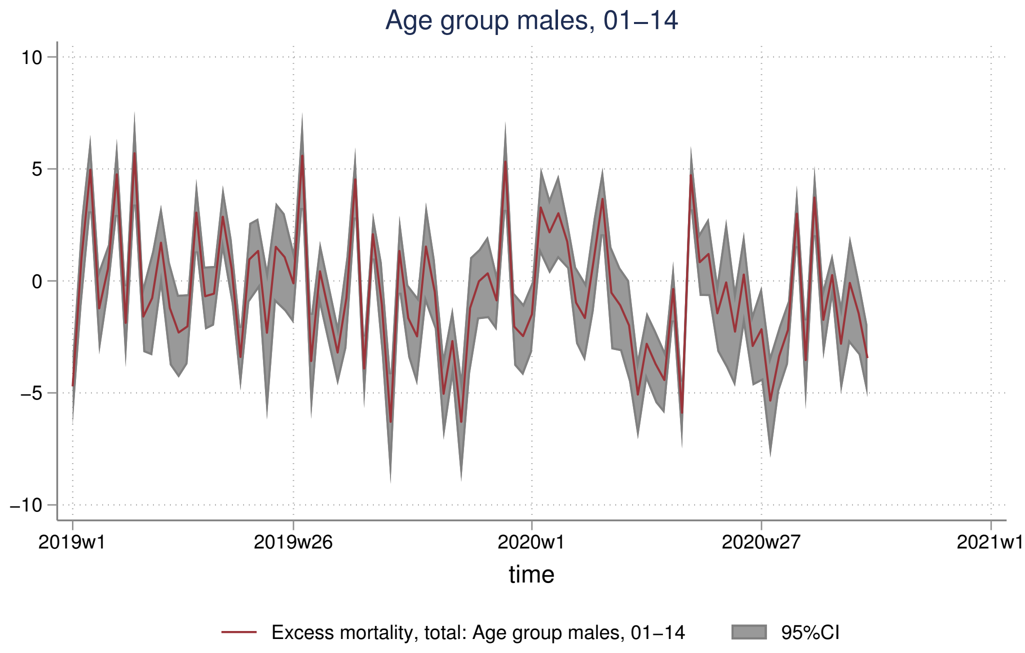

Figure 192: Males 01-14 all-cause excess deaths, from 2020 wk1

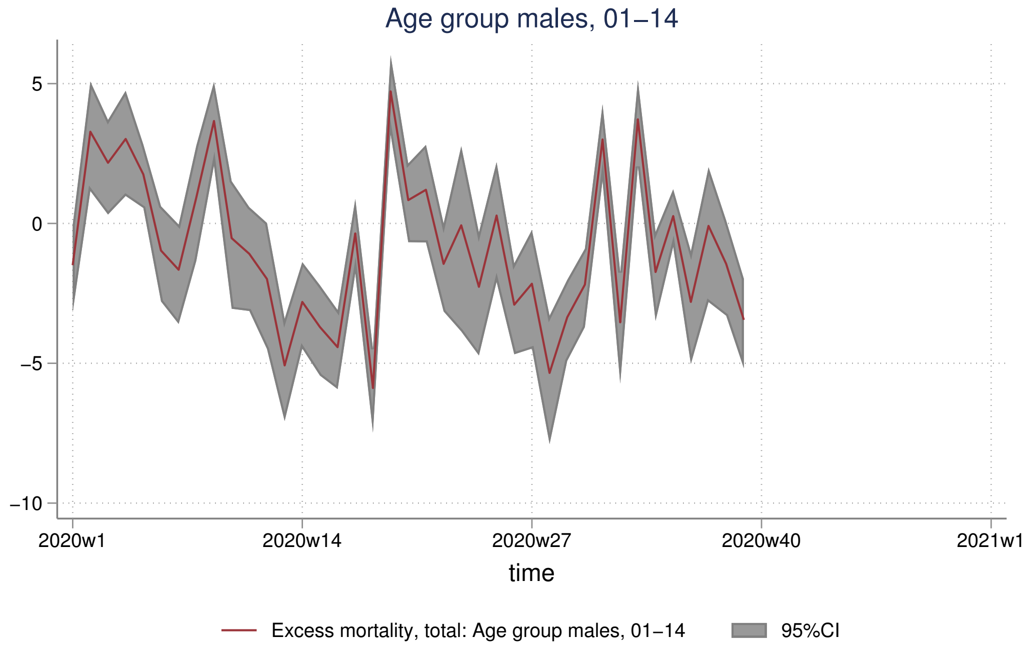

Figure 193: Males 01-14 all-cause excess deaths (−COVID19), from 2010 wk1

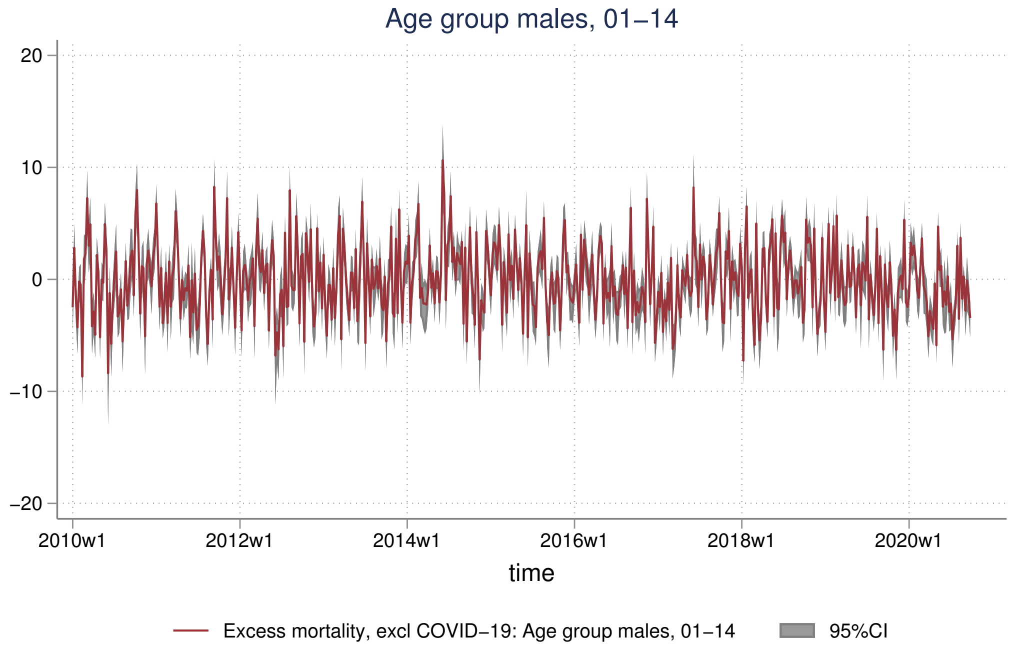

Figure 194: Males 01-14 all-cause excess deaths (−COVID19), from 2019 wk1

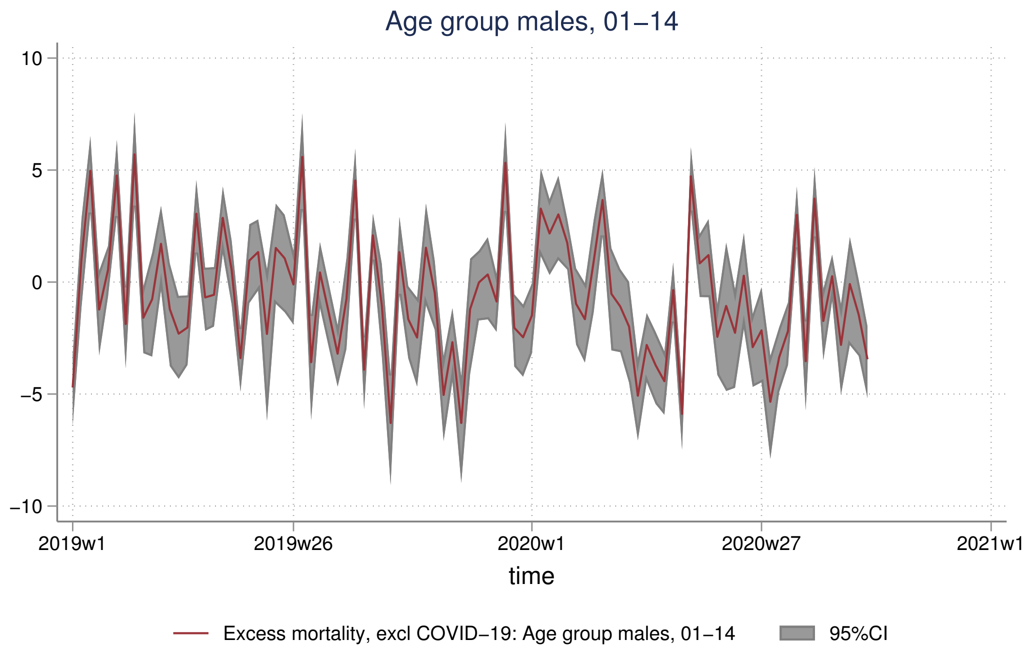

Figure 195: Males 01-14 all-cause excess deaths (−COVID19), from 2020 wk1

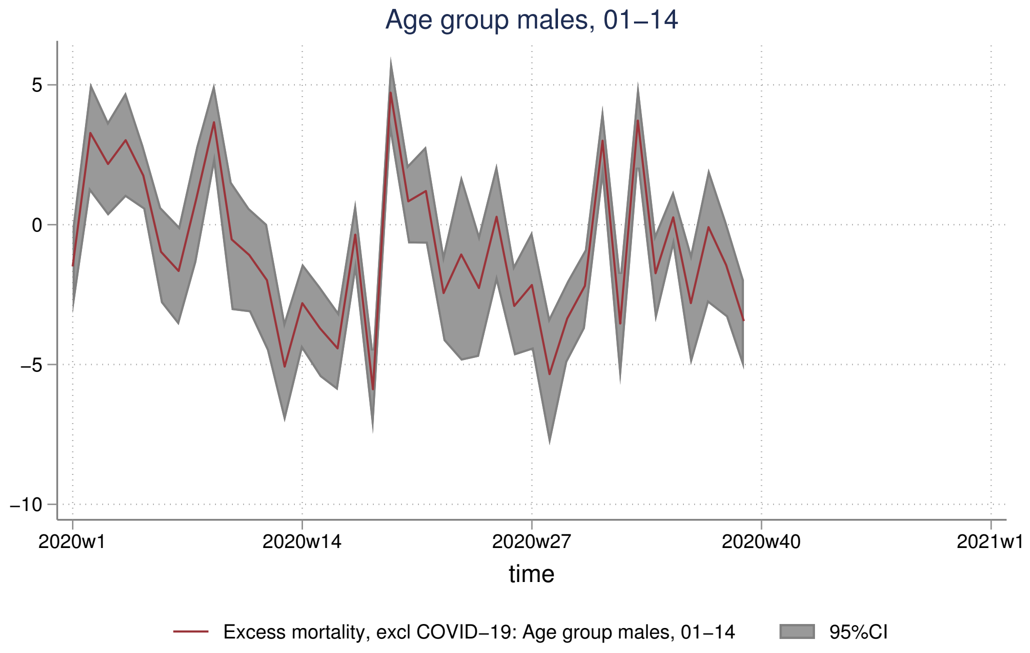

4.4 Males aged 15-44

Figure 196: Males 15-44 mortality time trend and model, from 2010 wk1

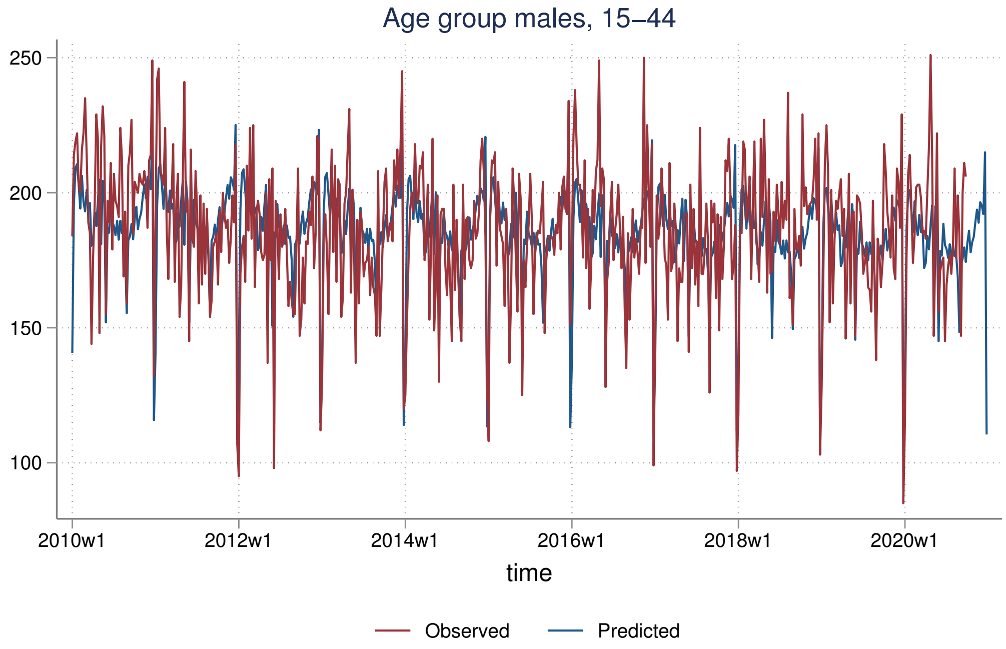

Figure 197: Males 15-44 mortality time trend and model, from 2019 wk1

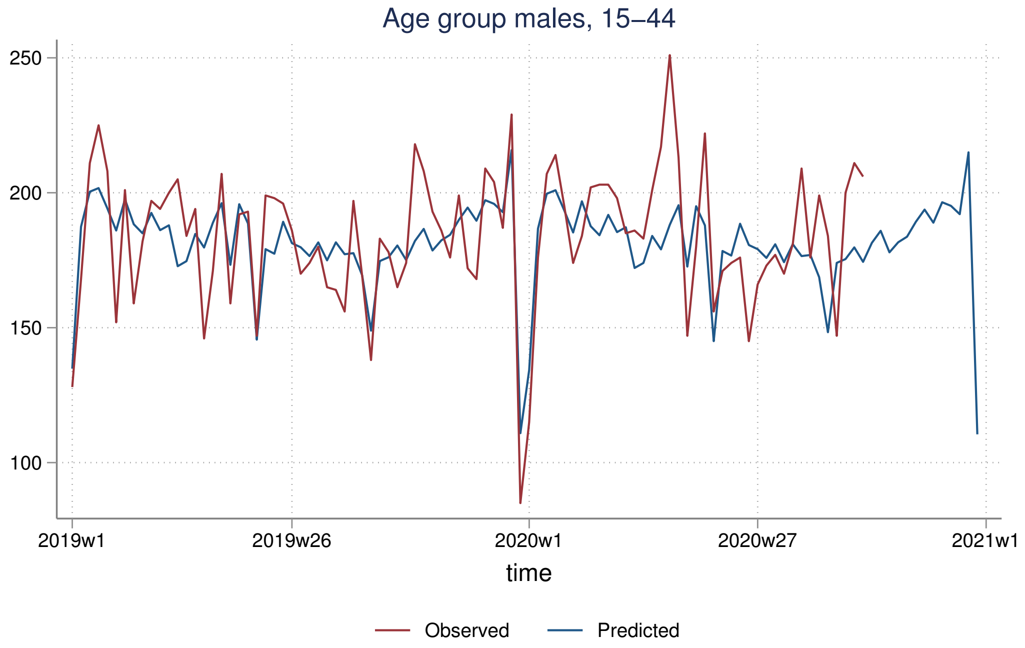

Figure 198: Males 15-44 mortality time trend and model, from 2020 wk1

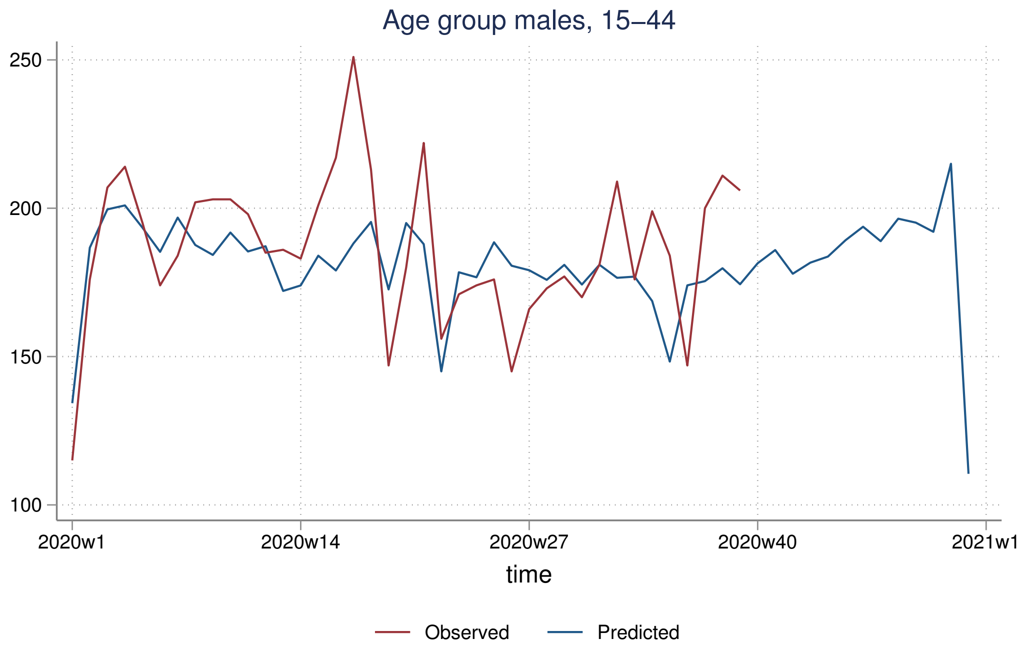

Figure 199: Males 15-44 all-cause excess deaths, from 2010 wk1

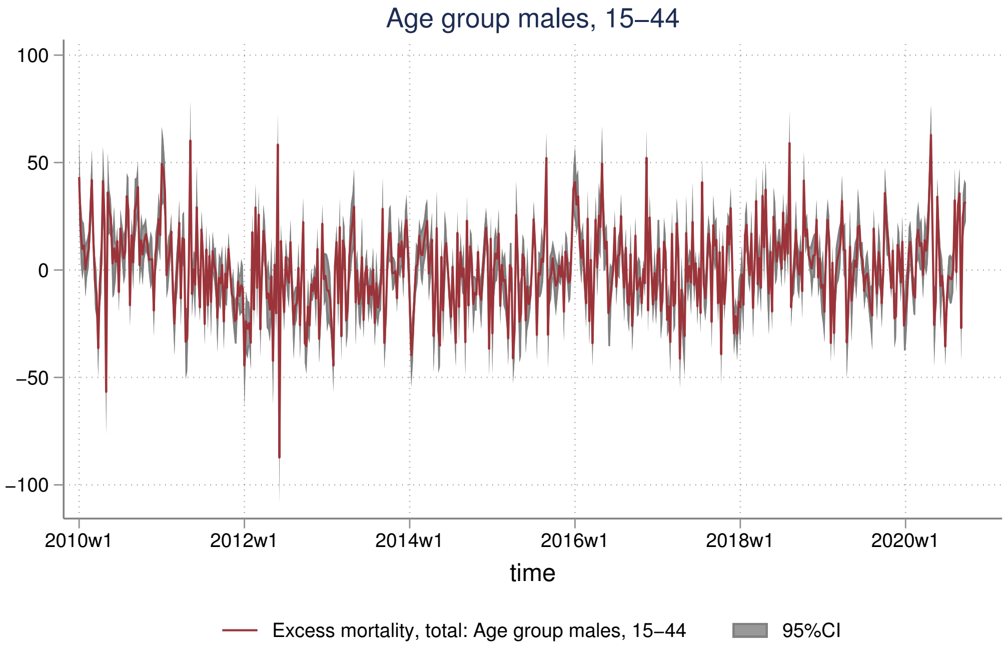

Figure 200: Males 15-44 all-cause excess deaths, from 2019 wk1

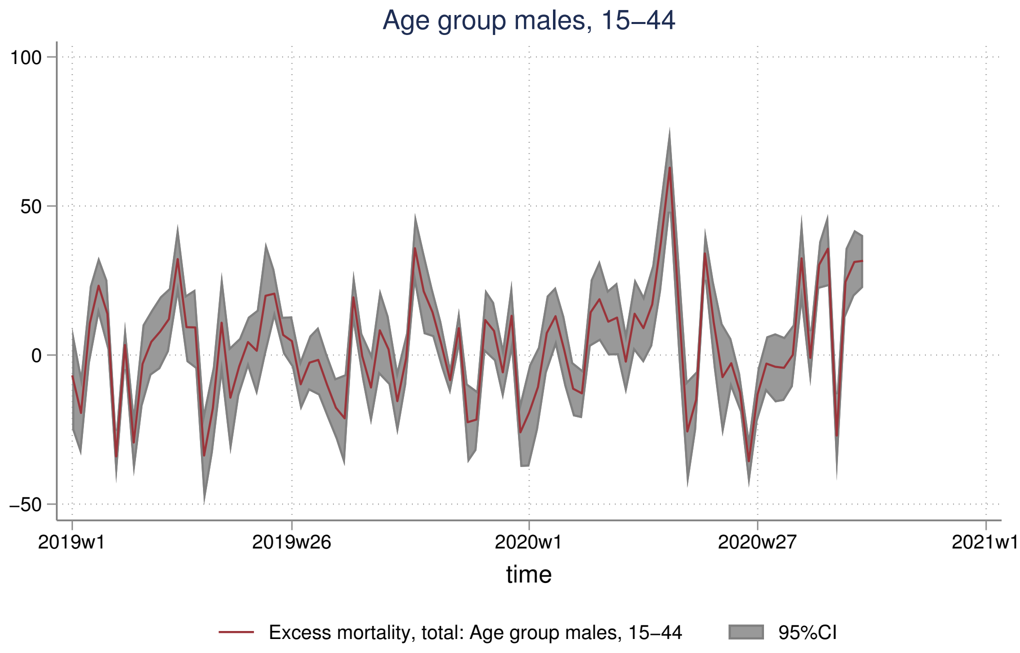

Figure 201: Males 15-44 all-cause excess deaths, from 2020 wk1

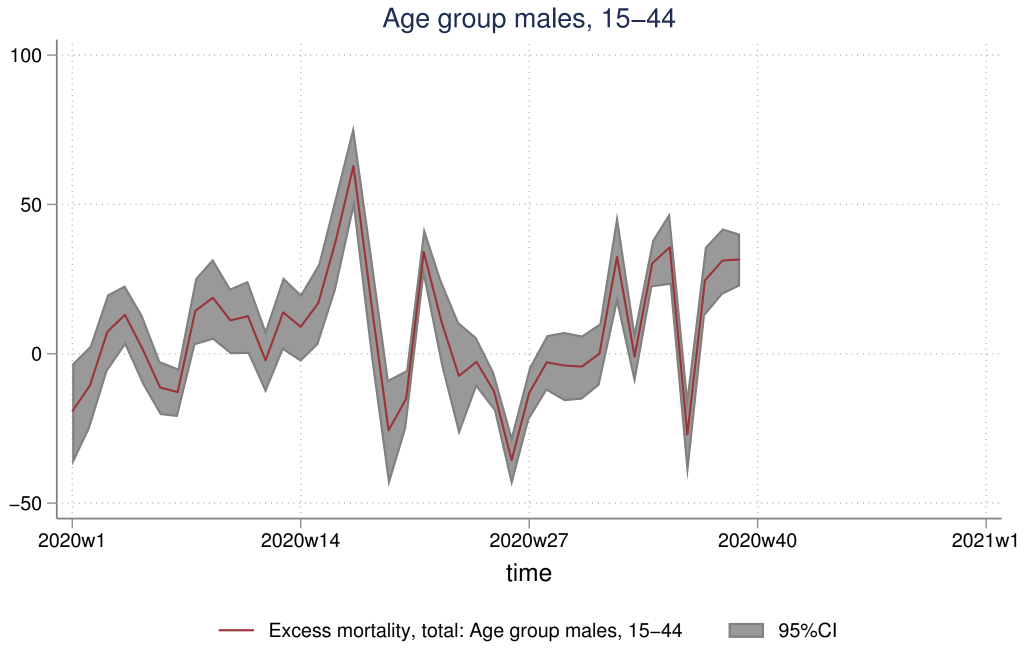

Figure 202: Males 15-44 all-cause excess deaths (−COVID19), from 2010 wk1

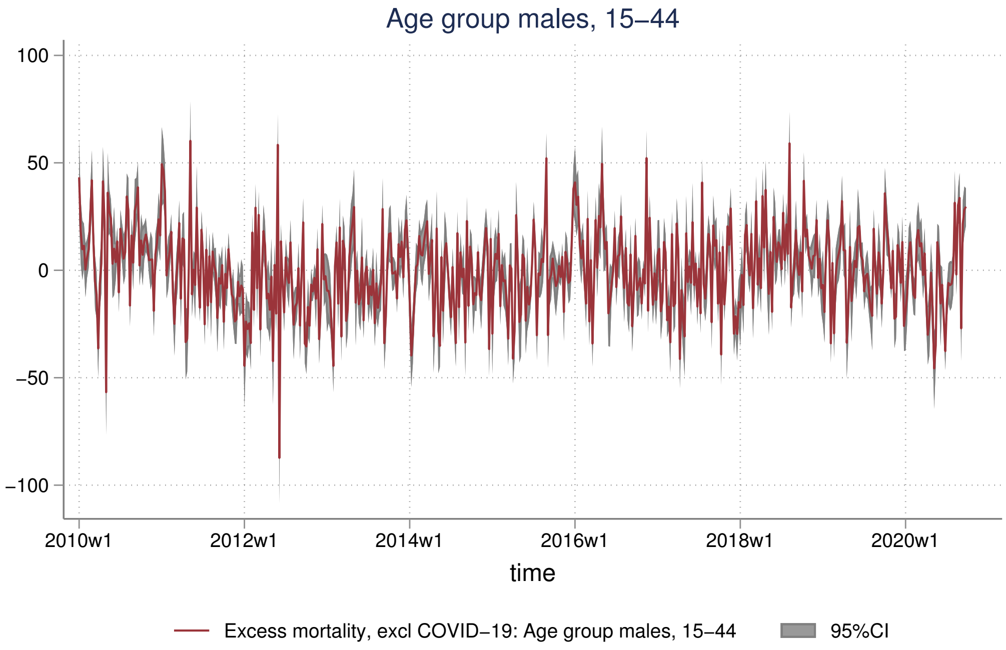

Figure 203: Males 15-44 all-cause excess deaths (−COVID19), from 2019 wk1

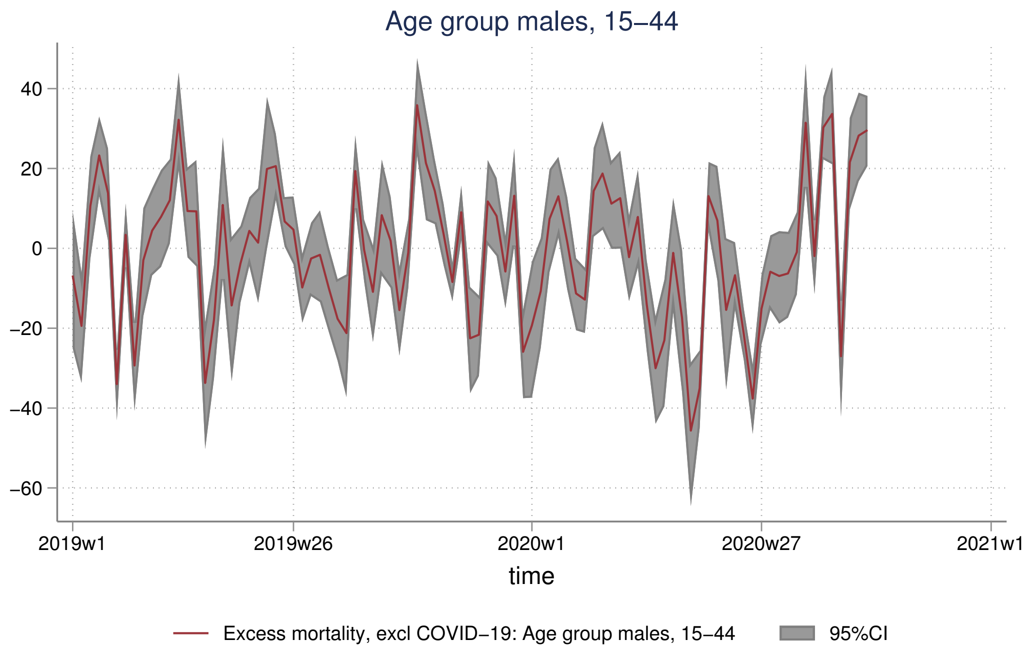

Figure 204: Males 15-44 all-cause excess deaths (−COVID19), from 2020 wk1

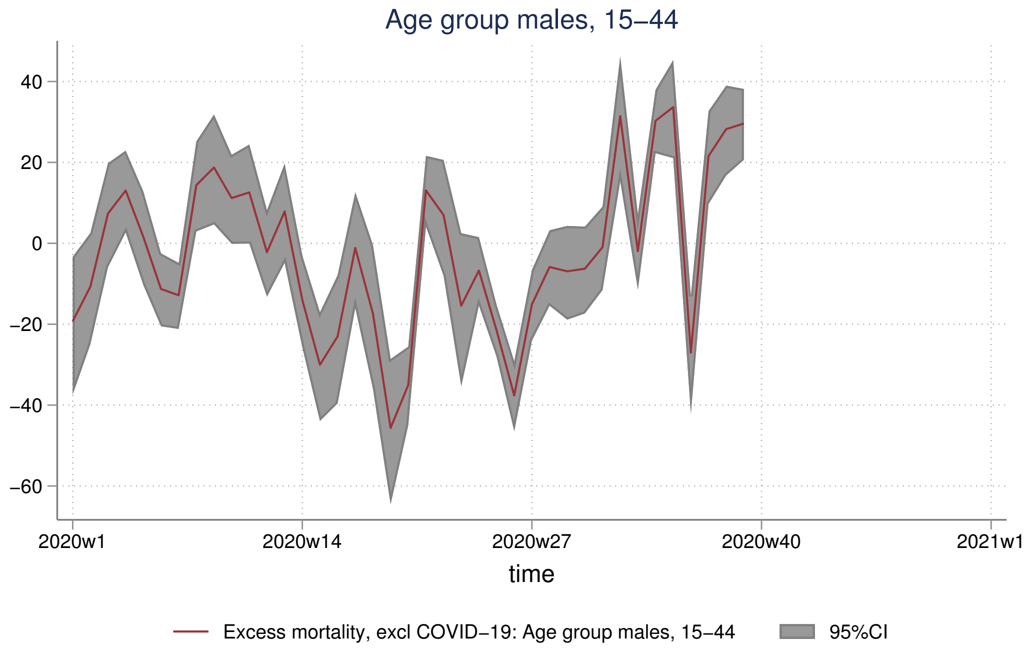

4.5 Males aged 45-64

Figure 205: Males 45-64 mortality time trend and model, from 2010 wk1

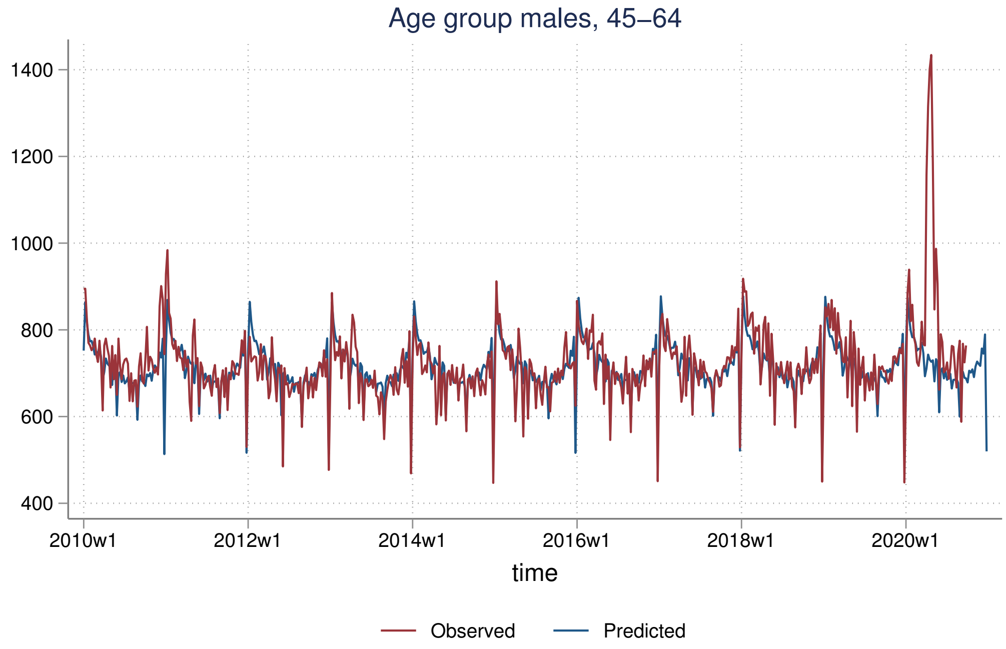

Figure 206: Males 45-64 mortality time trend and model, from 2019 wk1

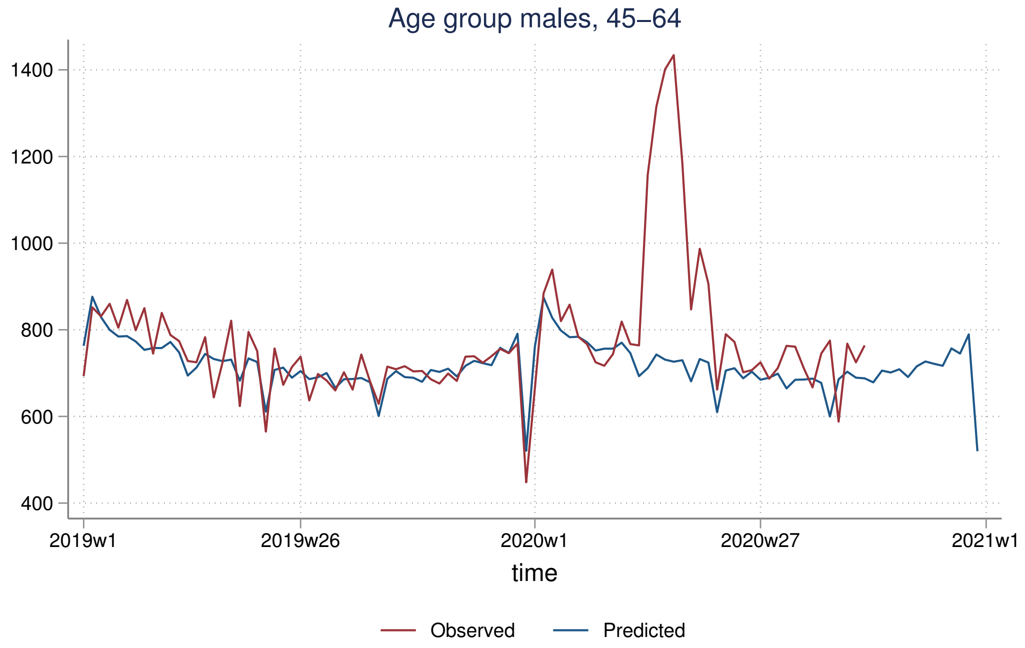

Figure 207: Males 45-64 mortality time trend and model, from 2020 wk1

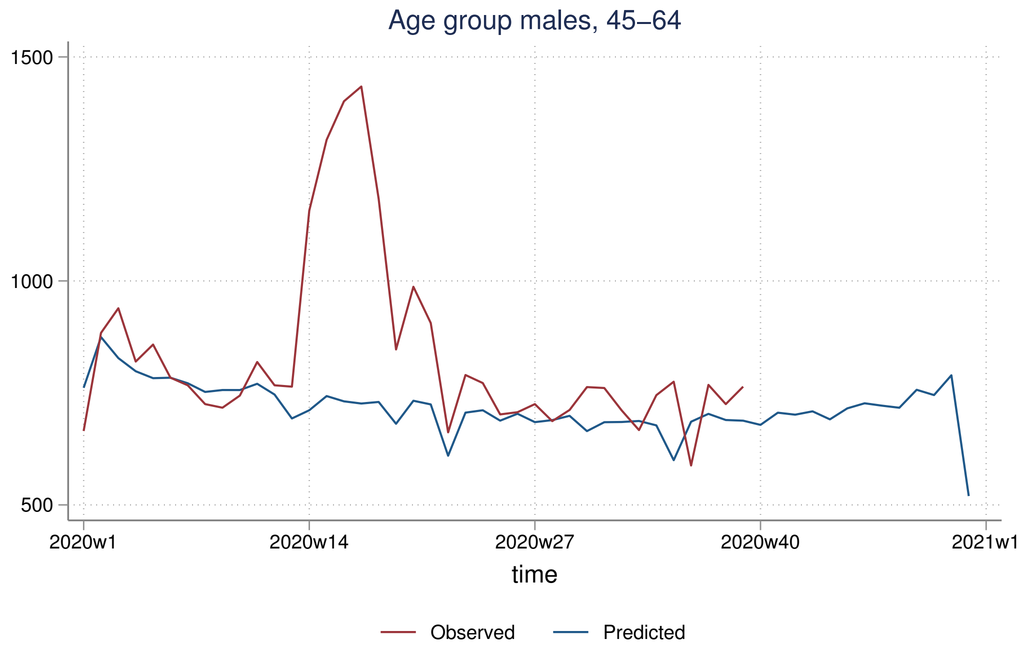

Figure 208: Males 45-64 all-cause excess deaths, from 2010 wk1

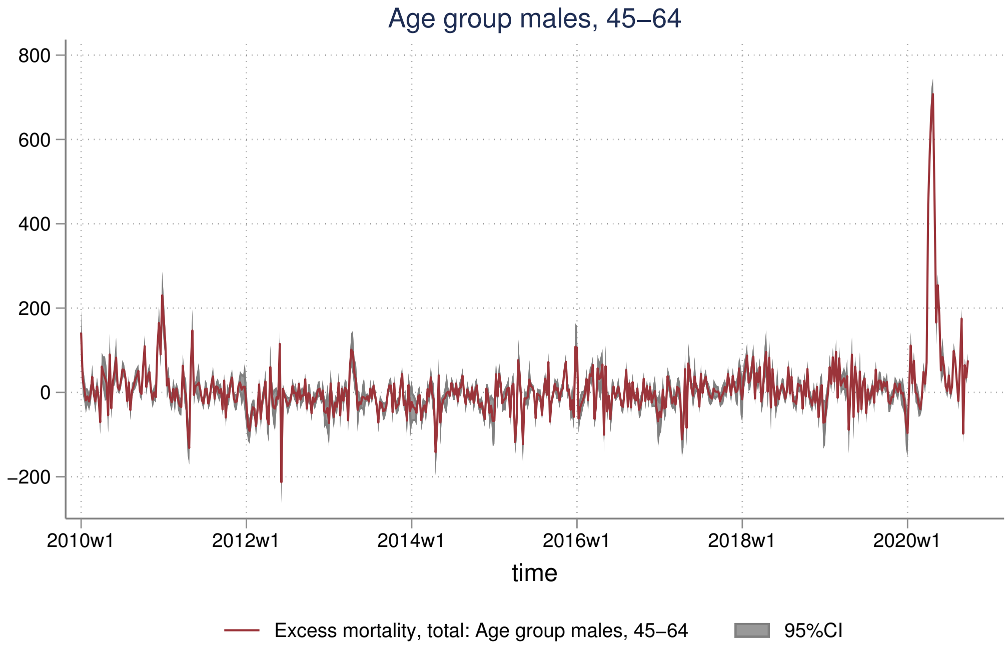

Figure 209: Males 45-64 all-cause excess deaths, from 2019 wk1

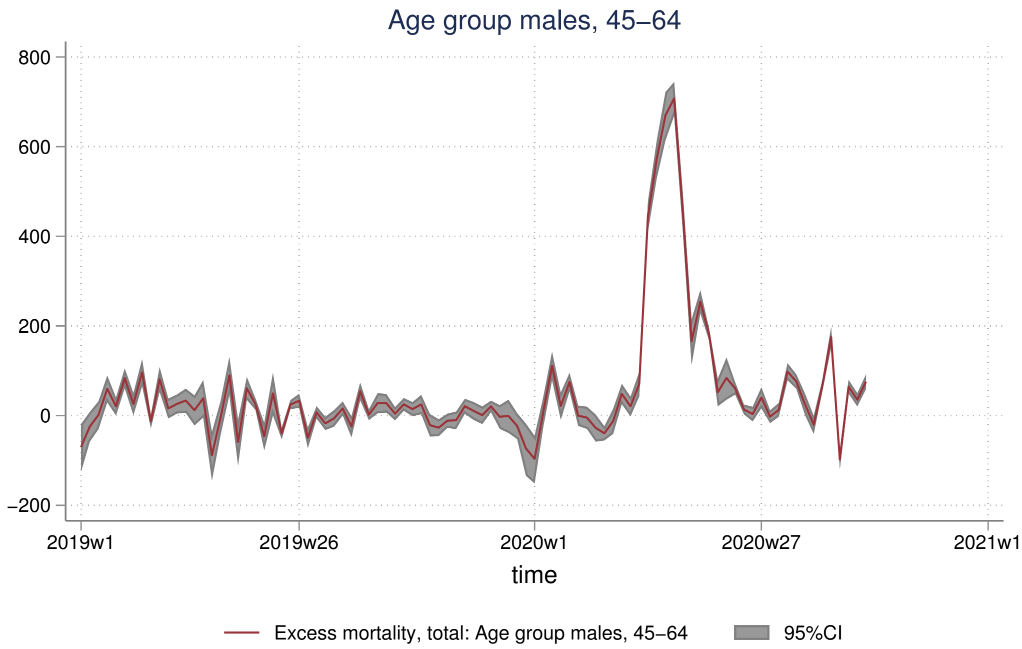

Figure 210: Males 45-64 all-cause excess deaths, from 2020 wk1

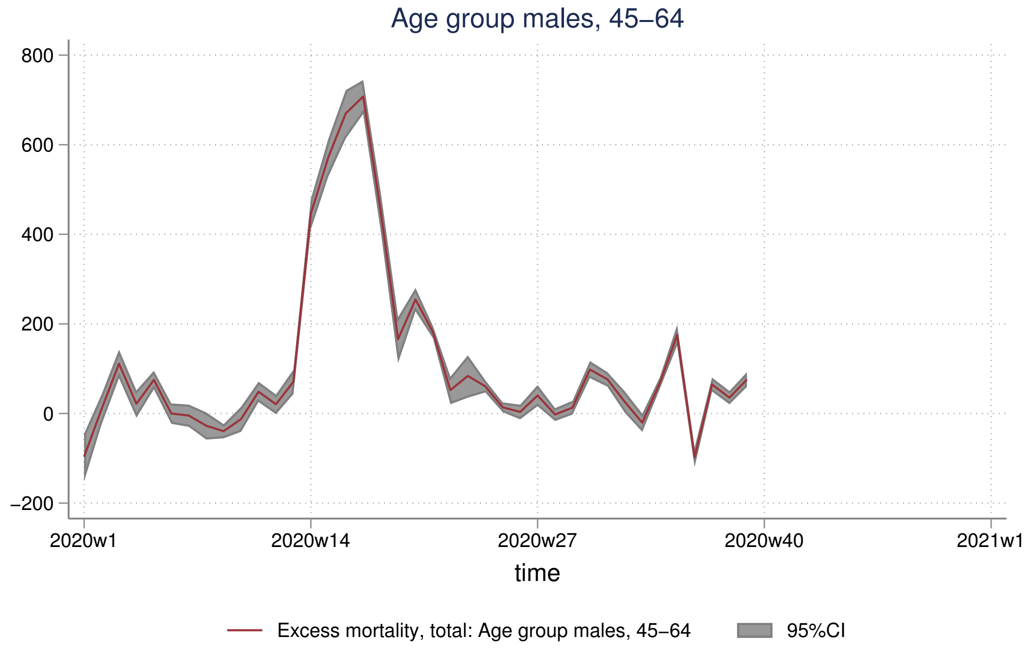

Figure 211: Males 45-64 all-cause excess deaths (−COVID19), from 2010 wk1

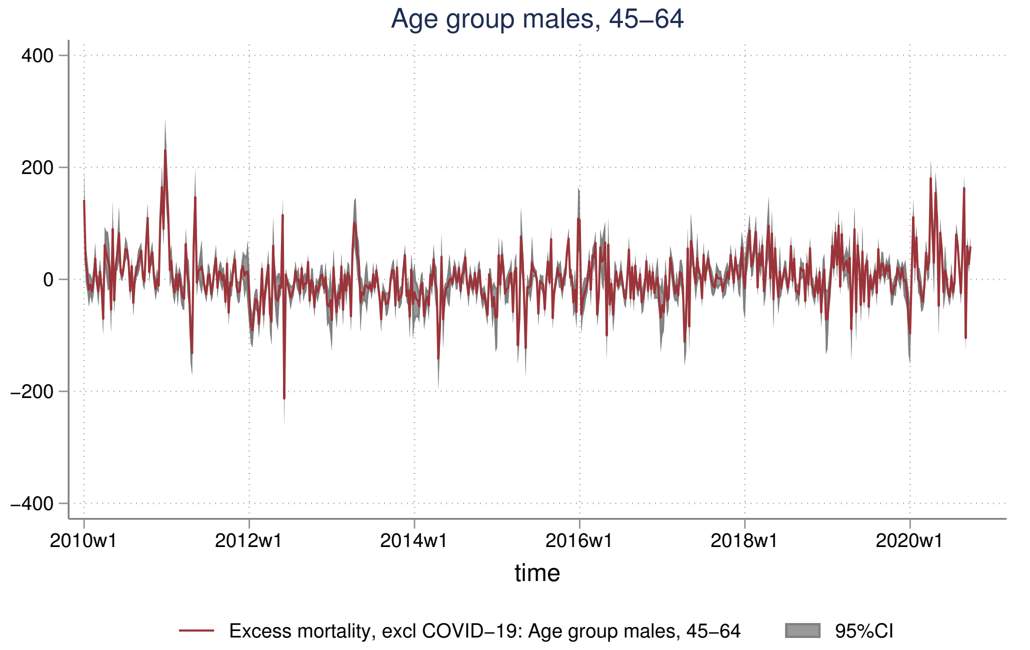

Figure 212: Males 45-64 all-cause excess deaths (−COVID19), from 2019 wk1

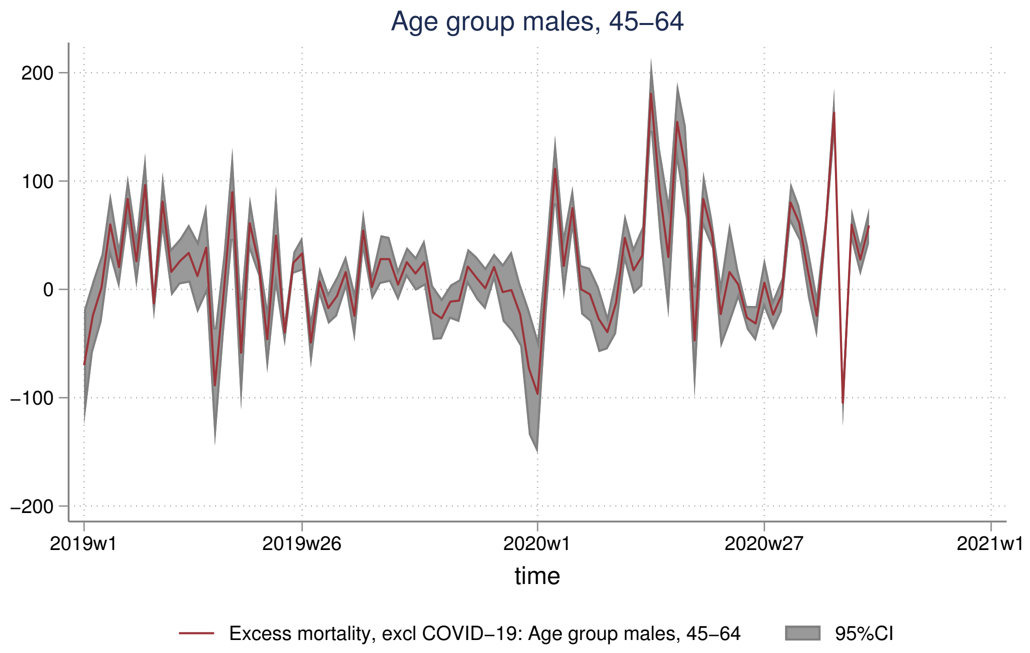

Figure 213: Males 45-64 all-cause excess deaths (−COVID19), from 2020 wk1

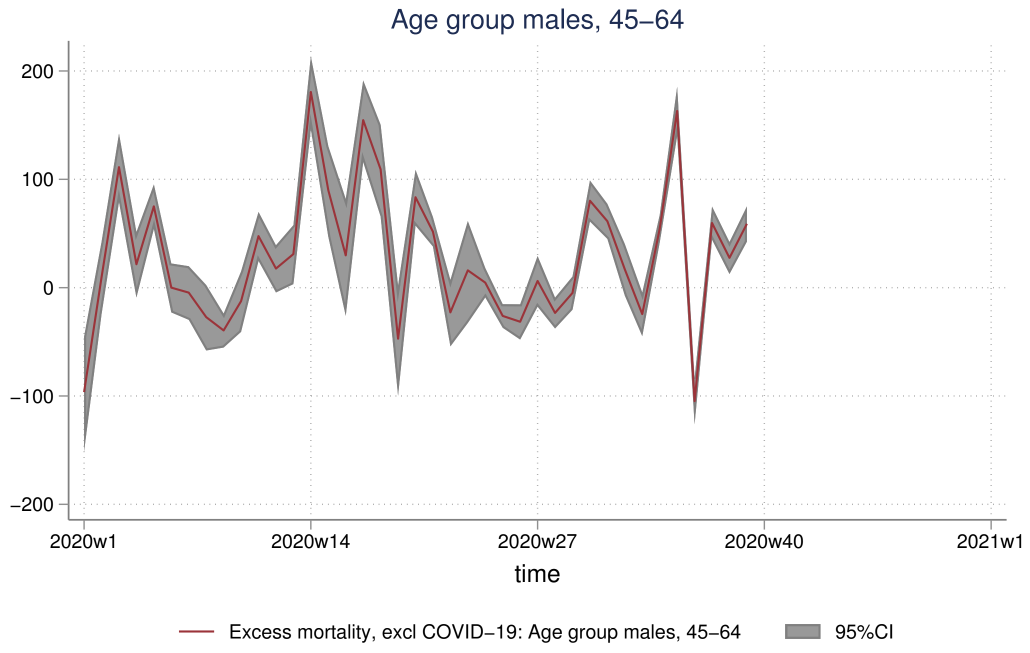

4.6 Males aged 65-74

Figure 214: Males 65-74 mortality time trend and model, from 2010 wk1

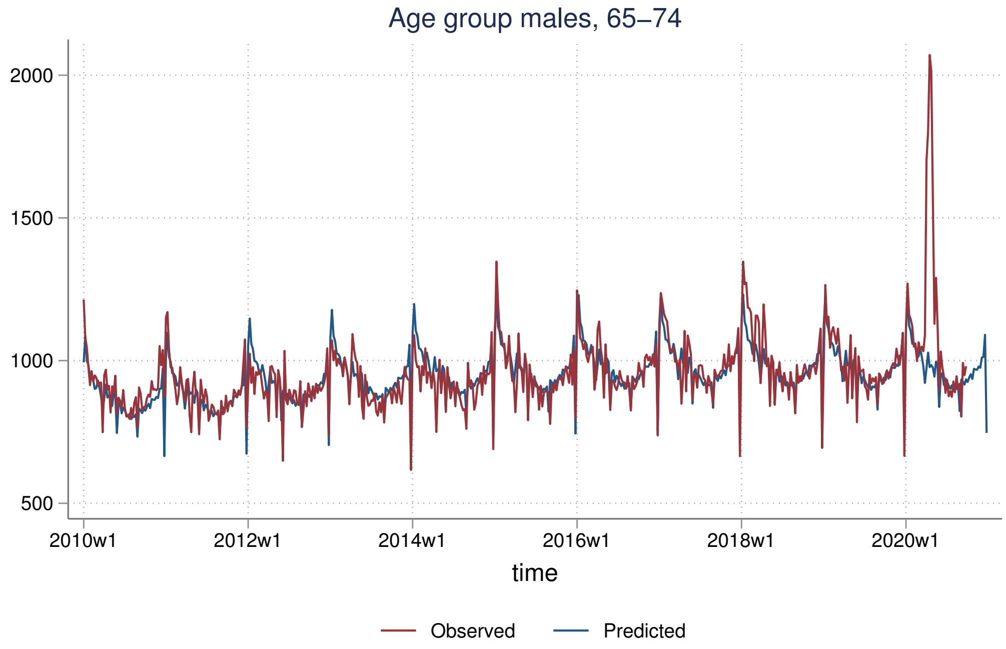

Figure 215: Males 65-74 mortality time trend and model, from 2019 wk1

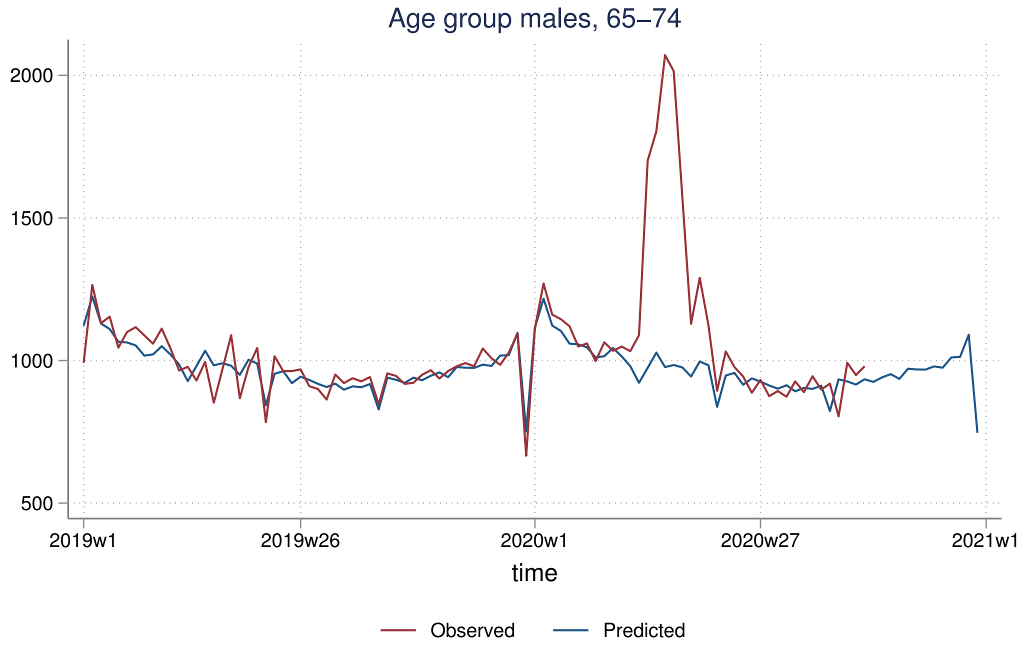

Figure 216: Males 65-74 mortality time trend and model, from 2020 wk1

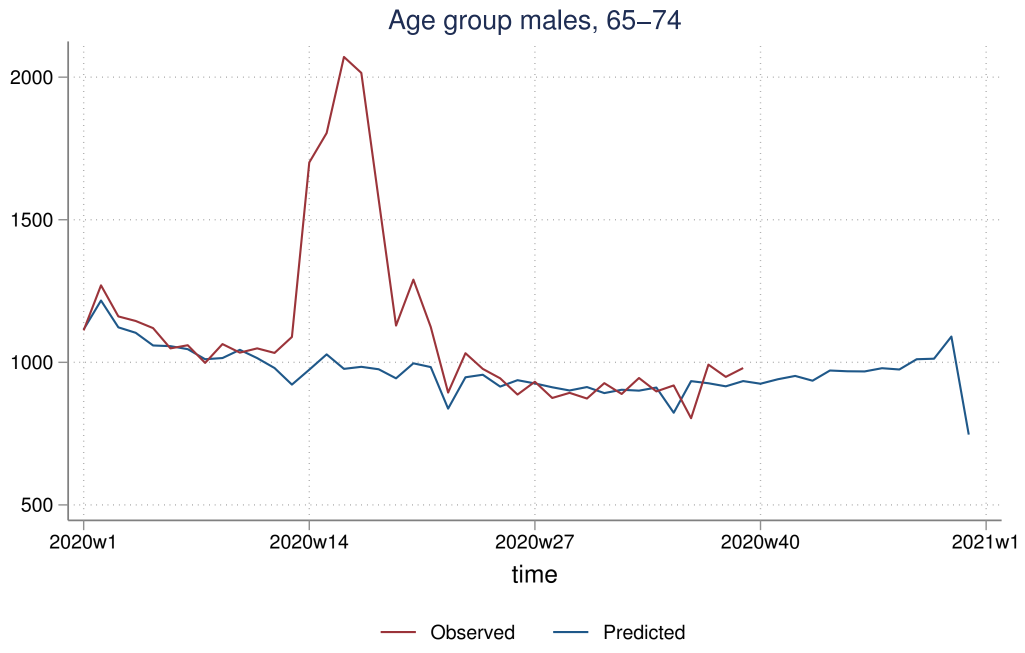

Figure 217: Males 65-74 all-cause excess deaths, from 2010 wk1

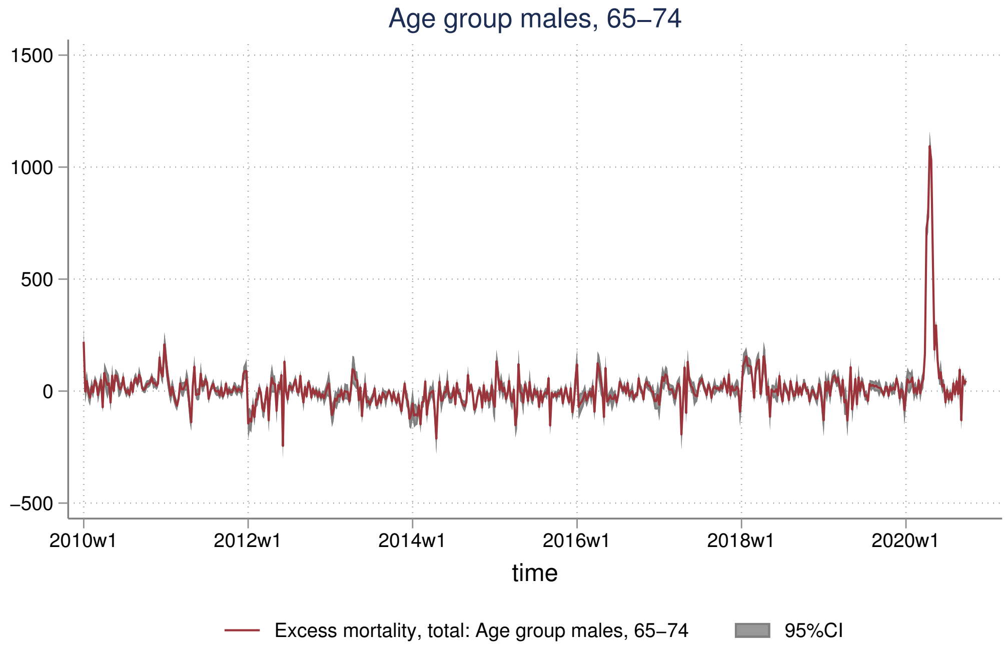

Figure 218: Males 65-74 all-cause excess deaths, from 2019 wk1

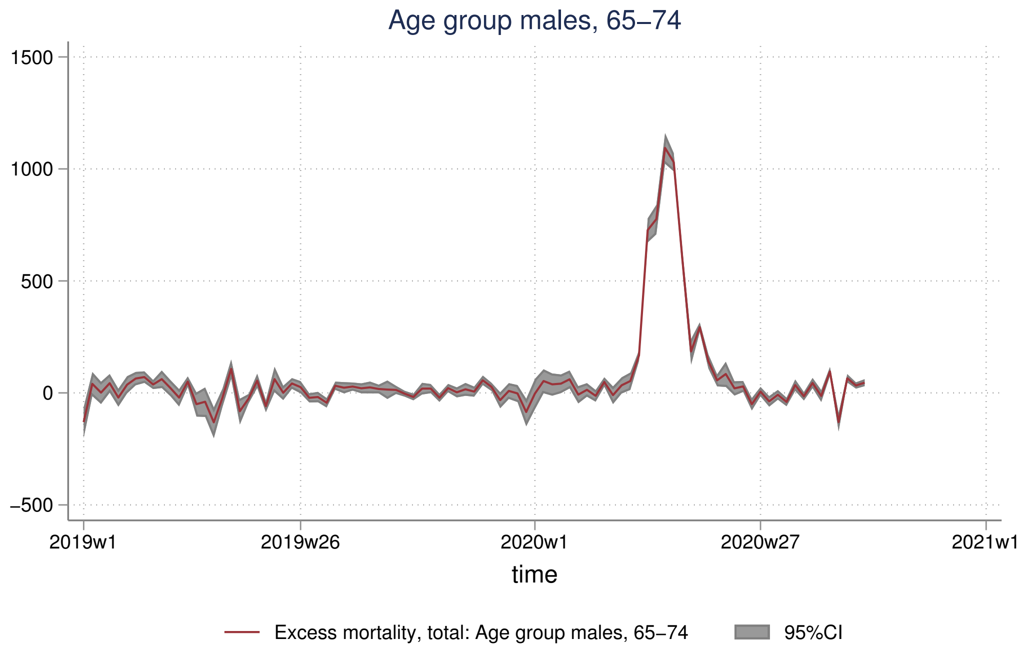

Figure 219: Males 65-74 all-cause excess deaths, from 2020 wk1

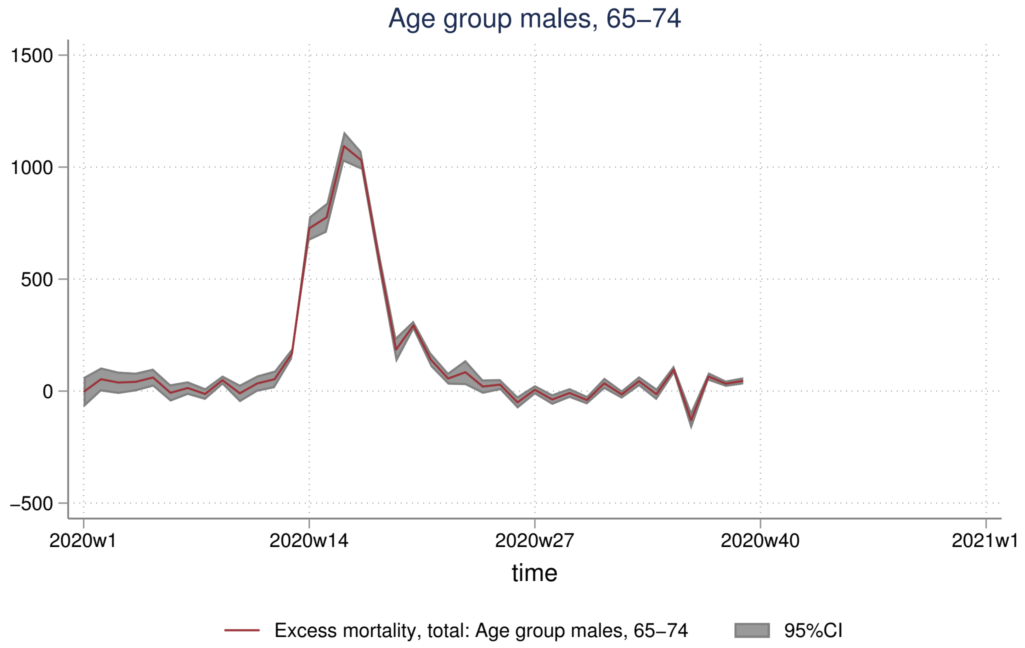

Figure 220: Males 65-74 all-cause excess deaths (−COVID19), from 2010 wk1

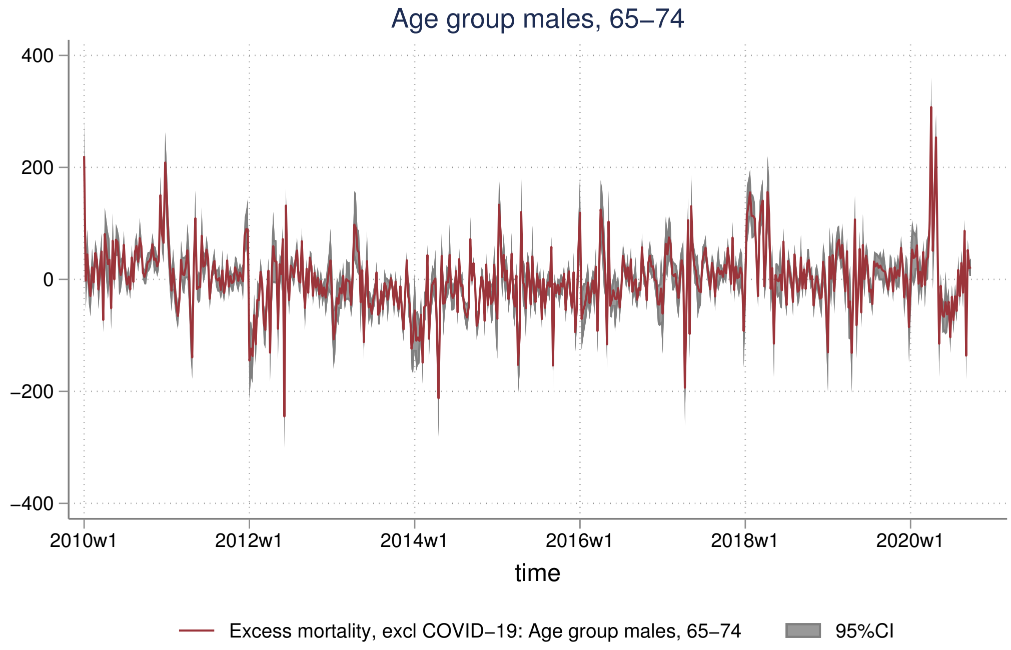

Figure 221: Males 65-74 all-cause excess deaths (−COVID19), from 2019 wk1

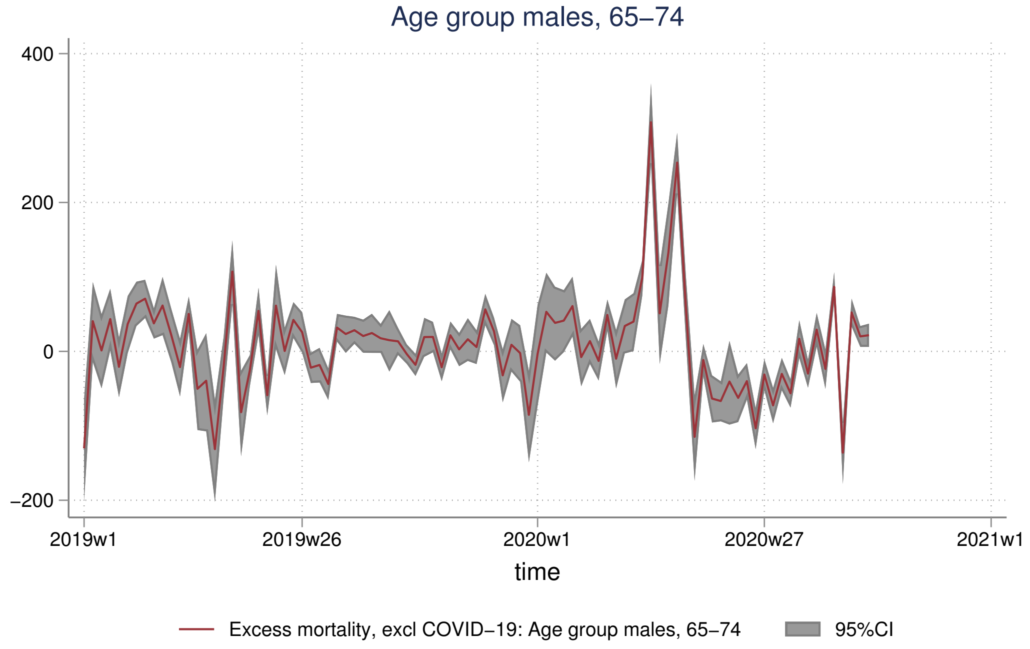

Figure 222: Males 65-74 all-cause excess deaths (−COVID19), from 2020 wk1

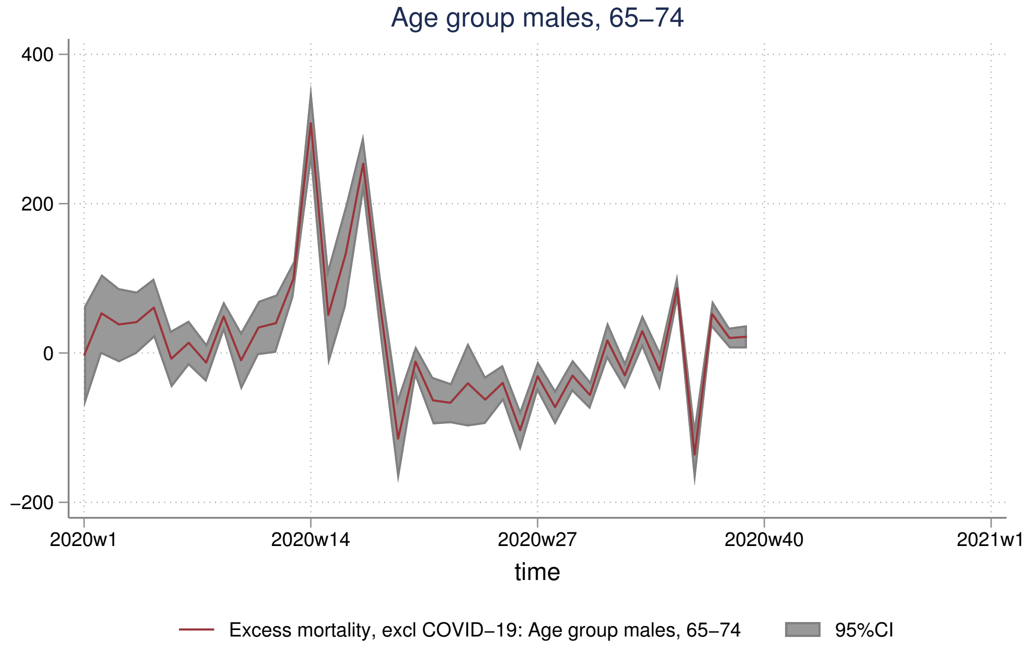

4.7 Males aged 75-84

Figure 223: Males 75-84 mortality time trend and model, from 2010 wk1

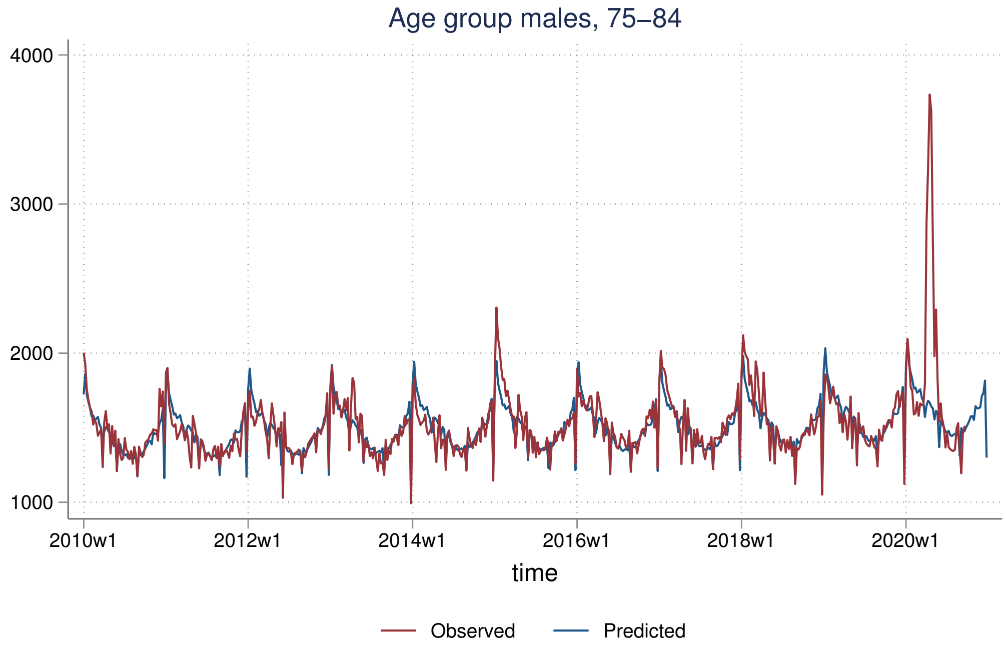

Figure 224: Males 75-84 mortality time trend and model, from 2019 wk1

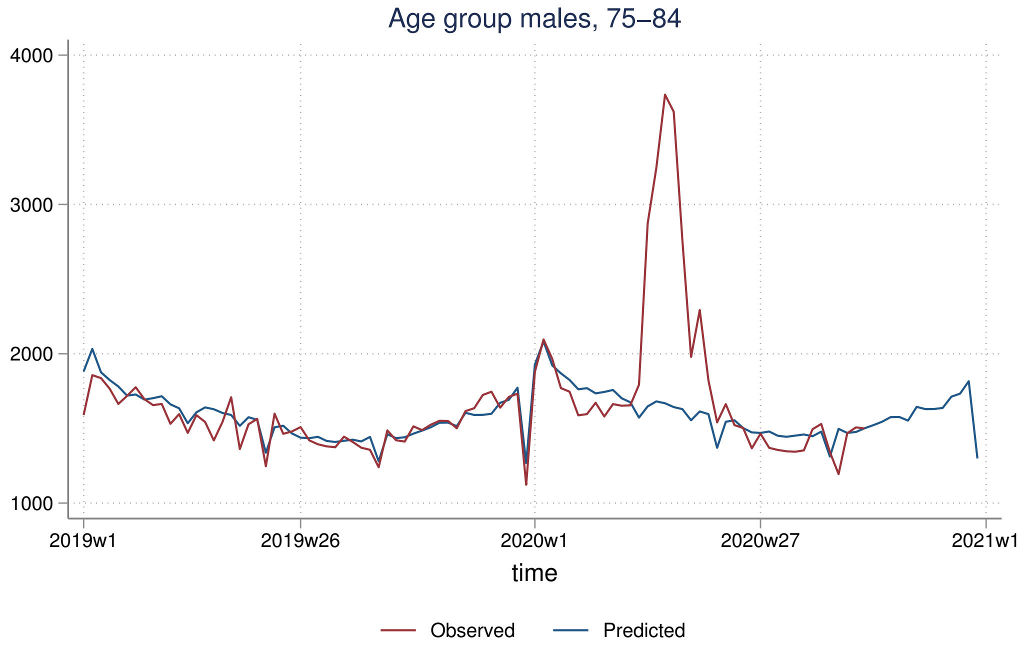

Figure 225: Males 75-84 mortality time trend and model, from 2020 wk1

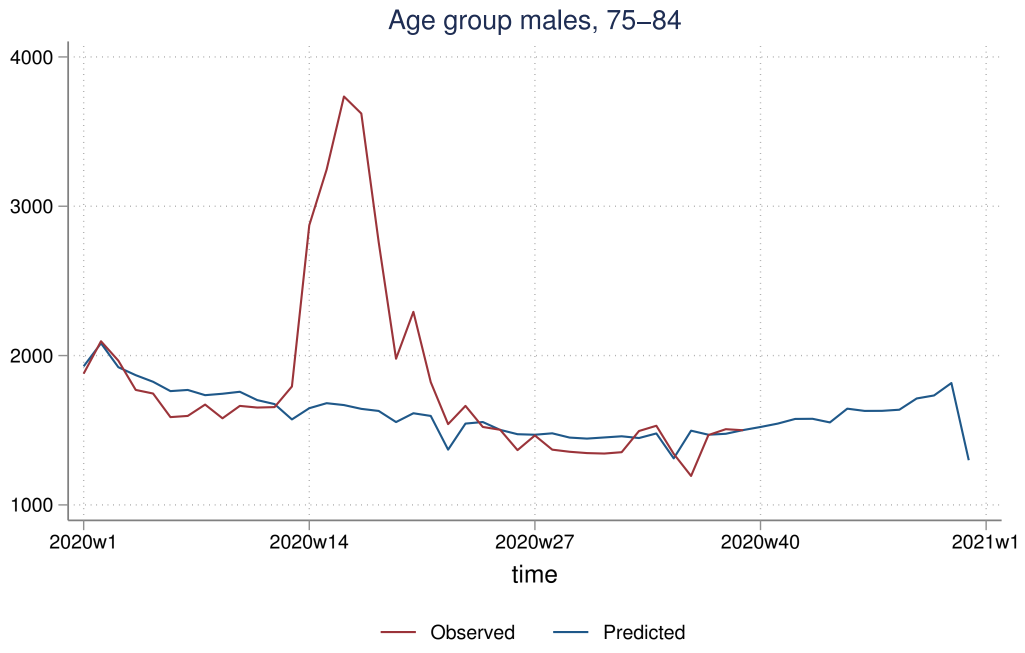

Figure 226: Males 75-84 all-cause excess deaths, from 2010 wk1

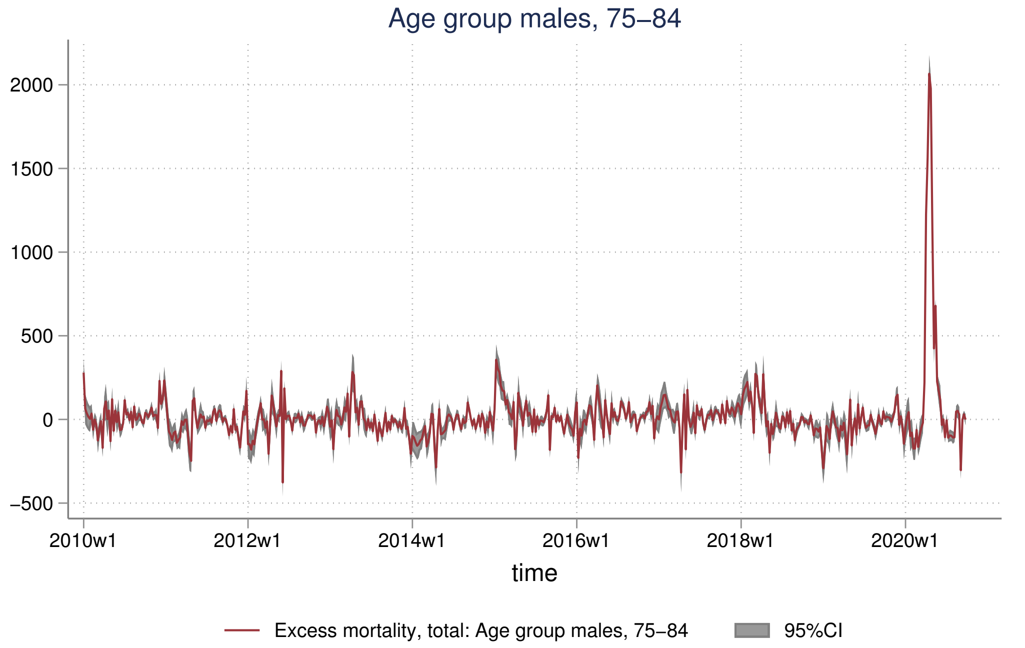

Figure 227: Males 75-84 all-cause excess deaths, from 2019 wk1

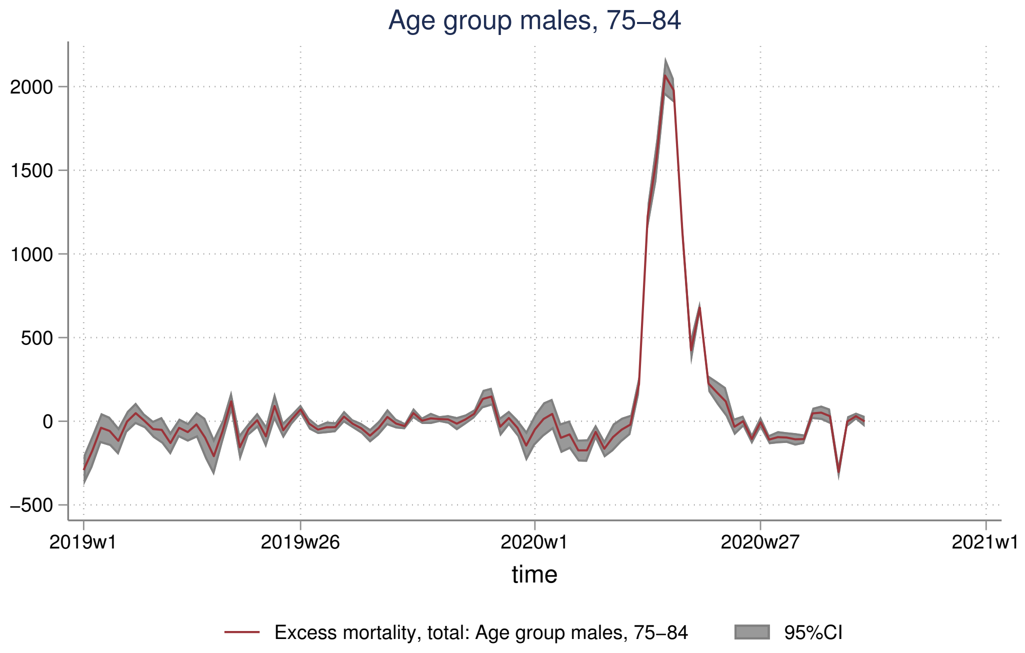

Figure 228: Males 75-84 all-cause excess deaths, from 2020 wk1

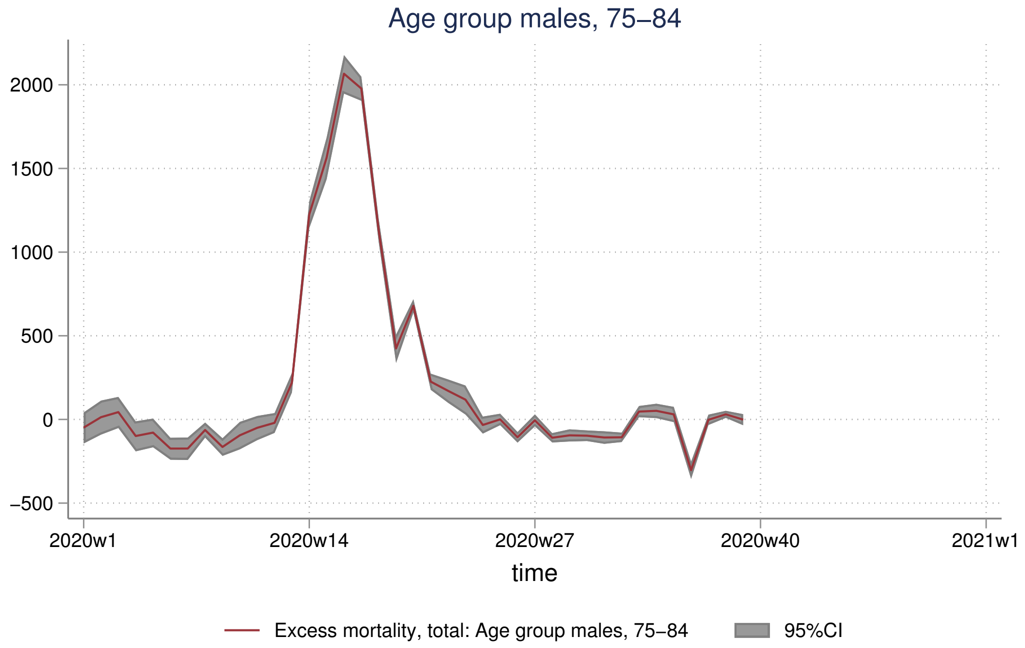

Figure 229: Males 75-84 all-cause excess deaths (−COVID19), from 2010 wk1

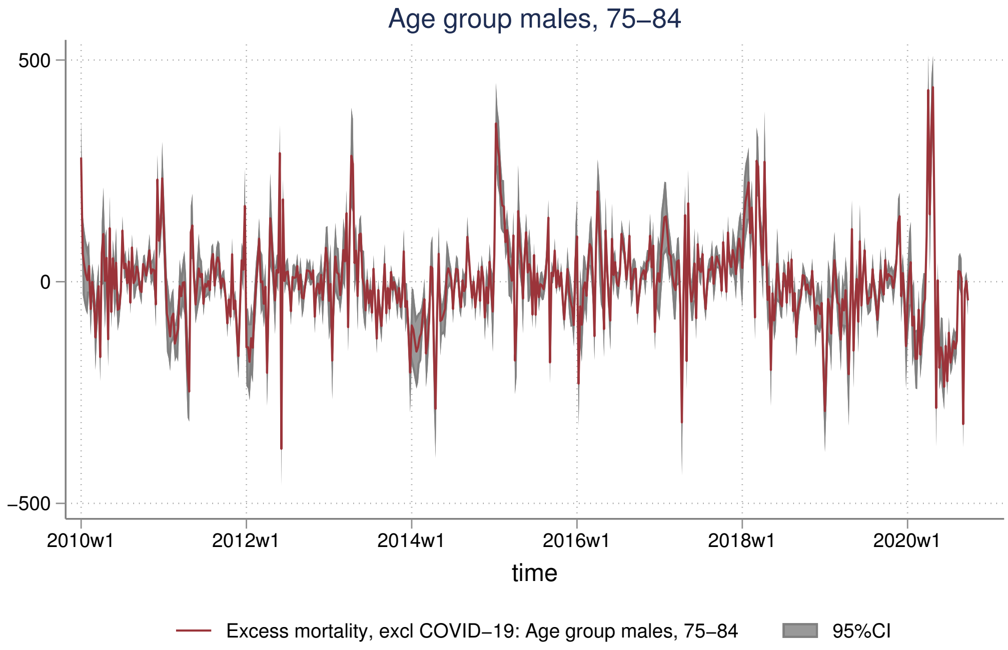

Figure 230: Males 75-84 all-cause excess deaths (−COVID19), from 2019 wk1

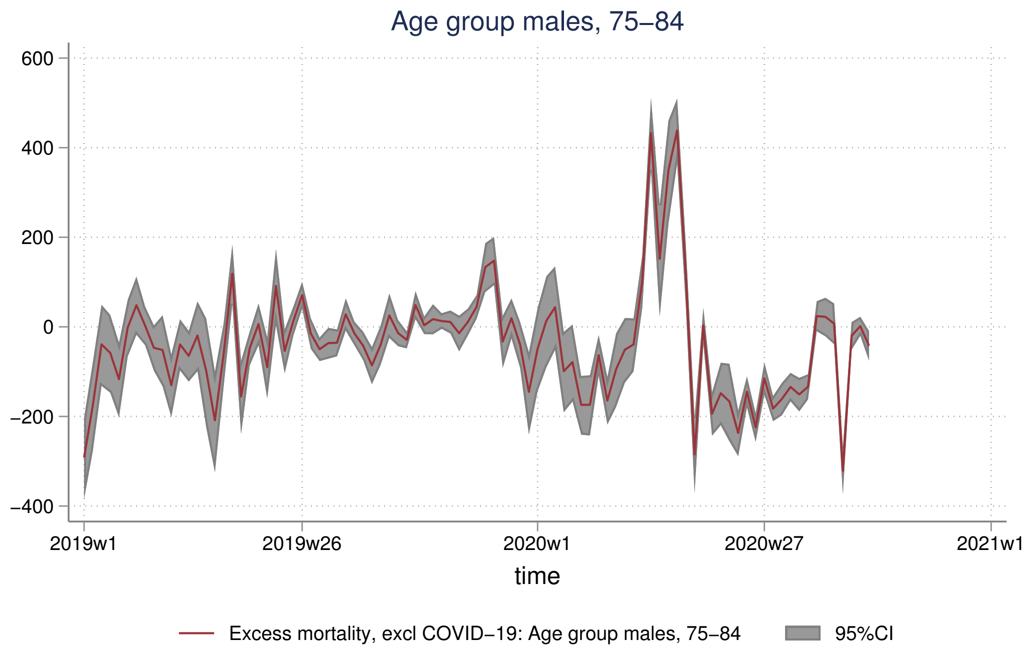

Figure 231: Males 75-84 all-cause excess deaths (−COVID19), from 2020 wk1

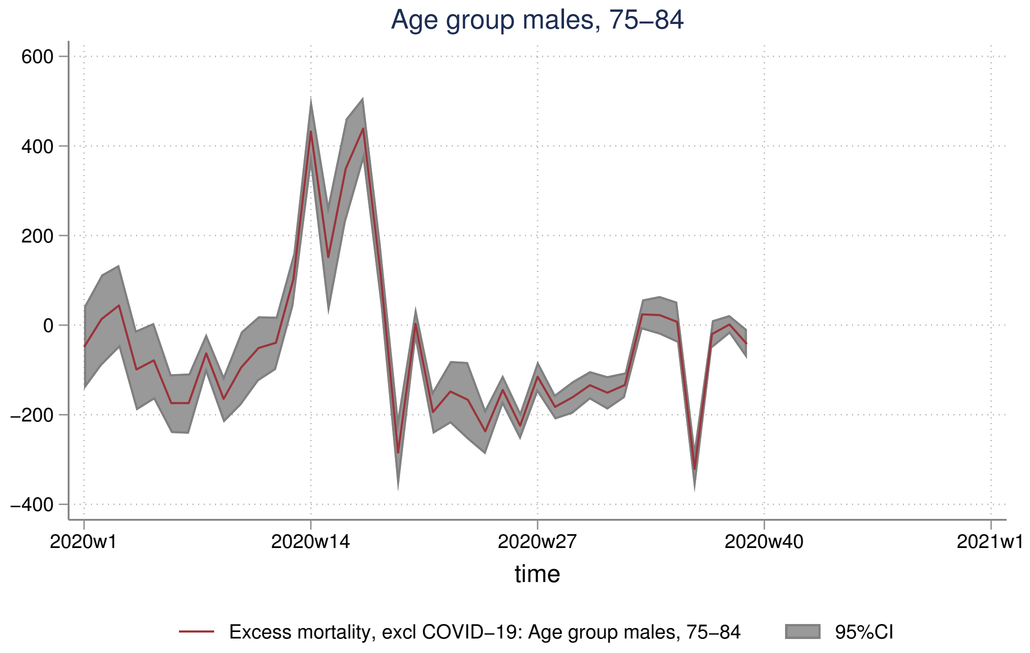

4.8 Males aged 85+

Figure 232: Males 85+ mortality time trend and model, from 2010 wk1

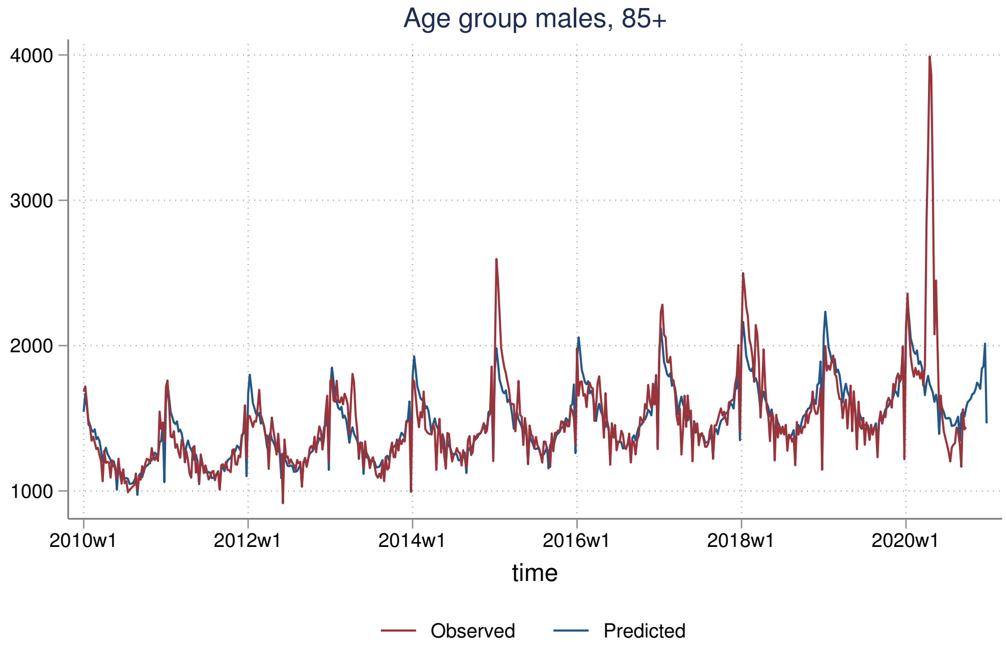

Figure 233: Males 85+ mortality time trend and model, from 2019 wk1

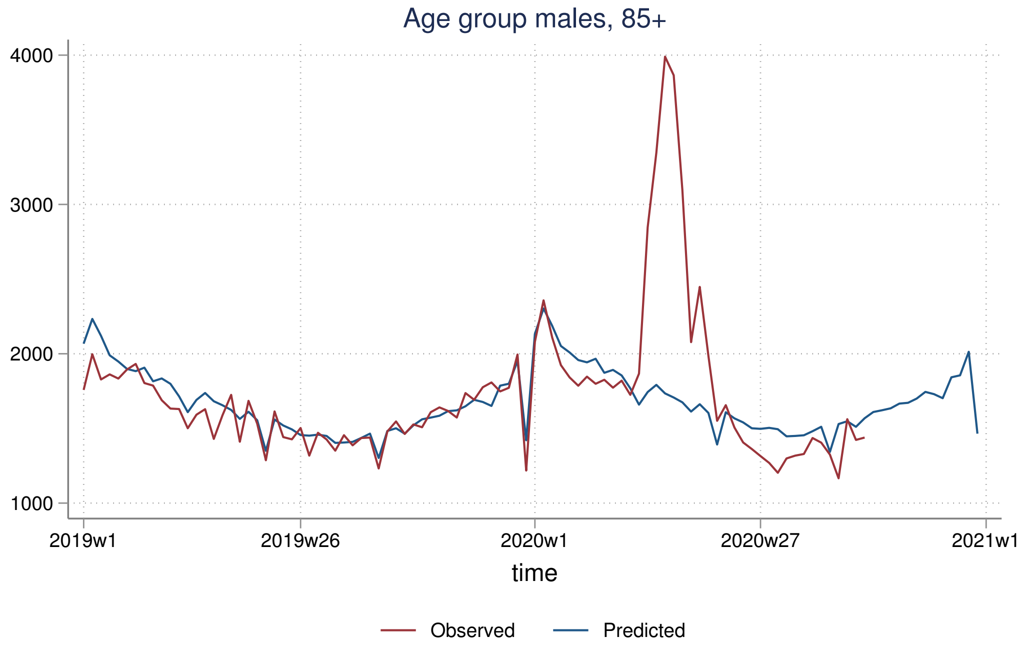

Figure 234: Males 85+ mortality time trend and model, from 2020 wk1

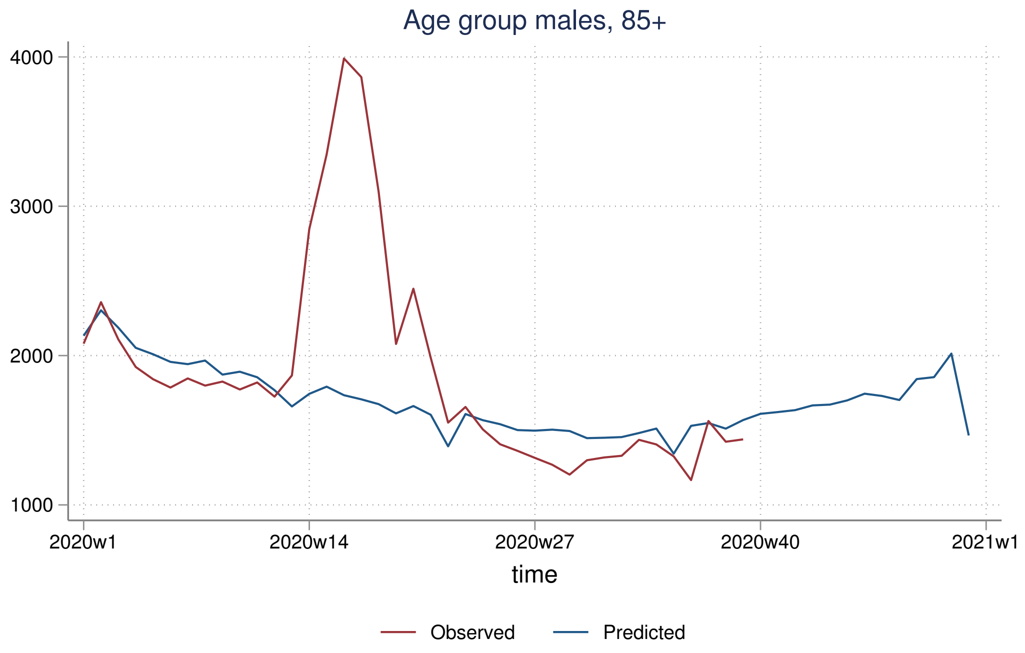

Figure 235: Males 85+ all-cause excess deaths, from 2010 wk1

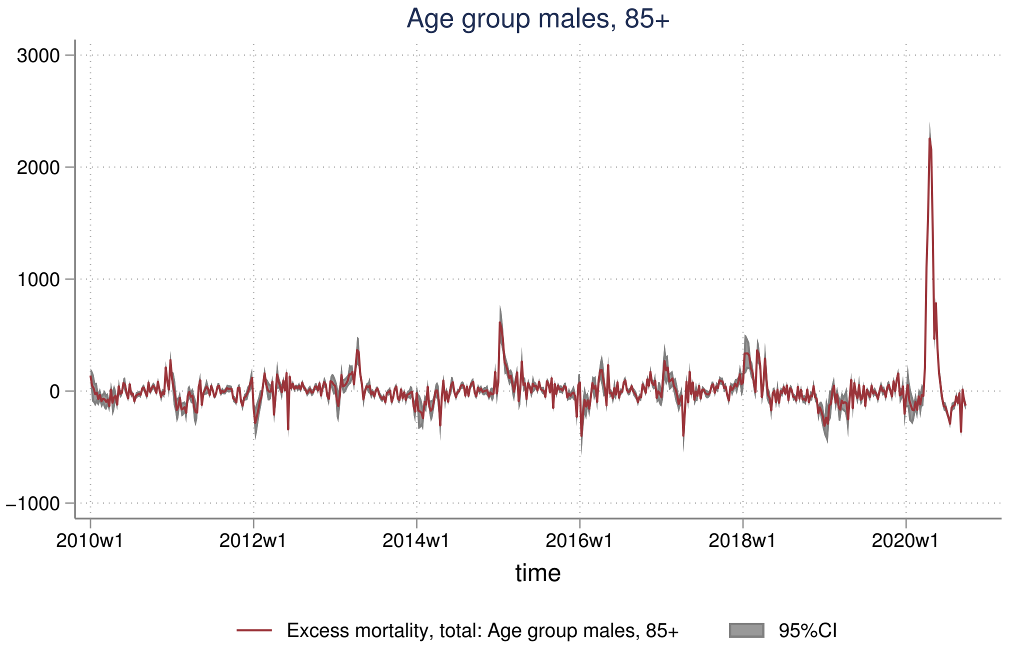

Figure 236: Males 85+ all-cause excess deaths, from 2019 wk1

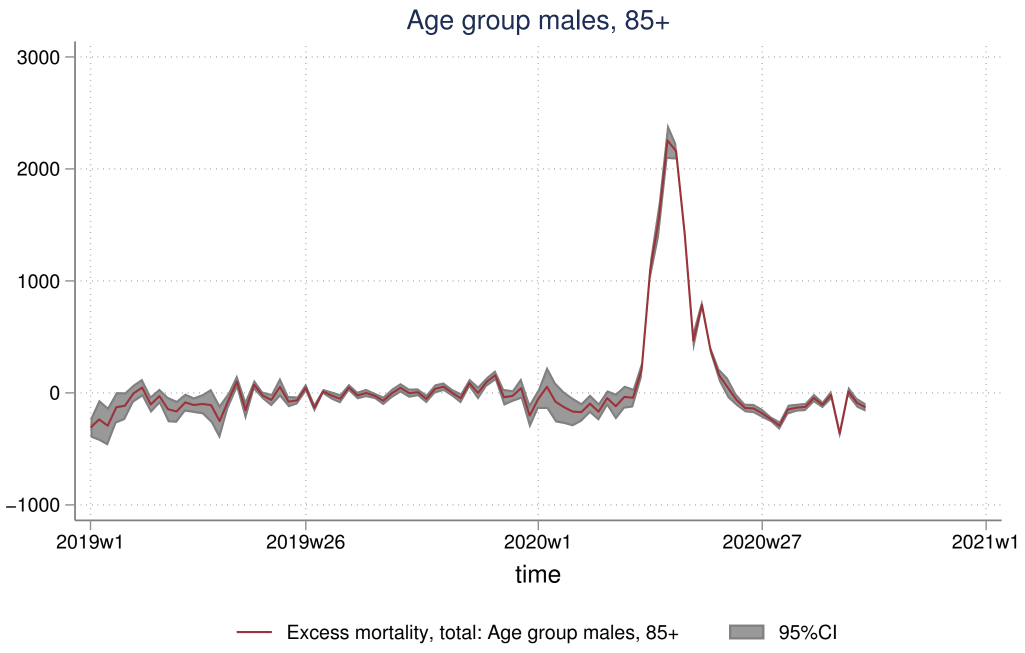

Figure 237: Males 85+ all-cause excess deaths, from 2020 wk1

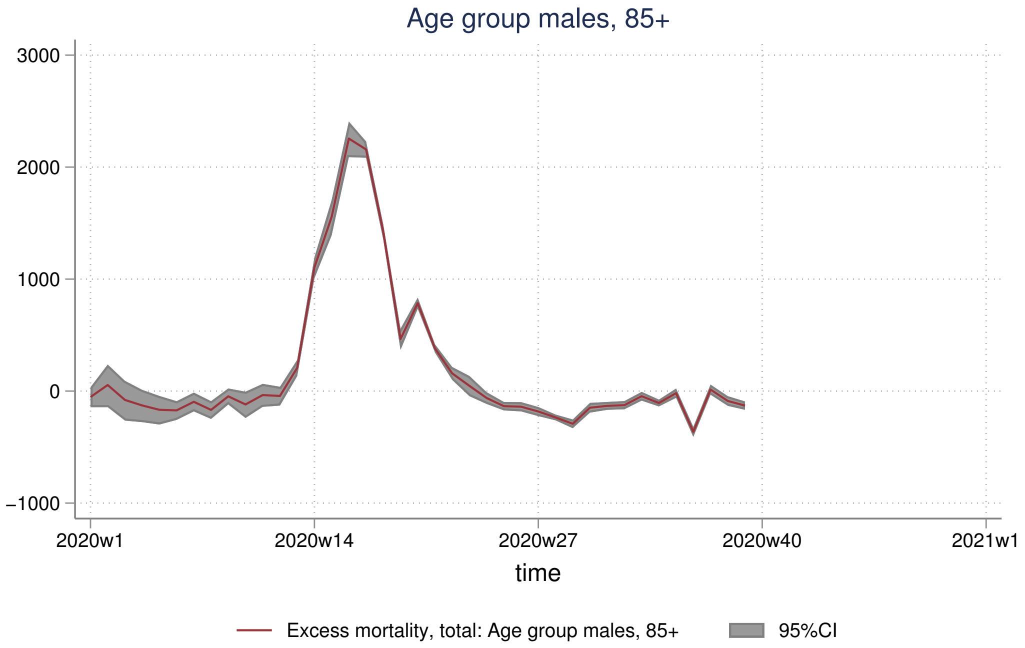

Figure 238: Males 85+ all-cause excess deaths (−COVID19), from 2010 wk1

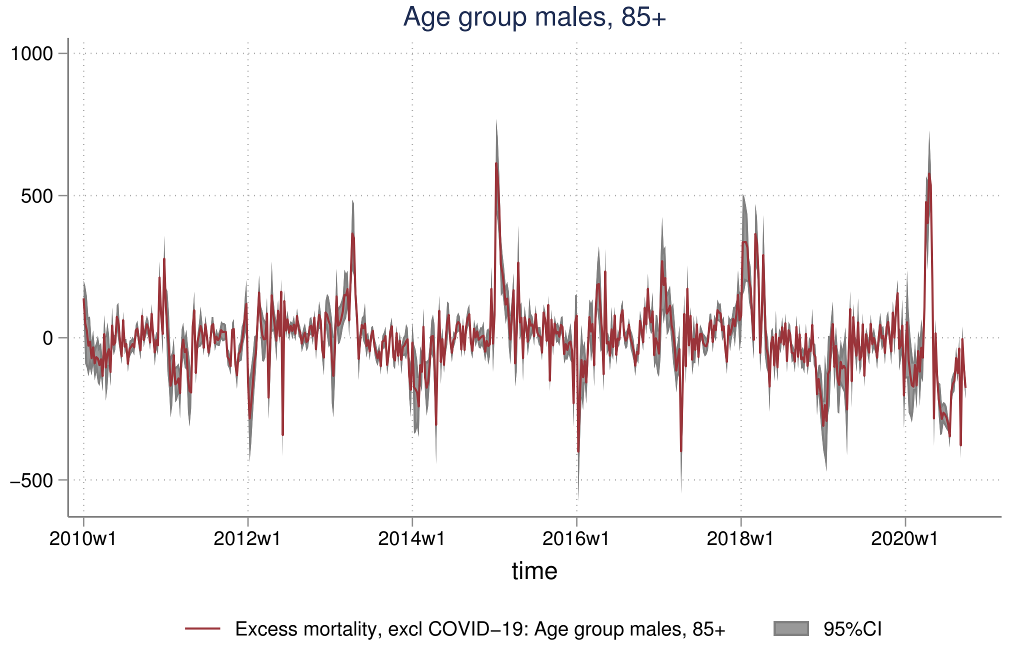

Figure 239: Males 85+ all-cause excess deaths (−COVID19), from 2019 wk1

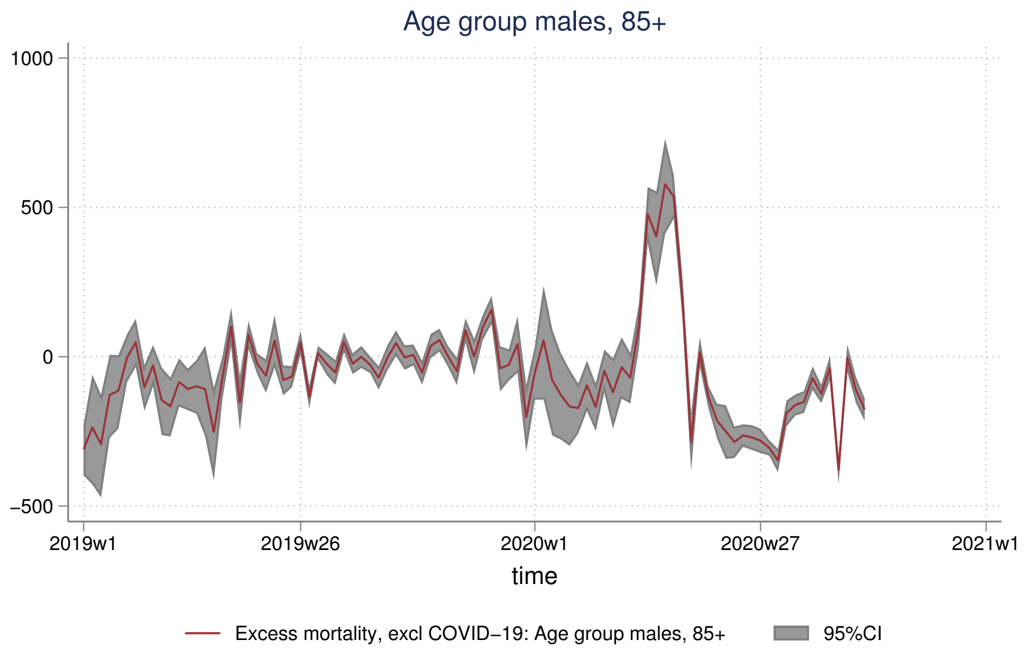

Figure 240: Males 85+ all-cause excess deaths (−COVID19), from 2020 wk1

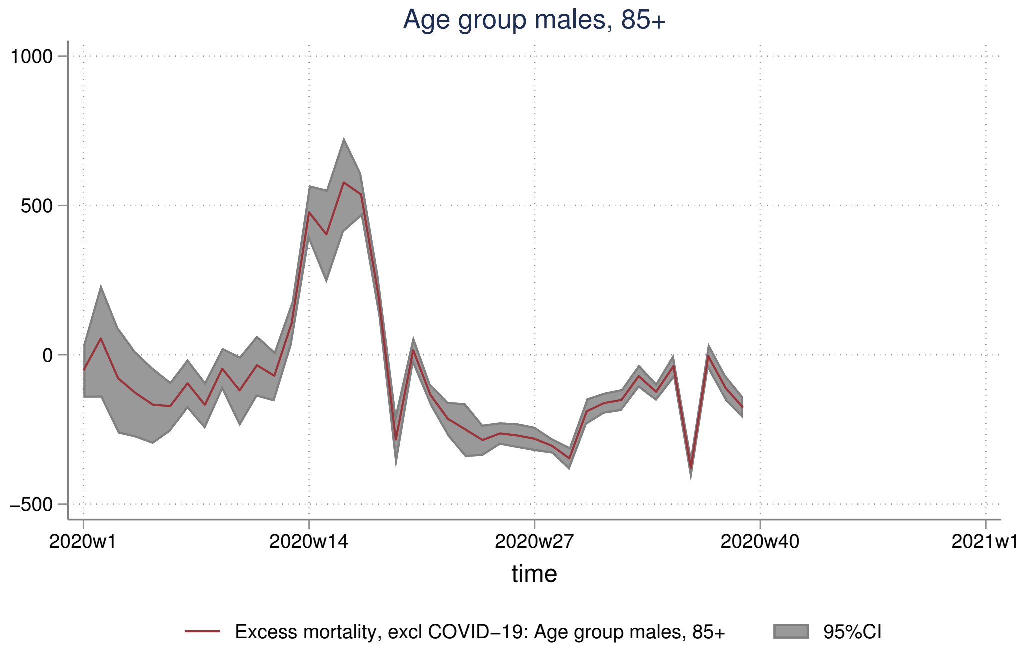

5 Age groups, females

5.1 Time trends

Figure 241: Female age group mortality time trends, fm 2010 wk1

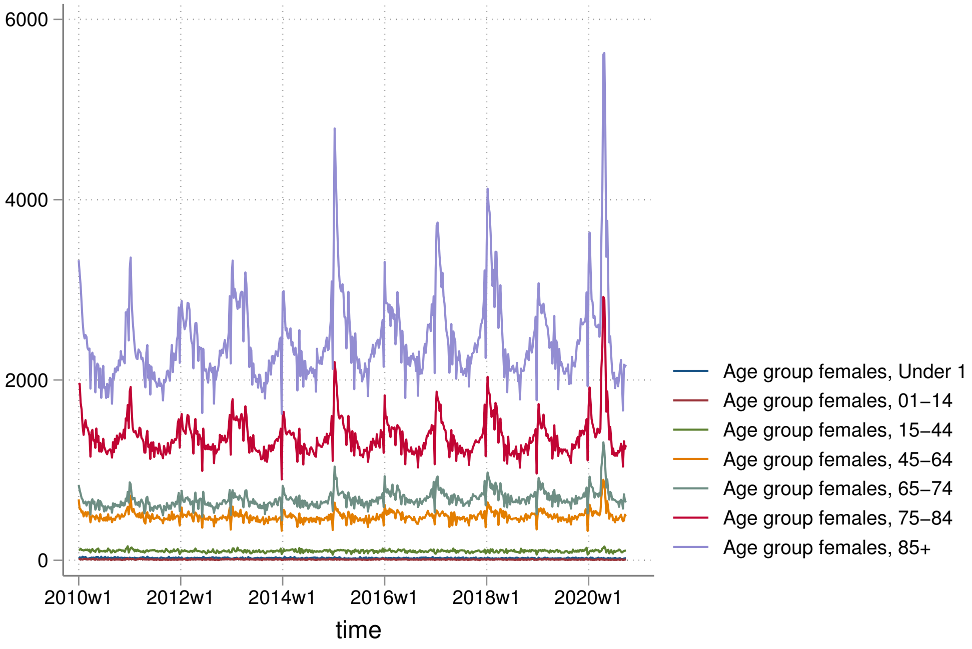

Figure 242: Female age group mortality time trends, fm 2019 wk1

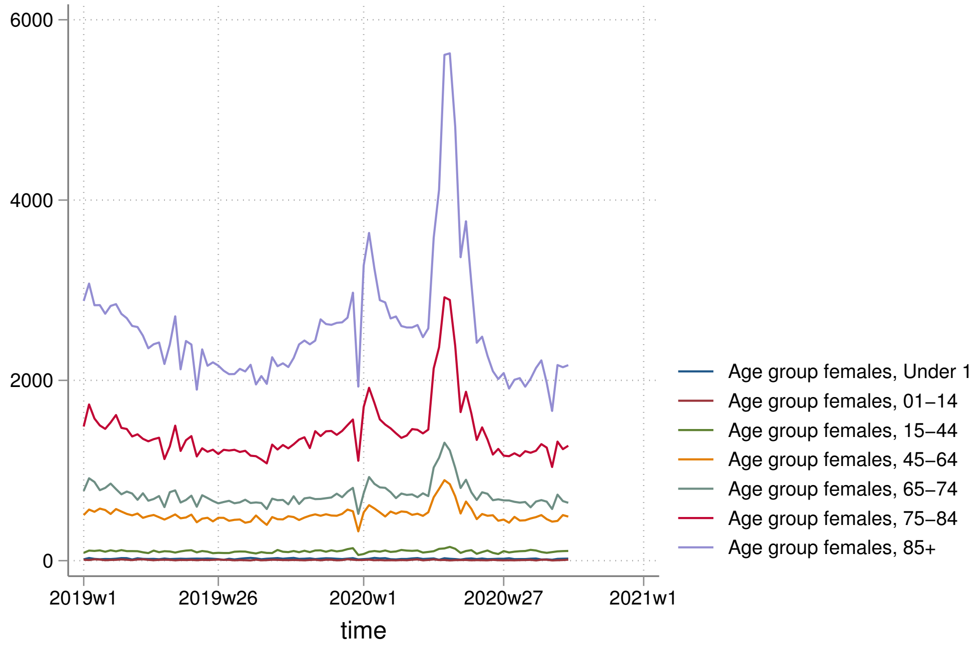

Figure 243: Female age group mortality time trends, fm 2020 wk1

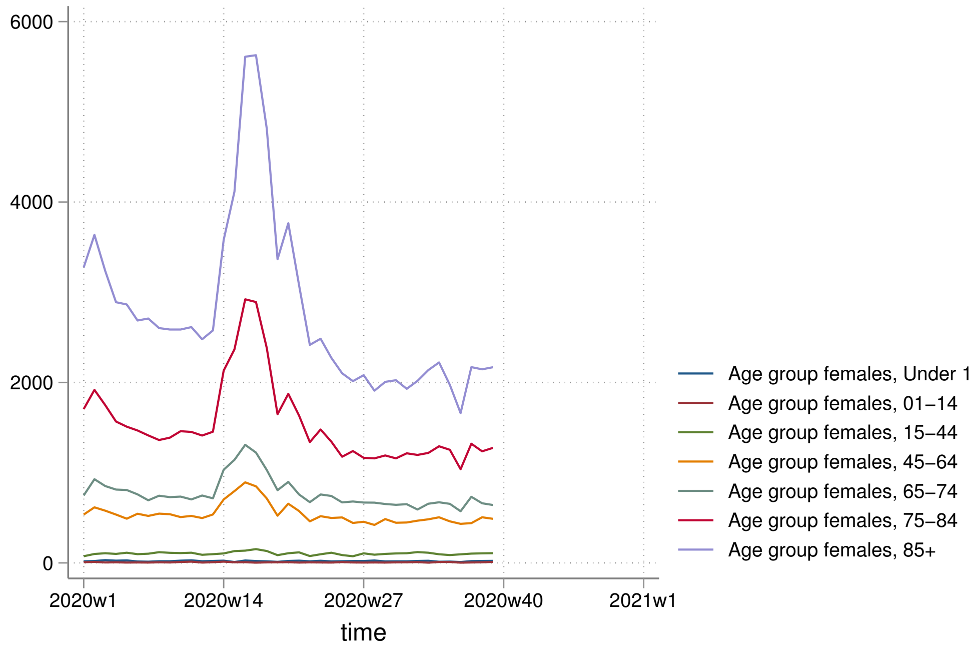

5.2 Females aged under 1

Figure 244: Females < 1 mortality time trend and model, fm 2010 wk1

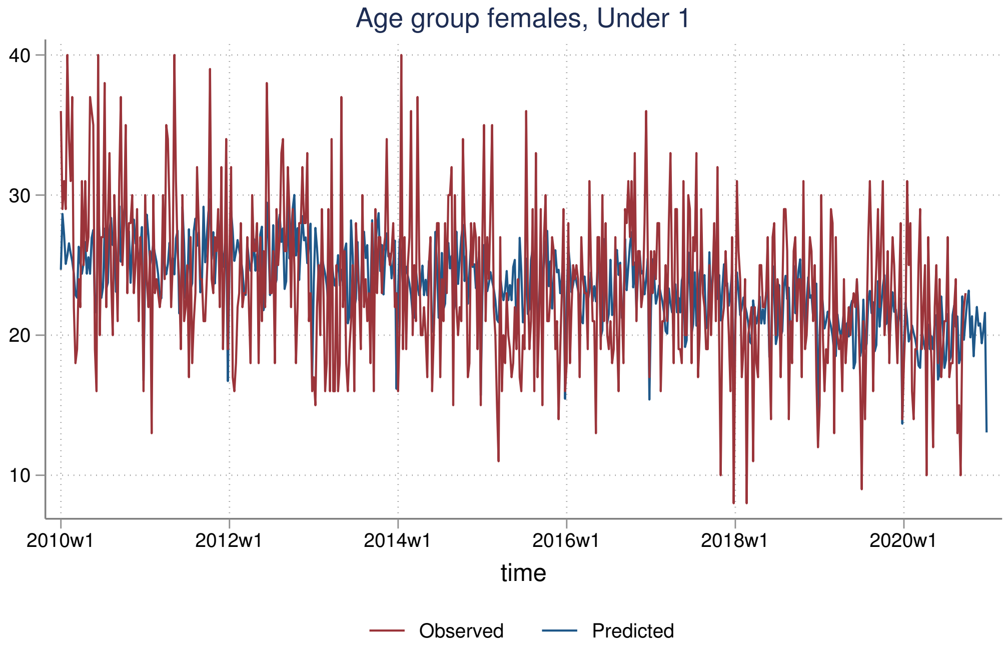

Figure 245: Females < 1 mortality time trend and model, fm 2019 wk1

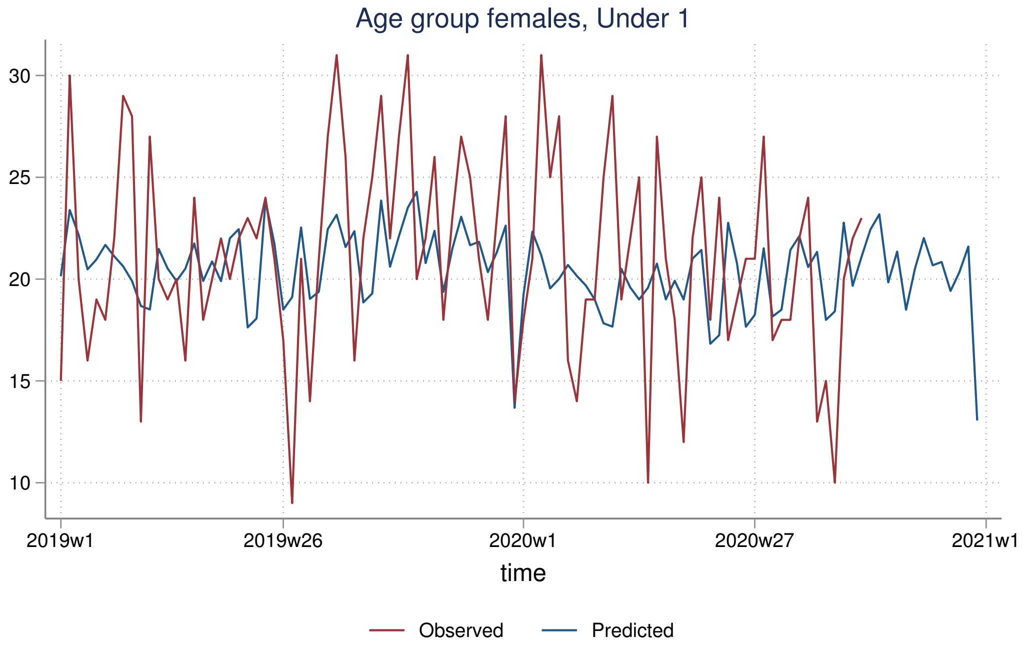

Figure 246: Females < 1 mortality time trend and model, fm 2020 wk1

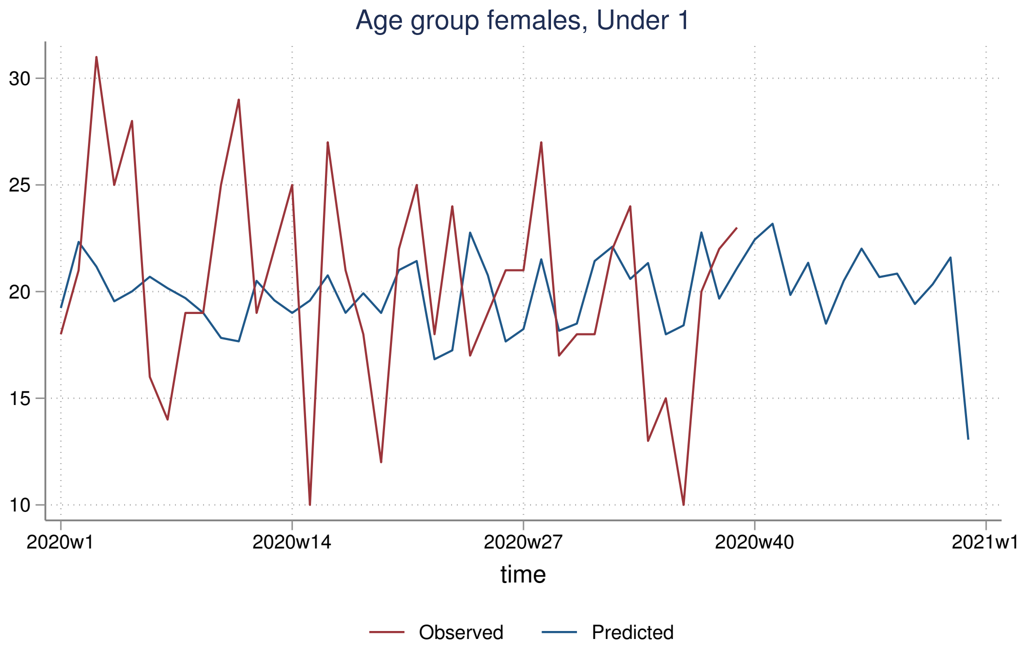

Figure 247: Females < 1 all-cause excess deaths, fm 2010 wk1

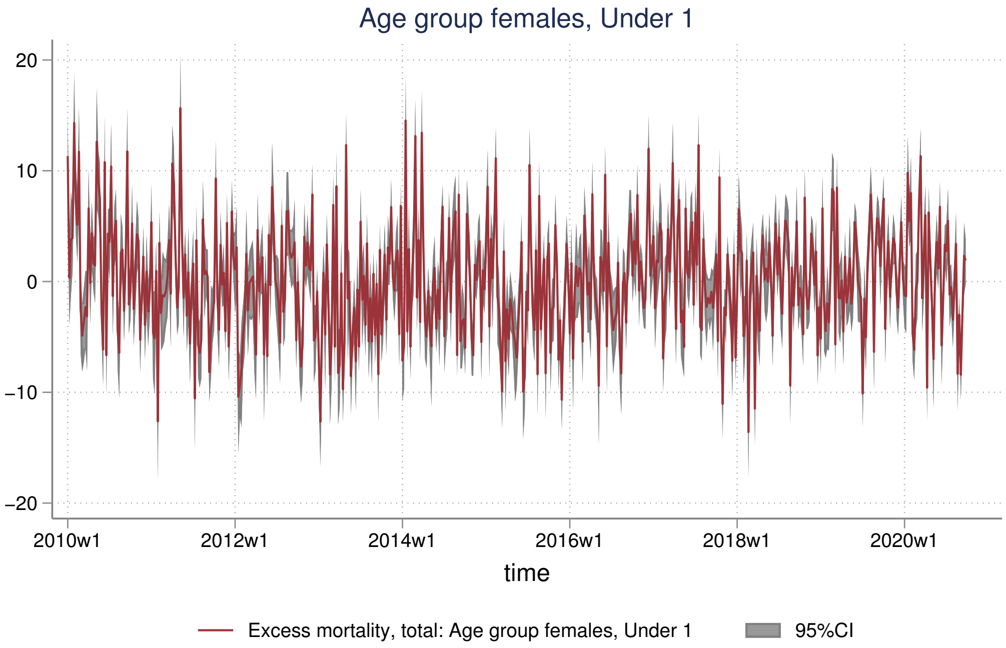

Figure 248: Females < 1 all-cause excess deaths, fm 2019 wk1

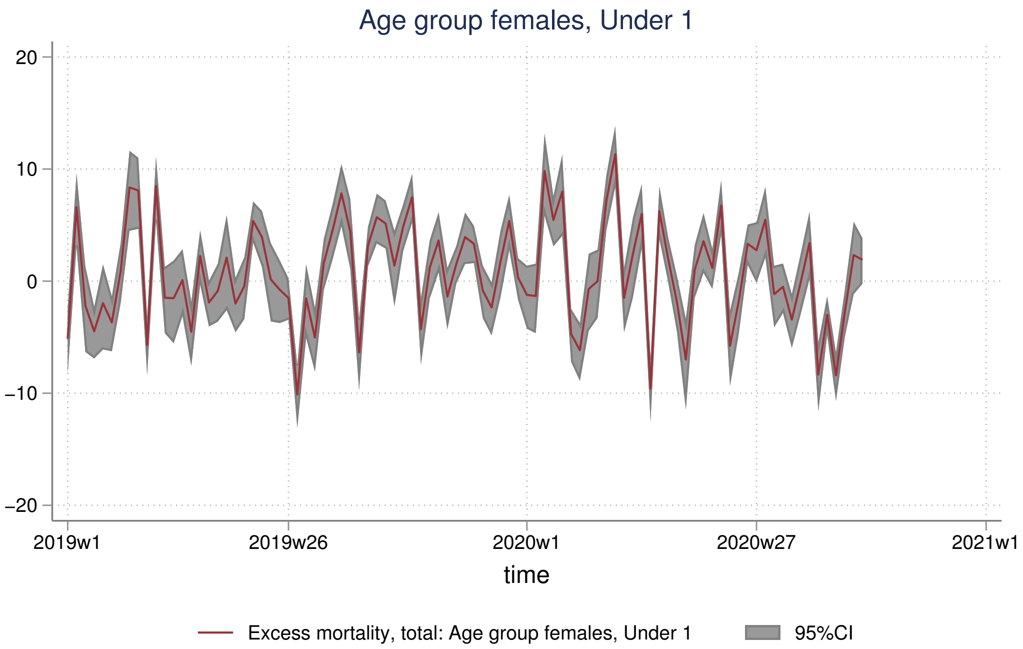

Figure 249: Females < 1 all-cause excess deaths, fm 2020 wk1

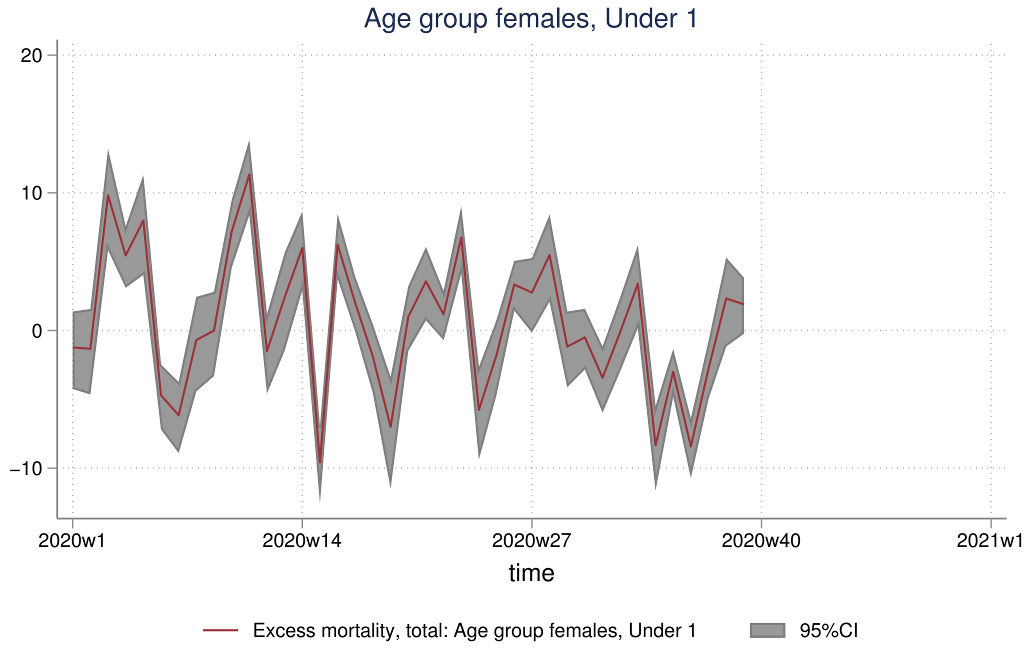

Figure 250: Females < 1 all-cause excess deaths minus COVID19, fm 2010 wk1

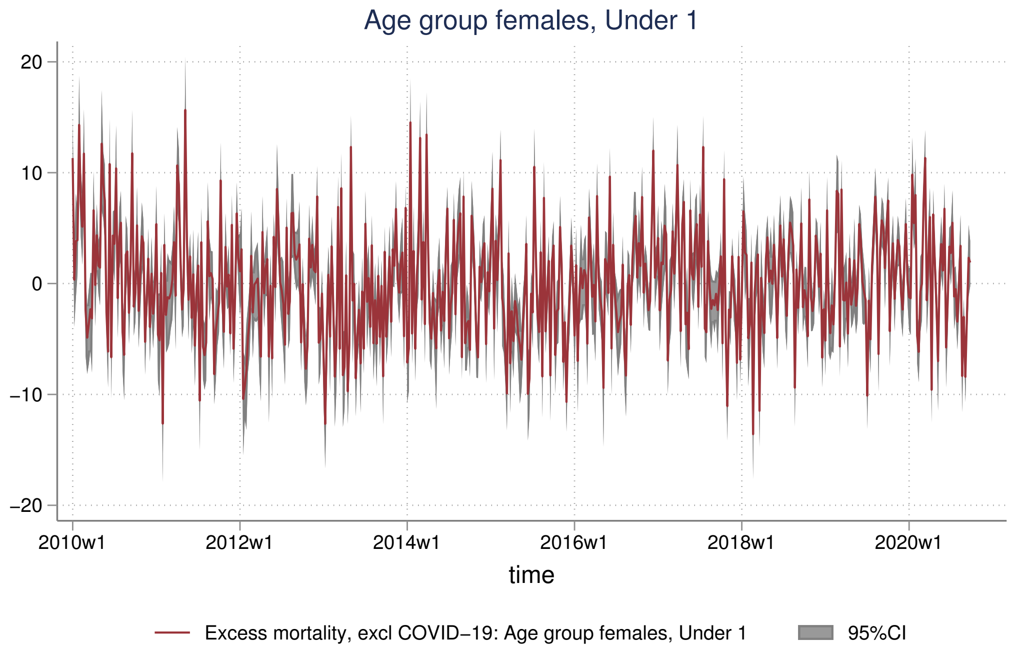

Figure 251: Females < 1 all-cause excess deaths minus COVID19, fm 2019 wk1

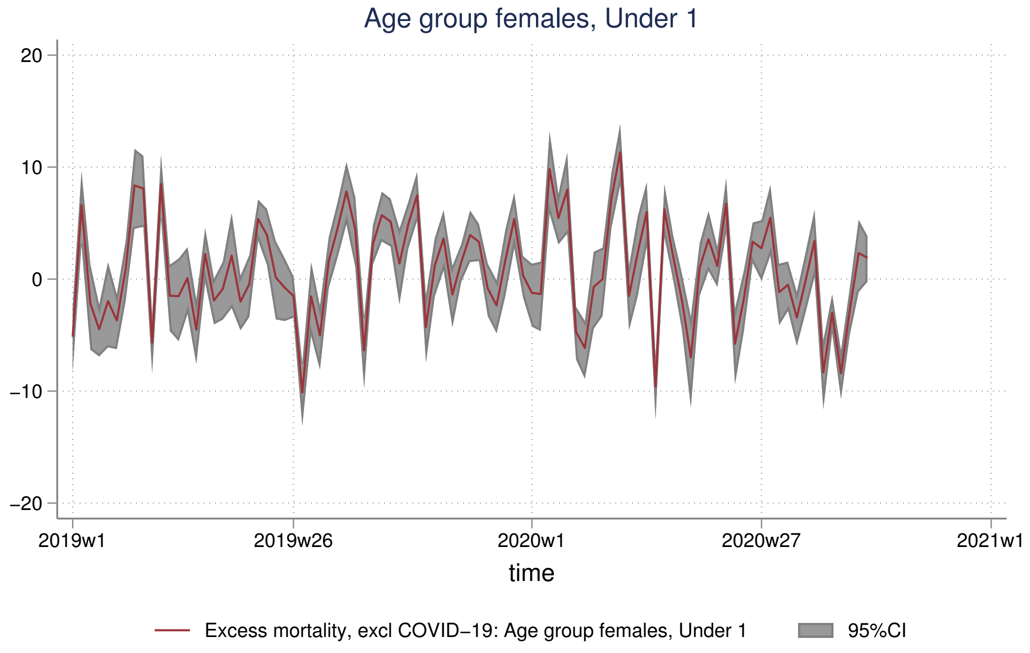

Figure 252: Females < 1 all-cause excess deaths minus COVID19, fm 2020 wk1

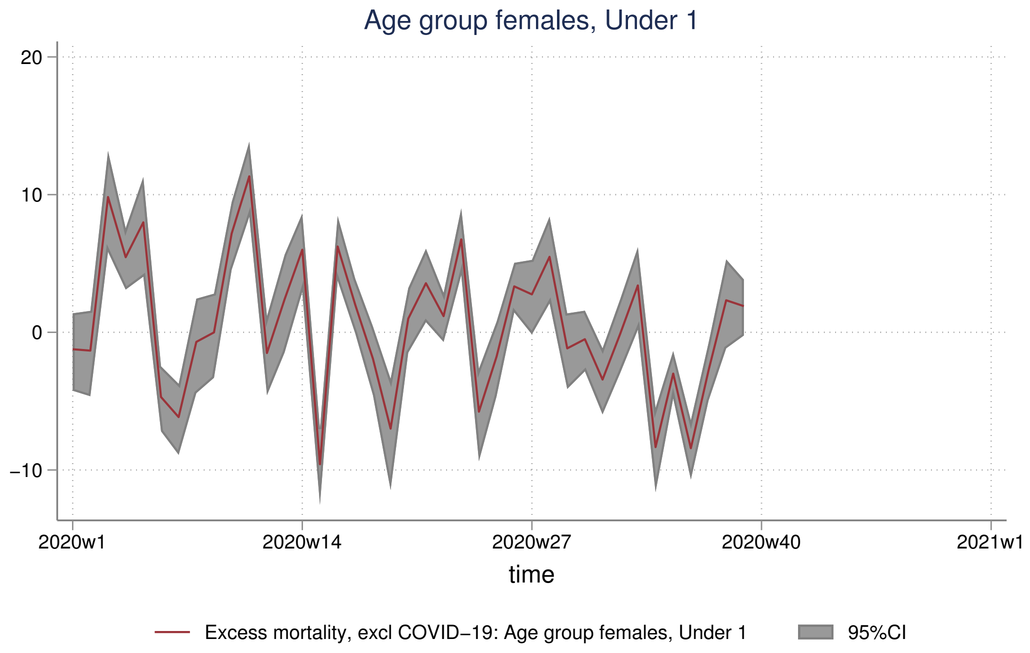

5.3 Females aged 01-14

Figure 253: Females 01-14 mortality time trend and model, fm 2010 wk1

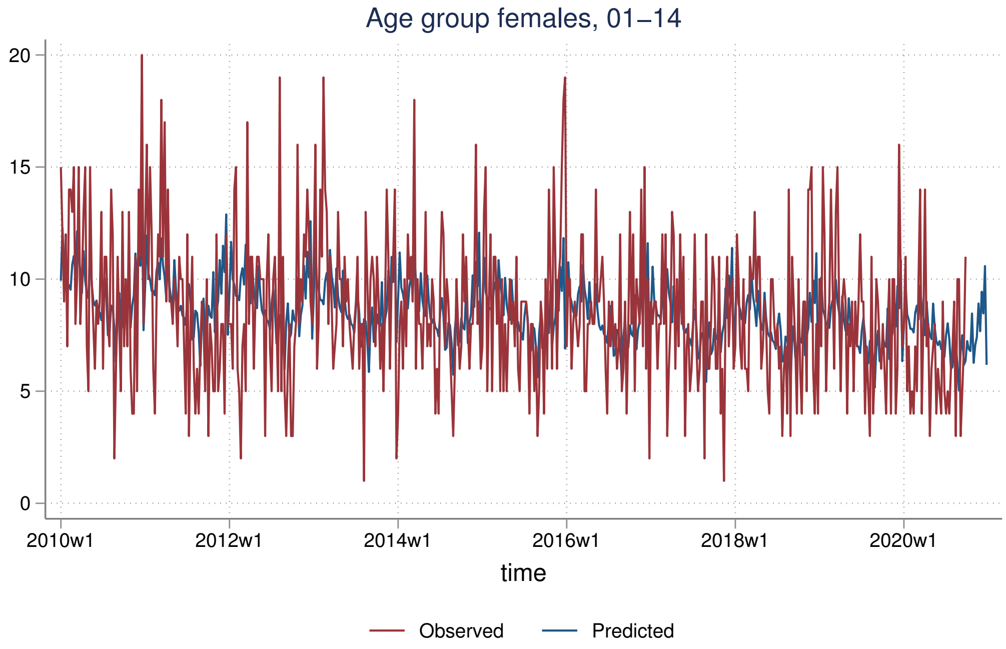

Figure 254: Females 01-14 mortality time trend and model, fm 2019 wk1

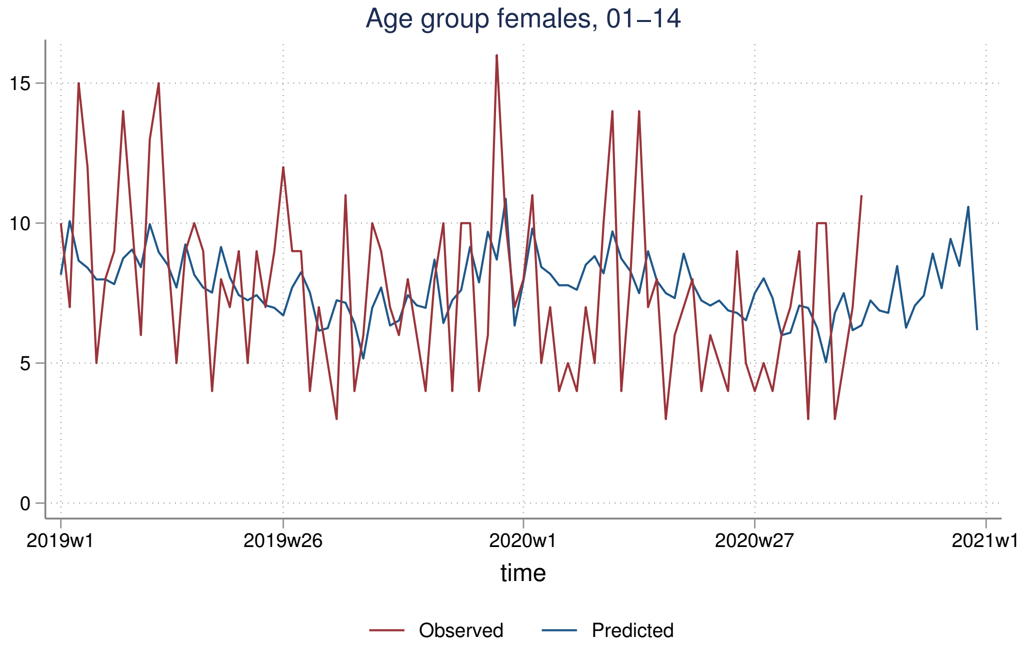

Figure 255: Females 01-14 mortality time trend and model, fm 2020 wk1

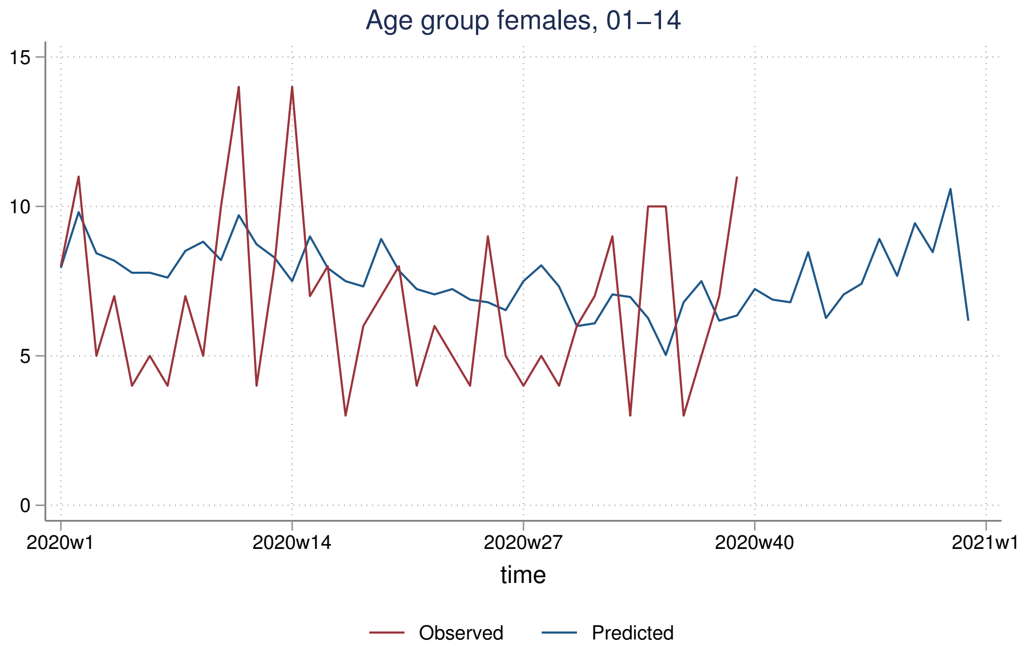

Figure 256: Females 01-14 all-cause excess deaths, fm 2010 wk1

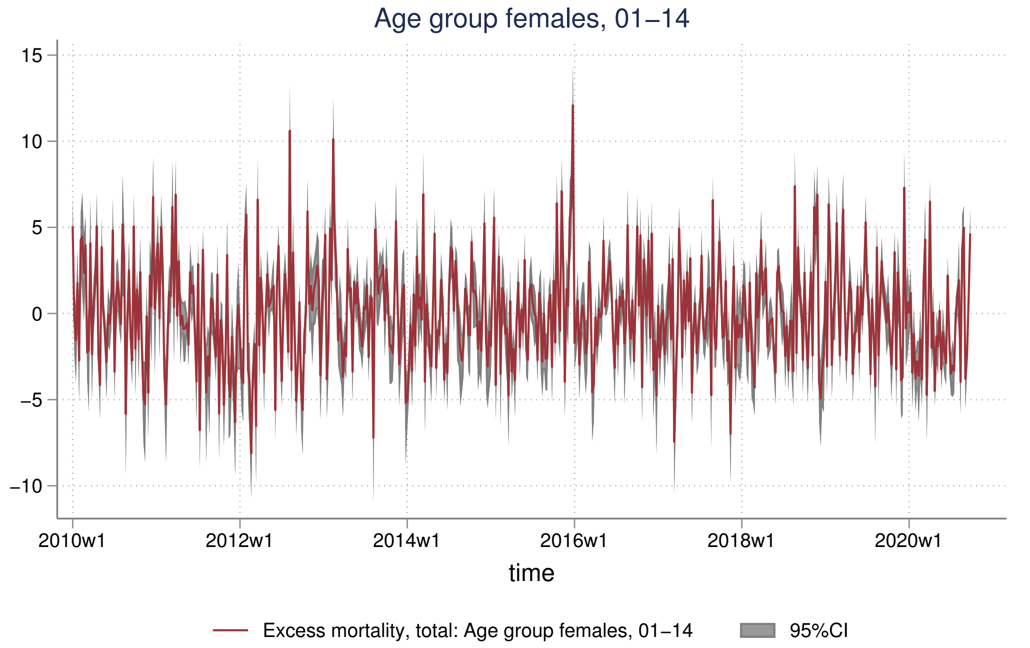

Figure 257: Females 01-14 all-cause excess deaths, fm 2019 wk1

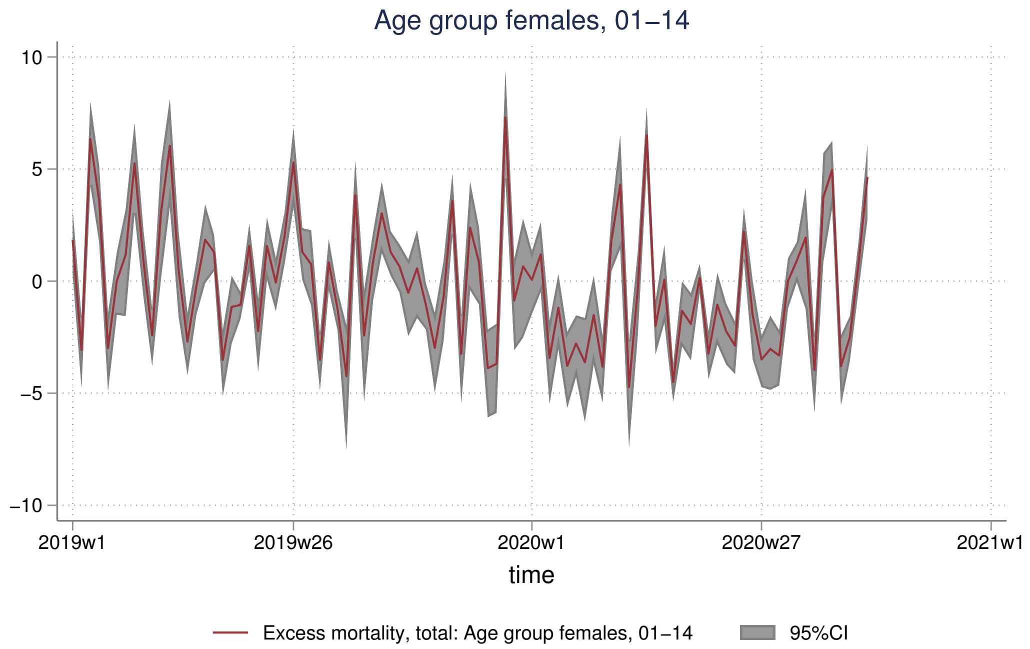

Figure 258: Females 01-14 all-cause excess deaths, fm 2020 wk1

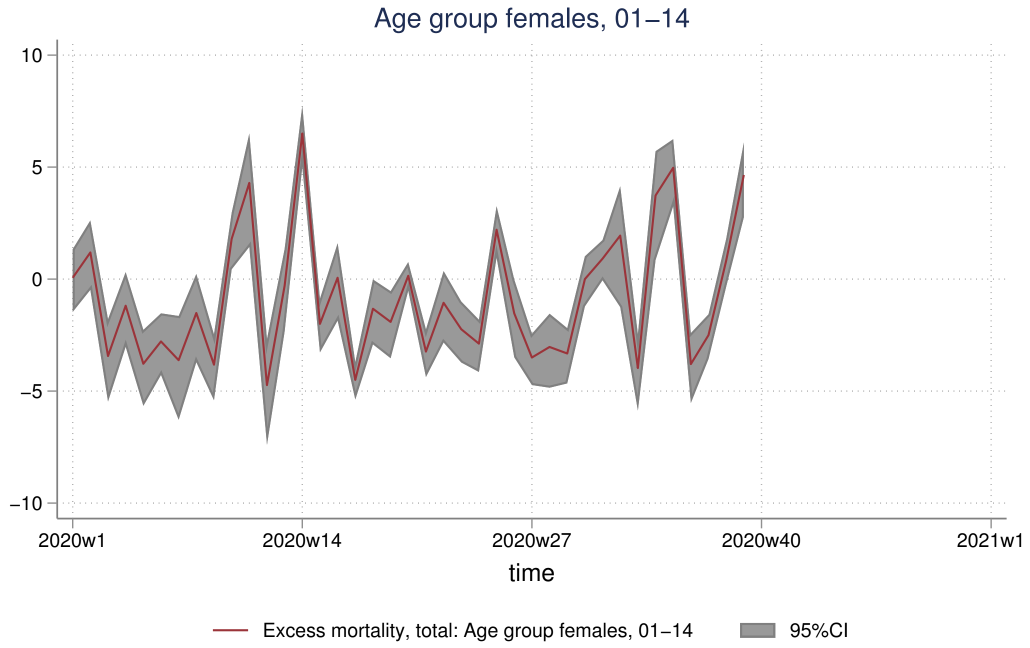

Figure 259: Females 01-14 all-cause excess deaths (−COVID19), fm 2010 wk1

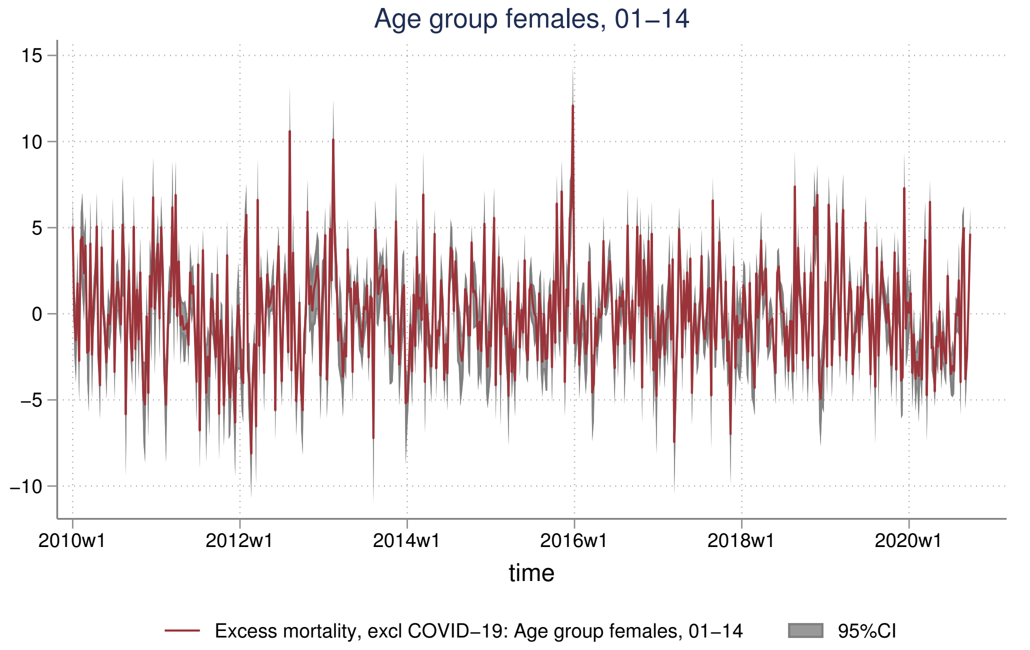

Figure 260: Females 01-14 all-cause excess deaths (−COVID19), fm 2019 wk1

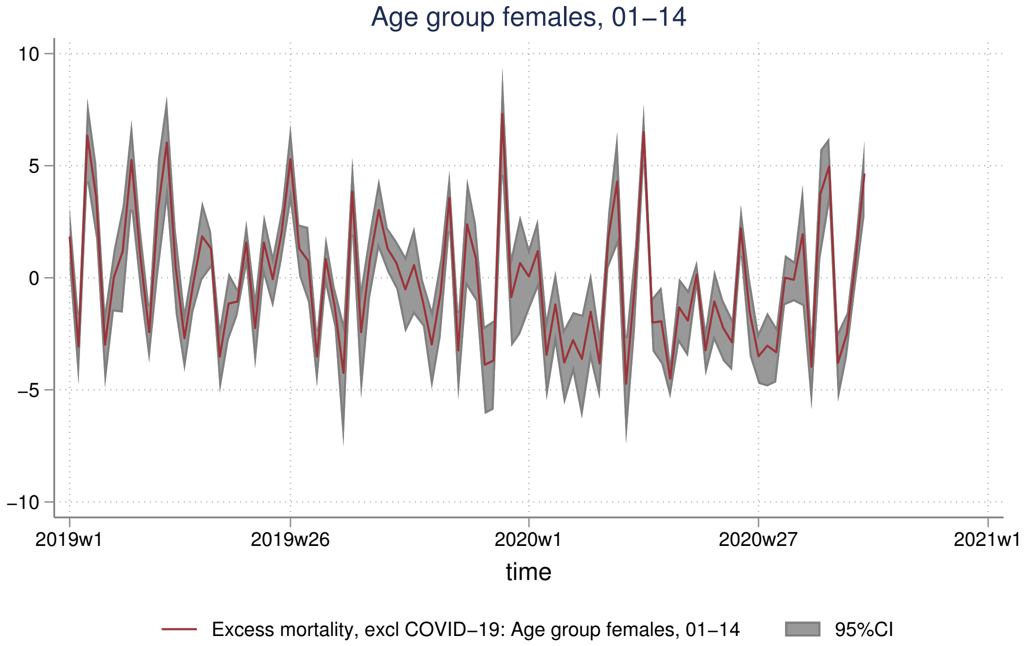

Figure 261: Females 01-14 all-cause excess deaths (−COVID19), fm 2020 wk1

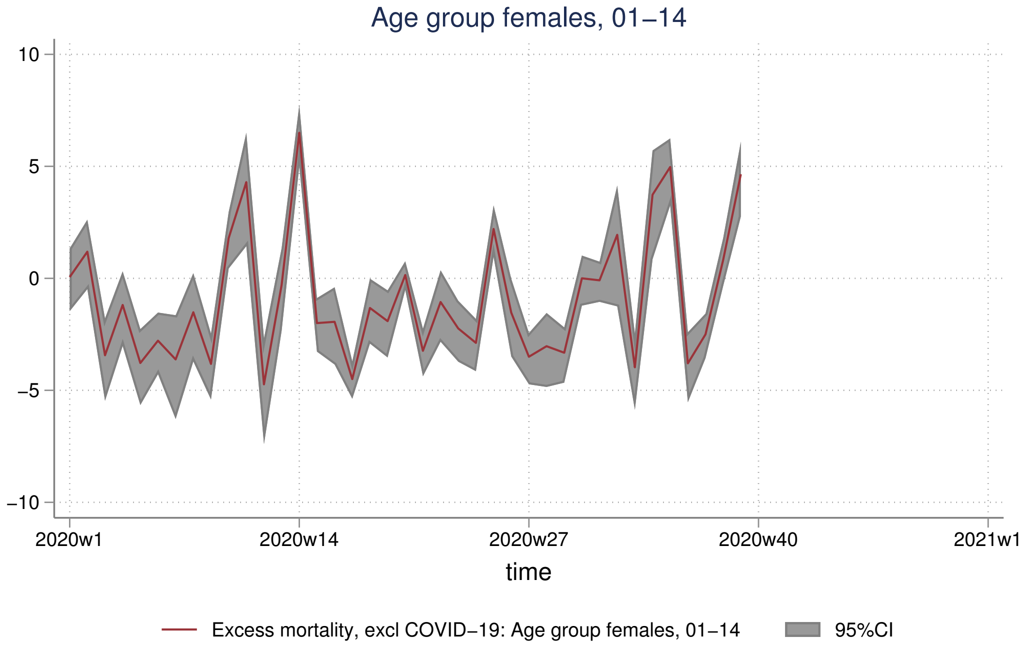

5.4 Females aged 15-44

Figure 262: Females 15-44 mortality time trend and model, fm 2010 wk1

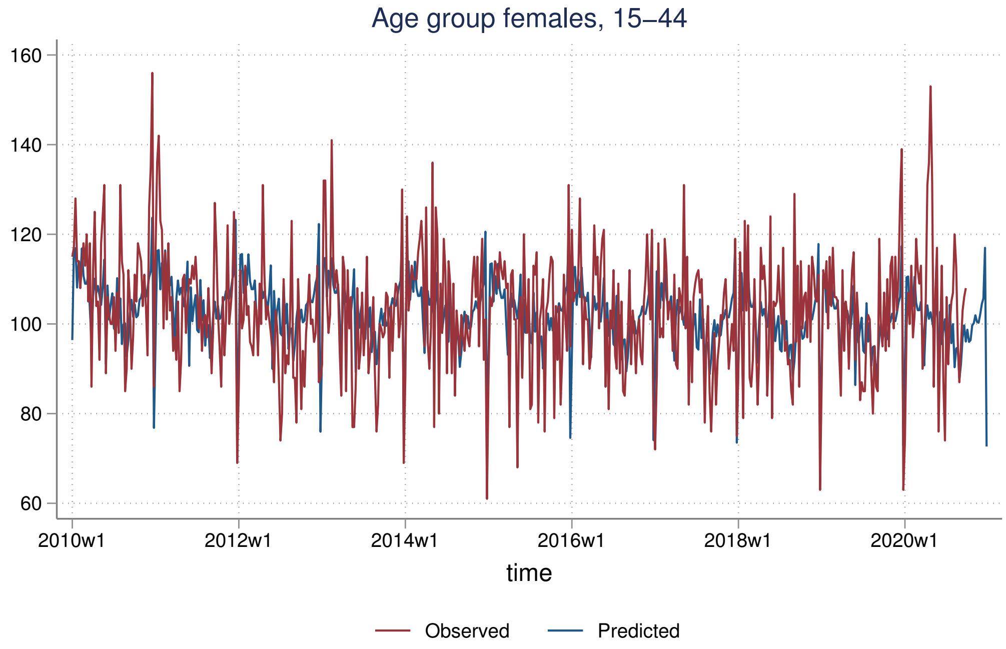

Figure 263: Females 15-44 mortality time trend and model, fm 2019 wk1

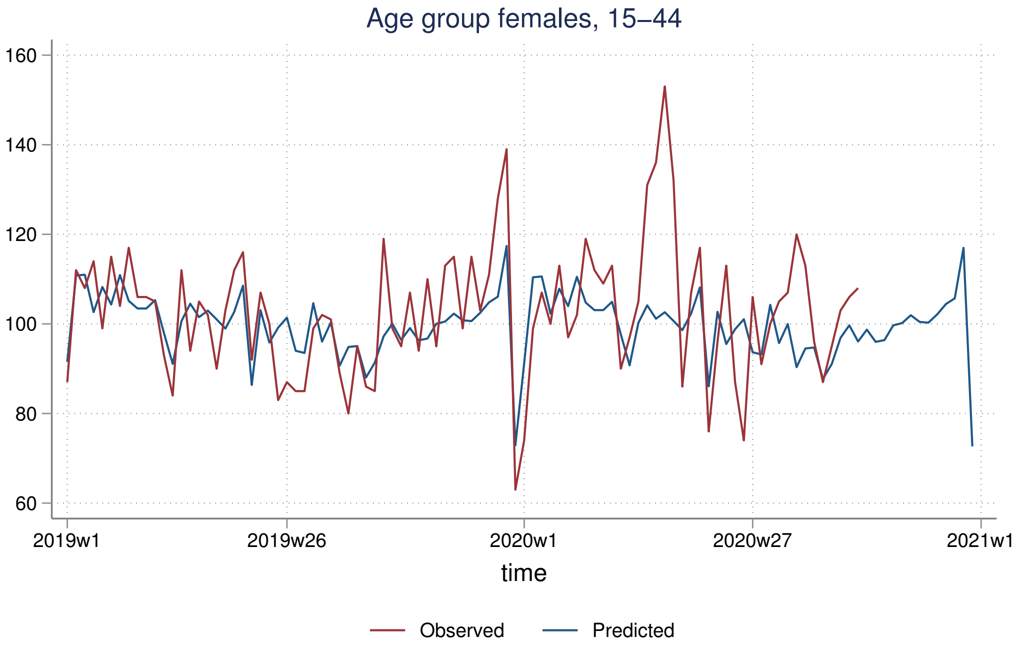

Figure 264: Females 15-44 mortality time trend and model, fm 2020 wk1

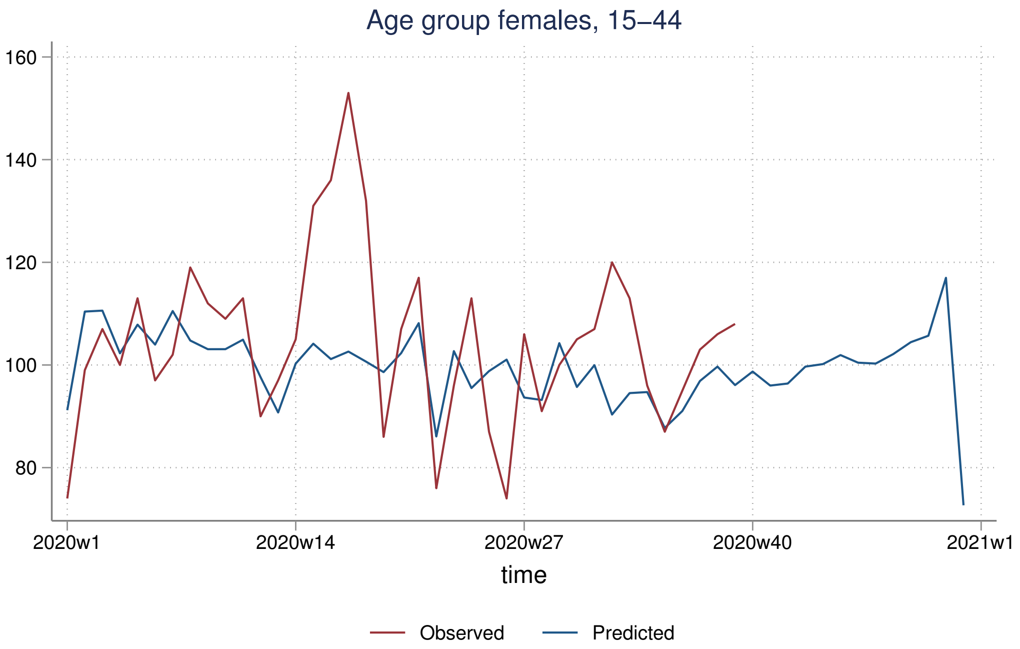

Figure 265: Females 15-44 all-cause excess deaths, fm 2010 wk1

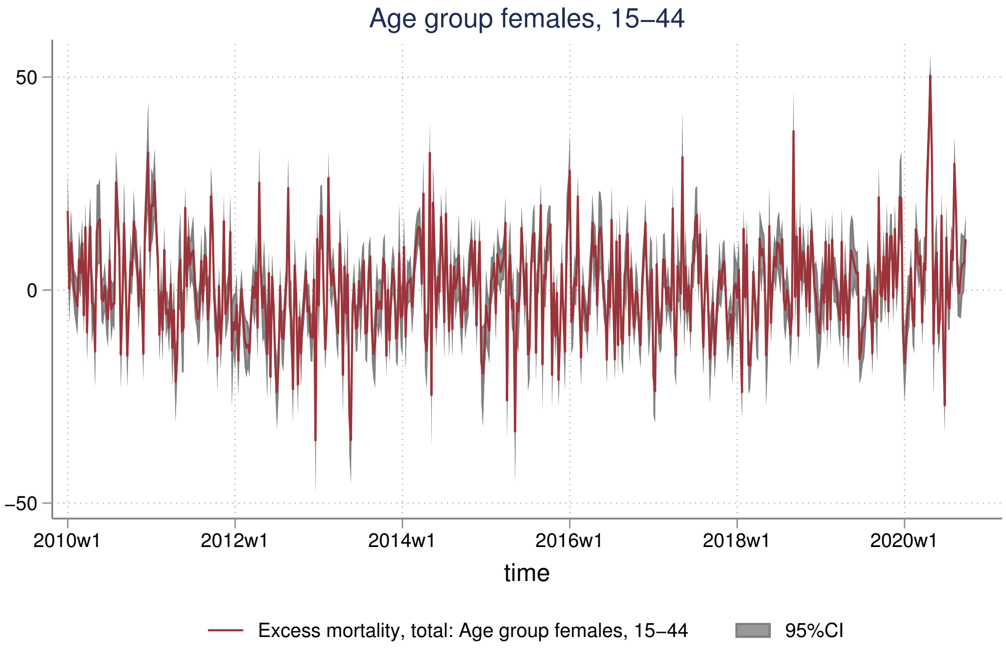

Figure 266: Females 15-44 all-cause excess deaths, fm 2019 wk1

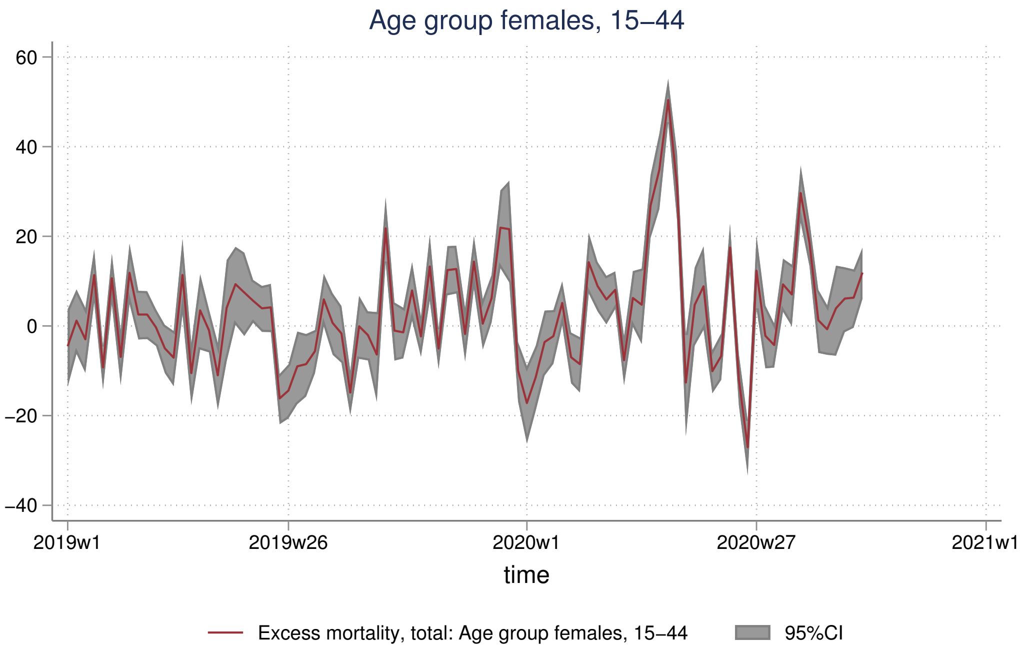

Figure 267: Females 15-44 all-cause excess deaths, fm 2020 wk1

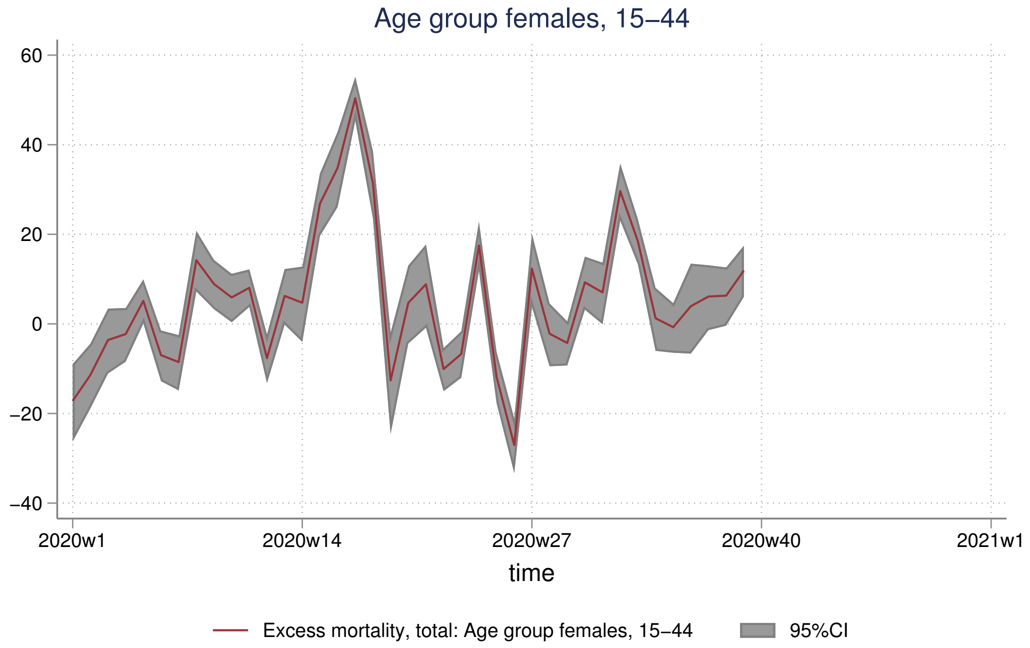

Figure 268: Females 15-44 all-cause excess deaths (−COVID19), fm 2010 wk1

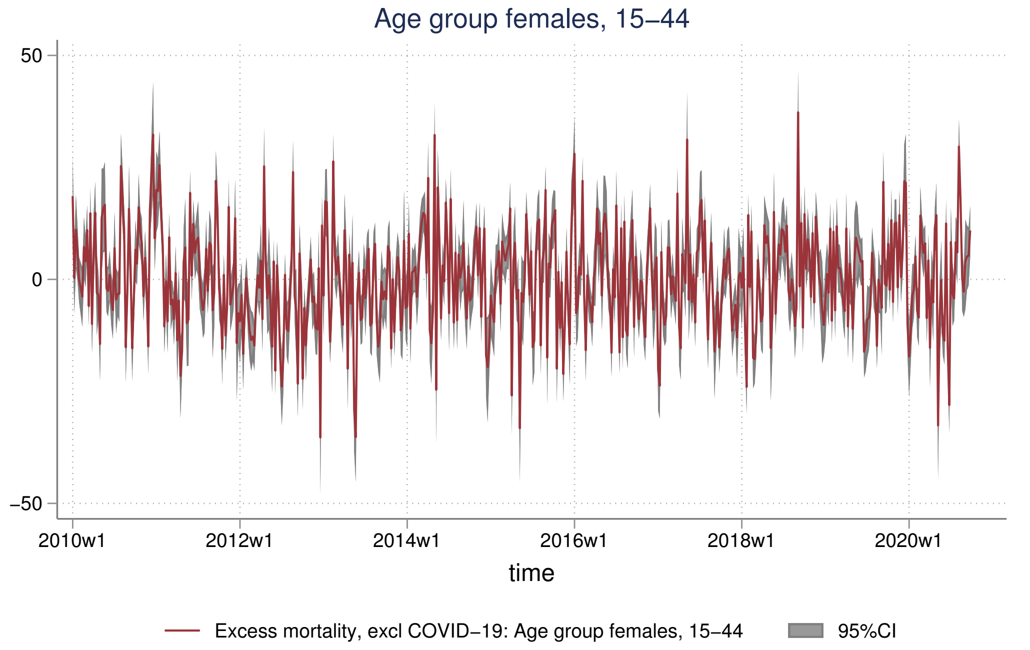

Figure 269: Females 15-44 all-cause excess deaths (−COVID19), fm 2019 wk1

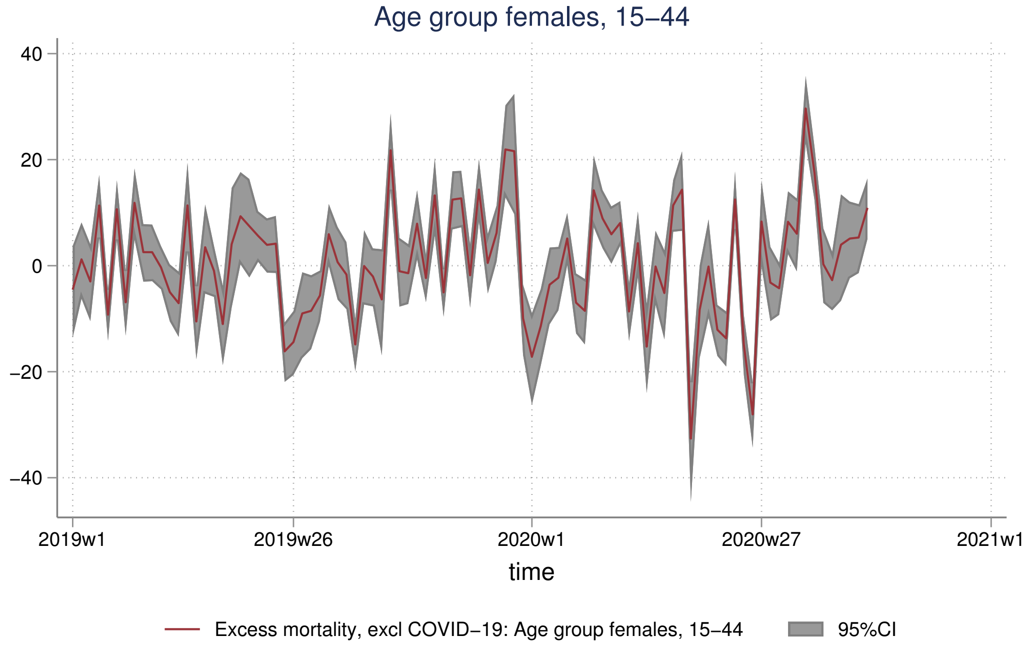

Figure 270: Females 15-44 all-cause excess deaths (−COVID19), fm 2020 wk1

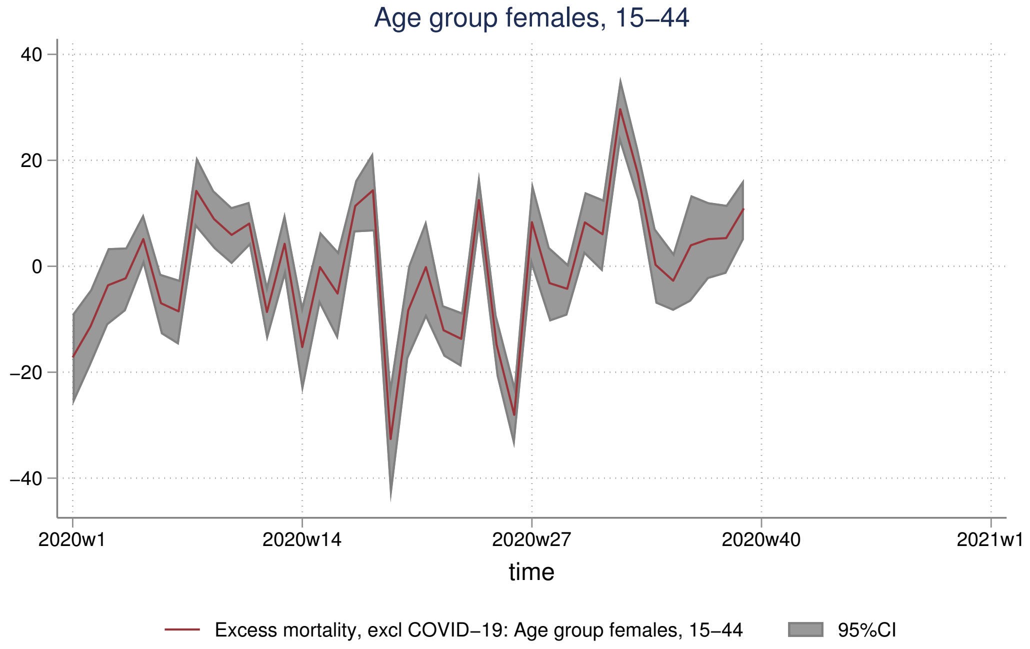

5.5 Females aged 45-64

Figure 271: Females 45-64 mortality time trend and model, fm 2010 wk1

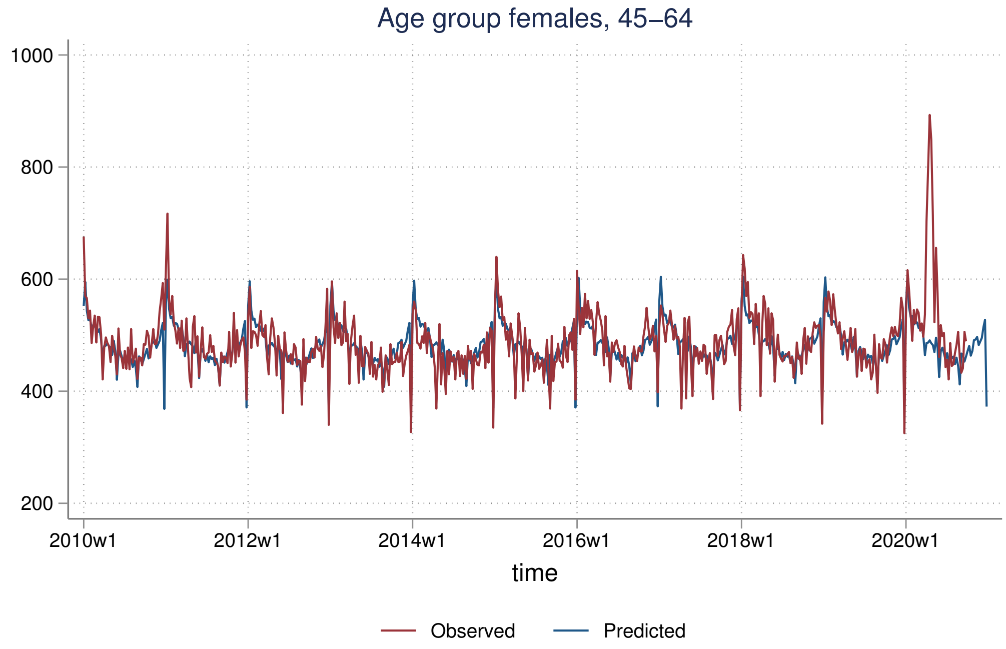

Figure 272: Females 45-64 mortality time trend and model, fm 2019 wk1

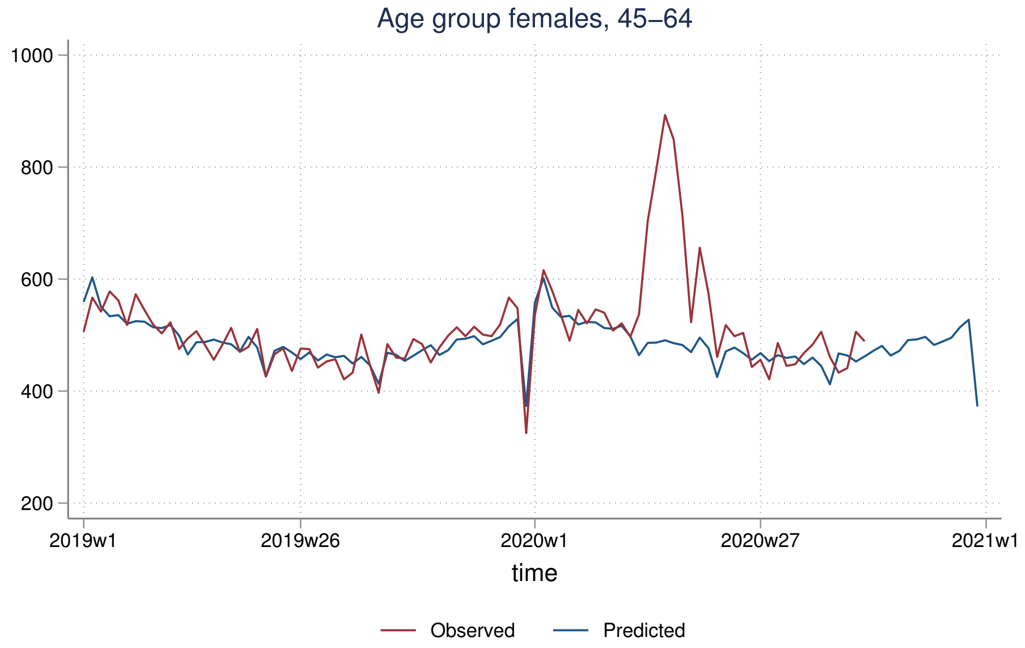

Figure 273: Females 45-64 mortality time trend and model, fm 2020 wk1

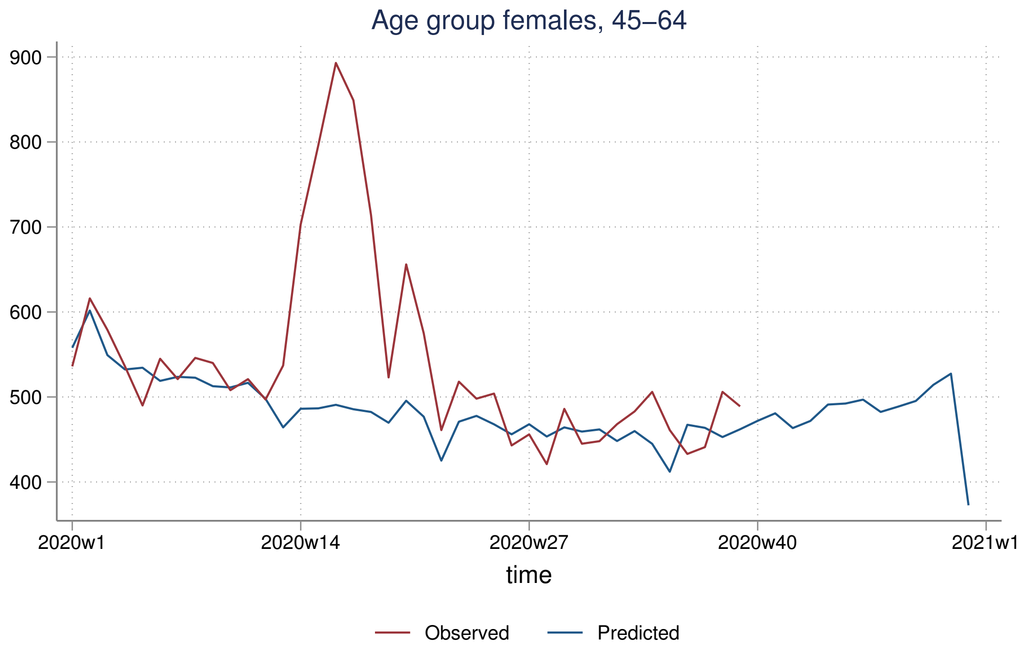

Figure 274: Females 45-64 all-cause excess deaths, fm 2010 wk1

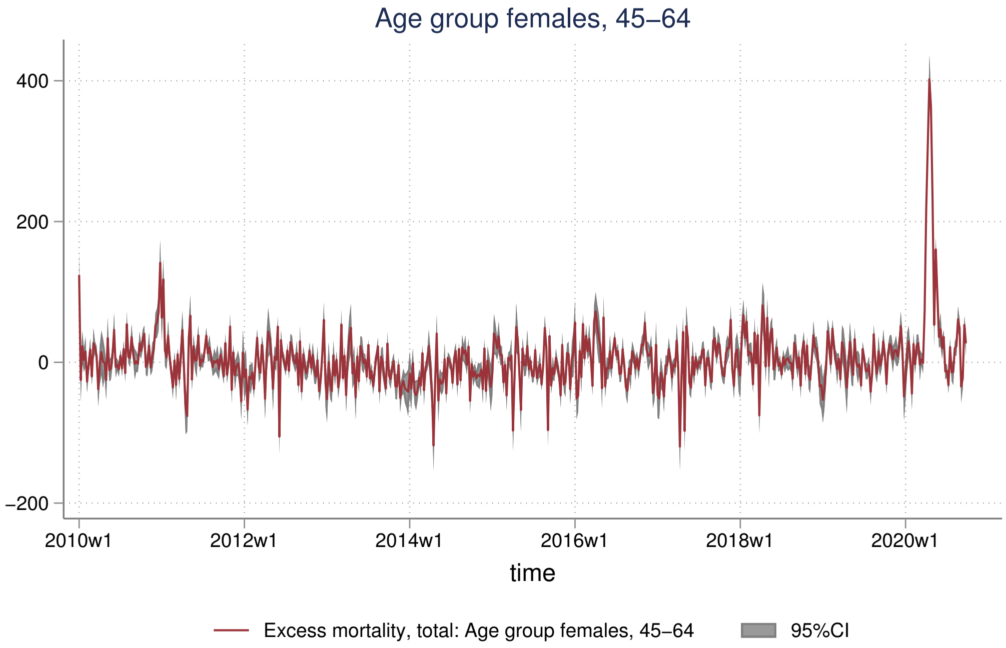

Figure 275: Females 45-64 all-cause excess deaths, fm 2019 wk1

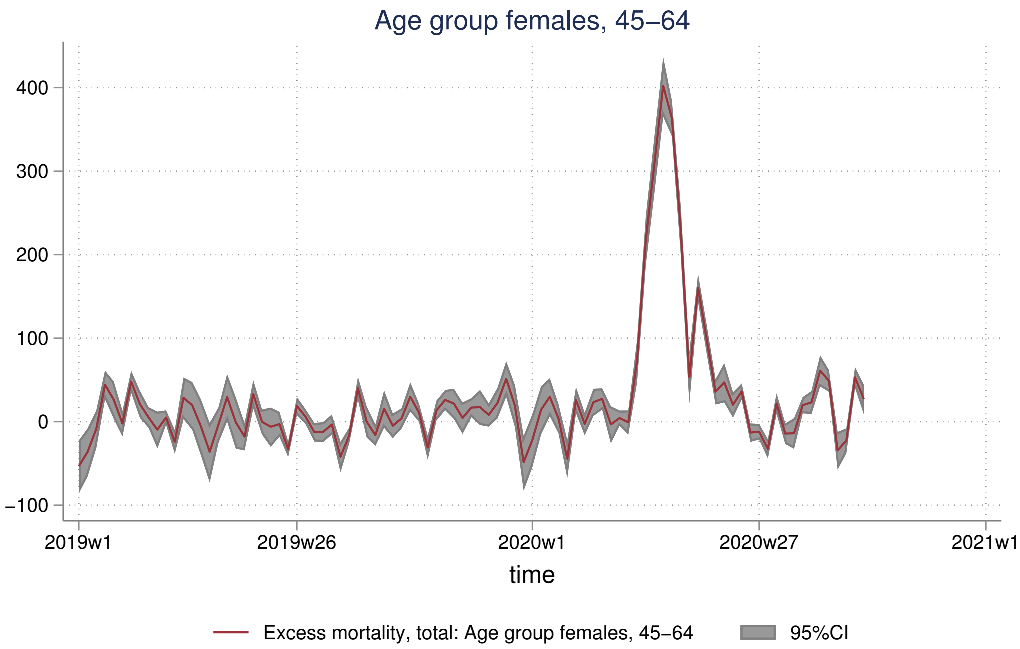

Figure 276: Females 45-64 all-cause excess deaths, fm 2020 wk1

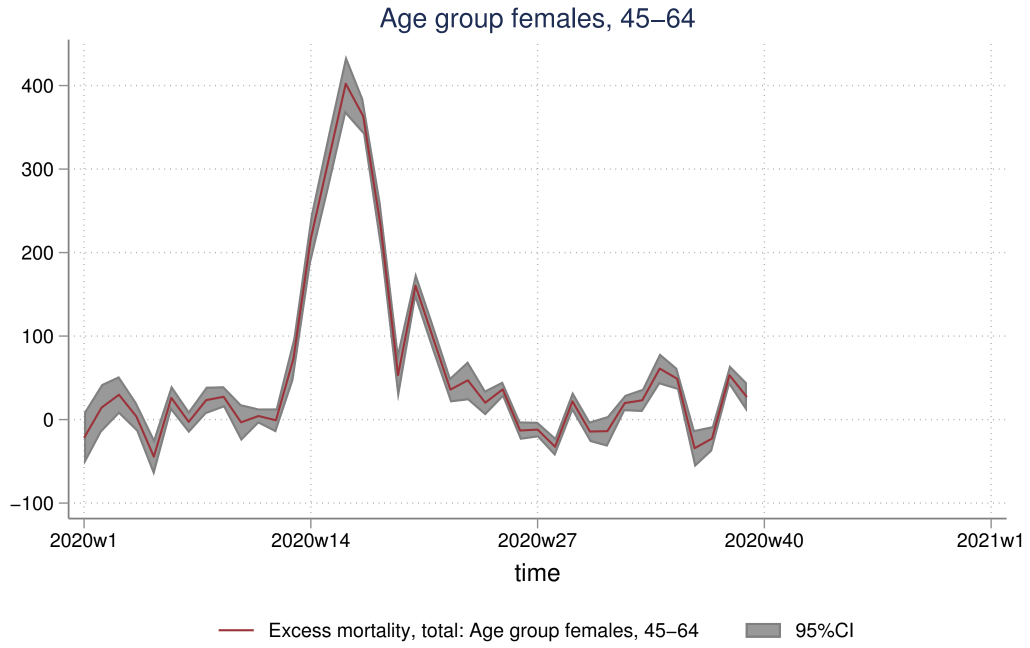

Figure 277: Females 45-64 all-cause excess deaths (−COVID19), fm 2010 wk1

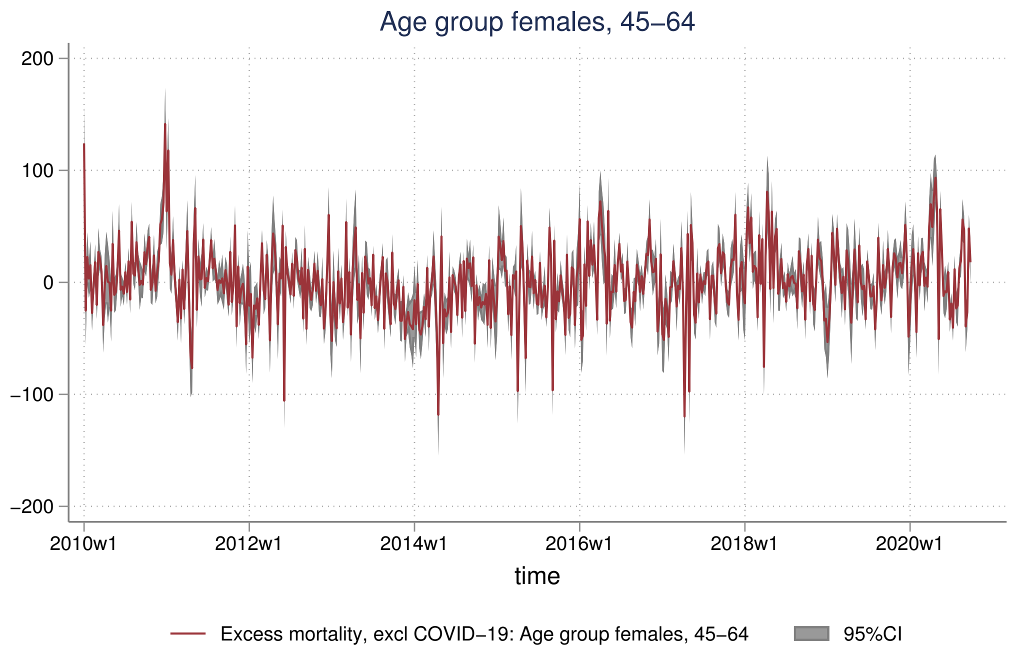

Figure 278: Females 45-64 all-cause excess deaths (−COVID19), fm 2019 wk1

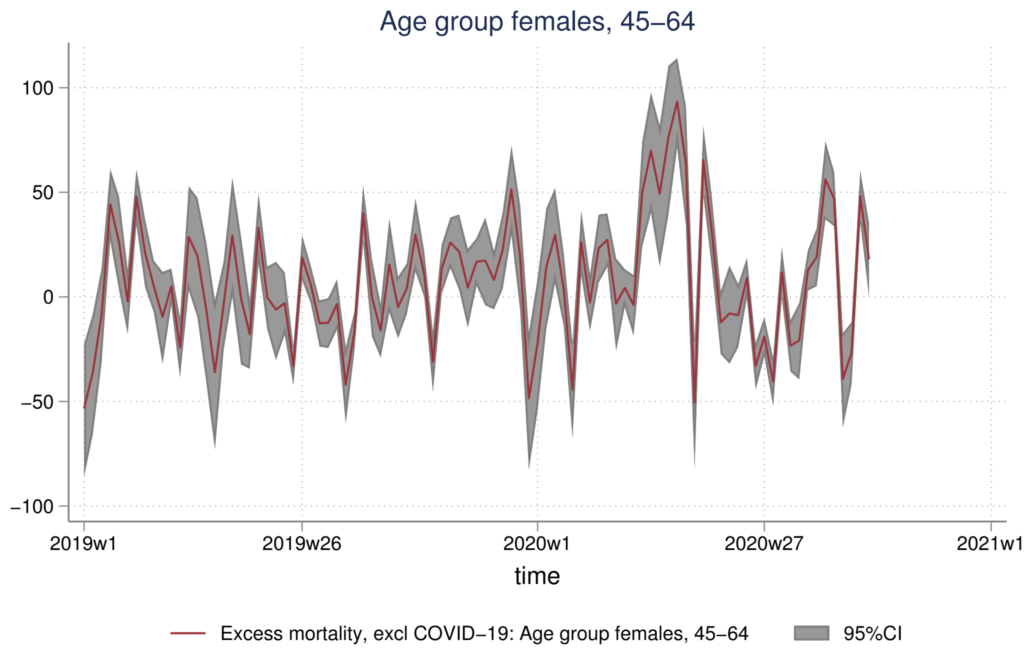

Figure 279: Females 45-64 all-cause excess deaths (−COVID19), fm 2020 wk1

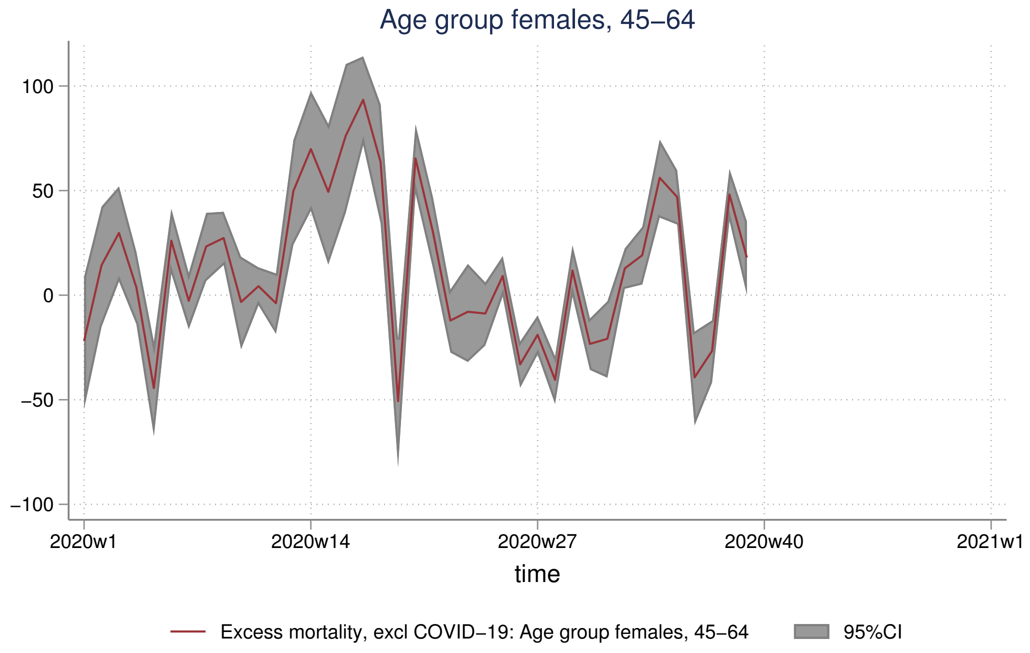

5.6 Females aged 65-74

Figure 280: Females 65-74 mortality time trend and model, fm 2010 wk1

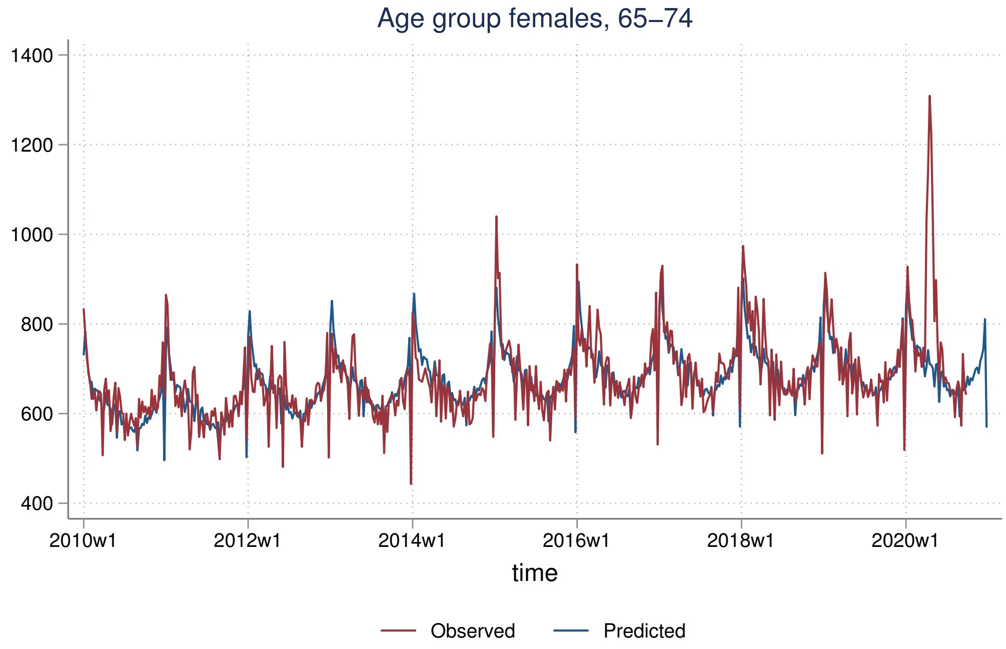

Figure 281: Females 65-74 mortality time trend and model, fm 2019 wk1

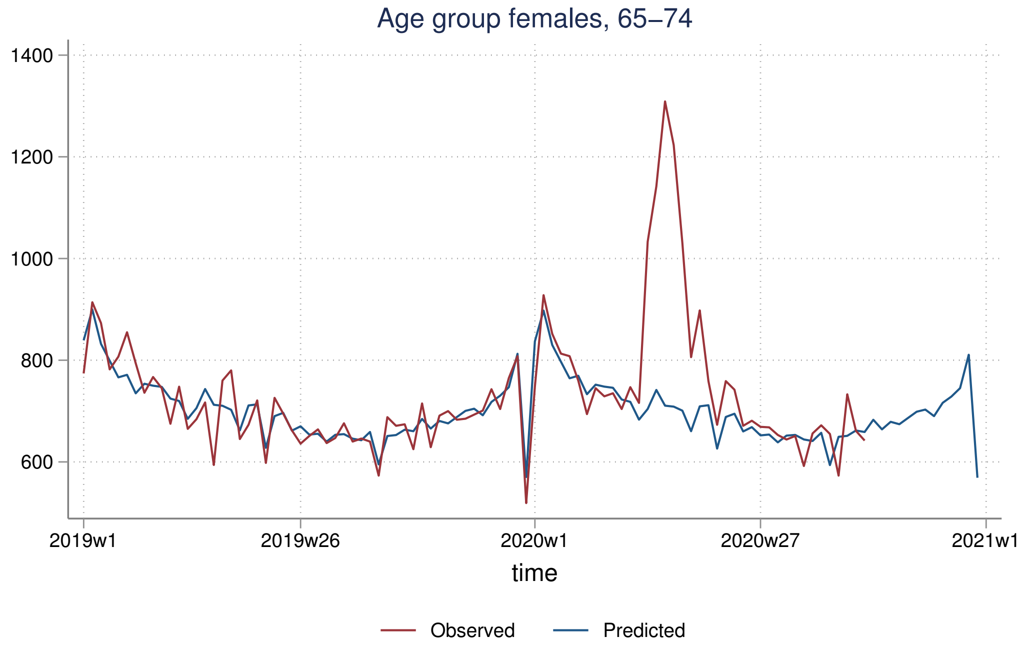

Figure 282: Females 65-74 mortality time trend and model, fm 2020 wk1

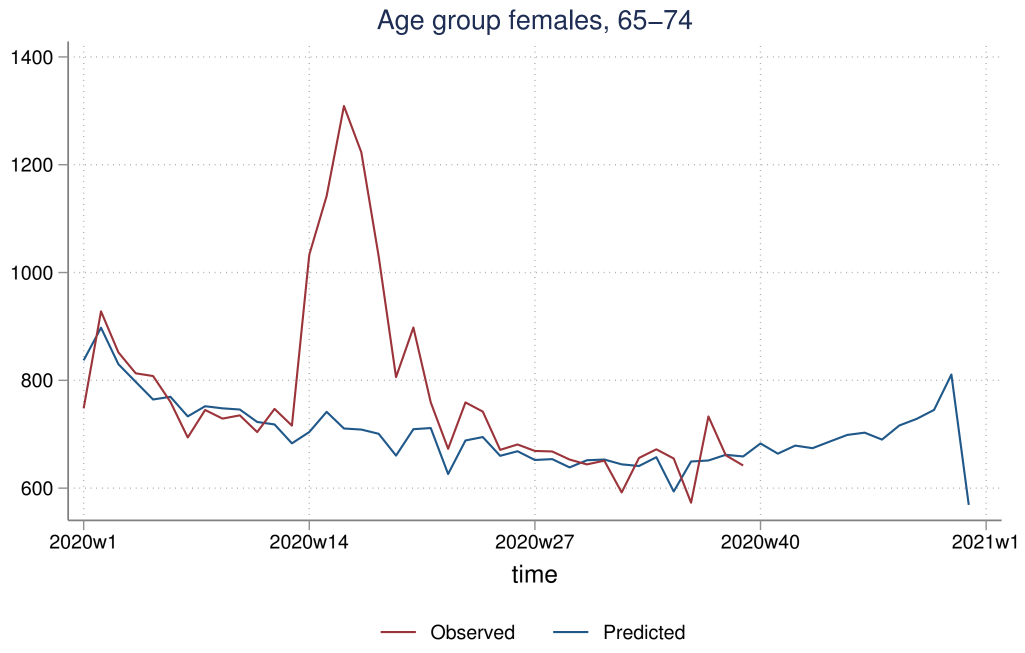

Figure 283: Females 65-74 all-cause excess deaths, fm 2010 wk1

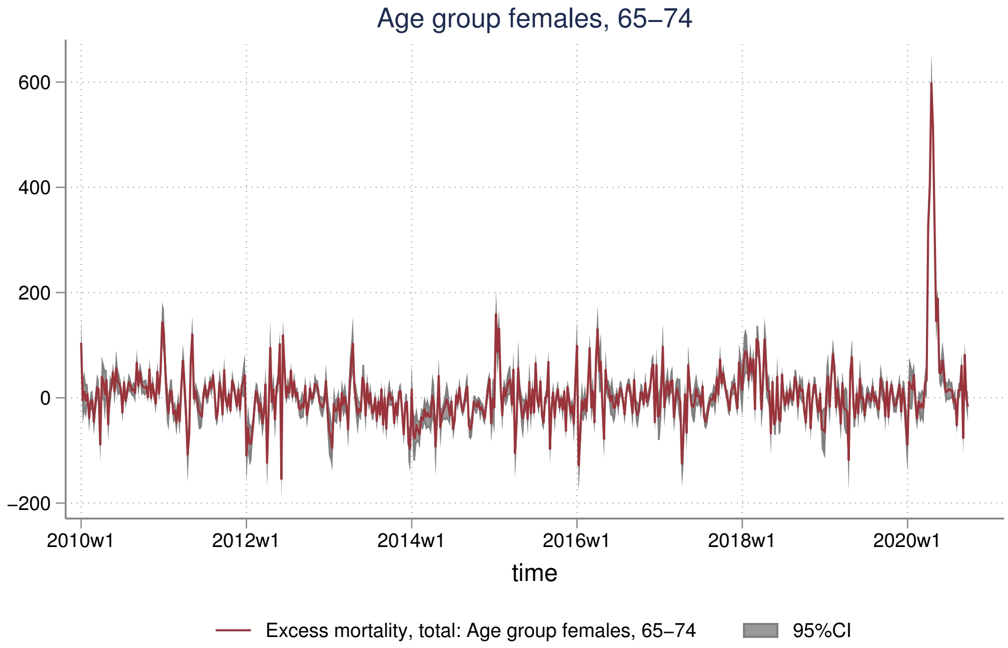

Figure 284: Females 65-74 all-cause excess deaths, fm 2019 wk1

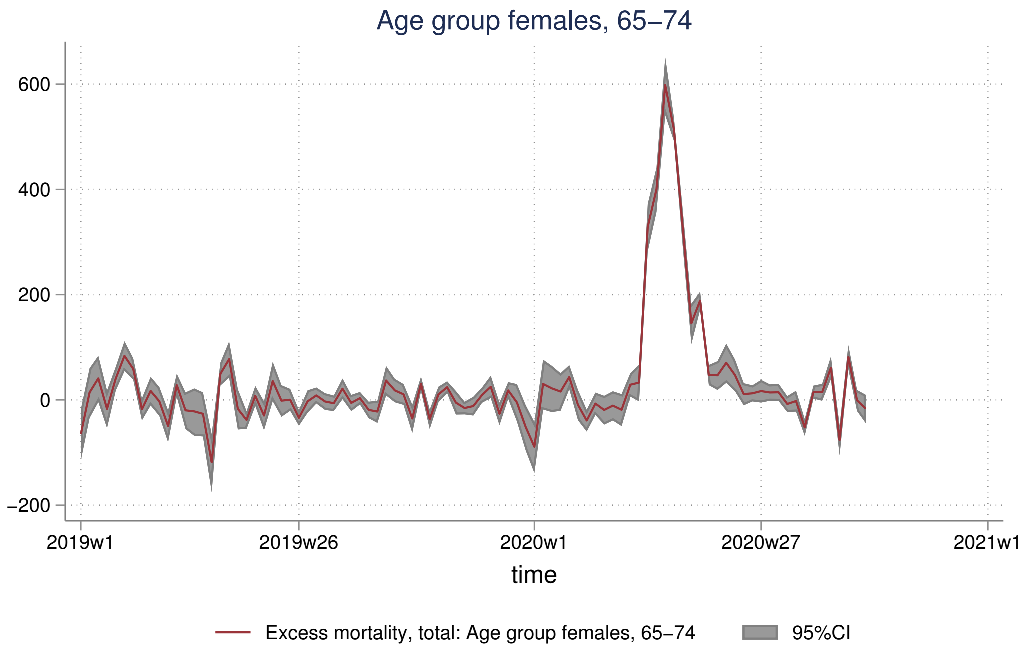

Figure 285: Females 65-74 all-cause excess deaths, fm 2020 wk1

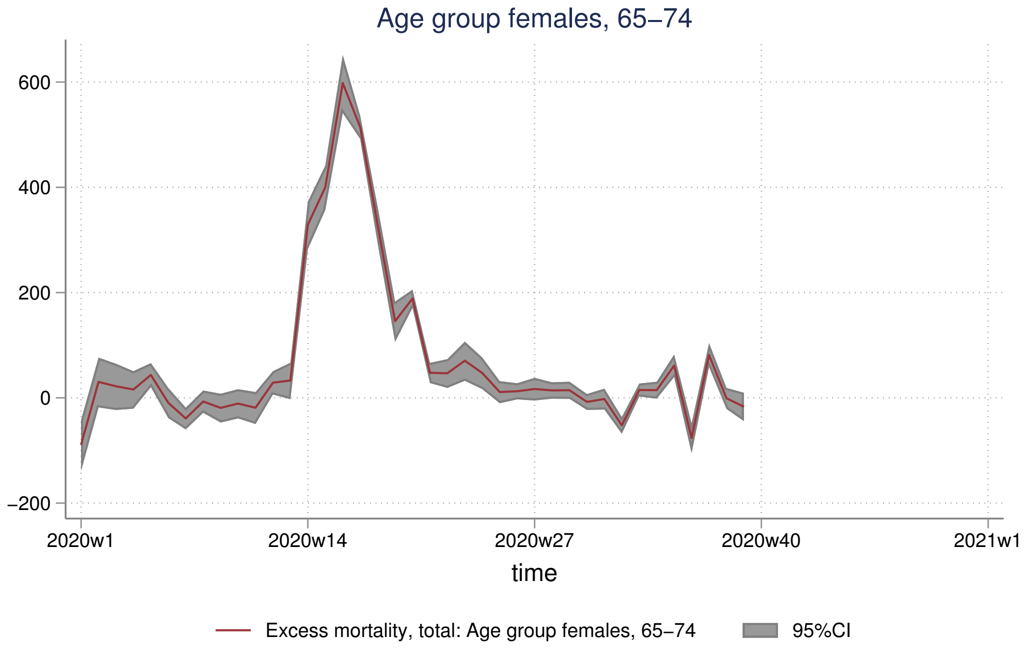

Figure 286: Females 65-74 all-cause excess deaths (−COVID19), fm 2010 wk1

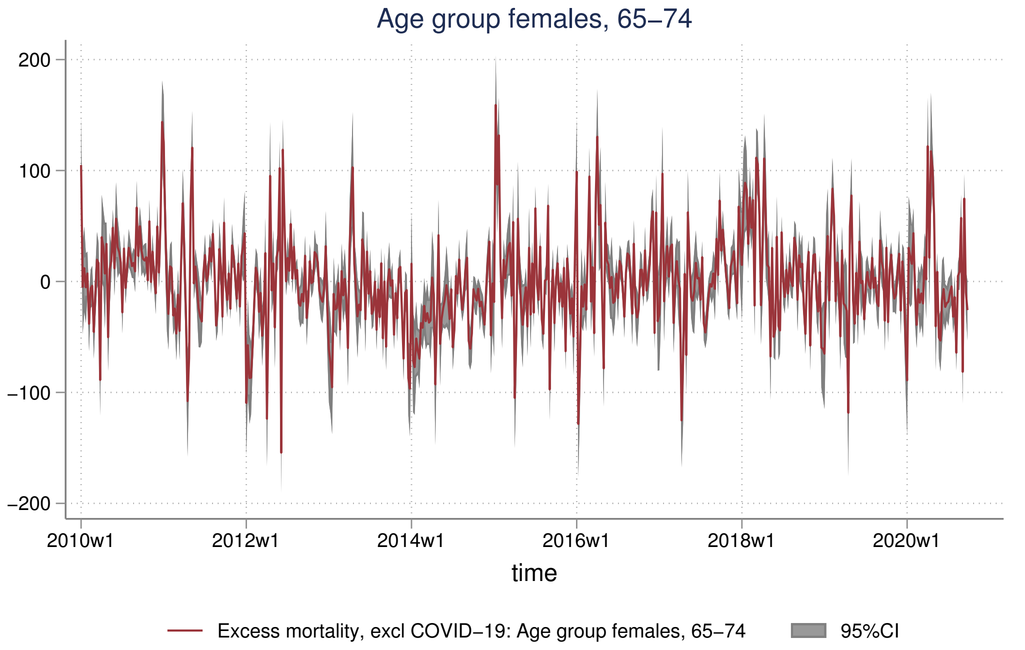

Figure 287: Females 65-74 all-cause excess deaths (−COVID19), fm 2019 wk1

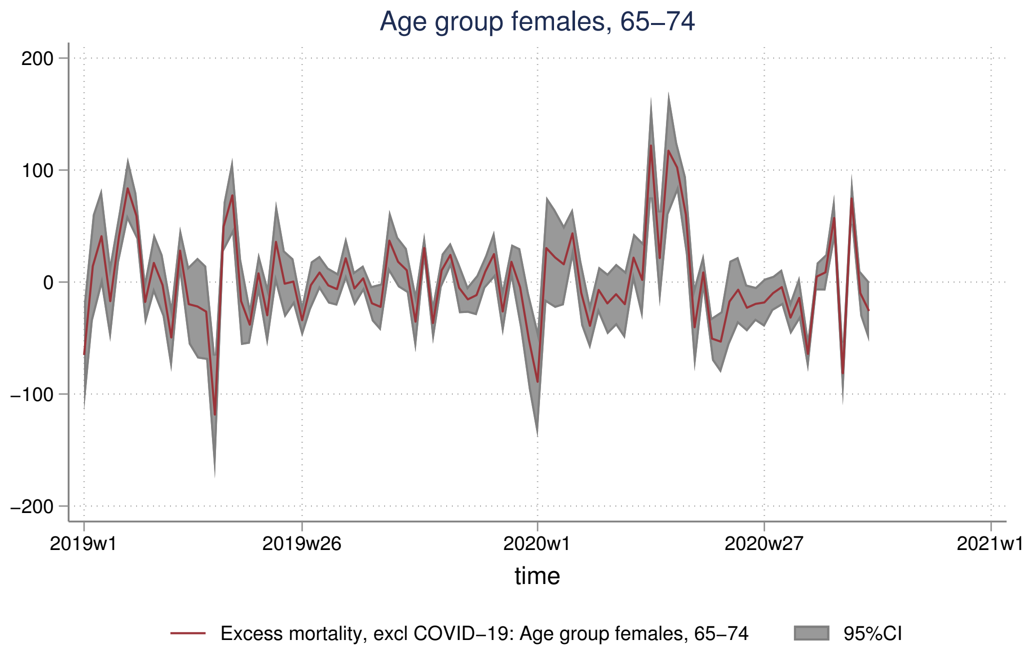

Figure 288: Females 65-74 all-cause excess deaths (−COVID19), fm 2020 wk1

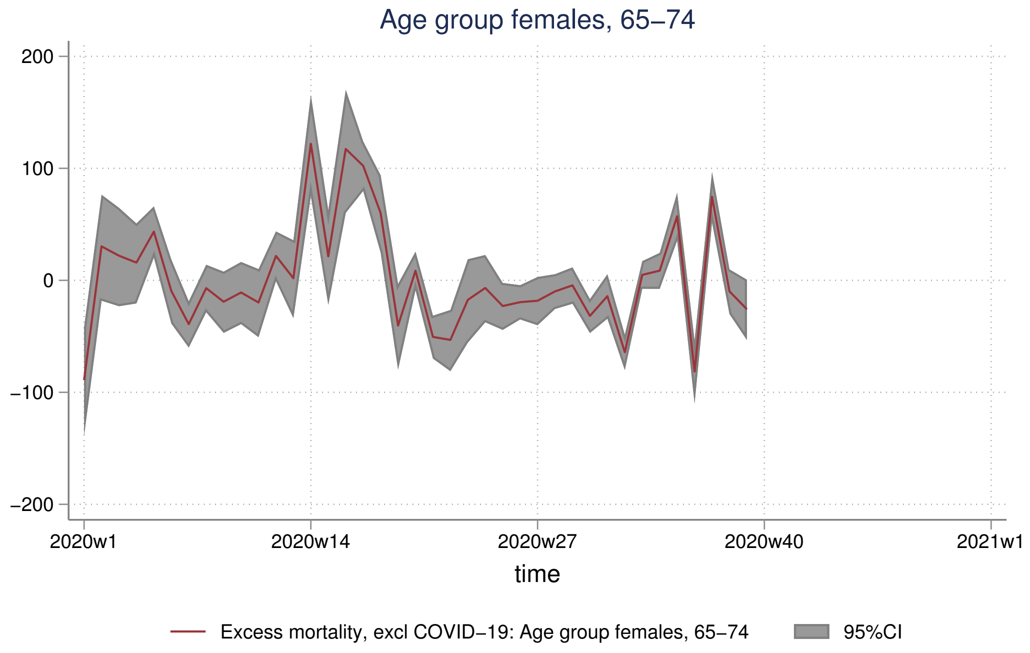

5.7 Females aged 75-84

Figure 289: Females 75-84 mortality time trend and model, fm 2010 wk1

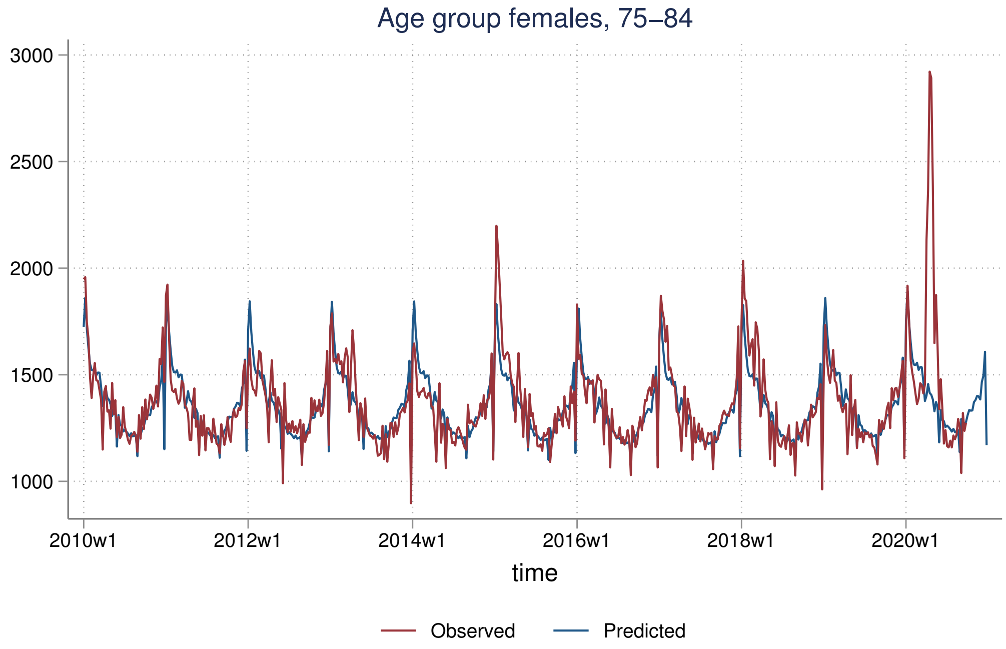

Figure 290: Females 75-84 mortality time trend and model, fm 2019 wk1

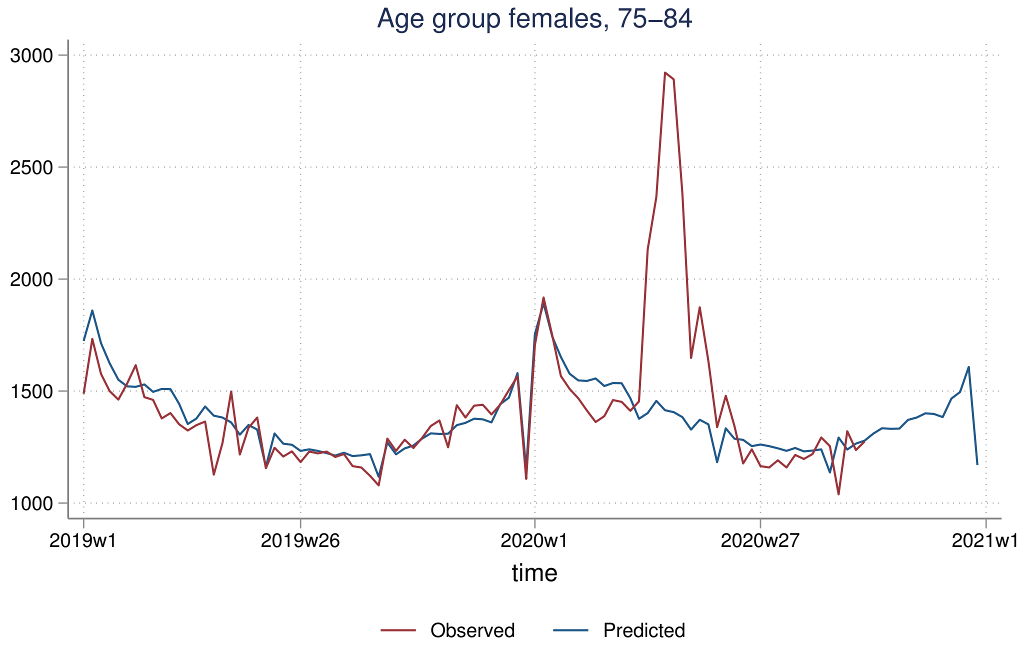

Figure 291: Females 75-84 mortality time trend and model, fm 2020 wk1

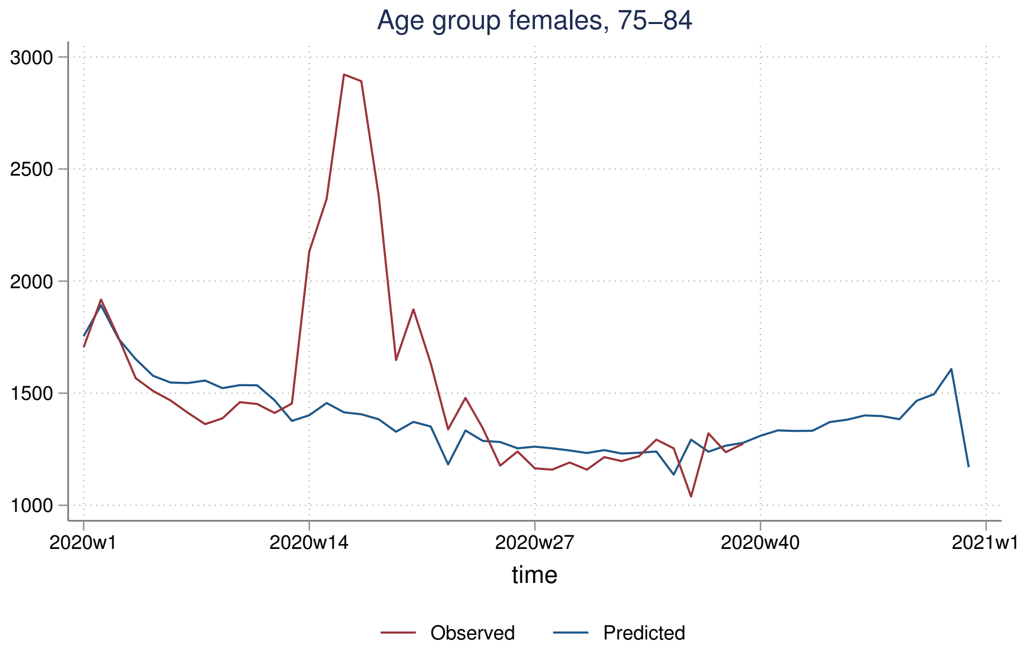

Figure 292: Females 75-84 all-cause excess deaths, fm 2010 wk1

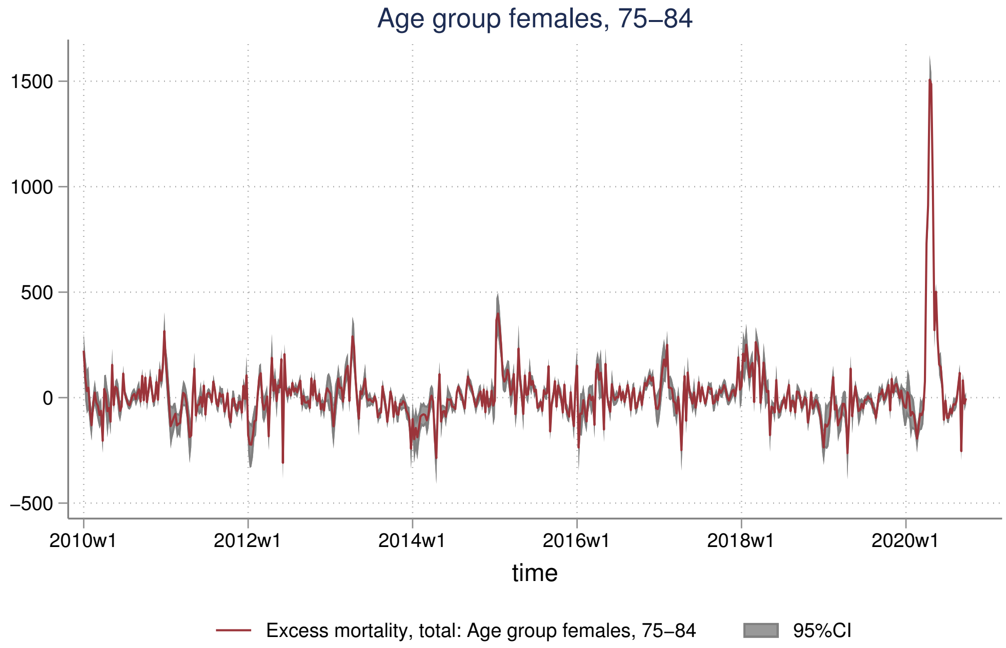

Figure 293: Females 75-84 all-cause excess deaths, fm 2019 wk1

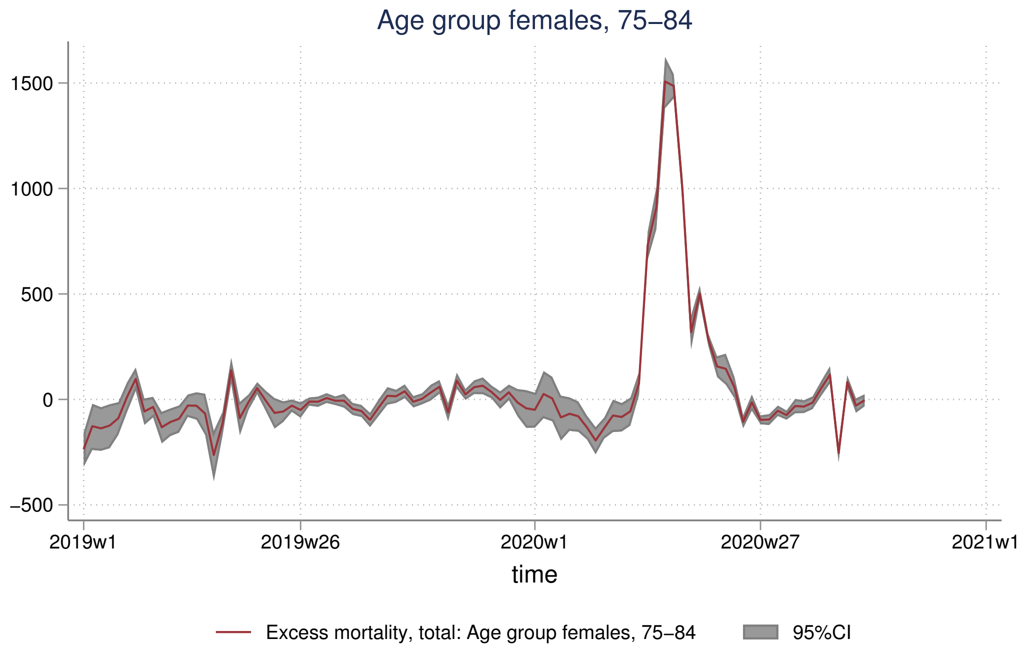

Figure 294: Females 75-84 all-cause excess deaths, fm 2020 wk1

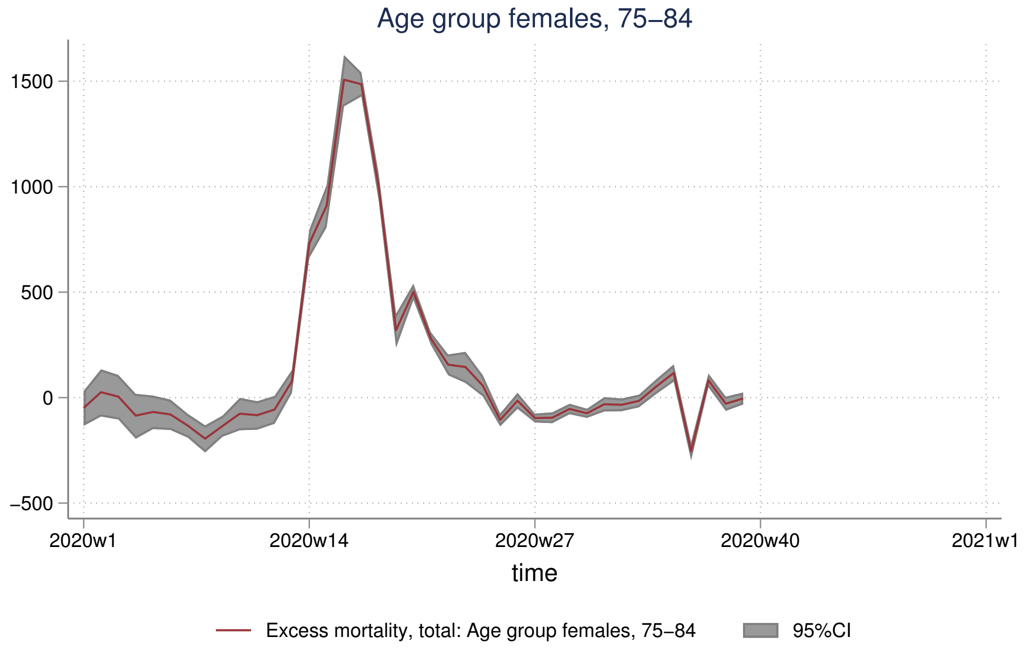

Figure 295: Females 75-84 all-cause excess deaths (−COVID19), fm 2010 wk1

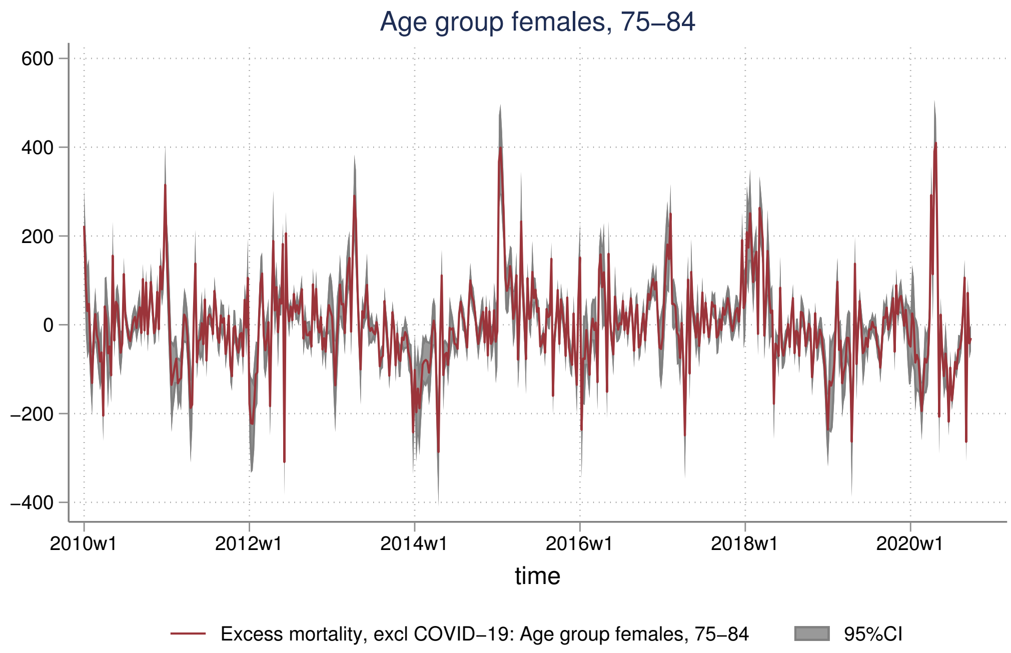

Figure 296: Females 75-84 all-cause excess deaths (−COVID19), fm 2019 wk1

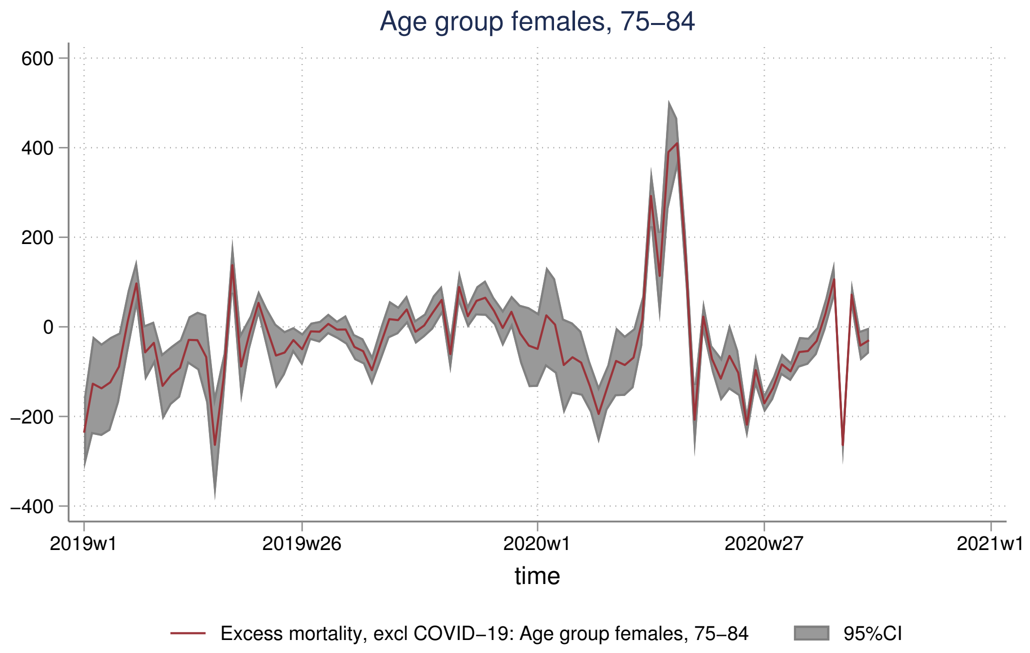

Figure 297: Females 75-84 all-cause excess deaths (−COVID19), fm 2020 wk1

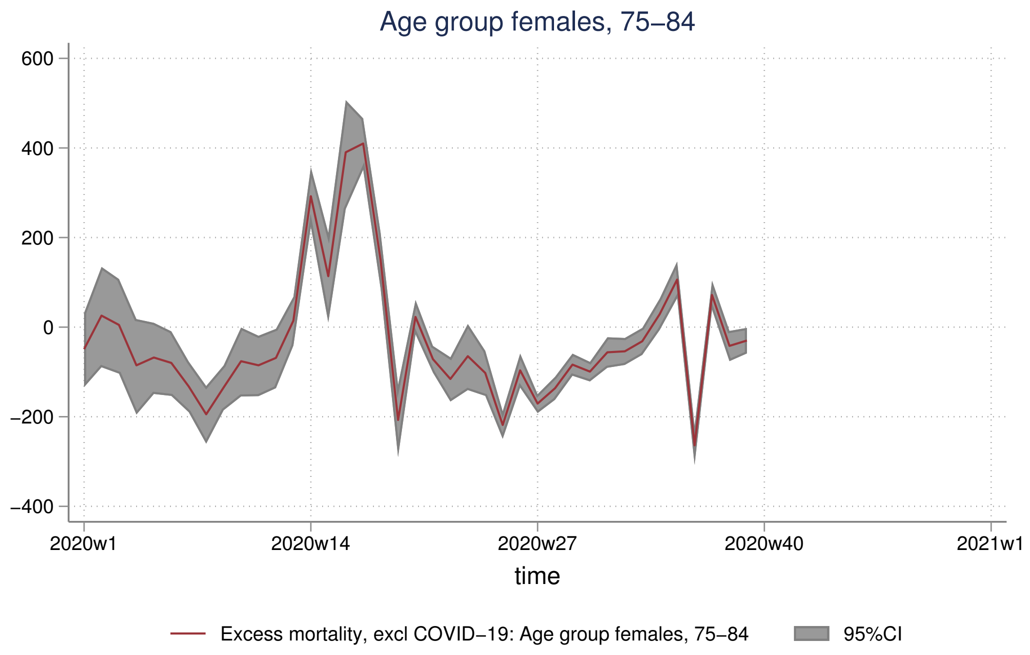

5.8 Females aged 85+

Figure 298: Females 85+ mortality time trend and model, fm 2010 wk1

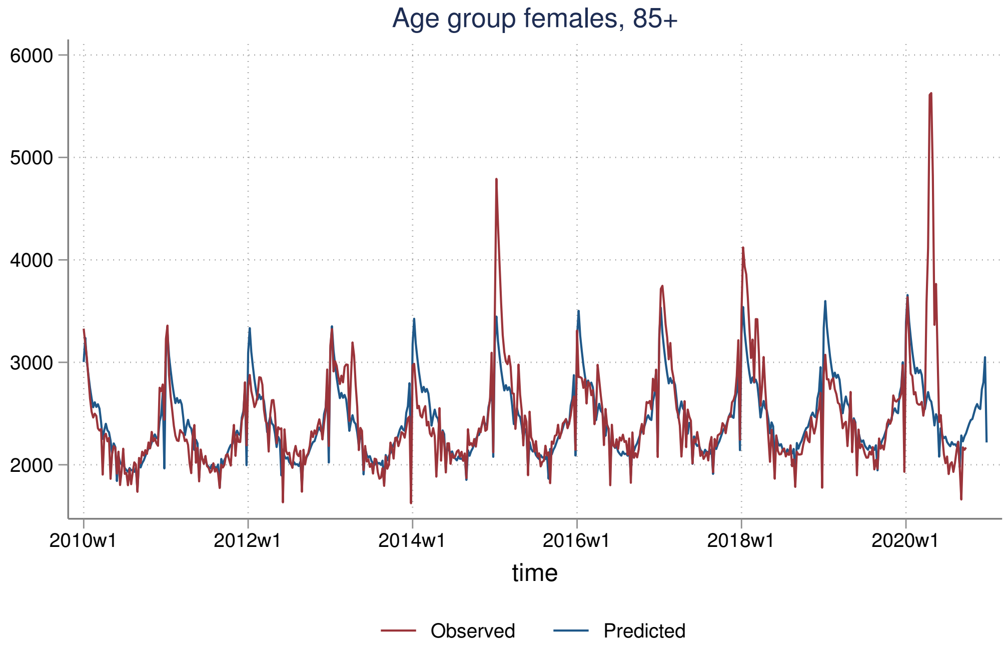

Figure 299: Females 85+ mortality time trend and model, fm 2019 wk1

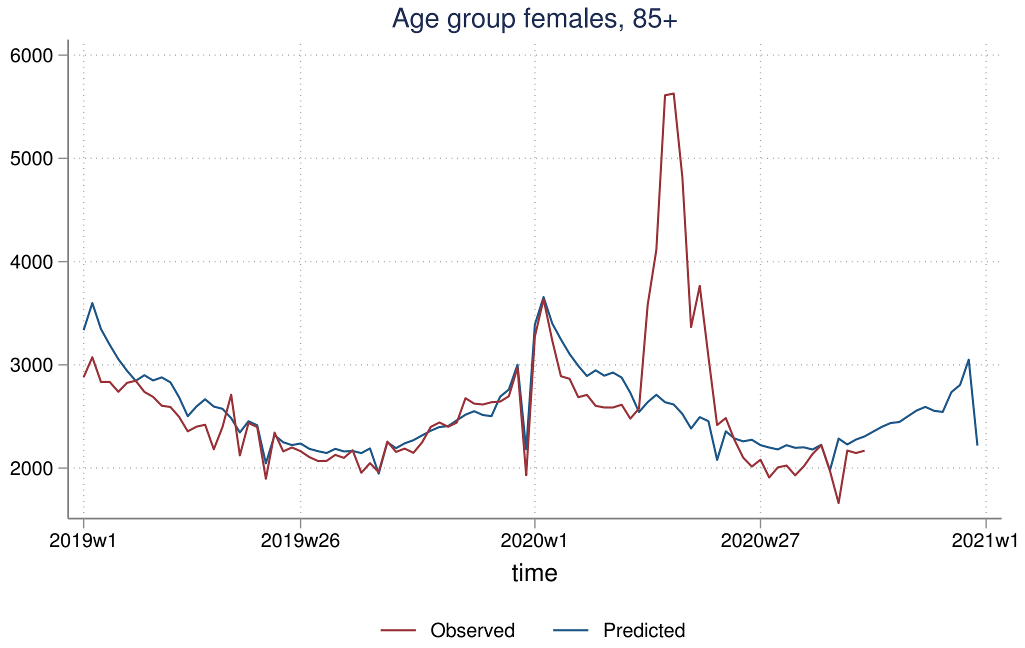

Figure 300: Females 85+ mortality time trend and model, fm 2020 wk1

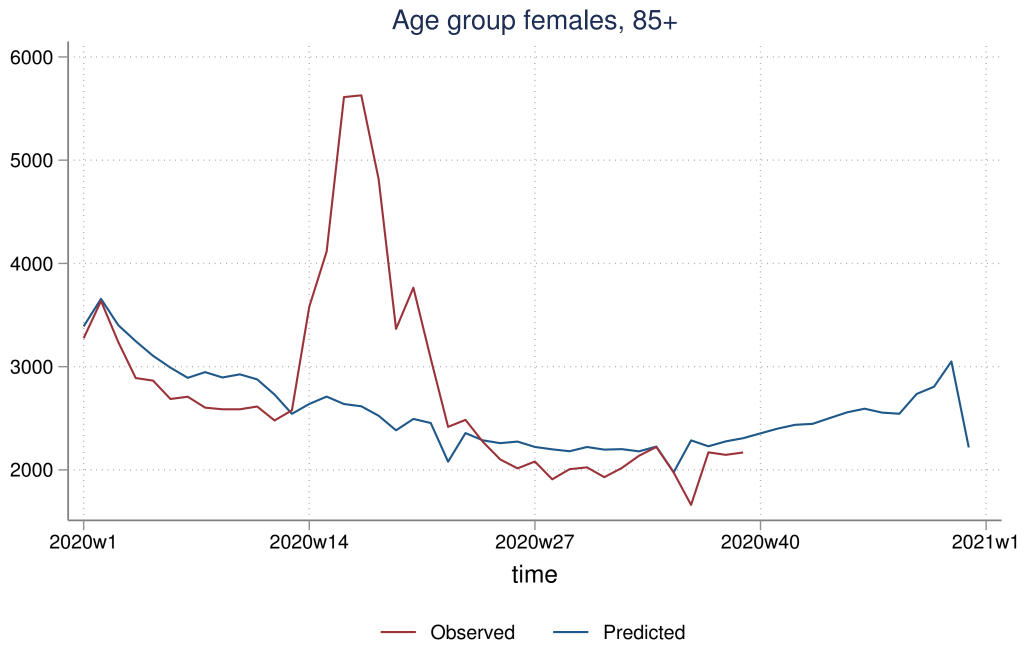

Figure 301: Females 85+ all-cause excess deaths, fm 2010 wk1

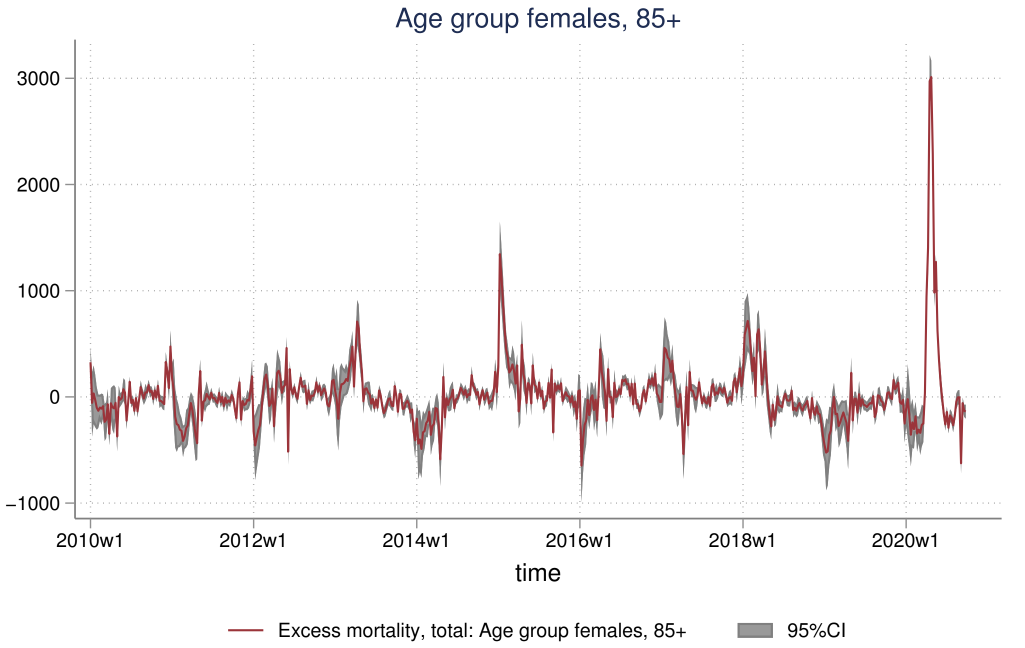

Figure 302: Females 85+ all-cause excess deaths, fm 2019 wk1

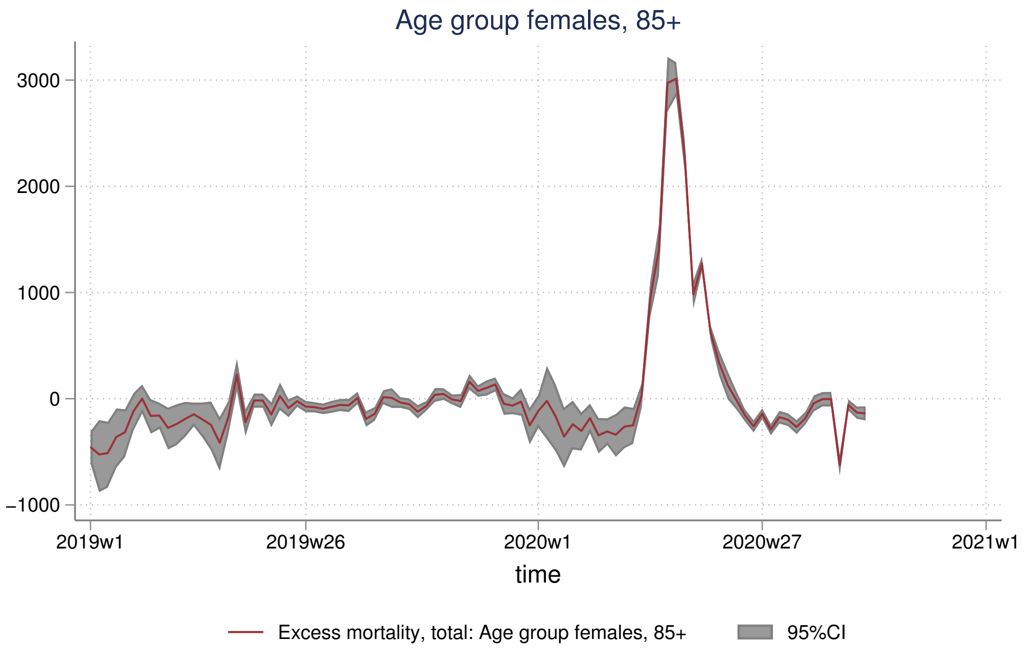

Figure 303: Females 85+ all-cause excess deaths, fm 2020 wk1

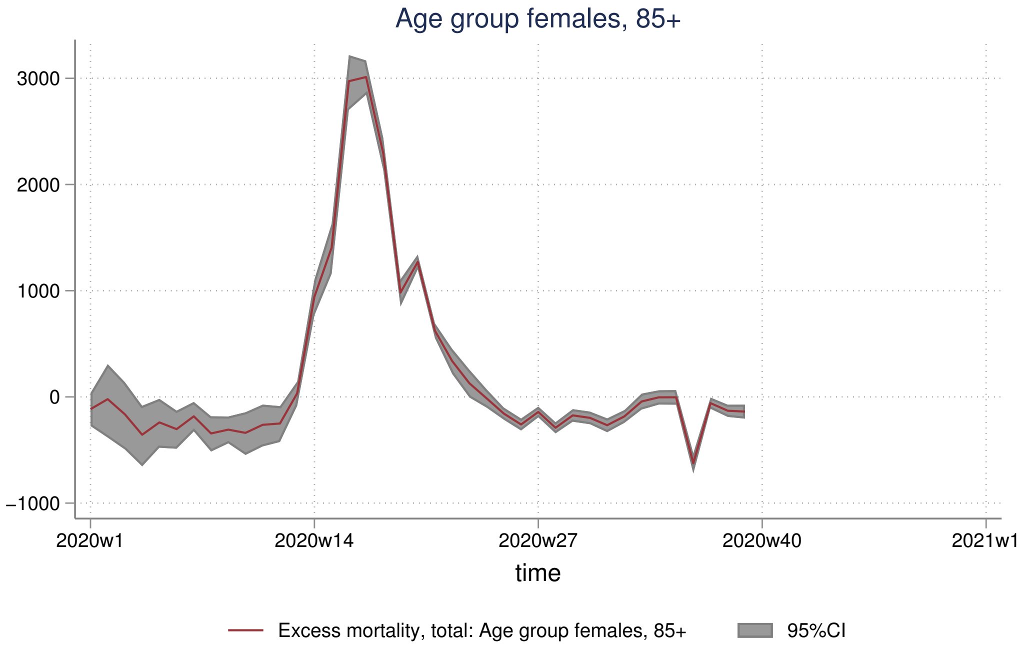

Figure 304: Females 85+ all-cause excess deaths (−COVID19), fm 2010 wk1

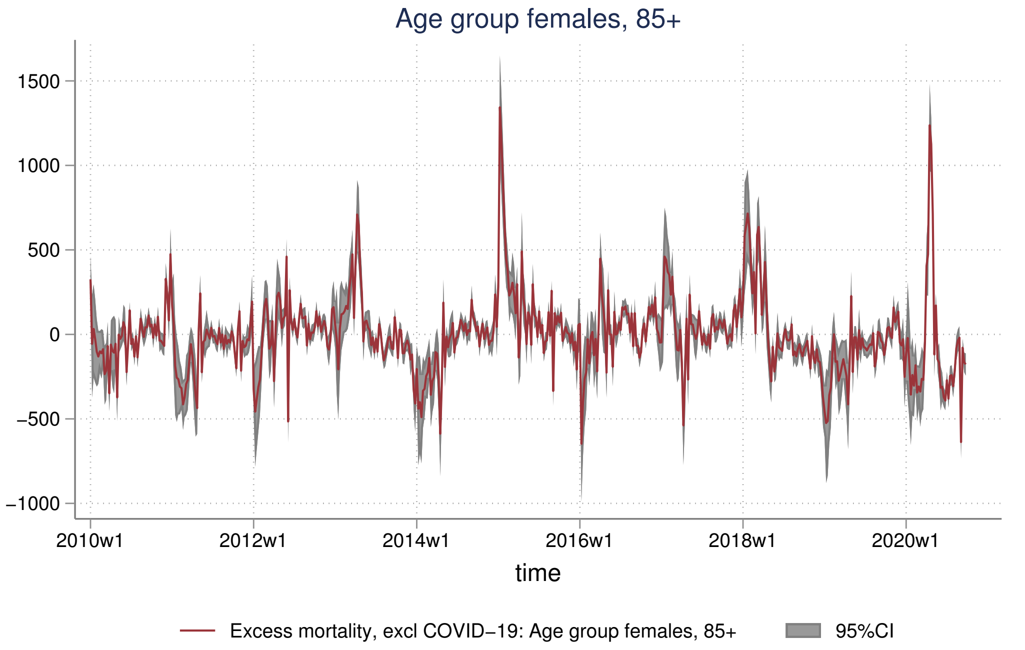

Figure 305: Females 85+ all-cause excess deaths (−COVID19), fm 2019 wk1

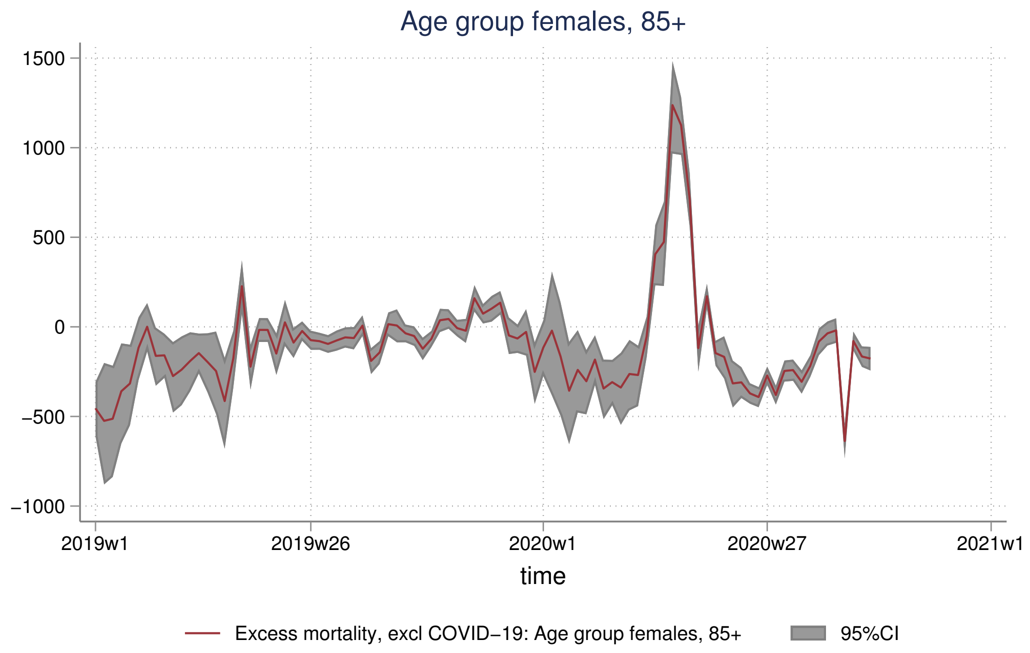

Figure 306: Females 85+ all-cause excess deaths (−COVID19), fm 2020 wk1

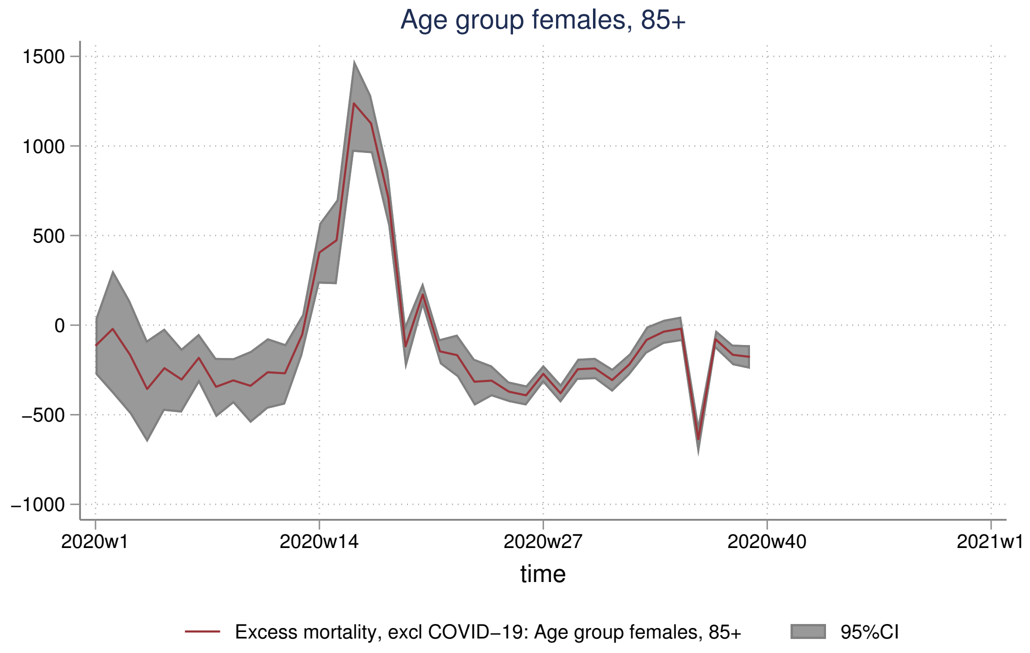

References

ONS (2020), *User guide to mortality statistics.* Accessed: 2020-05-06.
URL: *https://www.ons.gov.uk/peoplepopulationandcommunity/birthsdeathsandmarriages/deaths/methodologies/userguidetomortalitystatisticsjuly2017*
