# Supplementary material for: Excess mortality in England and Wales during the first wave of the COVID-19 pandemic
Source: J Epidemiol Community Health. 2020 Oct 15;75(3):213–23. doi: 10.1136/jech-2020-214764 (PMC7892396; doi:10.1136/jech-2020-214764)

Appendix 1 to excess mortality in England and Wales during the 2020 COVID19 pandemic

Evangelos Kontopantelis
Mamas A Mamas
John Deanfield
Miqdad Asaria
Tim Doran

October 7, 2020

Contents

1 All-cause excess deaths, from 2020 week 1 **2**

2 All-cause minus COVID-19 related excess deaths, from 2020 week 1 **4**

3 All-cause excess deaths, per $100,000$ **population, from 2020 week 1** **6**

4 All-cause excess deaths minus COVID-19 related, per $100,000$ **population, from 2020 week 1** **8**

1 All-cause excess deaths, from 2020 week 1

Figure 1: All-cause excess deaths, by region

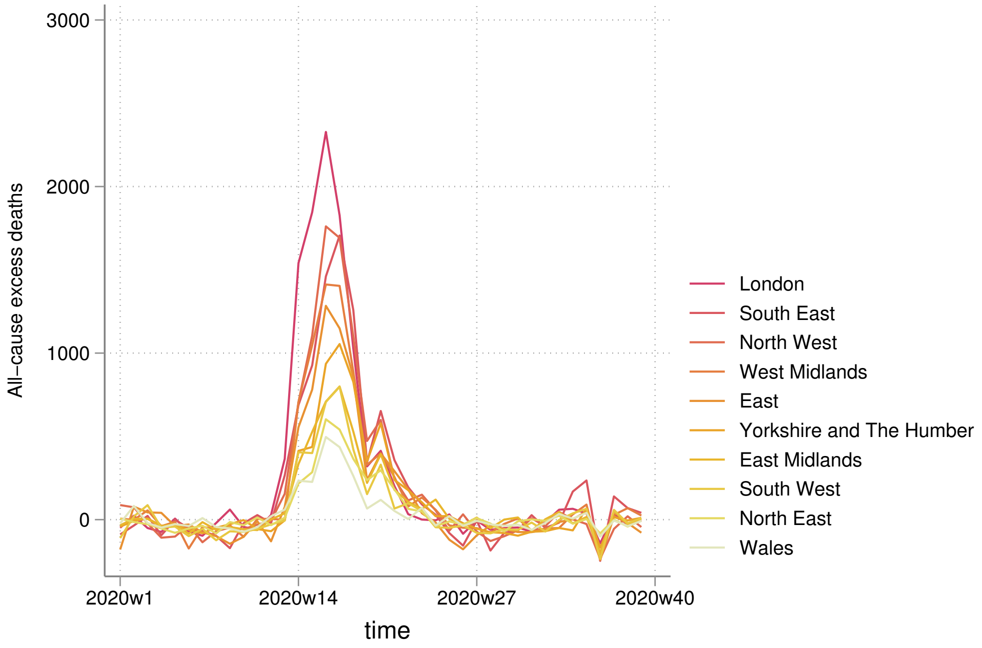

Figure 2: All-cause excess deaths, by age group

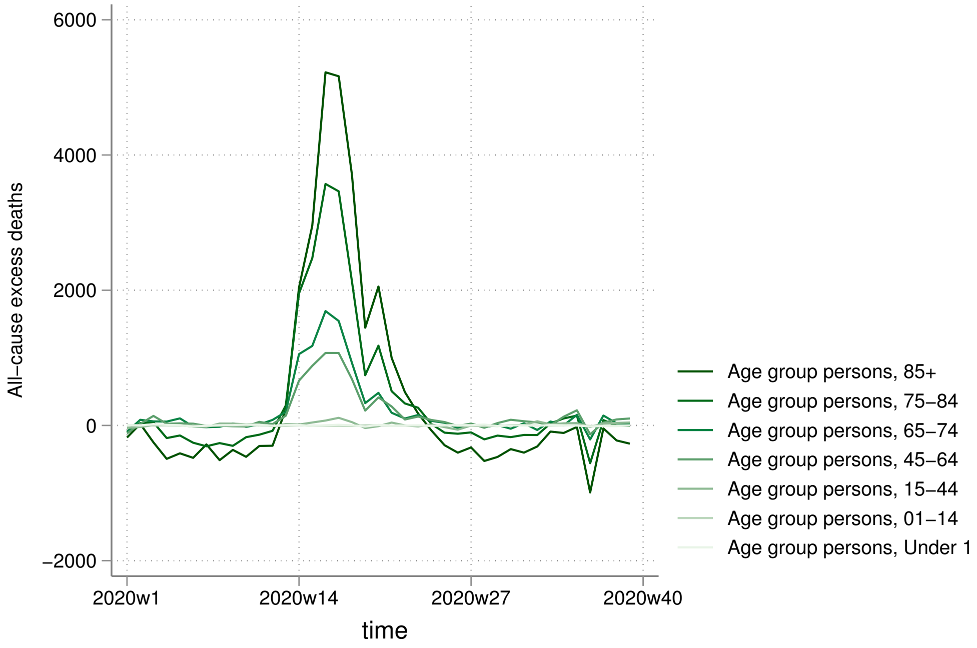

Figure 3: All-cause excess deaths, by age group and sex

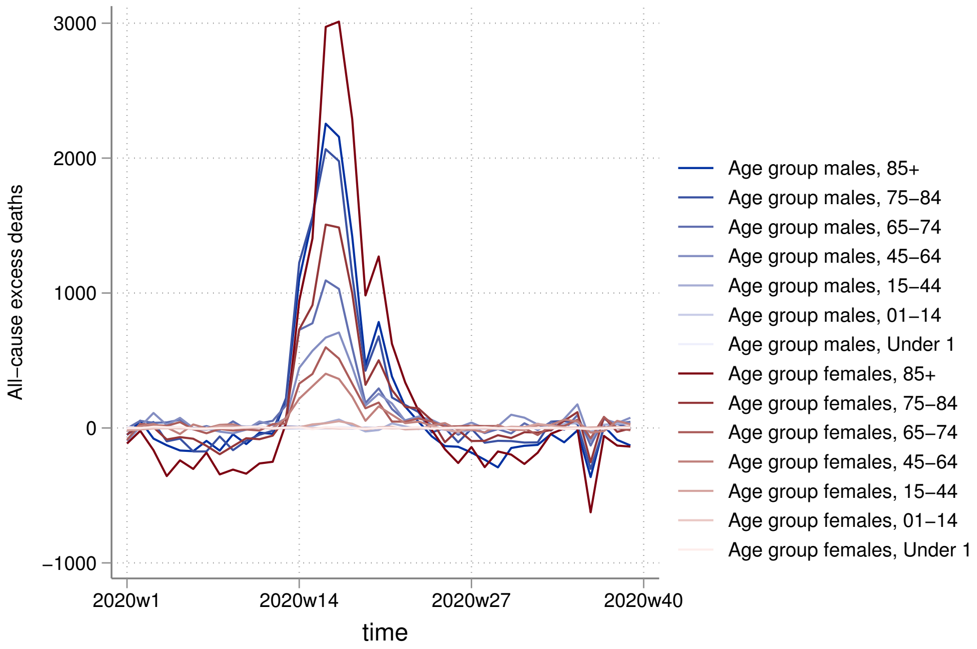

2 All-cause minus COVID-19 related excess deaths, from 2020 week 1

Figure 4: All-cause minus COVID-19 excess deaths, by region

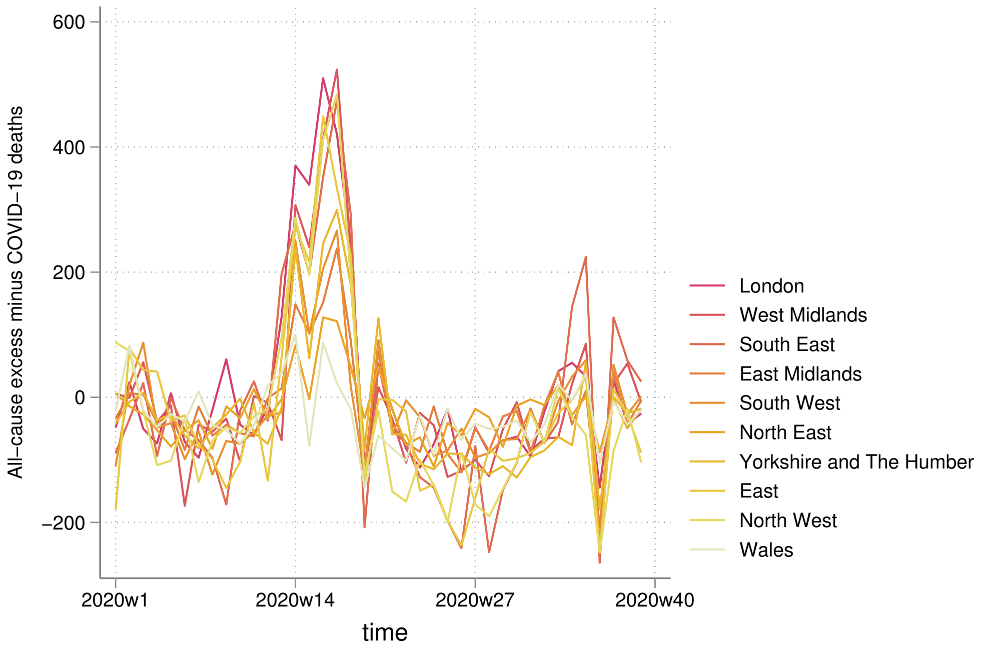

Figure 5: All-cause minus COVID-19 excess deaths, by age group

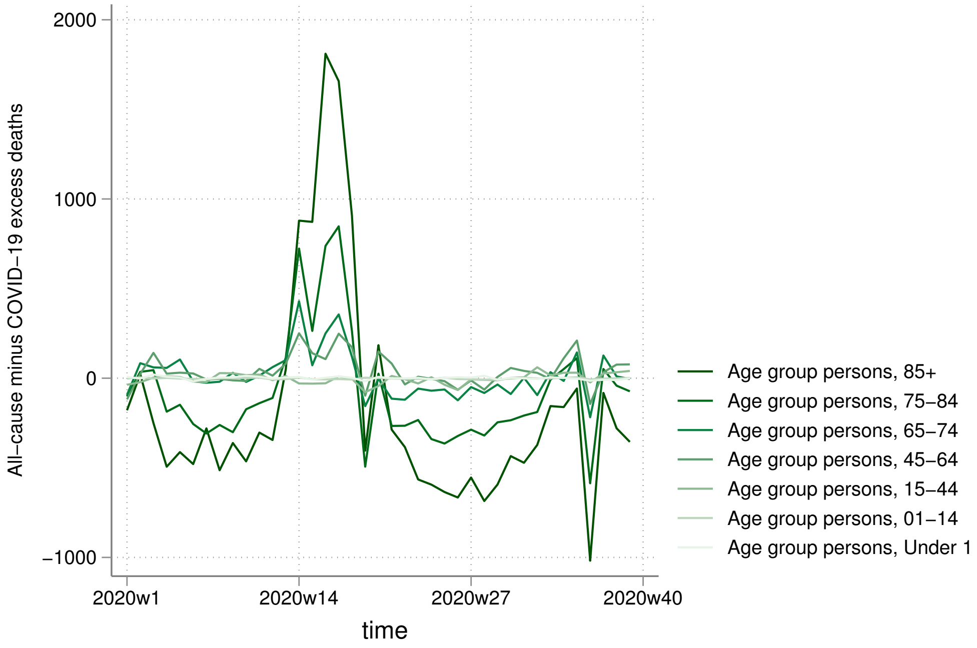

Figure 6: All-cause minus COVID-19 excess deaths, by age group and sex

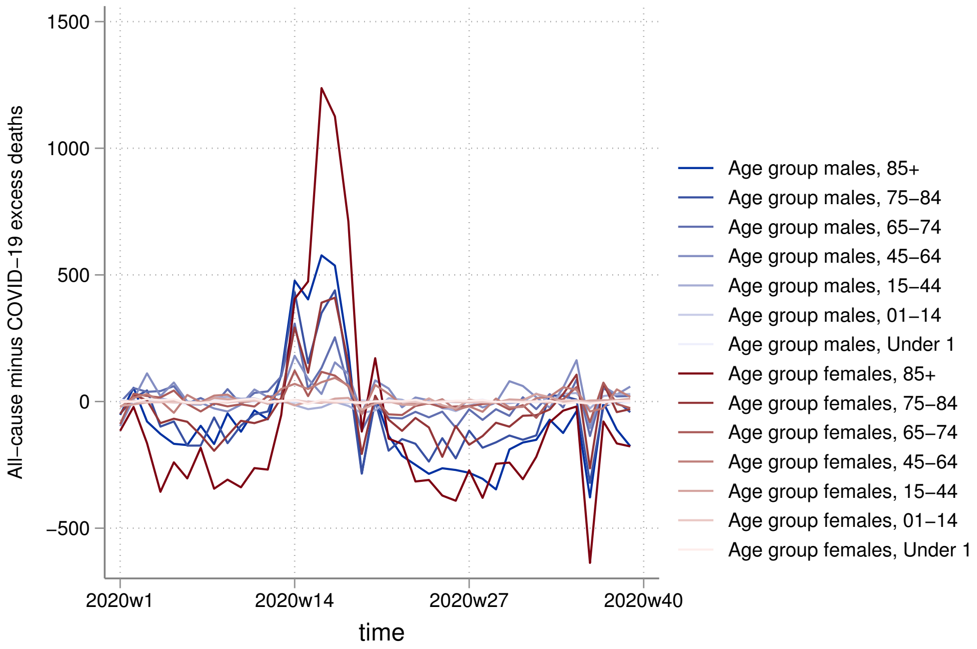

3 All-cause excess deaths, per $100,000$ population, from 2020 week 1

Figure 7: All-cause excess deaths per 100k, by region

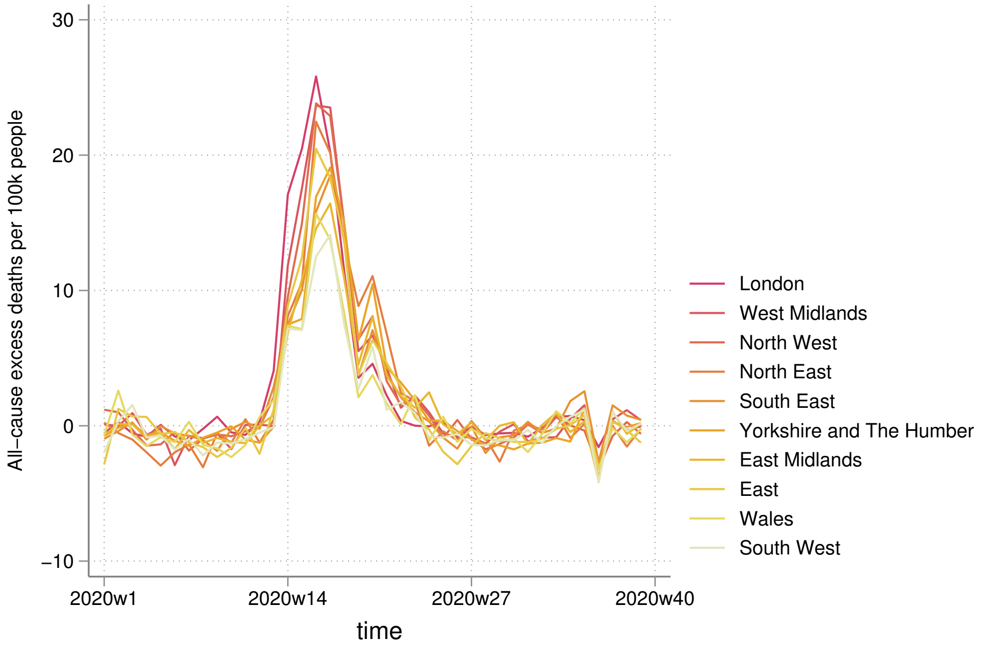

Figure 8: All-cause excess deaths per 100k, by age group

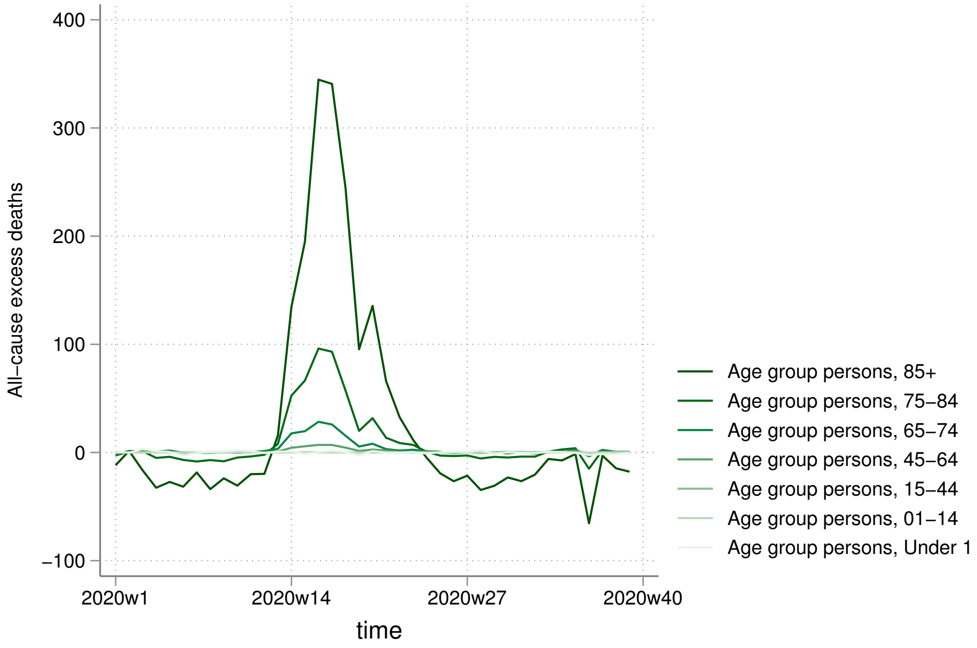

Figure 9: All-cause excess deaths per 100k, by age group and sex

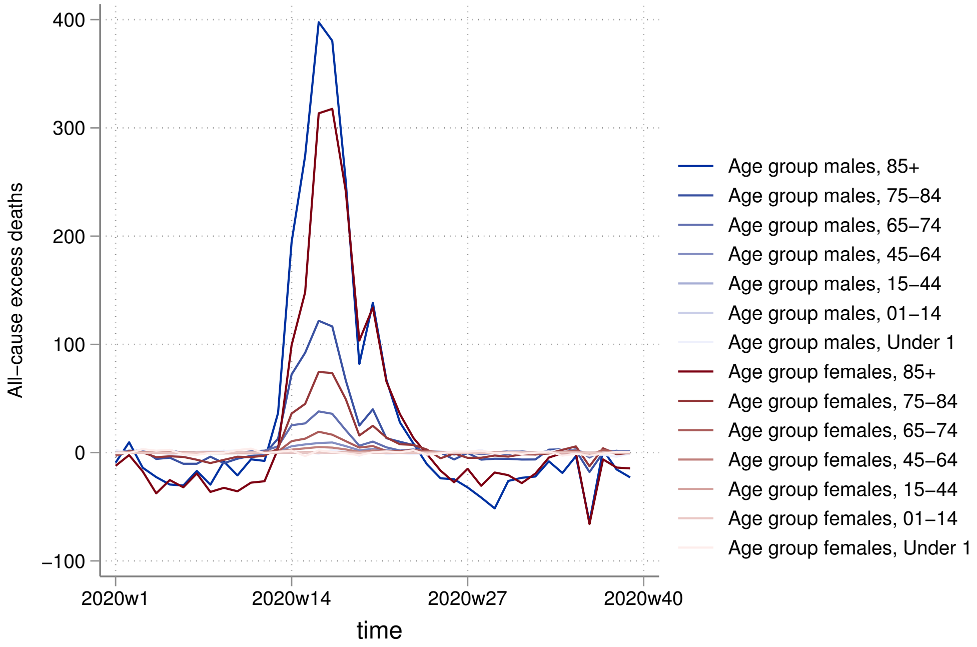

4 All-cause excess deaths minus COVID-19 related, per $100,000$ population, from 2020 week 1

Figure 10: All-cause minus COVID-19 excess deaths per 100k, by region

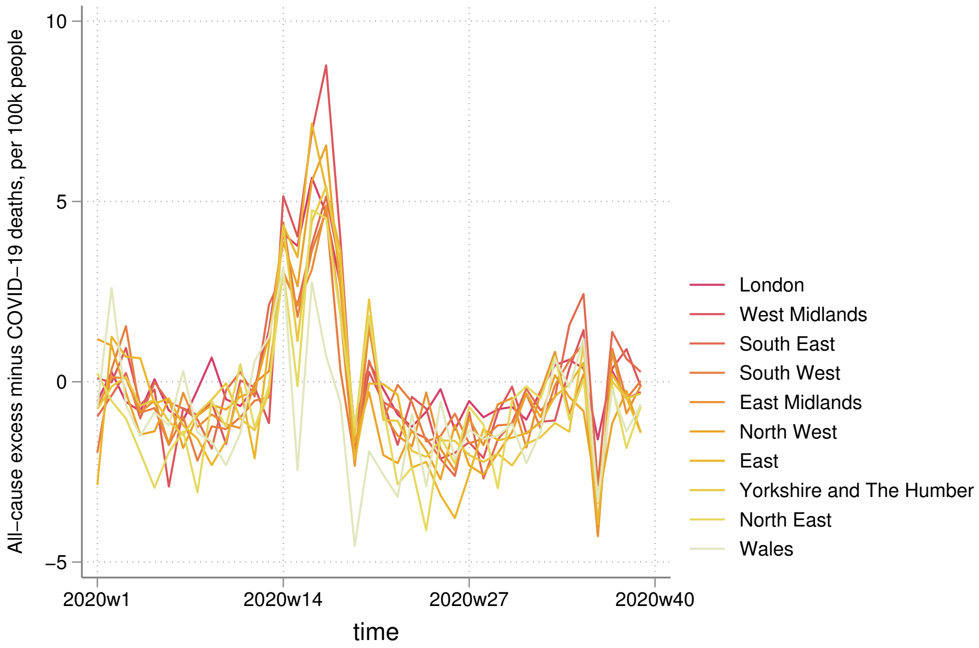

Figure 11: All-cause minus COVID-19 excess deaths per 100k, by age group

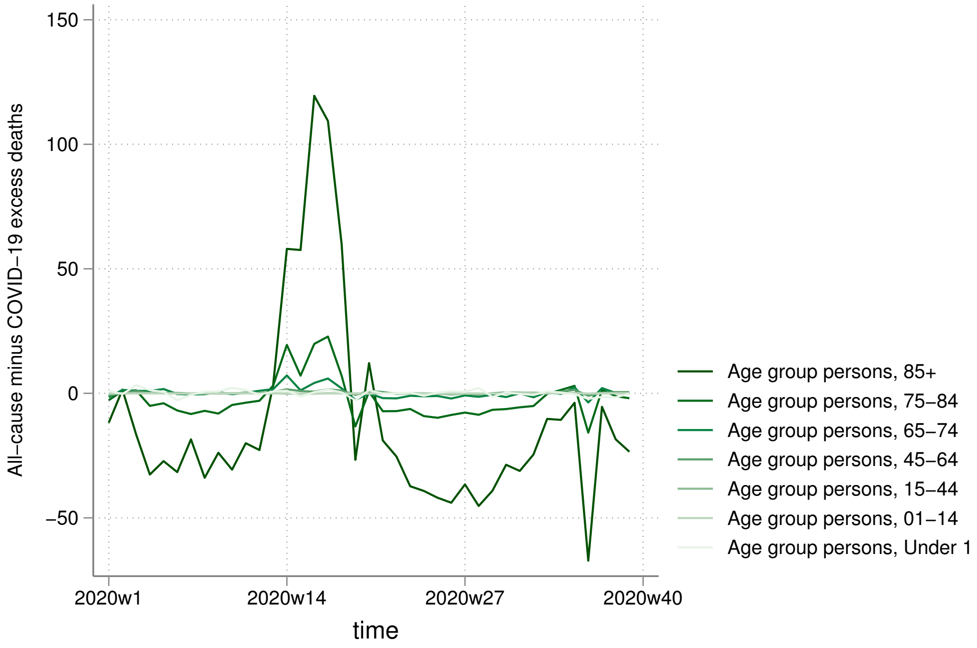

Figure 12: All-cause minus COVID-19 excess deaths per 100k, by age group and sex

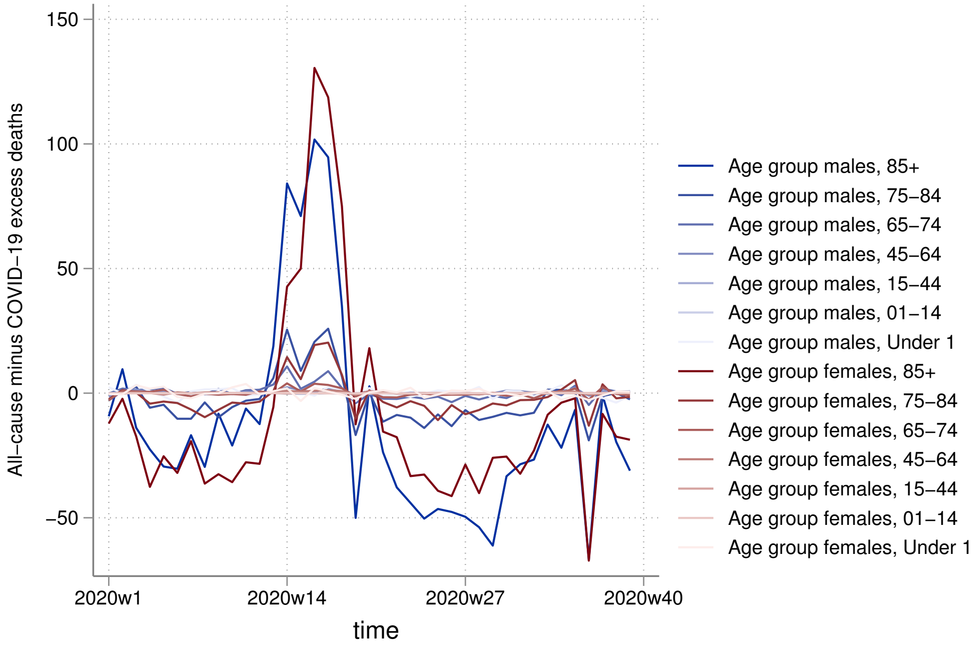

Supplement: Supplementary file 3 [file jech-2020-214764supp001.pdf]
